# Geography, environment and organismal traits in the diversification of a major tropical herbaceous angiosperm radiation

**DOI:** 10.1093/aobpla/ply008

**Published:** 2018-01-30

**Authors:** Jamie Males

**Affiliations:** Department of Plant Sciences, University of Cambridge, Downing Street, Cambridge, UK

**Keywords:** Bioclimate, Bromeliaceae, diversity, Neotropics, niche differentiation, water-use strategies

## Abstract

The generation of plant diversity involves complex interactions between geography, environment and organismal traits. Many macroevolutionary processes and emergent patterns have been identified in different plant groups through the study of spatial data, but rarely in the context of a large radiation of tropical herbaceous angiosperms. A powerful system for testing interrelated biogeographical hypotheses is provided by the terrestrial bromeliads, a Neotropical group of extensive ecological diversity and importance. In this investigation, distributional data for 564 species of terrestrial bromeliads were used to estimate variation in the position and width of species-level hydrological habitat occupancy and test six core hypotheses linking geography, environment and organismal traits. Taxonomic groups and functional types differed in hydrological habitat occupancy, modulated by convergent and divergent trait evolution, and with contrasting interactions with precipitation abundance and seasonality. Plant traits in the Bromeliaceae are intimately associated with bioclimatic differentiation, which is in turn strongly associated with variation in geographical range size and species richness. These results emphasize the ecological relevance of structural-functional innovation in a major plant radiation.

## Teaser

The terrestrial bromeliads are a diverse group of plants native to the Americas. In this article, the distributions of 564 species are analysed to answer key questions about plant–environment interactions. Contrasting biological characteristics can explain the occurrence of different species in different climatic zones, and species native to arid zones appear to be particularly specialized. Species which are more tolerant of a range of climates tend to occur across larger geographical ranges, and closely related species in large genera tend to show differentiation in their climatic preferences.

## Introduction

Generation and maintenance of diversity of plant lineages often involves complex interactions between biogeography, climate and plant traits. The study of these interactions has generated a range of hypotheses and theories, such as those which deal with the role of trait-based niche specialization in shaping species distributions (e.g. Slayter *et al.* 2013), or with latitudinal gradients in geographical range sizes (e.g. Hulshof *et al.* 2013). While targeted analyses of the validity of specific evolutionary biogeographical hypotheses are common, there are comparatively few instances of in-depth case studies being used to examine simultaneously the relevance and relative importance of a range of such hypotheses within a particular taxonomic group. This is particularly true in the context of tropical herbaceous angiosperms, despite the fact that this functional group accounts for a high proportion of global floristic diversity and provides a wealth of underappreciated ecosystem functions (Ewel and Bigelow 1996; Dodd *et al.* 1999; Royo and Carson 2006).

An excellent system in which to explore the relevance of fundamental biogeographical and ecological hypotheses to the diversity of tropical herbaceous angiosperms is the Neotropical Bromeliaceae (Poales). This monocot family includes some 3500 species (Butcher and Gouda, cont. updated), which display highly contrasting growth forms and ecologies, and have diversified rapidly and recently (Givnish *et al.* 2011, 2014). Convergent origins of morphological and physiological key innovations including epiphytism, crassulacean acid metabolism (CAM) and the tank growth form have significantly impacted on the diversification dynamics of specific bromeliad lineages (Benzing 2000; Crayn *et al.* 2004; Givnish *et al.* 2014; Silvestro *et al.* 2014), and are used to define a series of functional types (Pittendrigh 1948; Benzing 2000; Males 2016). Process-based studies of the impact of structural-functional innovation on bromeliad ecophysiological diversity are gaining renewed attention (Males 2016; Palma-Silva *et al.* 2016), but effective contextualization of such studies requires a clear picture of the biogeography and bioclimatology of bromeliad taxonomic groups and functional types. Various efforts have been made to reconstruct the historical biogeography of the Bromeliaceae and subfamilial lineages (e.g. Jabaily and Sytsma 2010, 2013; Givnish *et al.* 2011; Versieux *et al.* 2012; Wagner *et al.* 2013b), and present-day distributional patterns have been considered in general discussions of bromeliad biology (e.g. Benzing 2000). However, while it is widely acknowledged that the bromeliads occupy a remarkably diverse range of environments, there are no published large-scale analyses of variation in the geographic and climatological distributions of bromeliads. More complete, quantitative understanding of patterns in bromeliad species distributions is fundamental to understanding the relevance of divergences in ecophysiological traits for niche differentiation, and the degree of environmental specialization at different taxonomic levels (Silvertown *et al.* 2006).

**Figure 1.**
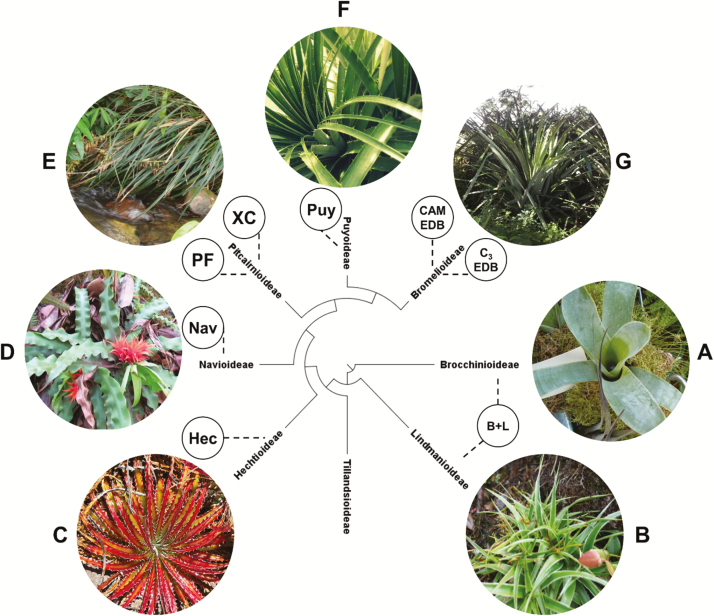
Distribution of major terrestrial lineages in the Bromeliaceae, with examples of morphological diversity. (A) *Brocchinia reducta* (Brocchinioideae)—photograph by BotBln (CC); (B) *Connellia quelchii* (Lindmanioideae)—photograph by Gérard Vigo (CC); (C) *Hechtia texensis* (Hechtioideae)—photograph by Stan Shebs (CC); (D) *Navia tentaculata* (Navioideae)—photograph by Thore Noernberg (CC); (E) *Pitcairnia ulei* (Pitcairnioideae)—photograph by João Medeiros (CC); (F) *Puya alpestris* (Puyoideae)—photograph by JM; (G) *Bromelia karatas* (Bromelioideae)—photograph by JM. Circles indicate phylogenetic positions of taxonomic groups mentioned in the text: B + L = Brocchinioideae and Lindmanioideae; Hec = *Hechtia*; Nav = Navioideae; PF = *Pitcairnia* and *Fosterella*; XC = Xeric Clade Pitcairnioideae; Puy = *Puya*; CAM EDB = CAM early-diverging Bromelioideae; C_3_ EDB = C_3_ early-diverging Bromelioideae. Phylogenetic relationships based on Givnish *et al.* (2011, 2014).

This investigation focuses on the terrestrial and saxicolous bromeliad lineages, in which species distributions are expected to be less strongly affected by microenvironmental factors than for epiphytic bromeliads (Pittendrigh 1948; Benzing 2000), and are therefore more amenable to analysis of environmental habitat occupancy (*sensu*Whittaker *et al.* 1973; Kearney 2006) based on distributional data. Terrestrial species dominate 6/8 bromeliad subfamilies, and can be divided into four functional types on the basis of photosynthetic pathway and leaf morphoanatomy: C_3_ mesic terrestrials, C_3_ succulent terrestrials, C_3_-CAM intermediate succulent terrestrials and CAM succulent terrestrials (cf. alternative schemes based on growth habit and water-uptake mechanism in Pittendrigh 1948; Benzing 2000; Males 2016). Some of the phylogenetic and morphological diversity in the terrestrial bromeliads is illustrated in Fig. 1.

For 564 species, distributional data were used to estimate geographic range sizes and quantify bioclimatic variables. Since water availability is recognized as a critical factor in bromeliad ecophysiological differentiation (Benzing 2000; Males 2016) and variation in species hydrological niche has been shown in other plant groups to be an important driver of distributional patterns and determinant of species coexistence (Silvertown *et al.* 1999; Ogle and Reynolds 2004; Araya *et al.* 2011), estimation of differences in habitat occupancy was focussed on hydrological factors (mean annual precipitation, MAP; precipitation in driest month, *P*_dry_; precipitation seasonality, *P*_seas_; aridity index, AI; ratio of actual evapotranspiration to potential evapotranspiration, AET/PET). Using these data, six core hypotheses were tested, linking biogeography, climate and plant traits. It was expected that analyses would identify differentiation in habitat position (mean value) and range among terrestrial bromeliad taxonomic groups and functional types, and that key plant traits could explain this. It was also hypothesized that habitat overlap would be greater among species in lineages that display more specialized biotic interactions, while species from more arid environments would show narrow habitat ranges as a result of environmental specialization. Finally, positive correlations were expected between habitat range and geographical range size, and between species diversity and diversity in habitat position and range within genera. These hypotheses are summarized, with supporting references, in Table 1.

**Table 1.** Fundamental hypothetical relationships tested in this investigation, linking biogeography, climate and plant traits.

Hypothesis	Rationale	References
1. Major taxonomic groups and functional types differ significantly in habitat position and range	Consequence of adaptive ecological diversification associated with niche evolution and possible biome shifts	Donoghue and Edwards (2014)
2. Convergent plant traits are associated with convergent patterns in habitat occupancy	Independent evolutionary origins of key traits facilitate equivalent transitions across environmental space	Grime (2006); Freschet *et al.* (2011); Ogburn and Edwards (2013)
3. The degree of habitat overlap is higher in taxonomic groups with more specialized biotic interactions	Sharp differentiation of biotic niche may permit sympatric coexistence under a shared environmental regime	Johnson (2010); Pauw (2013)
4. Species occupying more arid habitats will tend to show narrower habitat ranges	Extreme environments favour evolution of ecological specialists	Thuiller *et al.* (2004); Carboni *et al.* (2016)
5. Geographical range size is positively correlated with hydrological habitat range	Species that are tolerant of a wider range of environments are able to colonize wider geographical regions	Morin and Chuine (2006); Essl *et al.* (2009); Banta *et al.* (2012); Slayter *et al.* (2013)
6. Larger genera will tend to show greater variety in habitat position and range	Accumulation of species diversity through climatic niche evolution	Kozak and Wiens (2010); Schnitzler *et al.* (2012)

This study found clear evidence of divergences in hydrological habitat position and range among taxonomic and functional groups of terrestrial bromeliads, with convergent and divergent bioclimatic relations being associated with key plant traits. Taxonomic groups with more specialized biotic interactions tended to show greater habitat overlap, while narrow habitat ranges were observed in species native to more arid habitats. There was a clear correlation between geographical range size and habitat range, and more species-rich genera showed greater diversity in both habitat position and range. These results emphasize the structured complexity of bioclimatic interactions in a family which is rapidly emerging as a model system in tropical herbaceous angiosperm evolutionary ecology and physiology (Males 2016; Palma-Silva *et al.* 2016).

## Methods

### Taxon sampling

All terrestrial (and saxicolous) bromeliads in the following groups were considered in this investigation: Brocchinioideae (*Brocchinia*); Lindmanioideae (*Connellia*, *Lindmania*); Hechtioidae (*Hechtia*); Navioideae (*Brewcaria*, *Cottendorfia*, *Navia*, *Sequencia*, *Steyerbromelia*); Pitcairnioideae (*Deuterocohnia*, *Dyckia*, *Encholirium*, *Fosterella*, *Pitcairnia*); Puyoideae (*Puya*); and early-diverging Bromelioideae (*Ananas*, *Bromelia*, *Cryptanthus*, *Deinacanthon*, *Disteganthus*, *Fascicularia*, *Fernseea*, *Greigia*, *Neoglaziovia*, *Ochagavia*, *Orthophytum*). A complete list of currently recognized (February 2017) taxon names for each of these genera was generated using The Plant List (2013) and the Bromeliad Taxon List (Butcher and Gouda, cont. updated). The saxicolous species in the Tillandsioideae subfamily (e.g. *Alcantarea* spp., *Vriesea* spp.), many of which are narrow environmental endemics (Versieux *et al.* 2012; da Costa *et al.* 2014), were not included because they form a relatively minor component of the overwhelmingly epiphytic Tillandsioideae.

Species were assigned to functional types according to the presence or absence of morphological succulence and photosynthetic pathway information from the carbon isotope ratio data set of Crayn *et al.* (2015). The total data set included 261 C_3_ mesic terrestrials, 97 C_3_ succulent terrestrials, 11 C_3_-CAM succulent terrestrials and 195 CAM succulent terrestrials (for further details **see Supporting Information—Table S1**).

### Collection and processing of distributional data

Species names were used to query the Global Biodiversity Information Facility (GBIF) for distributional data. For species for which three or more georeferenced presence points were available, data were downloaded and subjected to manual quality control. Any presence points lying obviously outside of the native range of the species (e.g. on another continent) were removed, as were those corresponding to the geographical locations of herbaria or living collections. Duplicate records were also removed. The total number of species used in subsequent analyses was 564. The effects of sample size (number of presence points) on habitat occupancy metrics were analysed **[see Supporting Information—Table S2]**. The limitations of this approach and a comparison with alternative approaches based on species distribution modelling are covered in the Discussion.

### Preparation of bioclimatic data

Bioclimatic layers (MAP; precipitation in driest month, *P*_dry_; and precipitation seasonality, *P*_seas_) were downloaded from the Bioclim database (Hijmans *et al.* 2005) at 30 arc-second resolution. Aridity index, actual evapotranspiration (AET) and potential evapotranspiration (PET) layers were obtained at the same resolution from the CGIAR-CSI portal (Zomer *et al.* 2007, 2008). The Bioclim and CGIAR-CSI data are independent. The bioclimatic variables selected for hydrological habitat position and range estimation are shown, with the rationale for their inclusion, in Table 2.

**Table 2.** Bioclimatic variables used in terrestrial bromeliad hydrological habitat position and range analysis, showing rationale for inclusion and source of data.

Variable	Definition	Rationale	Source
MAP	Mean annual precipitation, mm	Proxy for the absolute quantity of water available during each year. Species may differ in the absolute quantity of water required to maintain turgor and transpiration.	Bioclim (Hijmans *et al.* 2005)
AI	Aridity index, mm mm^−1^	Proxy for the degree of dryness. Species may respond differently to chronic water deficit depending on morphological, anatomical and physiological specialization.	CGIAR-CSI (Zomer *et al.* 2007, 2008)
AET/PET	Actual evapotranspiration/potential evapotranspiration, mm mm^−1^	Proxy for plant water supply relative to demand. Species may differ in their requirements depending on water use and hydraulic characteristics.	CGIAR-CSI (Zomer *et al.* 2007, 2008)
*P*_dry_	Precipitation in driest month, mm	Proxy for the absolute degree of water limitation during the dry season. Species may differ in the minimum quantity of dry-season precipitation required to maintain physiological function.	Bioclim (Hijmans *et al.* 2005)
*P*_seas_	Precipitation seasonality, %	Proxy for the intensity of the dry season relative to the remainder of the year. Species may differ in their requirement for environmental equability throughout the year.	Bioclim (Hijmans *et al.* 2005)

A script was compiled in R (R Development Core Team 2008) using the ‘raster’ package (Hijmans *et al.* 2017) to enable automated retrieval of the value of each bioclimatic variable at each presence point. For each species, the mean and range were then calculated across all values of each bioclimatic variable. With the exception of MAP and AI (*r*^2^ = 0.92), pairwise non-linear and linear regression analyses showed that bioclimatic variables were not strongly correlated (*r*^2^ < 0.70).

### Assessment of hydrological habitat position and range

The mean and range of individual bioclimatic variables were utilized as univariate indicators of species’ hydrological habitat position and range. To estimate multivariate hydrological habitat position scores, the mean values of all environmental variables were log-transformed and subjected to principal component analysis (PCA) in R. Species scores in the climate space defined by the first two principal components (PC1, PC2) were then used as comparative estimates of hydrological habitat position scores. The same PCA-based procedure was used to calculate estimates of multivariate hydrological habitat ranges.

### Assessment of overlap between univariate habitat indicator ranges

In order to determine the extent of hydrological habitat overlap within taxonomic groups, a custom-designed function based on the ‘proxy’ and ‘stats’ packages was implemented in R. The script generated pairwise distance matrices for all species within a taxonomic group, with the distance function set to calculate the absolute extent of overlap between minimum and maximum indicator values for either species. The mean and standard deviation were calculated across the entire resultant matrix to provide measures of the extent and variation in pairwise species-level overlap. For each group, the mean extent of overlap was then normalized by dividing by the mean species-level range in that indicator variable in that taxonomic group. Because of the collinearity between many of the indicator variables (see below), only AI and *P*_seas_ were used for habitat overlap analysis.

### Relationship between hydrological habitat range and geographical range

To estimate species geographical range sizes, presence data were imported into QGIS (QGIS Development Team 2016) and convex hulls were fitted. The ellipsoidal area tool was then used to calculate geographical range sizes in km^2^. Range sizes were compared with ranges of bioclimatic indicator variables for all species, and separately for each functional type.

### Relationships between species richness and diversity in hydrological habitat position and range across genera

The ‘convhulln’ function from the R package ‘geometry’ was used to calculate the area of the smallest convex hull covering all species scores for each genus represented by three or more species in the PC1–PC2 space performed on the hydrological habitat position and range data sets for all species. This provided measures of the diversity of hydrological habitat position and range in each genus. Species richness values for each genus were obtained from the New Bromeliad Taxon List (Butcher and Gouda, cont. updated).

## Results

### Variation in hydrological habitat position and range between taxonomic groups

**Figure 2.**
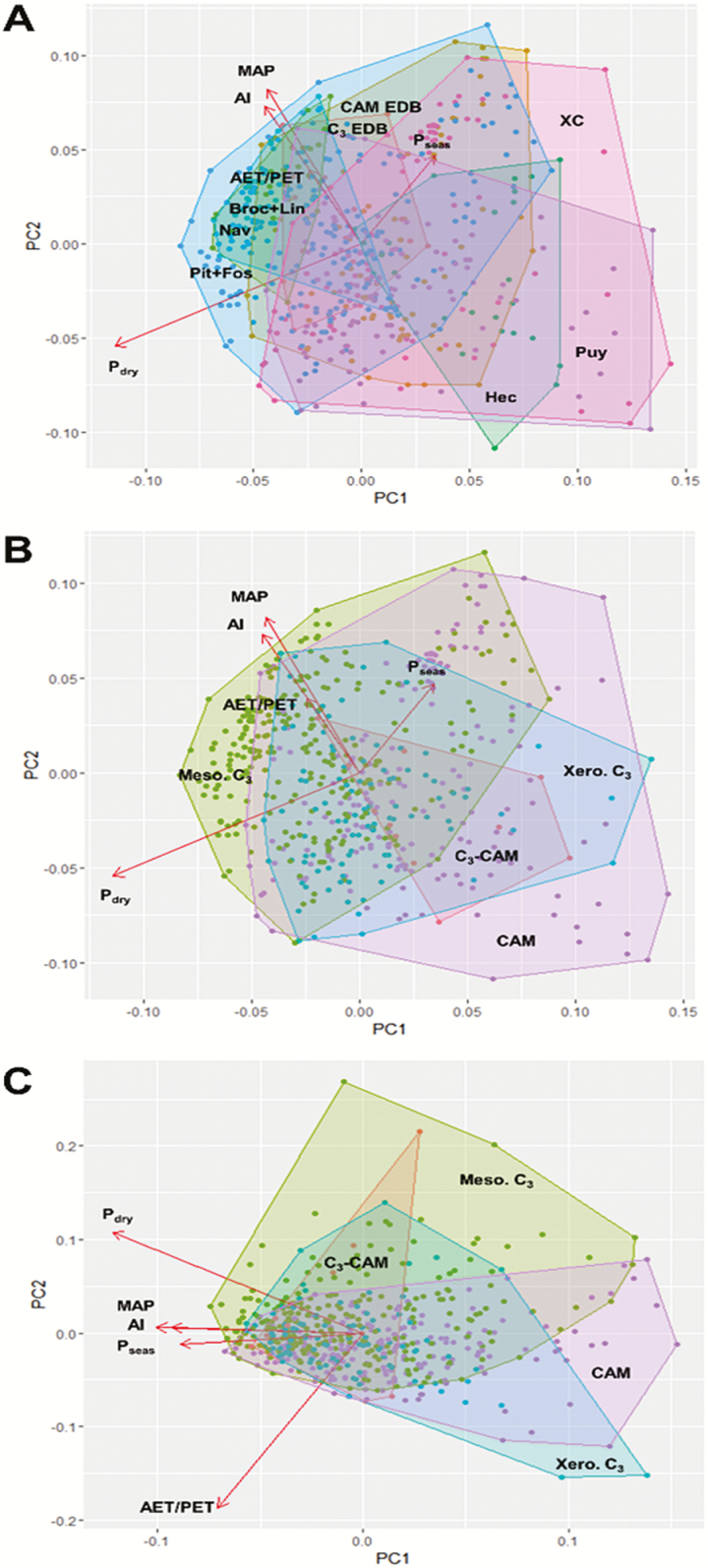
PC1–PC2 biplots for hydrological habitat occupancy properties of 564 terrestrial bromeliad species showing differentiation and overlap among taxonomic groups and functional types. Arrows show bioclimatic variable loadings. (A) PC1–PC2 biplot based on PCA of mean values of bioclimatic variables (MAP, AI, AET/PET, *P*_dry_, *P*_seas_). Species scores are plotted and grouped by taxonomic group, with separate convex hulls covering all species belonging to the following groups: Brocchinioideae and Lindmanioideae (Broc + Lin); Hechtioideae (Hec); Navioideae (Nav); *Pitcairnia* and *Fosterella* (Pit + Fos); Xeric Clade Pitcairnioideae (XC); Puyoideae (Puy); C_3_ early-diverging Bromelioideae genera (C_3_ EDB); and CAM early-diverging Bromelioideae genera (CAM EDB). (B) PC1–PC2 biplot based on PCA of mean values of bioclimatic variables (MAP, AI, AET/PET, *P*_dry_, *P*_seas_). Species scores are plotted and grouped by functional group, with separate convex hulls covering all species belonging to the following functional groups: C_3_ mesic terrestrial (Meso. C_3_); C_3_ succulent terrestrial (Xero. C_3_); C_3_-CAM succulent terrestrial; and CAM succulent terrestrial. (C) PC1–PC2 biplot based on PCA of ranges of bioclimatic variables (MAP, AI, AET/PET, *P*_dry_, *P*_seas_). Species scores are plotted and grouped by functional group, with separate convex hulls covering all species belonging to the following functional groups as in (B).

To test whether major taxonomic groups and functional types differed significantly in habitat position and range (Hypothesis 1), median values were compared across these groupings. Bioclimatic scores averaged across species within each genus showed a large amount of variation in median scores and ranges (for full data and interpretation of taxonomic and geographic coverage of GBIF distributional data **see Supporting Information—Tables S3 and S4 and Fig. S1**]. When PCA using bioclimatic data for all species was performed, PC1 and PC2 explained 77.6 and 12.2 % of the total variance in the data, respectively. The alignment of bioclimatic variable loadings reflected two major axes of variation corresponding to overall environmental moisture (MAP, AI, AET/PET) and precipitation seasonality (*P*_seas_, *P*_dry_). Taxonomic groups showed extensive overlap, but there were clear patterns (Fig. 2A).

The early-diverging Brocchinioideae and Lindmanioideae were restricted to the area of climate space associated with relatively high overall moisture and low/moderate precipitation seasonality. Species of the genus *Hechtia* (Hechtioideae) were located exclusively in moderately/highly seasonal, low/moderate moisture environments. Navioideae were principally clustered in the same area of climate space as Brocchinioideae and Lindmandioideae, with one important outlier being the species *Cottendorfia florida*, which was associated with more seasonal, lower-moisture environments. Species in the two early-diverging genera of the Pitcairnioideae (*Pitcairnia* and *Fosterella*) occupied a wide area of climate space, but did not occur in areas characterized by very low overall moisture. These areas were however occupied by Xeric Clade Pitcairnioideae, which covered a similarly broad region of climate space to that occupied by *Pitcairnia* and *Fosterella*. Although there was some overlap with the latter, the region occupied by the Xeric Clade was shifted towards the drier end of the overall moisture axis. *Puya* spp. (Puyoideae) covered a roughly comparable area of climate space to that occupied by Xeric Clade Pitcairnioideae, although they did not occur in very strongly seasonal environments. Among the terrestrial Bromelioideae, the basal C_3_ genera were confined to relatively high-moisture environments with moderate levels of seasonality, while CAM genera occupied a wider range of climate space that included considerably more seasonal and arid environments. Further PCAs and more detailed description of variation within taxonomic groups are available **[see Supporting Information—Fig. S2]**.

When comparisons were made between functional types rather than taxonomic groups, there was extensive within-group variation in functional types, but some clear differences between groups could be discerned (Fig. 2B). C_3_ mesic terrestrials generally occurred in environments with higher overall moisture and lower seasonality. C_3_ and C_3_-CAM succulent terrestrials, while showing extensive overlap with C_3_ mesic terrestrials, also occurred in drier and more seasonal environments. Meanwhile, CAM succulent terrestrials showed the broadest ranging habitat occupancy in the climate space, occurring in all environments except those with the very highest overall moisture and lowest seasonality.

In PCA on hydrological habitat range data for all species, PC1 and PC2 explained 75.3 and 12.1 % of the total variance in the data, respectively. This analysis identified three independent axes of variation in the bioclimatic variables: (i) AET/PET; (ii) *P*_dry_; and (iii) MAP, AI and *P*_seas_ (Fig. 2C). The loadings for AET/PET and *P*_dry_ were orthogonal, with the loadings for the third, multifactorial axis located approximately midway between. Functional types showed a high degree of overlap at the centre of PC1–PC2 space, suggesting that relatively broad hydrological habitat ranges occur in all taxonomic groups and are associated with tolerance of variation in a range of bioclimatic factors. However, the loadings for species in different functional types radiated differentially into the areas of the PC1–PC2 space associated with narrow habitat ranges, in a manner suggestive of contrasting environmental drivers of hydrological habitat range among different functional types. Overall, more succulent species (C_3_ and CAM succulent terrestrials) appeared to be more prone to limitation in habitat range by *P*_dry_.

Quantification of the hydrological habitat properties of all species facilitated the identification of traits connected with occupancy of particular regions of habitat space (Hypothesis 2), as covered in the Discussion.

### Hydrological habitat overlap analysis

To test whether there was greater hydrological habitat overlap in taxonomic groups with more specialized biotic interactions (Hypothesis 3), habitat overlap analysis was performed using the univariate indicators AI and *P*_seas_. The results are displayed in Table 3. In terms of AI, the lowest levels of univariate habitat overlap occurred in the C_3_ early-diverging Bromelioideae and the Navioideae, while the highest levels occurred in the Xeric Clade Pitcairnioideae and the *Pitcairnia*–*Fosterella* grade. The ranking of taxonomic groups by univariate habitat overlap in terms of *P*_seas_ was slightly different, with the most noticeable contrast being the shift in the position of Navioideae to very low levels of overlap. Navioideae species therefore appear to be unusual in overlapping considerably in their AI ranges but not in *P*_seas_ ranges.

**Table 3.** Mean species-level range overlap for AI and precipitation seasonality (*P*_seas_) within terrestrial bromeliad taxonomic groups, showing absolute values and values normalized by mean species-level variable range.

Taxonomic group	AI (mm mm^−1^)			*P*_seas_ (%)		
	Overlap	Mean range	Overlap/mean	Overlap	Mean range	Overlap/mean
Brocchinioideae–Lindmanioideae (*n* = 25)	403.49	5911.66	0.068	1.960	21.86	0.090
Hechtioideae (*n* = 26)	106.34	5026.96	0.021	1.800	18.40	0.098
Navioideae (*n* = 28)	826.78	4710.64	0.176	1.260	16.65	0.076
*Pitcairnia*–*Fosterella* (*n* = 208)	509.97	10187.66	0.050	4.210	32.94	0.128
Xeric Clade (*n* = 79)	554.83	3955.18	0.140	6.321	23.33	0.271
Puyoideae (*n* = 99)	499.21	7549.20	0.066	3.594	31.10	0.116
C_3_ early-diverging Bromelioideae (*n* = 22)	87.88	12449.85	0.007	1.083	36.54	0.030
CAM early-diverging Bromelioideae (*n* = 77)	252.93	5551.77	0.046	2.320	29.13	0.080

### Relationships between hydrological habitat position and range

To test whether species native to more arid habitats showed narrow hydrological habitat ranges (Hypothesis 4), linear regression analyses were performed between the species-specific positions and ranges for each bioclimatic variable across the whole data set and within taxonomic groups and functional types. Across the whole data set (*n* = 564), the only strong correlation between position and range for a bioclimatic variable was for *P*_dry_ (+ve, *r*^2^ = 0.40, *P* < 0.001). This relationship suggests that species adapted to low levels of precipitation during the driest part of the year are strongly specialized and restricted to such environments, whereas species adapted to higher levels of precipitation during the driest month of the year are more tolerant of a wider range of levels. Consistent with this contention, there were significant but much weaker correlations between mean and range for MAP (+ve, *r*^2^ = 0.06, *P* < 0.001) and for AI (+ve, *r*^2^ = 0.11, *P* < 0.001). In the case of AET/PET, there was a very weak negative correlation (*r*^2^ = 0.03, *P* < 0.001), with a steep decline in range occurring at the very highest mean values. The weakness of these correlations suggests that very different degrees of specialization can coexist under any given environment.

### Relationship between hydrological habitat range and geographical range

**Figure 3.**
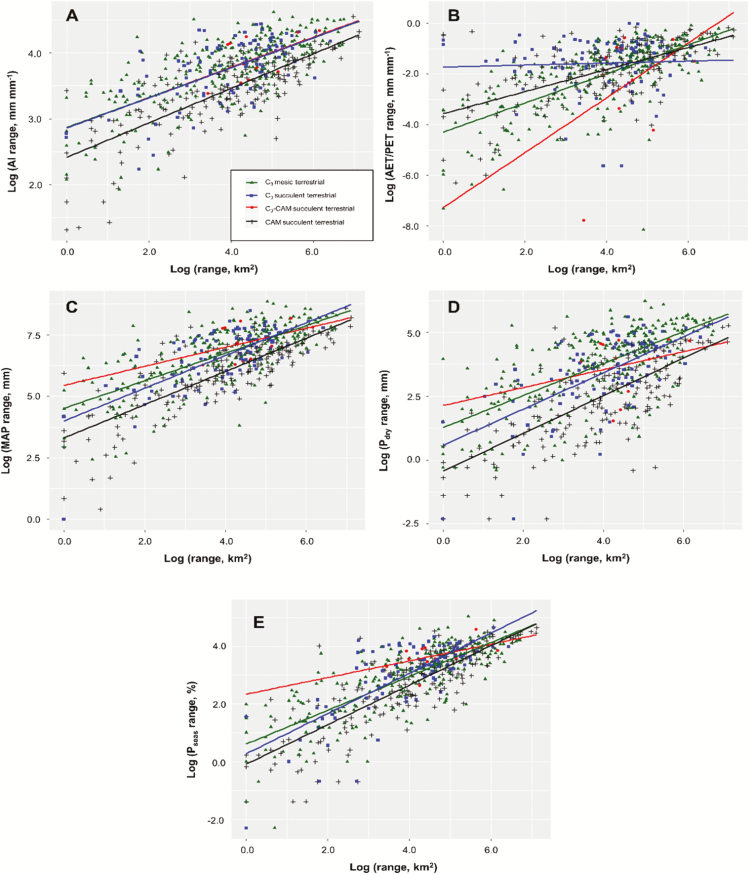
Strong positive relationships between log-transformed geographical range sizes (km^2^) and ranges of bioclimatic indicator variables: (A) AI; (B) AET/PET; (C) MAP; (D) *P*_dry_; (E) *P*_seas_. Lines show linear regression for each functional type (see legend in (A)).

To test for a link between hydrological habitat range and geographical range (Hypothesis 5), linear regression was performed between these metrics across all species (*n =* 564). This revealed strong positive correlations between log-transformed geographical range size and ranges for each bioclimatic indicator variable: AI (*r*^2^ = 0.48, *P* < 0.001), AET/PET (*r*^2^ = 0.31, *P* < 0.001), MAP (*r*^2^ = 0.52, *P* < 0.001), *P*_dry_ (*r*^2^ = 0.41, *P* < 0.001) and *P*_seas_ (*r*^2^ = 0.62, *P* < 0.001). These relationships are illustrated in Fig. 3. There was no significant difference in mean geographical range size between functional types (analysis of variance: *F* = 0.62, *P =* 0.600).

### Relationships between species richness and diversity in hydrological habitat position and range across genera

To test for a relationship between genus size and diversity in hydrological habitat position and range (Hypothesis 6), linear regression was performed between these metrics across 19 genera represented by three or more species in the bioclimatic data sets. This analysis identified a strong positive correlation between diversity in hydrological habitat position and range (*r*^2^ = 0.62, *P* < 0.001). Genera which showed greater diversity in hydrological habitat position therefore tended also to show greater variety in hydrological habitat range. Following log-transformation, there were additionally strong positive correlations between species richness and range in hydrological habitat position (*r*^2^ = 0.53, *P* < 0.001) and between species richness and range in hydrological habitat range (linear regression: *r*^2^ = 0.79, *P* < 0.001). Thus, larger genera tended to show significantly greater diversity with respect to both hydrological habitat position and range.

## Discussion

The analyses of biogeographical and bioclimatological patterns in the terrestrial bromeliads presented here provide timely clarification of critical questions relating to the ecophysiological diversity of a major plant radiation. These fresh insights not only help to improve our understanding of the evolutionary ecology of the important bromeliad family, but also represent significant contributions to the discourse surrounding core concepts in biogeographical patterns and processes in tropical herbaceous angiosperms.

### Taxonomic and geographic coverage of GBIF data

The presence data obtained from the GBIF portal and analysed in this investigation covered nearly half of all terrestrial bromeliad species (Butcher and Gouda, cont. updated), and included equal proportional representation of the species diversity of genera in all subfamilies with terrestrial elements. However, it is clear that some genera are better represented in GBIF data sets than others. For example, the genera *Fosterella* and *Greigia* are of comparable species diversity (31 spp. and 35 spp., respectively), but differed substantially in terms of the availability of sufficient, reliable presence data (80.6 % of *Fosterella* spp. and 51.4 % of *Greigia* spp.). The under-recording of specific taxa may be explained in part by biases in the geographic coverage of presence data, or may reflect the narrow endemism that is a feature of many bromeliad lineages (Benzing 2000). Low densities of presence points in regions such as the Amazon Basin may in part be indicative of lower sampling effort in these more remote regions. However, it is clear from the literature that low presence point densities in these regions are to some extent a reflection of genuinely lower species diversity and population densities (Givnish *et al.* 2011, 2014). Overall, it is difficult to determine what proportion of variation in apparent habitat ranges could be a by-product of bias in the number of presence points available for different species, which is an important caveat to the interpretations given here. This is a recognized limitation of GBIF data, and limited sampling effort may be especially characteristic of some of the remote and challenging Neotropical environments to which many bromeliads are native (Yesson *et al.* 2007; Beck *et al.* 2014). For rare species, the question of accurate representation of habitat occupancy by limited records is intensified by the recent observation (made using distributional data) that many rare Neotropical angiosperms are more geographically widely distributed than might be expected (Zizka *et al.* 2017).

The straightforward approach utilized here, which had the advantage of allowing extensive taxonomic sampling and no modelling-related assumptions, could in future be complemented by the elaboration of correlative species distribution models for representative species of each major taxonomic group and functional type for which sufficient presence data are available.

### Diversity and drivers of hydrological habitat position

Consistent with their broad geographic range and noted ecological diversity, the terrestrial bromeliads showed a wide variety of hydrological habitat positions. Despite extensive hydrological habitat overlap, there was evidence of differentiation among taxonomic and functional groups consistent with Hypothesis 1 (Table 1). Furthermore, in corroboration of Hypothesis 2 (Table 1), the observed variation can be related to existing knowledge of differences in species’ life-history, morphological and physiological traits. Understanding how functional traits underpin plant–environment interactions and thus define species’ environmental niches is crucial for efforts to predict the responses of species and communities to climate change (Violle and Jiang 2009).

Guiana Shield lineages (Brocchinioideae, Lindmanioideae, Navioideae) were almost universally constrained to a common area of high-moisture, low-seasonality climate space. These groups, which include some of the earliest-diverging bromeliads, lack innovations such as succulence, CAM or root xylem vessels that might have enhanced their capacity to invade other regions of climate space (Givnish *et al.* 2011, 2014; Males 2016). While *Connellia* spp. are able to survive in somewhat drier habitats than most Guiana Shield species, perhaps due to their reduced, stiff foliage, the only species to have escaped this narrow area of climate (and geographical) space is *C. florida*, a rhizomatous, drought-deciduous pyrophyte native to the Brazilian Cerrado. Heavy investment in subterranean storage structures and adaptive responses to seasonal stresses and fires enable this species to thrive under very different climatic regimes from those to which its closest relatives are adapted (Benzing 2000). It is also notable that among *Brocchinia* species, saxicolous tank-forming species such as *B. hechtioides* and *B. reducta* were associated with relatively low levels of moisture availability, where the ability to capture water in tanks could be advantageous (Givnish *et al.* 1997; Benzing 2000).

Perhaps the most comparable group to the Guiana Shield bromeliads is the *Pitcairnia–Fosterella* grade (Pitcairnioideae), which also comprises C_3_ mesic terrestrials, but is substantially more diverse in terms of hydrological habitat occupancy. This diversity could relate to the origin of root vessels in *Pitcairnia*, which presumably facilitate more efficient root-mediated water uptake (Tomlinson 1969; Males 2016), and the greater evolutionary lability in leaf form seen in *Pitcairnia* and *Fosterella*. It is perhaps significant that among *Pitcairnia* spp., many species occurring in high-moisture environments display broad, (pseudo-)petiolate leaf blades, whereas those from the driest and most seasonal environments frequently display highly reduced, sometimes spinose, linear leaf blades (Males 2017). Similarly, convergent cases of petiolate leaf morphology in the genera *Cryptanthus* and *Disteganthus* appeared to be associated with high levels of moisture, and the same is probably true for the strongly petiolate *Bromelia scarlatina*, for which sufficient distributional data were not available for analysis but which is closely related to the *B. tubulosa*, which was associated with higher moisture levels than any of its congeners. The same results would probably be found for rare petiolate epiphytes such as the endangered *Aechmea tayoensis* (IUCN 2016; cf. placement in *Ananas* in Sass and Specht 2010), which is restricted to high-rainfall regions of Ecuador, and *Ronnbergia morreniana* from Colombia and Ecuador. Cruz *et al.* (2017) note that multiple accessions for the petiolate species *Cryptanthus beuckeri* were not recovered as monophyletic in their phylogenetic analyses, suggesting that petiolate leaves may have arisen several times within that genus and underscoring the intriguingly recurrent nature of this trait in the bromeliads. Leaf shape is intimately associated with tank-mediated water trapping and foliar venation architecture, and therefore varies in tandem with leaf hydraulic properties and responses to water deficit (Males 2017). The more xeromorphic leaf morphoanatomy displayed by *Fosterella* spp. relative to most *Pitcairnia* spp. was not associated with any major difference in hydrological habitat position, although it is accepted that most *Fosterella* spp. tend to occupy very exposed microsites with free drainage (Wagner *et al.* 2013b). Even the two lowland Amazonian species are restricted to rocky bluffs and fluvial boulders (Wagner *et al.* 2013b). As in some *Pitcairnia* spp., endurance of extreme seasonality in *Fosterella* spp. is often associated with drought-induced deciduousness (Benzing 2000), which can hydraulically isolate the stem and root system from the atmosphere under severe evaporative demand. Deciduousness has been described in many *Pitcairnia* and *Fosterella* species native to seasonal habitats and rocky substrata, although more empirical research is needed to characterize the total phylogenetic distribution of this trait and the climatic thresholds and physiological processes which underpin it.

Succulence and CAM evolved independently in the Hechtioideae, Xeric Clade Pitcairnioideae, Puyoideae and Bromelioideae (Givnish *et al.* 2011; Crayn *et al.* 2015; Males 2016), and each of these lineages extends into regions of climate space characterized by lower total moisture and stronger precipitation seasonality than is observed for any but the most xeromorphic of C_3_ or C_3_-CAM species. This lends strong quantitative support to the notion that origins of CAM have allowed different bromeliad lineages to adapt to continuously or seasonally water-limited environments (Griffiths and Smith 1983; Smith *et al.* 1986; Martin 1994; Benzing 2000; Males 2016). CAM species with hydrological habitat positions corresponding to particularly arid conditions were typically thick- and narrow-leaved (e.g. *Deinacanthon urbanianum*, *Neoglaziovia variegata*). However, miniaturization was also associated with environmental extremity in *Deuterocohnia*, which is perhaps analogous to the combination of neoteny and xeromorphy that occurs in the atmospheric epiphytes of the genus *Tillandsia*. Meanwhile, CAM species occurring in less arid conditions were sometimes relatively thin-leaved (e.g. *Hechtia lundelliorum* and *H. tillandsioides*). In the case of *Hechtia*, new phylogenetic analyses are needed to determine if thin-leaved species are early-diverging within the genus and are representative of the intermediate form between a C_3_ mesic terrestrial ancestor and the highly xeromorphic succulent CAM species of the crown radiation of *Hechtia*. It is interesting to note that in the Crayn *et al.* (2015) carbon isotope ratio data set, while still clearly strong CAM plants, *H. lundelliorum* and the morphologically similar *H. caerulea* display two of the most negative values of all *Hechtia* species (−15.2 and −15.8 ‰, respectively).

In genera with both C_3_ and CAM (and sometimes C_3_-CAM) species, there was limited evidence for hydrological habitat differentiation between species of contrasting photosynthetic pathways. This was perhaps true of *Cryptanthus*, where the only species for which CAM appears to be absent, *C. schwackeanus* (Crayn *et al.* 2015), showed higher mean values for total moisture variables than definite CAM species. In *Puya*, C_3_ species generally occupied the area of climate space associated with higher moisture and lower seasonality, with the regions occupied by C_3_-CAM and CAM species being broadly congruent and shifted further towards lower moisture and higher seasonality.

Some C_3_ succulent terrestrial bromeliads occur well into the temperate zone of South America, with *Fascicularia bicolor* and *Ochagavia* spp. recorded as far south as Chiloé (Zizka *et al.* 2009). Adaptation to subtropical precipitation (and temperature) regimes explains why several of these species (e.g. *F. bicolor*, *O. carnea*) have become naturalized and even invasive at high latitudes in north-west Europe (Nelson and Zizka 1997; Morais *et al.* 2017). The success of *Ochagavia* species in considerably more seasonal environments than their close relative *F. bicolor* could be explained by the development of more extensive hydrenchyma in *Ochagavia* species, especially when compared with *F. bicolor* ssp. *canaliculata*, which is largely restricted to Valdivian temperate rainforest (Zizka *et al.* 2009). Other *Ochagavia* species for which sufficient distributional data were not available, *O. andina* and *O. elegans*, occur as saxicoles in drier Andean habitats and on the exposed coastal cliffs of the Juan Fernández Islands, respectively; both situations where high hydraulic capacitance could be particularly advantageous (Zizka *et al.* 2009). The extent to which such considerations might apply to *Greiga* and *Puya* spp. is unclear, since availability of comparative morphological, anatomical and ecophysiological data for these plants is currently very limited.

Variation in hydrological habitat position for *Orthophytum* species was consistent with the suggestion of Louzada *et al.* (2010) that species in the derived polyploid lineage occur in more xeric environments than earlier-diverging diploid *Orthophytum* species (for full results **see Supporting Information—Table S1**). Polyploidization can have dramatic effects on plant–environment interactions (Levin 1983; Baker *et al.* 2017; Donkpegan *et al.* 2017), and Paule *et al.* (2017) have recently demonstrated that its occurrence in *Fosterella* was associated with a shift in temperature niche. It is possible that changes in ploidy could also impact on plant water relations via cell size effects, thereby altering hydrological niche position and/or width (Males 2016). It is notable that polyploidy also occurs in other terrestrial bromeliads with both extreme hydrological habitat positions, such as the Xeric Clade Pitcairnioideae, and very wide hydrological habitat ranges, including the early-diverging Bromelioideae (Gitaí *et al.* 2014). In future, complete taxon sampling for concurrent ploidy analysis and phylogenetic estimation could provide further important insights in this area.

Some of the unexplained interspecific variation in hydrological habitat position could relate to differences in germination requirement and seedling ecophysiology. Müller *et al.* (2016) describe germination as a key ‘bottleneck’ in the determination of species’ distributions. The literature on bromeliad germination biology is quite extensive relative to pollination and dispersal, and seedling mortality in bromeliads has been studied quite intensively in the epiphytic Tillandsioideae subfamily (particularly the genera *Tillandsia* and *Vriesea*; Hietz *et al.* 2002; Winkler *et al.* 2005; Bader *et al.* 2009; Montes-Recinas *et al.* 2012; Toledo-Aceves *et al.* 2012). The applicability of the results from these groups to terrestrial species is not clear, but the general consensus arising from work on bromeliad regeneration niches is that they are strongly influenced by climatic conditions (Winkler *et al.* 2007; Wagner *et al.* 2013a). More research is needed to determine the extent to which the sensitivities of the earliest stages of plant development impact on bromeliad distributions and habitat occupancy.

### Diversity and drivers of hydrological habitat range

Hydrological habitat range varied extensively among the terrestrial bromeliad species considered here. Variation in the width of hydrological habitat ranges of C_3_ mesic terrestrials tended to align more closely with loadings for variables related to overall moisture, indicating the pre-eminent importance of adequate (often high) water supply for these species. The apparently greater importance of *P*_dry_ in limiting hydrological habitat range in C_3_ and CAM succulent terrestrial bromeliads is consistent with the hypothesis that succulent plants tend to occur within relatively narrow ranges of dry-season precipitation (Ellenberg 1981; Ogburn and Edwards 2010; Males 2017).

Particularly broad hydrological habitat ranges occurred in certain taxonomic groups. This was true of *Ananas* spp., for which a broad hydrological niche could partly explain why the pineapple, *Ananas comosus*, can be grown successfully in so many tropical and subtropical regions of the world (Bartholomew *et al.* 2002). Broad hydrological habitat range in species of other early-diverging CAM Bromelioideae (e.g. *Bromelia* spp., *N. variegata*) provides good evidence of the flexibility of CAM and the physiological advantages it confers under a wide range of environmental conditions (Lüttge 2010). However, C_3_-CAM species in the genus *Puya* tend to display greater hydrological habitat range than strictly C_3_ or CAM species, suggesting that the intermediate phenotype offers enhanced ecophysiological flexibility (Herrera *et al.* 2010; Quezada *et al.* 2014). In several genera, some of the broadest hydrological habitat ranges occurred in miniaturized species (e.g. *Deuterocohnia strobilifera*, *Lindmania subsimplex*, *Navia duidae*). There are various possible explanations for this observation. Small plant size could be associated with enhanced environmental tolerances in some cases (e.g. due to reduced surface area:volume ratio), but could also increase the importance of microclimatic factors that may not be well represented in the bioclimatic data sets used here. Other aspects of organismal biology not directly related to water use could be of relevance to the evolutionary context. For example, if propagule size scales with plant size, miniaturized species might be more effective at long-distance anemochorous dispersal, which could give them more frequent opportunities to invade regions with contrasting climatic conditions.

Other specialized growth forms occur in the terrestrial bromeliads, notably the tank growth form in *Brocchinia* spp. including *B. hechtioides* and *B. tatei*. Both of these species showed broader habitat ranges than their congeners, presumably due to the provision of external hydraulic capacitance and nutrient acquisition strategies by the tank (Givnish *et al.* 1997; Benzing 2000; Males 2016). Meanwhile, one of the broadest hydrological habitat ranges in the genus *Pitcairnia* occurred in *P. heterophylla*, which combines both drought-deciduousness and a tuberous rhizome which may provide high hydraulic capacitance and carbohydrate reserves that would help the plant survive periods of environmental adversity (Benzing 2000). Other instances of apparently broad hydrological habitat ranges can be explained by polymorphism within species. For example, *F. bicolor* includes two subspecies (ssp. *bicolor* and ssp. *canaliculata*) that differ in their investment in hydrenchyma and occupy distinct ecological zones. Each subspecies may in fact be relatively narrowly specialized, making this a promising system in which to study the structural-functional basis of environmental adaptation.

Narrow hydrological habitat ranges appeared to be driven by specificity to particular ranges of values for different bioclimatic variables in a species-dependent manner. In some cases, variables related to total moisture (MAP, AI, AET/PET) appeared to be more limiting, while in others factors related to precipitation seasonality (*P*_dry_, *P*_seas_) appeared more limiting. It was difficult to identify morphological factors that could explain this distinction or that were associated with narrow hydrological habitat range in general. However, some inferences could be drawn. Long, grass-like foliage in certain Brocchinioideae, Lindmanioideae and Navioideae was associated with narrow hydrological habitat range, perhaps because of the potential for high rates of water loss and hydraulic dysfunction in this high-conductance, low-capacitance arrangement. Similarly, long, thin, strap-like leaves in *Greigia alborosea* could limit its internal water-storage capacity and restrict it to high-moisture environments in its native Venezuela (Morillo *et al.* 2009). Interestingly, several of the *Orthophytum* species with narrow habitat range were caulescent rather than rosette-forming, suggesting that the caulescent growth form could be associated with greater environmental specialization.

Morphologically convergent succulent xerophytes (e.g. Xeric Clade Pitcairnioideae, *Puya* spp.) showed a considerable amount of variation in both hydrological habitat position and range in spite of their apparent structural similarity. While cryptic variation in internal anatomy could confer contrasting physiological characteristics and thereby promote ecological diversity among these groups, it seems likely that to a large extent the apparent segregation of environmental niches among these plants is the product of dispersal limitation or biotic interactions leading to spatial structuring.

**Figure 4.**
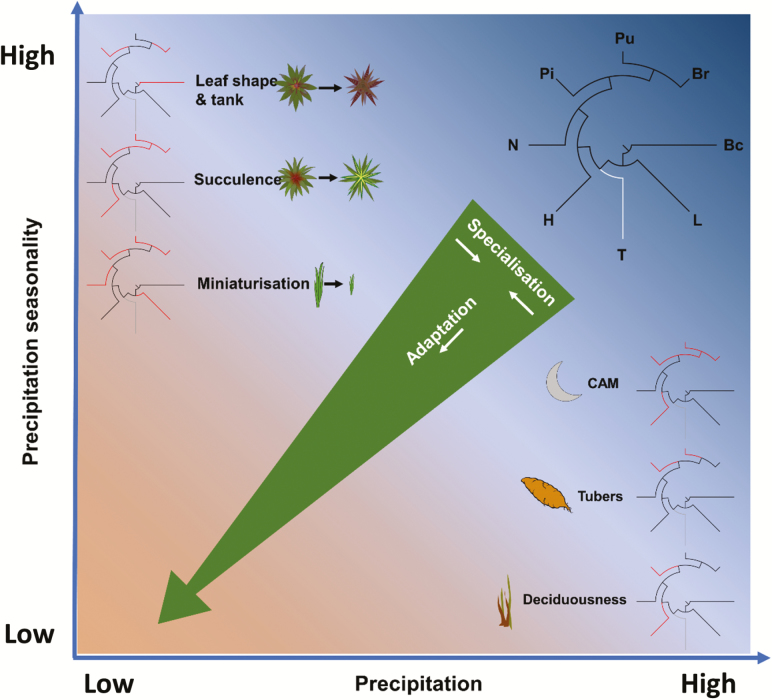
Major traits associated with adaptation to more arid and/or seasonal habitats and stronger environmental specialization (narrower habitat ranges). Reference cladogram (top-right) shows distribution of subfamilies: Bc = Brocchinioideae; L = Lindmanioideae; T = Tillandsioideae (not represented in this investigation); H = Hechtioideae; N = Navioideae; Pi = Pitcairnioideae; Pu = Puyoideae; Br = Bromelioideae. Red lines on cladograms adjacent to trait labels denote occurrence of the trait within a clade (NB not all members of the clade necessarily display the trait). For simplicity, only transitions towards occupancy of more arid and/or seasonal habitats and increasing environmental specialization are depicted.

As with hydrological habitat position, variation in habitat range is clearly strongly influenced by plant traits. Several traits that have evolved convergently in different bromeliad lineages appear to be frequently associated with a shift in habitat position towards more arid, seasonal environments, and/or increased environmental specialization (i.e. narrower habitat range). These include succulence, CAM and deciduousness (Fig. 4). Convergent evolution of traits such as petiolate leaf morphology can likewise be linked with invasion of more humid, aseasonal habitats. Instances of unique (rather than convergent) innovations of bioclimatic relevance are less easily identifiable, but include the origin of root xylem vessels in the genus *Pitcairnia*. The combination of convergent and divergent trait evolution has been shown to have been important in shaping the evolution of climatic niches in other plant groups (e.g. Evans *et al.* 2009), and is consistent with a complex mixture of adaptive constraints and opportunities (Losos 2011).

Of potentially profound significance for niche width and therefore for habitat range are intraspecific phenotypic variation and the capacity for phenotypic plasticity (Sultan 2001; González and Gianoli 2004; Miner *et al.* 2005; Sides *et al.* 2014; cf. Valladares *et al.* 2007). Neither of these phenomena are well-characterized in the bromeliads, and require further investigation.

### Variation in hydrological habitat overlap

The extent of univariate hydrological habitat overlap in terms of AI and *P*_seas_ was found to vary considerably between taxonomic groups, even when controlling for diversity in mean species-level bioclimatic ranges. Extensive hydrological habitat overlap occurs in the Xeric Clade Pitcairnioideae, which, when considered alongside their relatively uniform vegetative morphoanatomy and life history, suggests that pronounced species-level hydrological niche segregation is not a feature of this group. This supports Hypothesis 3 (Table 1), since there is some evidence for pollinator specificity in this clade (e.g. Machado and Lopes 2004; Christianini *et al.* 2013). By contrast, in lineages such as the C_3_ early-diverging Bromelioideae genera and the Hechtioideae, where there is less extensive overlap and much greater vegetative diversity, segregation in hydrological niche may have been more important in the generation of species diversity. That abiotic habitat specialization should be stronger in these higher-latitude groups is consistent with the concept that the relative importance of abiotic to biotic niche segregation as a driver of species diversity increases further from the equator (Hulshof *et al.* 2013). The Navioideae represent an interesting case, since they showed very different levels of overlap depending on which bioclimatic variable (AI or *P*_seas_) was considered. The comparatively low levels of habitat overlap in *P*_seas_ suggest that species diversification in Navioideae may have been contingent on adaptation to contrasting levels of *P*_seas_ while overall environmental moisture requirements have remained evolutionarily conserved.

### Relationships between hydrological habitat position and range

The only hydrological habitat indicator variable for which there was a strong, consistent relationship between species’ positions and ranges was *P*_dry_. This correlation supported Hypothesis 4 (Table 1) and with evidence from other plant groups (Thuiller *et al.* 2004; Carboni *et al.* 2016). It suggests that adaptation to lower precipitation levels during the driest part of the year involves greater environmental specialization, perhaps because particular structural or physiological traits associated with water scavenging or retention are optimally operative under particular sets of conditions. Weaker correlations between mean and range for MAP and AI are consistent with this hypothesis, but the fact that the relationships are not stronger implies that species of contrasting levels of specialization can occur under the same conditions, with important implications for bromeliad evolution and community ecology. However, the weak negative relationship between mean and range for AET/PET for some groups was in all cases driven by a cluster of very low range values at the highest mean values. This suggests that species adapted to the lowest levels of moisture deficit are highly specialized and perhaps restricted to narrow geographical ranges. The relationship was particularly strong in Navioideae, where it could relate to the narrow endemism of certain species in high-rainfall environments on the Guiana Shield (Givnish *et al.* 2011).

### Relationship between hydrological habitat range and geographical range size

In corroboration of Hypothesis 5 (Table 1), hydrological habitat range was strongly correlated with species’ geographical range size across all functional types in the terrestrial bromeliads, as has been observed in other taxonomic groups (Morin and Chuine 2006; Essl *et al.* 2009; Slayter *et al.* 2013). This scaling relationship is probably driven strongly by environmental tolerance, but trade-offs between environmental specialization and dispersal ability could also be relevant (Jocque *et al.* 2010). For example, species that are strongly adapted in their vegetative structure and function to sites characterized by very low water availability may allocate fewer resources to seed dispersal mechanisms.

### Relevance to bromeliad ecological and species diversity

The strong correlation between diversity of hydrological habitat position and range across genera highlights the degree of coordination in the evolution of plant–environment interactions in the terrestrial Bromeliaceae. Those genera which have radiated into diverse areas of hydrological habitat space tend to include species with the broadest range of hydrological habitat ranges. The accumulation of high levels of ecological diversity therefore seems to depend on the admixture of both hydrological generalists and specialists. Studies of other groups of organisms have demonstrated similar results, with climate niche lability being a good predictor of clade diversity (Martínez-Cabrera *et al.* 2012; Schnitzler *et al.* 2012; Koch *et al.* 2017), whereas in other cases phylogenetic niche conservatism has been invoked (Wiens *et al.* 2010; Crisp and Cook 2012; Skeels and Cardillo 2017). Transitions in life history may also have impacted on the rate of niche evolution in lineages such as *Puya* (Smith and Beaulieu 2009; Jabaily and Sytsma 2013; Ogburn and Edwards 2015).

The robust positive correlations between species richness and the ranges of hydrological habitat positions and ranges across genera support Hypothesis 6 (Table 1) and the idea that ecological diversification associated with differentiation in hydrological niche has been an important factor in the generation of species diversity in the terrestrial bromeliads. Moreover, these relationships are tentatively consistent with radiation into distinct climatic niches. Convincing demonstration of the operation of adaptive radiation within individual taxonomic groups will be dependent on improved phylogenetic resolution, comprehensive ecophysiological characterization of relevant species and the identification of trait divergences that can be linked to bioclimatic differentiation (Ackerly *et al.* 2006; Givnish 2015).

A range of other factors have been shown to be involved in the generation and maintenance of bromeliad species diversity, and could constrain species’ habitat occupancy to a small subspace of the suitable habitat predicted by trait-based fundamental hydrological niches. Notable examples include dispersal barriers and limitations (Linares-Palomino and Kessler 2009; Jabaily and Sytsma 2013; Givnish *et al.* 2014), and specialization in biotic interactions and mating systems (Krömer *et al.* 2008; Matallana *et al.* 2010; Palma-Silva *et al.* 2011; Christianini *et al.* 2013; Givnish *et al.* 2014; cf. Piacentini and Varassin 2007; Wendt *et al.* 2008). Little is known about other potentially important contributing factors to the overall environmental niche, such as sensitivity to soil composition and topographically or vegetationally determined differences in light regimes (Benzing 2000). Likewise, interspecific competition and facilitation effects (with bromeliads and non-bromeliads) could curtail or extend habitat occupancy, but are little-studied (Miller and Silander 1991; Scarano 2002). The realized habitat occupancy of bromeliad species may also be limited by disturbance phenomena, including human activity, hurricanes and fire (Miller and Silander 1991; Benzing 2000). The corollary of this is that species with narrower niches and habitat ranges are likely to be more vulnerable to disturbance and global change (Thuiller *et al.* 2005; Broennimann *et al.* 2006). Despite the fact that the hydrological component of the species niche is only one piece of the jigsaw, when the evidence presented here is considered alongside the acknowledged proliferation in the Bromeliaceae of innovations associated with water-use strategies, it is clear that specialization and differentiation in the hydrological habitat occupancy has been a central theme in bromeliad evolution (Males 2016). Further examination of bromeliad hydrological habitat occupancy could in future incorporate consideration of variation in water availability at a range of temporal scales to cast further light on the relevance of temporal variability for species diversity (Chesson *et al.* 2004; Reineking *et al.* 2006; Schwinning and Sala 2004; Reyer *et al.* 2013).

## Conclusions

The terrestrial bromeliads provide a powerful system in which to test the applicability of fundamental biogeographical and ecological hypotheses to radiations of tropical herbaceous angiosperms, an important but understudied functional group. Across the terrestrial bromeliads, hydrological habitat position and range varies systematically between taxonomic groups and functional types. Convergent and divergent life-history, morphological and physiological traits impact on species’ hydrological niches and drive differences in hydrological habitat occupancy. Overlap in habitat occupancy may be greater in lineages with more strongly specialized biotic interactions, while environmental specialization is stronger in species native to more arid habitats. Terrestrial bromeliads’ geographical range sizes are closely linked with the range of hydrological habitats in which they occur. Differentiation in hydrological habitat occupancy has probably been a critical aspect of the generation and maintenance of high levels of species diversity in the bromeliads.

## Sources of Funding

UK Natural Environment Research Council award no. 1359020.

## Conflict of Interest

None declared.

## Supporting Information

The following additional information is available in the online version of this article—

**Figure S1.** Geographic distribution of presence data for 564 terrestrial bromeliad species analysed in this investigation, plotted by taxonomic group.

**Figure S2.** PCA on hydrological habitat position and range data within taxonomic groups.

**Table S1.** Geographical and bioclimatic properties of the distributions of the 564 terrestrial bromeliad species analysed in this investigation.

**Table S2.** Correlations between sample size and calculated ranges for bioclimatic variables across all sampled terrestrial bromeliads.

**Table S3.** Extent of taxon sampling for distributional and bioclimatic analyses across terrestrial bromeliad genera and subfamilies.

**Table s4.** Pairwise linear correlations between genus-mean positions for bioclimatic indices based on data for 564 terrestrial bromeliad species.

## Supplementary Material

Supplementry Information
