# Supplementary material for: Geography, environment and organismal traits in the diversification of a major tropical herbaceous angiosperm radiation
Source: AoB Plants. 2018 Jan 30;10(1):ply008. doi: 10.1093/aobpla/ply008 (PMC5814923; doi:10.1093/aobpla/ply008)
Supplement: Supplementry Information [file ply008_suppl_supplementry_information.docx]

**Supporting Information Figure S1**. Geographic distribution of presence data for 564 terrestrial bromeliad species analysed in this investigation, plotted by taxonomic group.


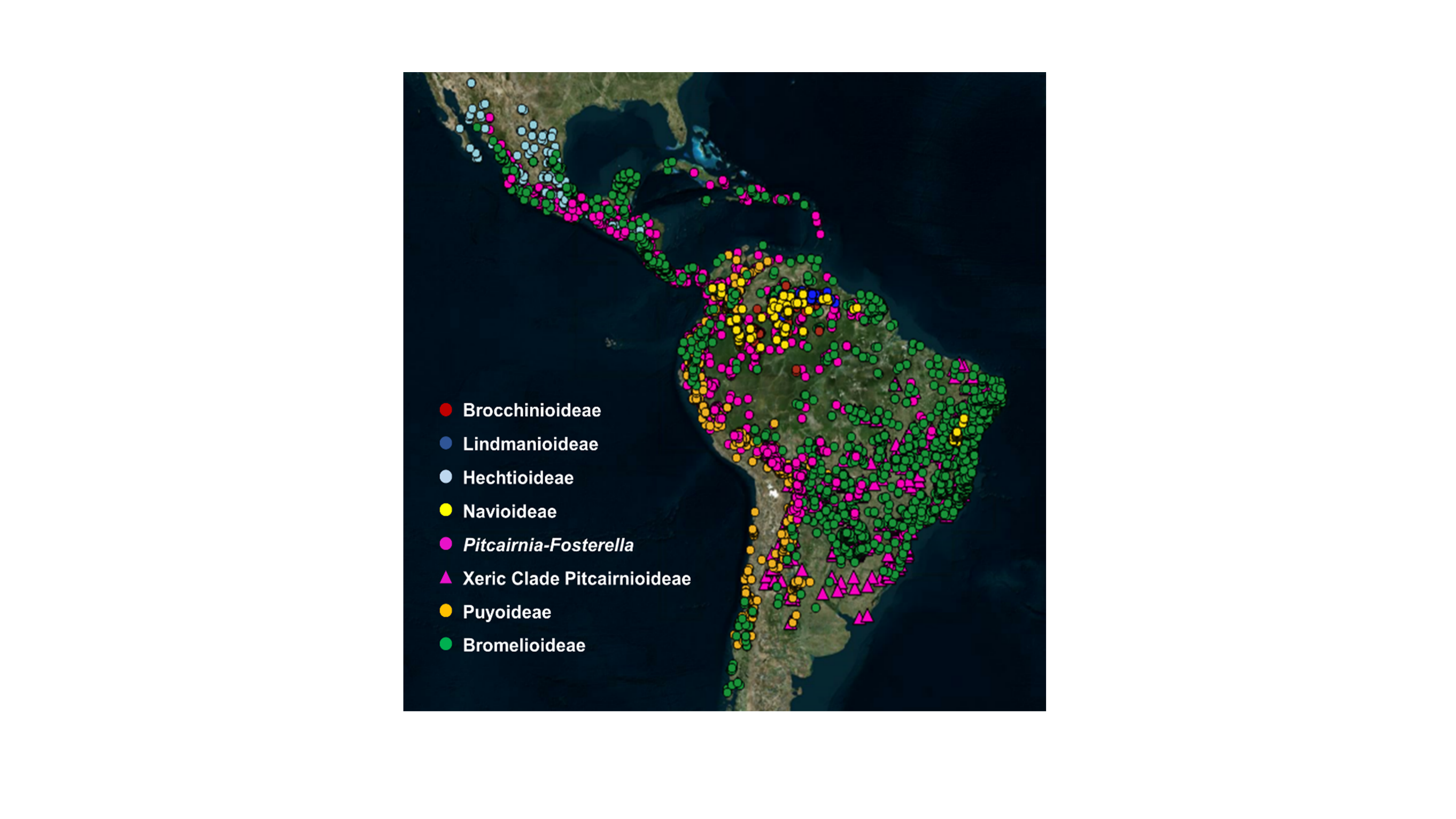


The geographic coverage of the data analysed in this investigation is displayed in Fig. S1. Variation in the density of presence data between different geographical regions was notable, with low densities in parts of Amazonia, the Caribbean islands, and the Central Andes. The effect of the number of presence points per species on univariate habitat ranges was also assessed across the entire dataset.

**Supporting Information Figure S2.** PCA on hydrological habitat position and range data within taxonomic groups.

PCA was performed separately on data for species within each major taxonomic group in order to identify more detailed, lineage-specific trends in variation in hydrological habitat position and range.

*Brocchinioideae and Lindmanioideae*

Position

PC1- 72.0%

PC2- 23.1%

Range

PC1- 67.0%

PC2- 15.4%

Among the Brocchinioideae and Lindmanioideae, the overall moisture vs. seasonality distinction was maintained, and the most distinctive hydrological habitat positions occurred in *Connellia* (Lindmanioideae), which clustered tightly in the area of the PC1-PC2 climate space characterised by lower overall moisture and moderate seasonality (Fig. S2.1). Scores for *Brocchinia* (Brocchinioideae) and *Lindmania* (Lindmanioideae) species covered wider habitat ranges, with the centre of the range for *Lindmania* spp. being located further towards the high-seasonality end of the climate space than for *Brocchinia* spp. The *Lindmania* species whose hydrological habitat position was associated with the highest level of seasonality was *L. thyrsoidea* L.B.Sm., while *L. nubigena* (L.B.Sm.) L.B.Sm. occupied the other end of this spectrum. In *Brocchinia*, a shorter gradient existed between *B. reducta* Baker and *B. delicatula* L.B.Sm., the latter being associated with the lowest levels of seasonality. Meanwhile the habitat position of *B. rupestris* (Gleason) Holst was associated with considerably lower overall moisture than any other species in the genus or in Lindmanioideae.

PCA on hydrological habitat range data for Brocchinioideae and Lindmanioideae identified two axes of variation corresponding to overall environmental moisture (MAP, AI, AET/PET) and seasonality (P_dry_, P_seas_; Fig. S2.2). In *Brocchinia*, the broadest habitat ranges occurred in *B. hechtioides* Mez and *B. tatei* L.B.Sm., two tank-forming species. *B. delicatula* showed narrow habitat range with respect to seasonality but not total moisture, while the reverse was true for *B. rupestris*. Among *Lindmania* species, broad habitat range occurred in species such as the strongly morphologically-reduced *L. subsimplex* L.B.Sm.. Narrow habitat range in terms of seasonality but not total moisture occurred in *L. thyrsoidea*, with the reverse being true for *L. nubigena*. Most *Connellia* species showed relatively broad habitat ranges, the exception being *C. caricifolia* L.B.Sm., which appeared to be strongly restricted by seasonality.


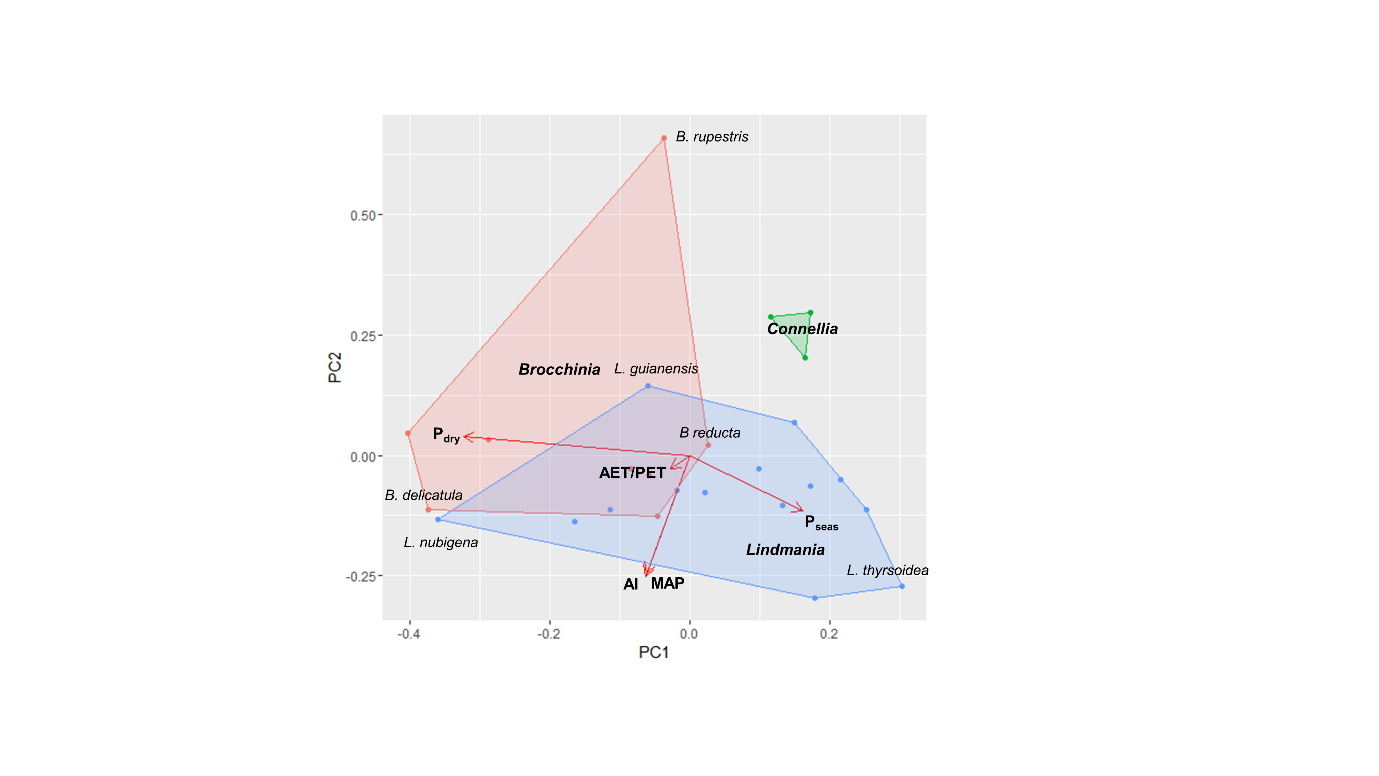


Figure S2.1. PC1-PC2 biplot based on PCA of mean values of bioclimatic variables (MAP, AI, AET/PET, P_dry_, P_seas_) for Brocchinioideae and Lindmanioideae (*n* = 35). Species scores are plotted and grouped by genus, with separate convex hulls covering all species belonging to each genus. PC1-PC2 scores for selected species are highlighted and arrows show bioclimatic variable loadings.


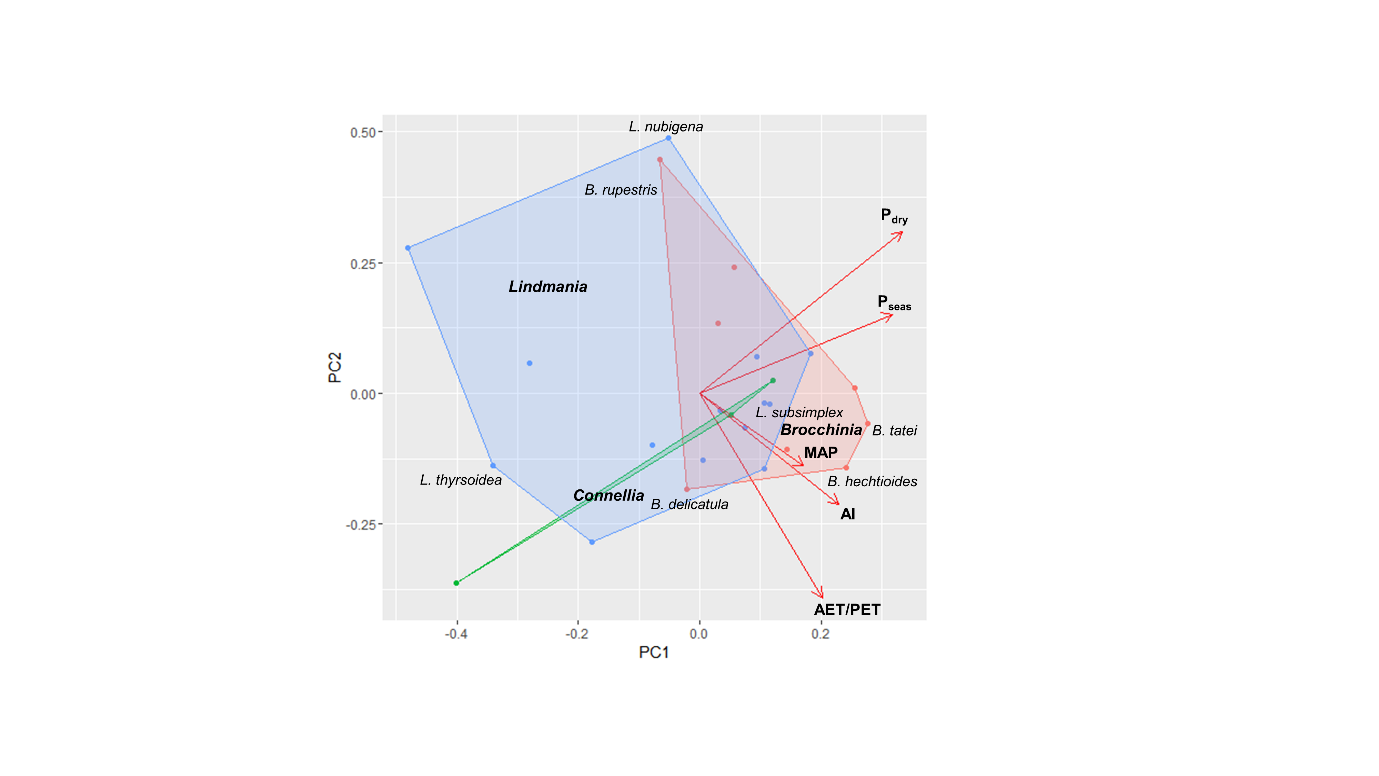


Figure S2.2. PC1-PC2 biplot based on PCA of ranges of bioclimatic variables (MAP, AI, AET/PET, P_dry_, P_seas_) for Brocchinioideae and Lindmanioideae (*n* = 35). Species scores are plotted and grouped by genus, with separate convex hulls covering all species belonging to each genus. PC1-PC2 scores for selected species are highlighted and arrows show bioclimatic variable loadings.

Hechtia *(Hechtioideae)*

Position

PC1- 74.4%

PC2- 23.9%

Range

PC1- 85.7%

PC2- 8.6%

In the Central-North American succulent CAM genus *Hechtia* (Hechtioideae) there was approximately equal variation across total moisture and seasonality axes (Fig. S2.3). Species such as *H. texensis* S.Watson occupied the area of climate space characterised by moderate seasonality and low overall moisture, while strongly seasonal environments with high overall moisture were associated with species including *H. laevis* L.B.Sm. The two species whose habitat positions were located in the region of climate space characterised by the highest moisture and lowest seasonality were *H. lundelliorum* L.B.Sm. and *H. tillandsioides* (André) L.B.Sm., both of which are relatively thin-leaved and lack prominent spines.

PCA of habitat range data for *Hechtia* identified two axes of variation corresponding to overall moisture (including P_dry_) and P_seas_ (Fig. S2.4). The broadest overall habitat range occurred in *H. glomerata* Zucc., while *H. stenopetala* Klotzsch showed higher habitat range in terms of overall moisture but not seasonality. *H. lyman-smithii* Burt-Utley & Utley appeared to be limited by seasonality more than by overall moisture, while the reverse was true of *H. liebmannii* Mez. *H. pedicellata* S.Watson appeared to be equally restricted by both factors.


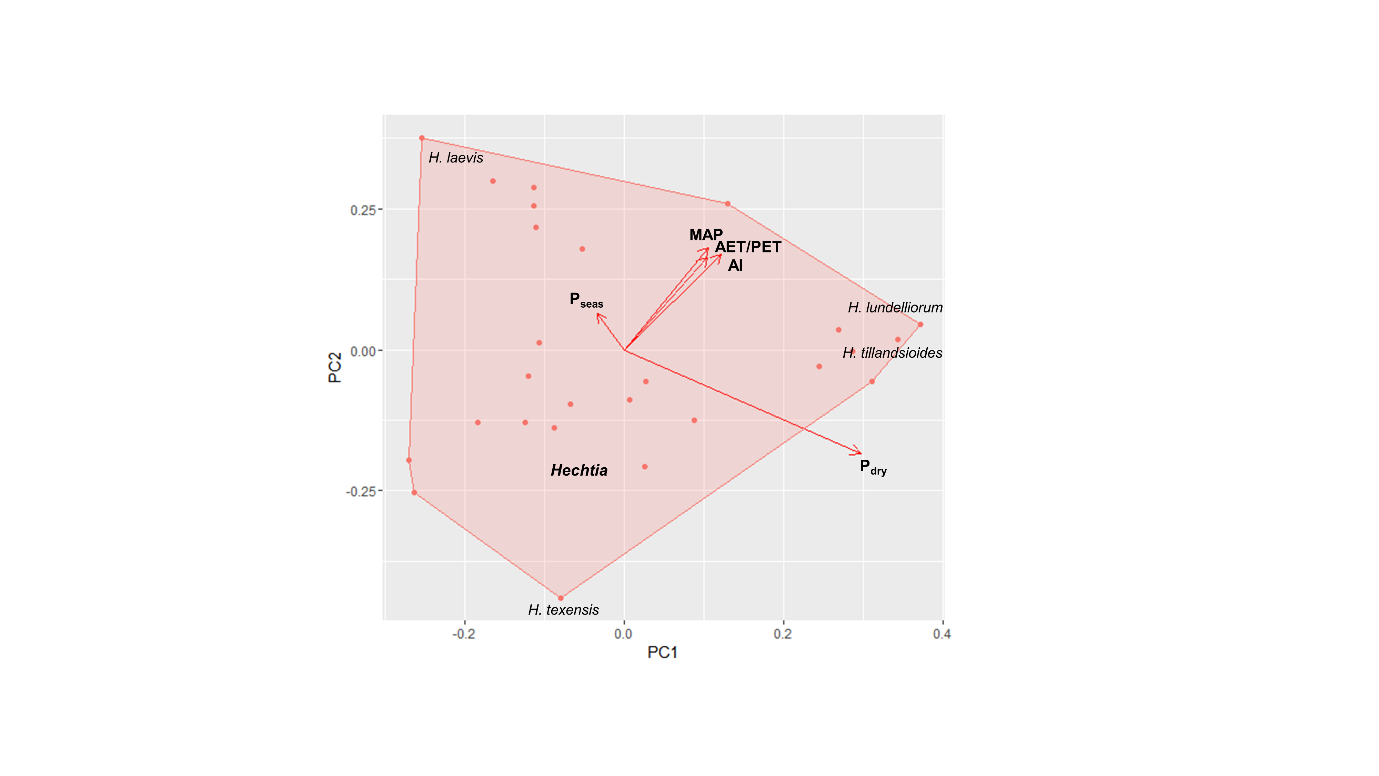


Figure S2.3. PC1-PC2 biplot based on PCA of mean values of bioclimatic variables (MAP, AI, AET/PET, P_dry_, P_seas_) for Hechtioideae (*n* = 26). Convex hull covers all species. PC1-PC2 scores for selected species are highlighted and arrows show bioclimatic variable loadings.


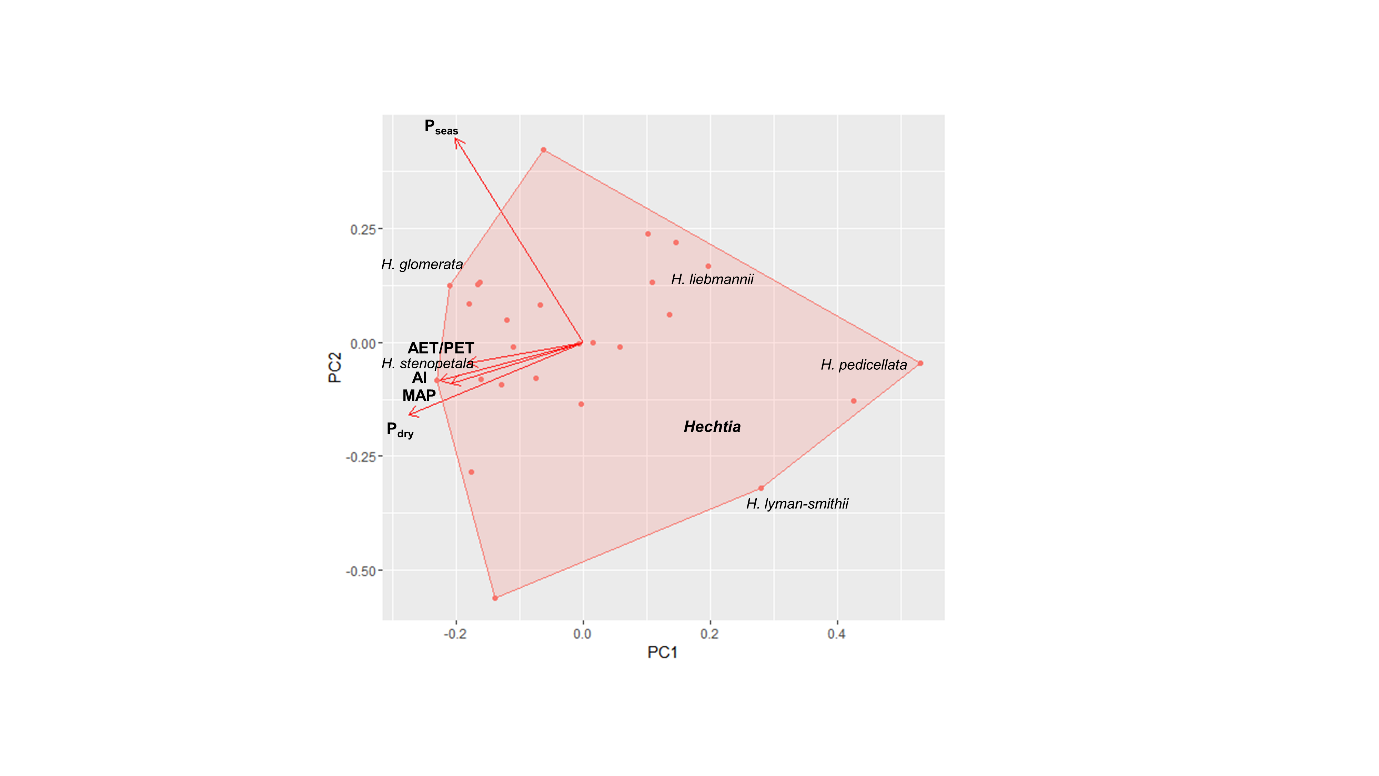


Figure S2.4. PC1-PC2 biplot based on PCA of ranges of bioclimatic variables (MAP, AI, AET/PET, P_dry_, P_seas_) for Hechtioideae (*n* = 26). Convex hull covers all species. PC1-PC2 scores for selected species are highlighted and arrows show bioclimatic variable loadings.

*Navioideae*

Position

PC1- 73.2%

PC2- 20.7%

Range

PC1- 63.9%

PC2- 20.8%

The results of PCA on hydrological habitat position data for the Navioideae were consistent with the overall moisture vs. seasonality distinction. In terms of variation in species’ scores, the strongest contrast was between the monospecific genus *Cottendorfia* and the remainder of the subfamily (Fig. S2.5). *C. florida* was located in the corner of the PC1-PC2 climate space associated with the lowest overall moisture and moderate seasonality. *Navia* spp. showed the widest range of habitat positions, and this variation was primarily parallel to the PC loading for seasonality, suggesting that overall moisture demand is relatively strongly conserved across the genus. Species associated with the highest levels of seasonality included *N. ovoidea* L.B.Sm., Steyerm. & H.Rob., while species such as *N. terramarae* L.B.Sm. & Steyerm. occupying the other end of the spectrum. The area of climate space occupied by *Brewcaria* spp. fell towards the centre of that occupied by *Navia* spp., and variation in habitat position in *Brewcaria* was again confined to the seasonality axis. While data were available for only three *Steyerbromelia* species, these seemed to conform to the same pattern, and their habitat positions were also located towards the centre of the range occupied by *Navia* spp. *Sequencia serrata* (L.B.Sm.) Givnish, the sole representative of its genus, was located towards the low-seasonality end of the area of climate space occupied by *Navia* spp. Overall, it appears that moisture requirement is relatively strongly conserved across the Navioideae, with the exception of *C. florida*, while there has been some degree of adaptation to different temporal distribution of precipitation.

When PCA was performed on habitat range data for Navioideae species, bioclimatic variable loadings fell onto three axes: 1) MAP and AI; 2) P_dry_ and P_seas_; 3) AET/PET (Fig. S2.6). The first and second of these axes were orthogonal, with the third lying approximately midway between. The broadest habitat ranges occurred in *Navia* spp., notably the diminutive *N. duidae* L.B.Sm. Narrow habitat range with respect to total moisture but not seasonality occurred in *N. luzuloides* L.B.Sm., Steyerm. & H.Rob., whereas the converse arrangement was true for *N. terramarae*. Habitat ranges of *Brewcaria*, *Cottendorfia* and *Steyerbromelia* species fell within the range of habitat ranges occupied by *Navia* spp., whereas *Sequencia serrata* displayed exceptional specificity with respect to overall moisture.


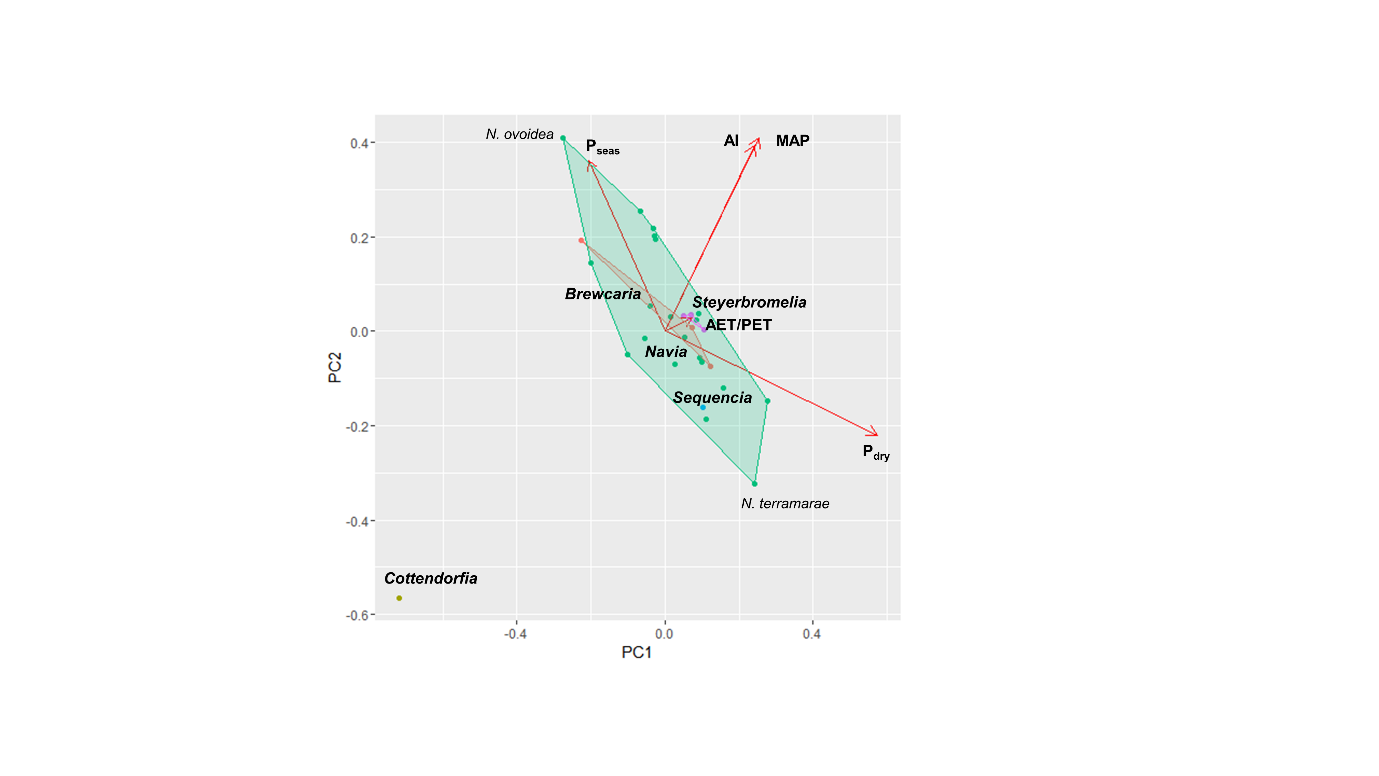


Figure S2.5. PC1-PC2 biplot based on PCA of mean values of bioclimatic variables (MAP, AI, AET/PET, P_dry_, P_seas_) for Navioideae (*n* = 28). Species scores are plotted and grouped by genus, with separate convex hulls covering all species belonging to each genus. PC1-PC2 scores for selected species are highlighted and arrows show bioclimatic variable loadings.


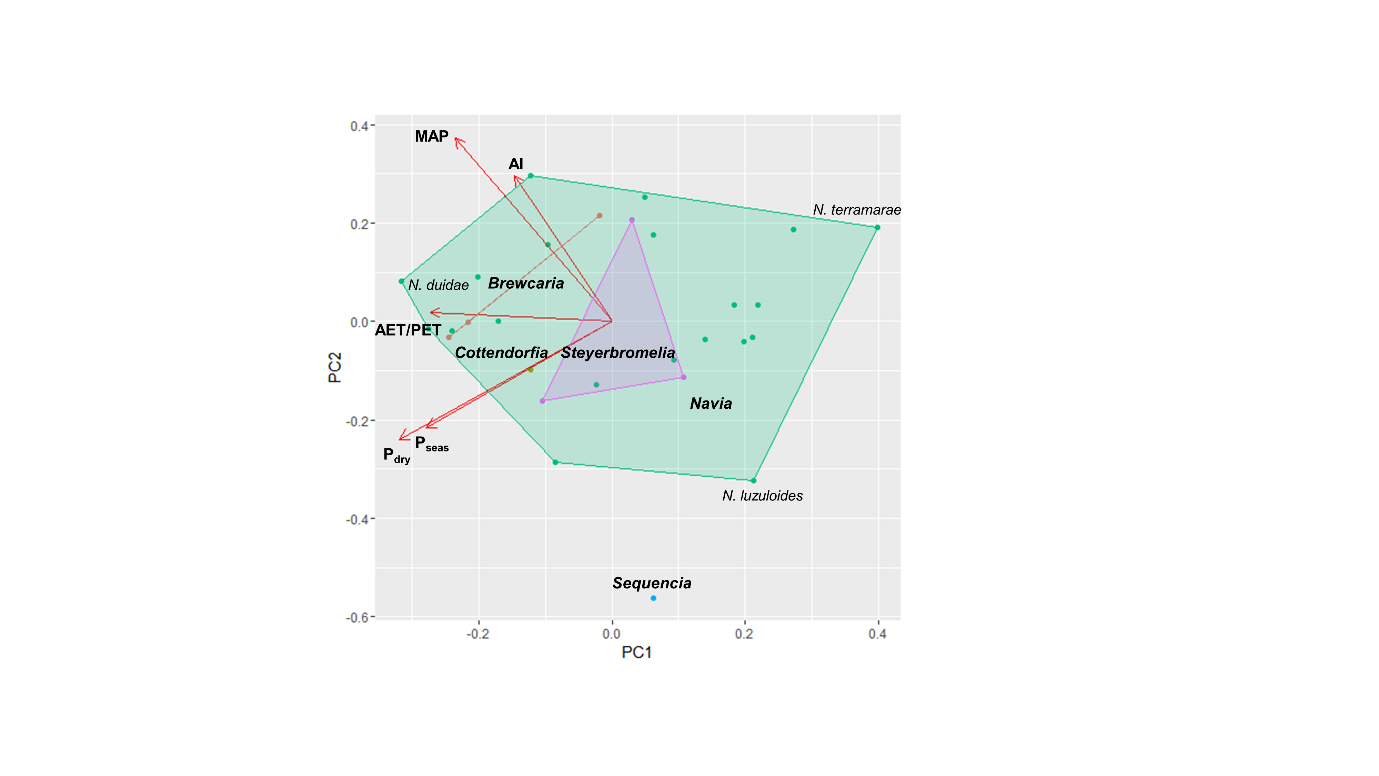


Figure S2.6. PC1-PC2 biplot based on PCA of ranges of bioclimatic variables (MAP, AI, AET/PET, P_dry_, P_seas_) for Navioideae (*n* = 28). Species scores are plotted and grouped by genus, with separate convex hulls covering all species belonging to each genus. PC1-PC2 scores for selected species are highlighted and arrows show bioclimatic variable loadings.

*Pitcairnia and Fosterella*

Position

PC1- 83.0%

PC2- 13.1%

Range

PC1- 79.4%

PC2- 11.4%

Hydrological habitat positions in the large genus *Pitcairnia* varied widely across the PC1-PC2 climate space, where the total moisture vs. precipitation seasonality distinction was supported (Fig. S2.7). Aseasonal, high-moisture environments were associated with broad-leaved, often petiolate species such as *P. archeri* L.B.Sm., while strongly seasonal environments with high overall moisture hosted species such as the largely coastal *P. calcicola* J.R.Grant & J.F.Morales. Seasonal low-moisture environments were associated with species with morphologically-reduced leaves, such as *P. micheliana* Andrews, while aseasonal low-moisture environments were occupied by species such as *P. nobilis* Mez & Sodiro. The range of habitat positions of *Fosterella* spp. fell within the core of the range occupied by *Pitcairnia* spp. *Fosterella* spp. showed slightly more dispersion along the overall moisture axis than the seasonality axis. The habitat position of the narrow-leaved South American lithophyte *F. albicans* (Griseb.) L.B.Sm. was located in the lowest-moisture and most seasonal corner of the climate space occupied by *Fosterella* spp. Seasonal, high-moisture environments were associated with the broad-leaved Central American species *F. micrantha* (Lindl.) L.B.Sm., while aseasonal, high-moisture environments were occupied by the lowland Amazonian species *F. pearcei* (Baker) L.B.Sm.

When PCA was performed on habitat range data for *Pitcairnia* and *Fosterella*, the bioclimatic variable loadings fell into four groupings: 1) AET/PET; 2) P_seas_; 3) MAP and AI; 4) P_dry_ (Fig. S2.8). *Pitcairnia* spp. such as *P. heterophylla* (Lindl.) Beer and *P. macranthera* André showed some of the broadest habitat ranges, while other species appeared to be differentially sensitive to different bioclimatic variables. *P. mituensis* L.B.Sm. displayed a particularly narrow habitat range with respect to AET/PET, whereas *P. condorensis* Manzan. & W.Till. appeared to be most strongly limited by P_seas_ and *P. prolifera* Rauh by P_dry_. Among *Fosterella* spp., the broadest habitat ranges occurred in species such as *F. pearcei*, while *F. caulescens* Rauh appeared to be limited by AET/PET and P_seas_, and *F. vasquezii* E.Gross & Ibisch by MAP/AI and P_dry_.


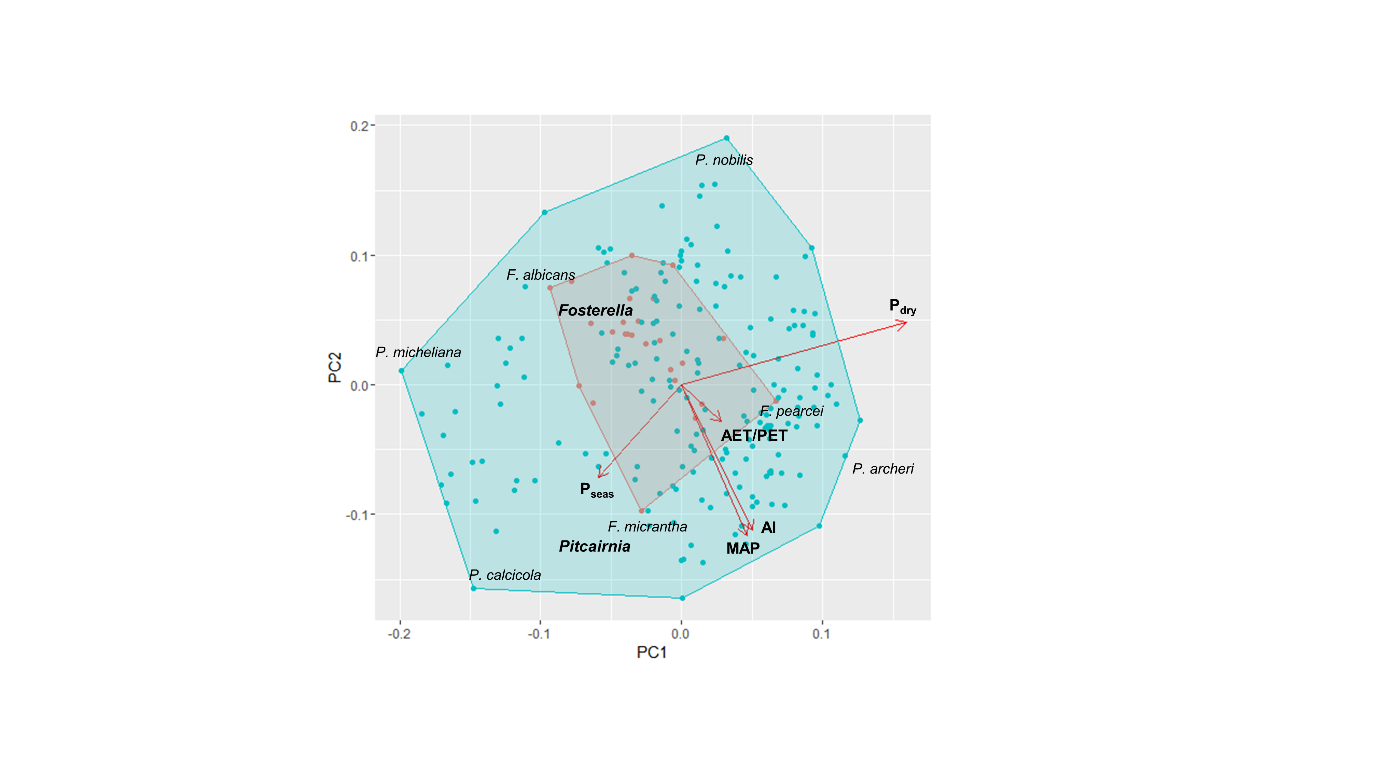


Figure S2.7. PC1-PC2 biplot based on PCA of mean values of bioclimatic variables (MAP, AI, AET/PET, P_dry_, P_seas_) for *Pitcairnia* and *Fosterella* (*n* = 208). Species scores are plotted and grouped by genus, with separate convex hulls covering all species belonging to each genus. PC1-PC2 scores for selected species are highlighted and arrows show bioclimatic variable loadings.


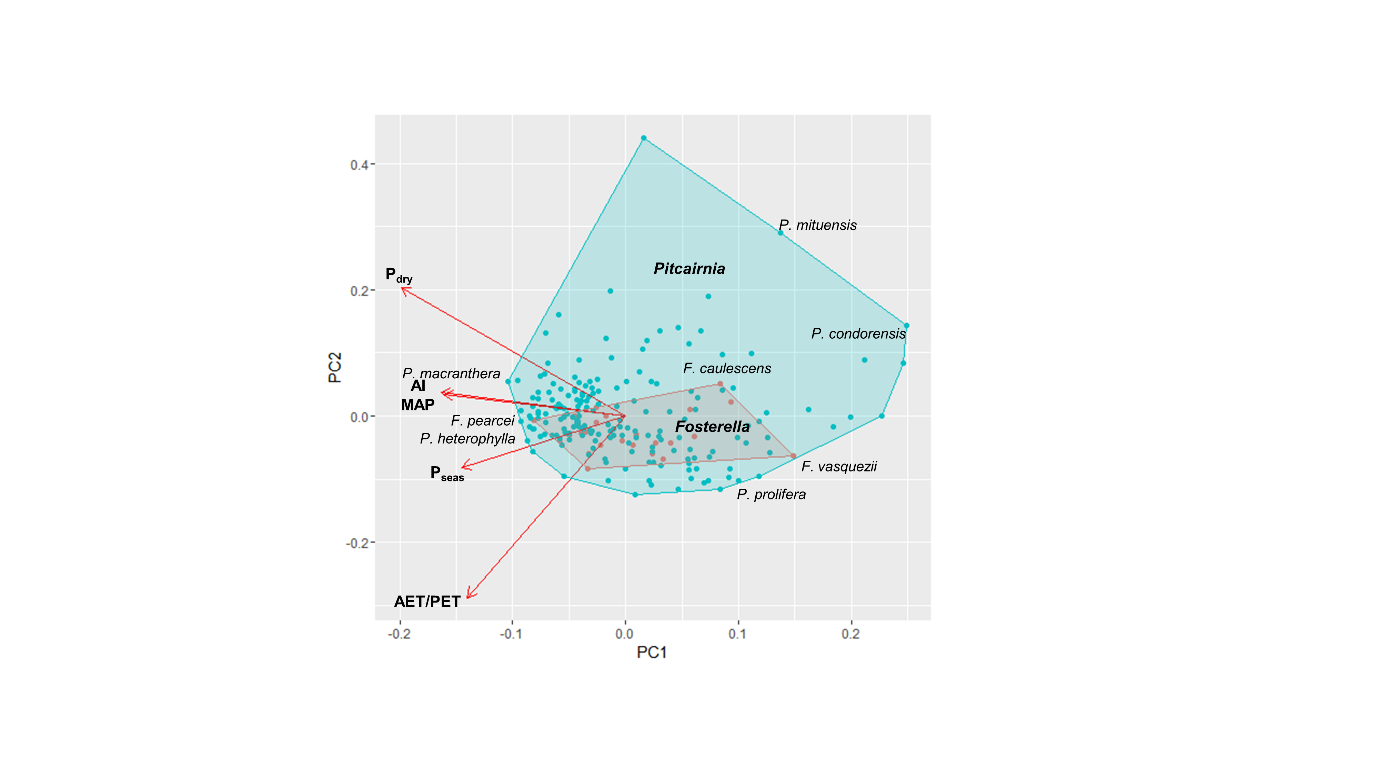


Figure S2.8. PC1-PC2 biplot based on PCA of ranges of bioclimatic variables (MAP, AI, AET/PET, P_dry_, P_seas_) for *Pitcairnia* and *Fosterella* (*n* = 208). Species scores are plotted and grouped by genus, with separate convex hulls covering all species belonging to each genus. PC1-PC2 scores for selected species are highlighted and arrows show bioclimatic variable loadings.

*Xeric Clade Pitcairnioideae*

Position

PC1- 76.4%

PC2- 19.9%

Range

PC1- 84.4%

PC2- 6.9%

For the Xeric Clade Pitcairnioideae, the total moisture vs. precipitation seasonality distinction in bioclimatic variable loadings held true (Fig. S2.9). Species’ scores for the three genera overlapped towards the centre of the PC1-PC2 climate space, but each genus also occupied more distinctive regions of the space. *Deuterocohnia* spp. occurred in the lowest moisture environments with moderate levels of seasonality. Species associated with the very driest regions included the miniaturised *De. haumanii* A.Cast. and *De. lorentziana* (Mez.) M.A.Spencer & L.B.Sm. Species with habitat positions located in slightly higher-moisture and less seasonal areas of the climate space included *De. meziana* Kuntze ex Mez. *Dyckia* is the largest of the Xeric Clade genera and showed the widest range of hydrological habitat positions. Two major loose clusters were identifiable: both towards the high-moisture end of the climate space, but towards opposite ends of the seasonality spectrum, reflecting the geographical distribution of *Dyckia* diversity in central and southern Brazil. From the first of these groups, the relatively large and broad-leaved *Dy. maritima* Baker was associated with the lowest levels of seasonality, while from the second group the highly spinose species *Dy. dawsonii* L.B.Sm. occurred in environments with the highest seasonality and highest overall moisture. One exceptional *Dyckia* species, *Dy. velascana* Mez, occurred in an area of habitat space comparable to those occupied by *Deuterocohnia* spp. Finally, in the genus *Encholirium* most variation was along the seasonality axis, with a lesser amount of variation in overall moisture. The species *E. spectabile* Mart. ex Schult. & Schult.f. was associated with the driest and most aseasonal environments, while *E. longiflorum* Leme occupied the area of habitat space characterised by the most extreme seasonality but somewhat higher overall moisture.

Three groupings of bioclimatic variable loadings were identified when PCA was performed on habitat range data for Xeric Clade species: 1) MAP, AI and AET/PET; 2) P_dry_; 3) P_seas_ (Fig. S2.10). The first and second of these axes were orthogonal, with the third lying approximately midway between. There was extensive overlap in habitat ranges between species of each of the three Xeric Clade genera. In *Deuterocohnia*, the broadest habitat range occurred in the miniaturised *De. strobilifera* Mez. *De. brevifolia* (Griseb.) M.A.Spencer & L.B.Sm. appeared to be sensitive to P_dry_ but not overall moisture or P_seas_, whereas the opposite was true for *De. seramisiana* R.Vásqez, Ibisch & E.Gross. The range of hydrological habitat range scores for *Dyckia* species was higher than for the other two genera, and the broadest habitat ranges occurred in species such as *Dy. pulquinensis* Wittm. *Dy. dawsonii* was strongly restricted by P_dry_ but not overall moisture or P_seas_, while the reverse held for species such as *Dy. exserta* L.B.Sm. In *Encholirium*, the broadest habitat range occurred in *E. spectabile*. P_dry_ appeared to be more restrictive than either overall moisture or P_seas_ for *E. ctenophyllum* Forzza & Zappi, whereas the opposite was true for *E. biflorum* Mez (Forzza).


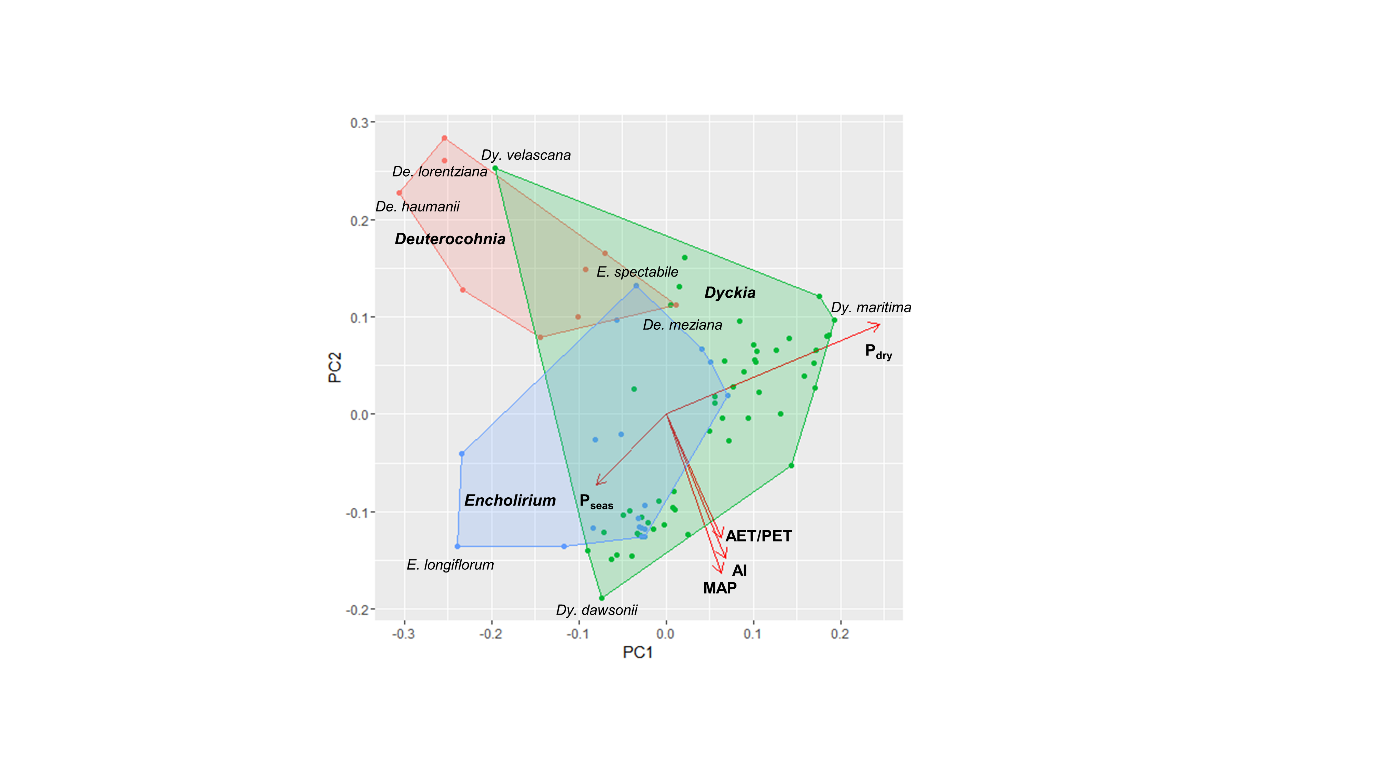


Figure S2.9. PC1-PC2 biplot based on PCA of mean values of bioclimatic variables (MAP, AI, AET/PET, P_dry_, P_seas_) for Xeric Clade Pitcairnioideae (*n* = 79). Species scores are plotted and grouped by genus, with separate convex hulls covering all species belonging to each genus. PC1-PC2 scores for selected species are highlighted and arrows show bioclimatic variable loadings.


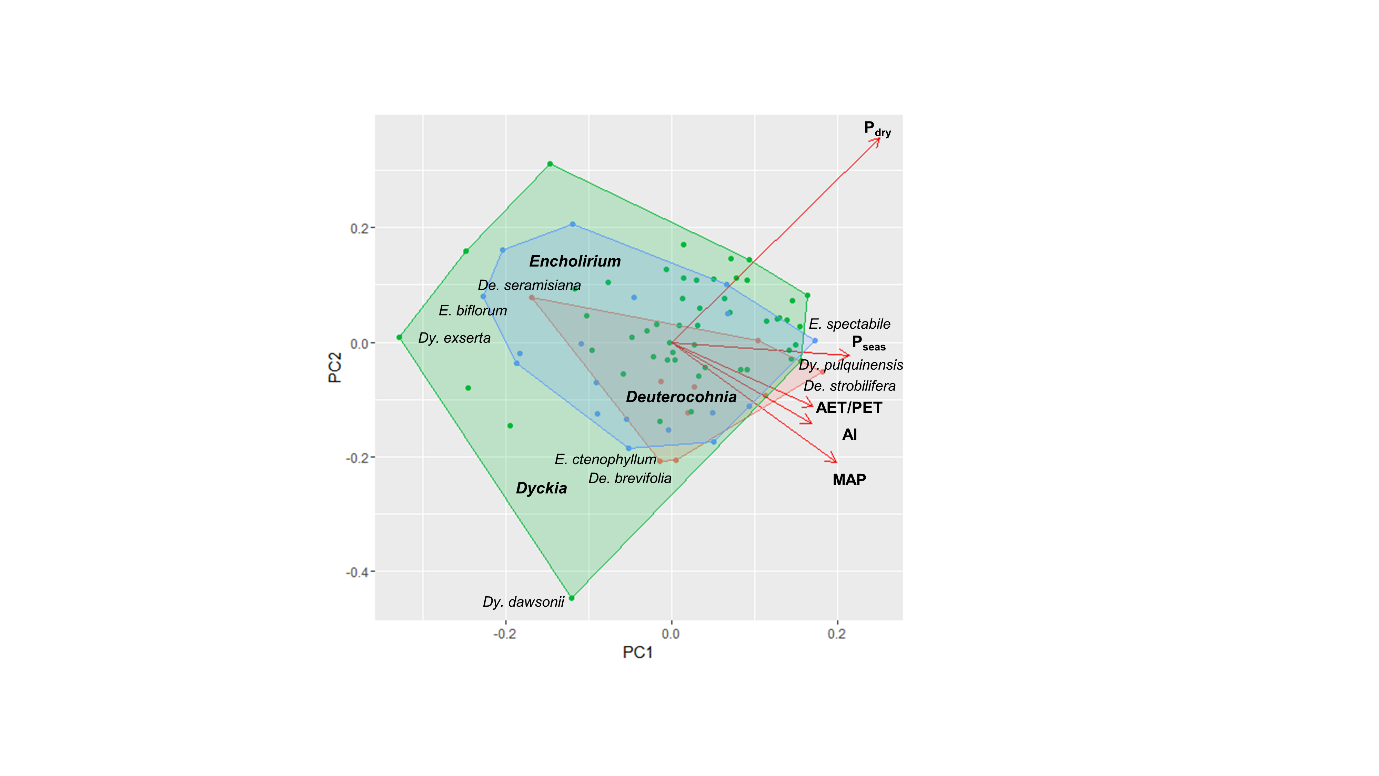


Figure S2.10. PC1-PC2 biplot based on PCA of ranges of bioclimatic variables (MAP, AI, AET/PET, P_dry_, P_seas_) for Xeric Clade Pitcairnioideae (*n* = 79). Species scores are plotted and grouped by genus, with separate convex hulls covering all species belonging to each genus. PC1-PC2 scores for selected species are highlighted and arrows show bioclimatic variable loadings.

Puya (Puyoideae)

Position

PC1- 67.1%

PC2- 20.1%

Range

PC1- 64.2%

PC2- 19.9%

PCA performed on habitat position data for *Puya* revealed a different association between bioclimatic variables from that which was observed in all other taxonomic groups (Fig. S2.11). MAP, AI, and P_dry_ were all approximately aligned along one axis (corresponding to PC1), with P_seas_ also aligned in the opposite direction, while AET/PET alone formed an orthogonal axis (PC2). Most *Puya* spp. clustered relatively tightly in PC1-PC2 climate space, but some species occupied more extreme regions. The most aseasonal, high-moisture environments were occupied by *P. fulgens* L.B.Sm. from the Peruvian Amazon. The Colombian montane forest species *P. roldanii* Betancur & Callejas scored lowly on PC1, suggesting its association with high-moisture environments, but highly on PC2, indicative of low AET/PET. Meanwhile the Argentinian *P. harmsii* (A.Cast.) A.Cast. occurred in the most seasonal, low-moisture environments, and *P. volcanensis* A.Cast., from the same geographical region, showed a similar habitat position except that it displayed a higher AET/PET score.

When habitat range data for *Puya* was subjected to PCA, two axes of variation were revealed, one corresponding to AET/PET and the other to all remaining bioclimatic variables (Fig. S2.12). One species showing particularly broad habitat range was *P. boliviensis* Baker. The Peruvian species *P. angusta* L.B.Sm. was more strongly restricted by AET/PET than by other factors, while the opposite was true of the Ecuadorean *P. sodiroana* Mez.


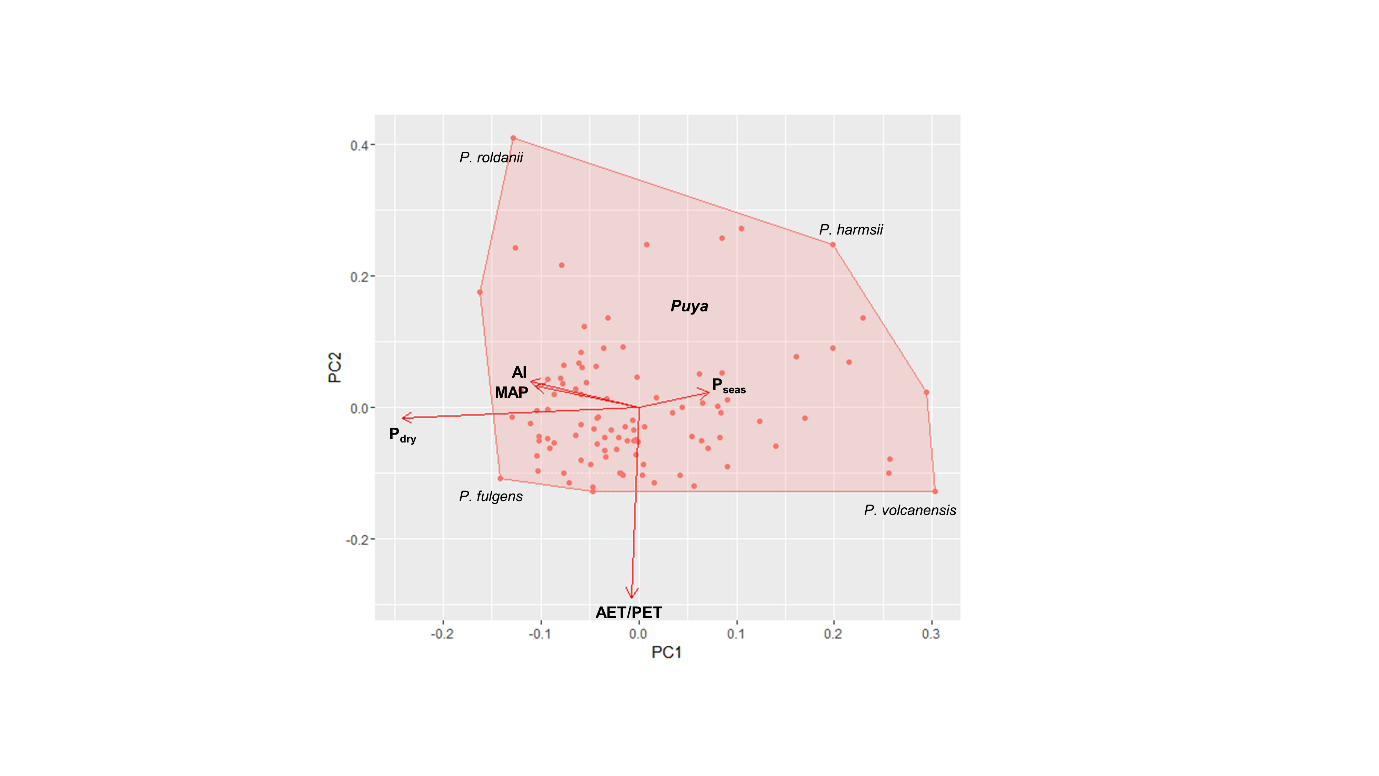


Figure S2.11. PC1-PC2 biplot based on PCA of mean values of bioclimatic variables (MAP, AI, AET/PET, P_dry_, P_seas_) for Puyoideae (*n* = 99). Convex hull covers all species. PC1-PC2 scores for selected species are highlighted and arrows show bioclimatic variable loadings.


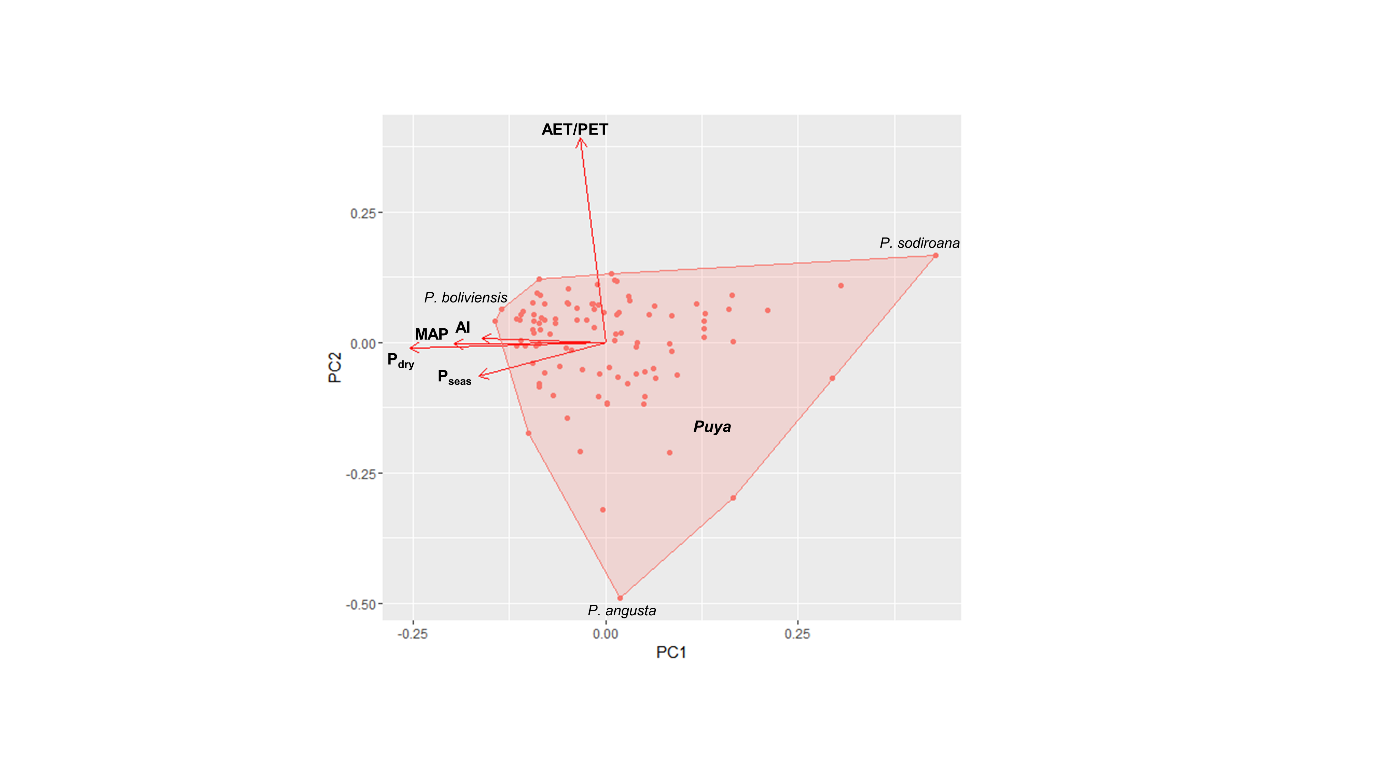


Figure S2.12. PC1-PC2 biplot based on PCA of ranges of bioclimatic variables (MAP, AI, AET/PET, P_dry_, P_seas_) for Puyoideae (*n* = 99). Convex hull covers all species. PC1-PC2 scores for selected species are highlighted and arrows show bioclimatic variable loadings

*C_3_ early-diverging Bromelioideae*

Position

PC1- 69.2%

PC2- 22.8%

Range

PC1- 62.5%

PC2- 16.5%

PCA for habitat position data for the four strictly C_3_ genera of early-diverging Bromelioideae (*Fascicularia*, *Fernseea*, *Greigia* and *Ochagavia*) were broadly consistent with the overall moisture vs. seasonality distinction (Fig. S2.13). The largest of the four genera is *Greigia*. *Greigia* spp. hydrological habitat positions varied roughly equally along the total moisture and seasonality axes. Andean species such as *G. sodiroana* Mez occupied the least seasonal but lowest overall moisture environments, while *G. kessleri* H.E.Luther occurred in more seasonal, low-moisture environments. Higher-moisture, seasonal environments were occupied by Central American species such as *G. rohwederi* L.B.Sm., while the species associated with the highest levels of overall moisture and moderate seasonality was *G. sylvicola* Standl.*.* Habitat positions of species of all other genera fell within the range occupied by *Greigia* spp. The monospecific Chilean genus *Fascicularia* was located in the high-moisture and moderately seasonal region of the climate space, while the single *Fernseea* species analysed was also associated with relatively high levels of moisture but stronger seasonality. Both Chilean *Ochagavia* species were associated with lower total moisture and strong seasonality.

PCA of habitat range data for C_3_ early-diverging bromelioid genera identified two groups of aligned bioclimatic variable loadings corresponding to overall moisture and seasonality (Fig. S2.14). The broadest hydrological habitat ranges occurred in the Ecuadorean *Greigia sodiroana*. The Bolivian *G. kessleri* was more sensitive to seasonality than total moisture, with the opposite being true for the Venezuelan *G. alborosea* (Griseb.) Mez. Both *Fascicularia bicolor* (Ruiz & Pav.) Mez and *Ochagavia litoralis* (Phil.) Zizka, Trumpler & Zöllner showed relatively broad habitat ranges, while that of *O. carnea* (Beer) L.B.Sm. & Looser was slightly narrower with respect to both overall moisture and seasonality. *Fernseea itatiaeae* (Wawra) Baker appeared to be considerably more sensitive to seasonality than to overall moisture.


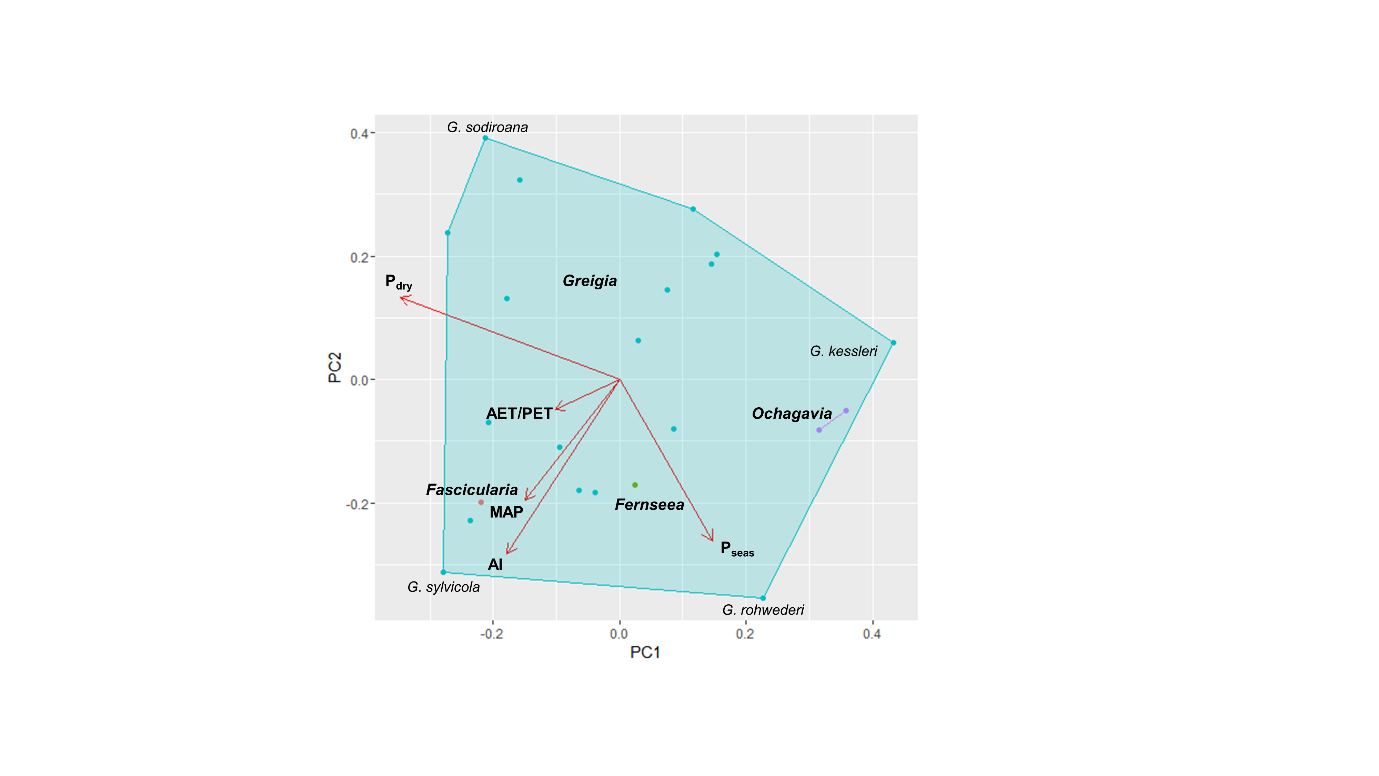


Figure S2.13. PC1-PC2 biplot based on PCA of mean values of bioclimatic variables (MAP, AI, AET/PET, P_dry_, P_seas_) for C_3_ early-diverging Bromelioideae (*n* = 22). Species scores are plotted and grouped by genus, with separate convex hulls covering all species belonging to each genus. PC1-PC2 scores for selected species are highlighted and arrows show bioclimatic variable loadings.


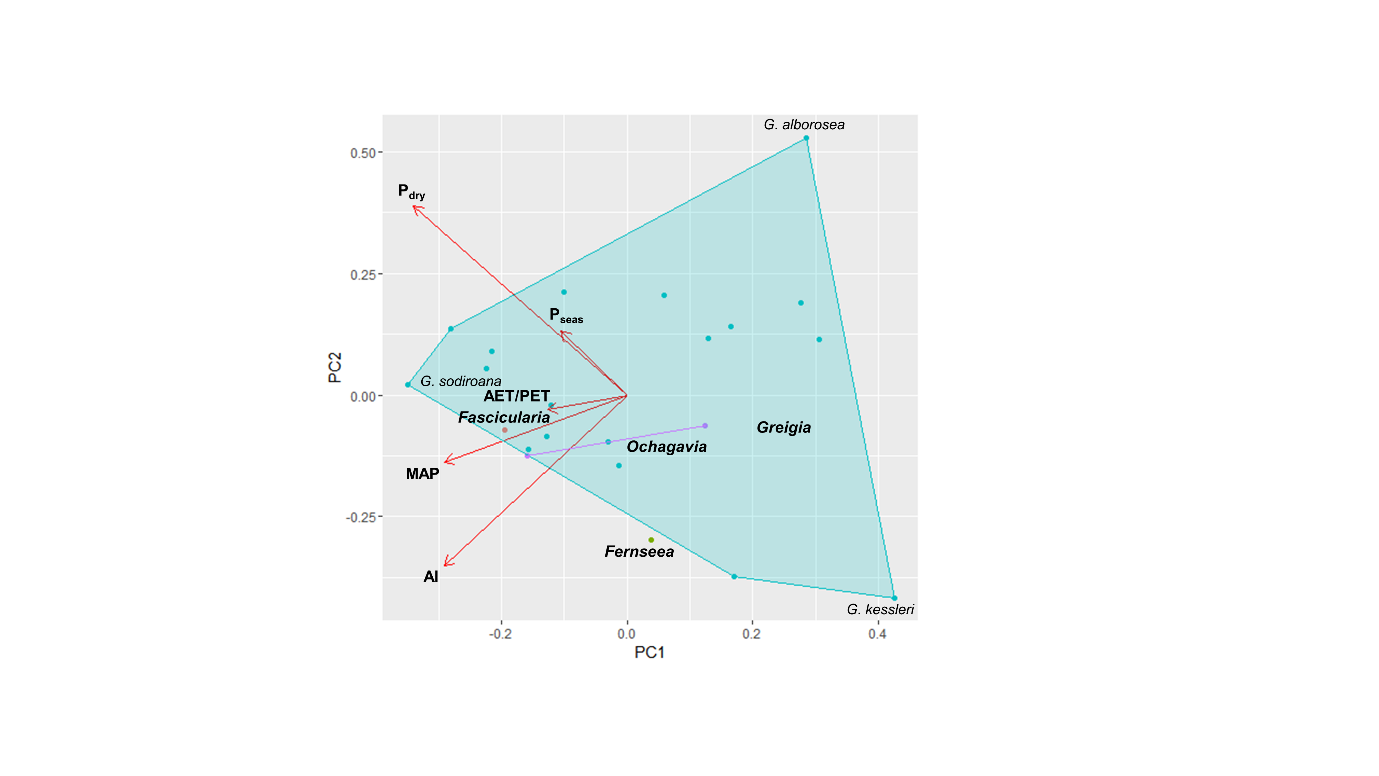


Figure S2.14. PC1-PC2 biplot based on PCA of ranges of bioclimatic variables (MAP, AI, AET/PET, P_dry_, P_seas_) for C_3_ early-diverging Bromelioideae (*n* = 22). Species scores are plotted and grouped by genus, with separate convex hulls covering all species belonging to each genus. PC1-PC2 scores for selected species are highlighted and arrows show bioclimatic variable loadings.

*CAM early-diverging Bromelioideae*

Position

PC1- 67.7%

PC2- 27.1%

Range

PC1- 80.9%

PC2- 8.7%

There was a clear overall moisture vs. seasonality distinction when PCA was performed on hydrological habitat positions for the CAM genera in the early-diverging Bromelioideae (Fig. S2.15). Among the CAM genera in the early-diverging Bromelioideae, the most distinctive genus was *Disteganthus*. All three species represented in the dataset clustered closely in the area of the PC1-PC2 climate space corresponding to high moisture and low seasonality. At the opposite end of the spectrum, the genera *Deinacanthon* and *Neoglaziovia* were associated with some of the driest and most seasonal environments. The genus with the broadest range of habitat positions was *Bromelia*. Species such as *B. tubulosa* L.B.Sm. were associated with less seasonal, high-moisture habitats, and indeed this species co-occurs with *Disteganthus* spp. in northern Amazonia. Among *Bromelia* spp. associated with high seasonality, there was a distinction between species such as *B. irwinii* L.B.Sm. which inhabit regions with higher overall moisture, and species like *B. arenaria* Ule which are restricted to drier regions. In *Cryptanthus*, the only species with a high-moisture, aseasonal habitat was *C. pseudopetiolatus* Philcox. As its name suggests, this species again displays pseudopetiolate leaves. In more seasonal but relatively high-moisture environments occurred species such as *C. schwackeanus* Mez, which displays a C_3_ δ^13^C signature (Crayn et al., 2015). Finally, the range of habitat positions in the genus *Orthophytum* was centred furthest towards the drier, more seasonal corner of the PC1-PC2 climate space, but was still extensive. Species associated with the lowest overall moisture levels and moderate degrees of seasonality included the strongly xeromorphic, acaulescent rosette species *O. saxicola* (Ule) L.B.Sm., while caulescent species like *O. schulzianum* Leme & M.Machado were associated with more seasonal environments with higher overall moisture.


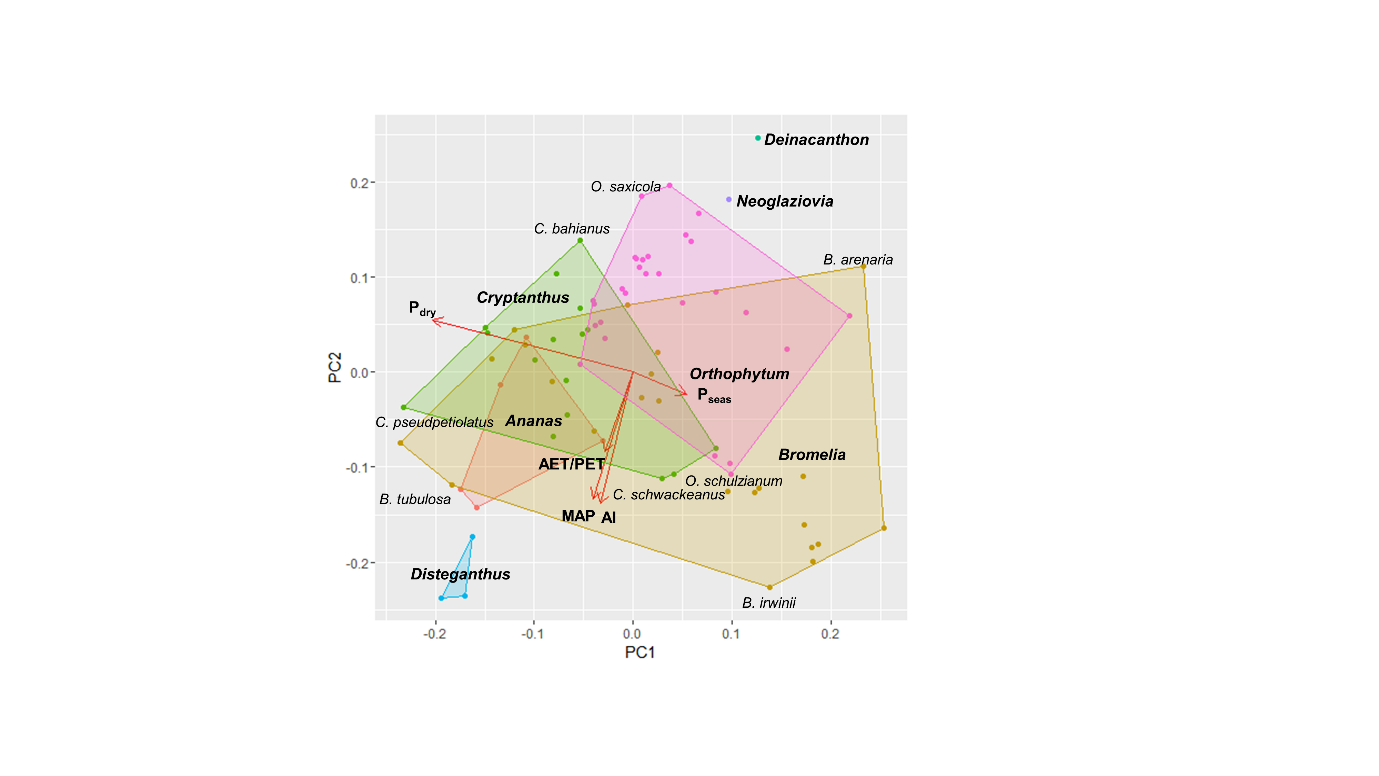


Figure S2.15. PC1-PC2 biplot based on PCA of mean values of bioclimatic variables (MAP, AI, AET/PET, P_dry_, P_seas_) for CAM early-diverging Bromelioideae (*n* = 77). Species scores are plotted and grouped by genus, with separate convex hulls covering all species belonging to each genus. PC1-PC2 scores for selected species are highlighted and arrows show bioclimatic variable loadings.


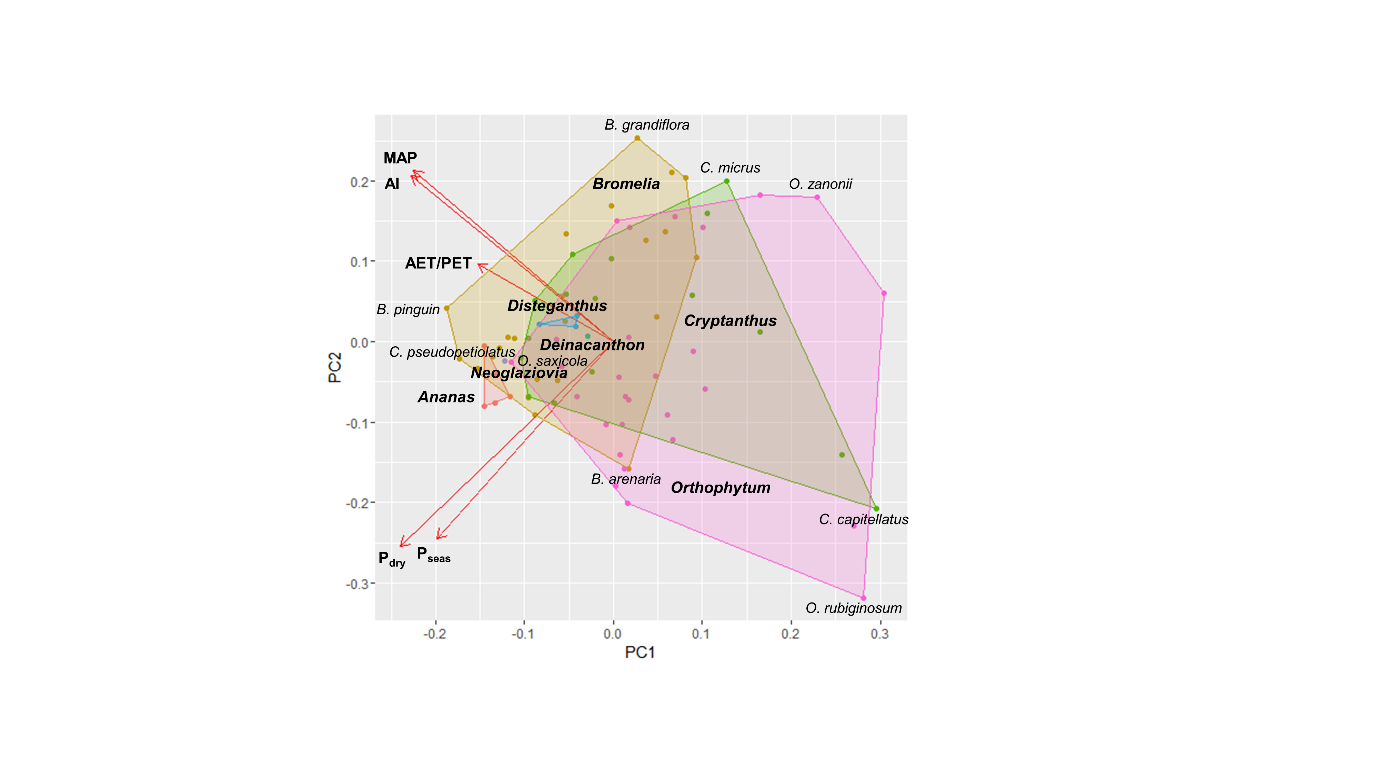


Figure S2.16. PC1-PC2 biplot based on PCA of ranges of bioclimatic variables (MAP, AI, AET/PET, P_dry_, P_seas_) for CAM early-diverging Bromelioideae (*n* = 77). Species scores are plotted and grouped by genus, with separate convex hulls covering all species belonging to each genus. PC1-PC2 scores for selected species are highlighted and arrows show bioclimatic variable loadings.

**Supporting Information Table S1**. Geographical and bioclimatic properties of the distributions of the 564 terrestrial bromeliad species analysed in this investigation.

**
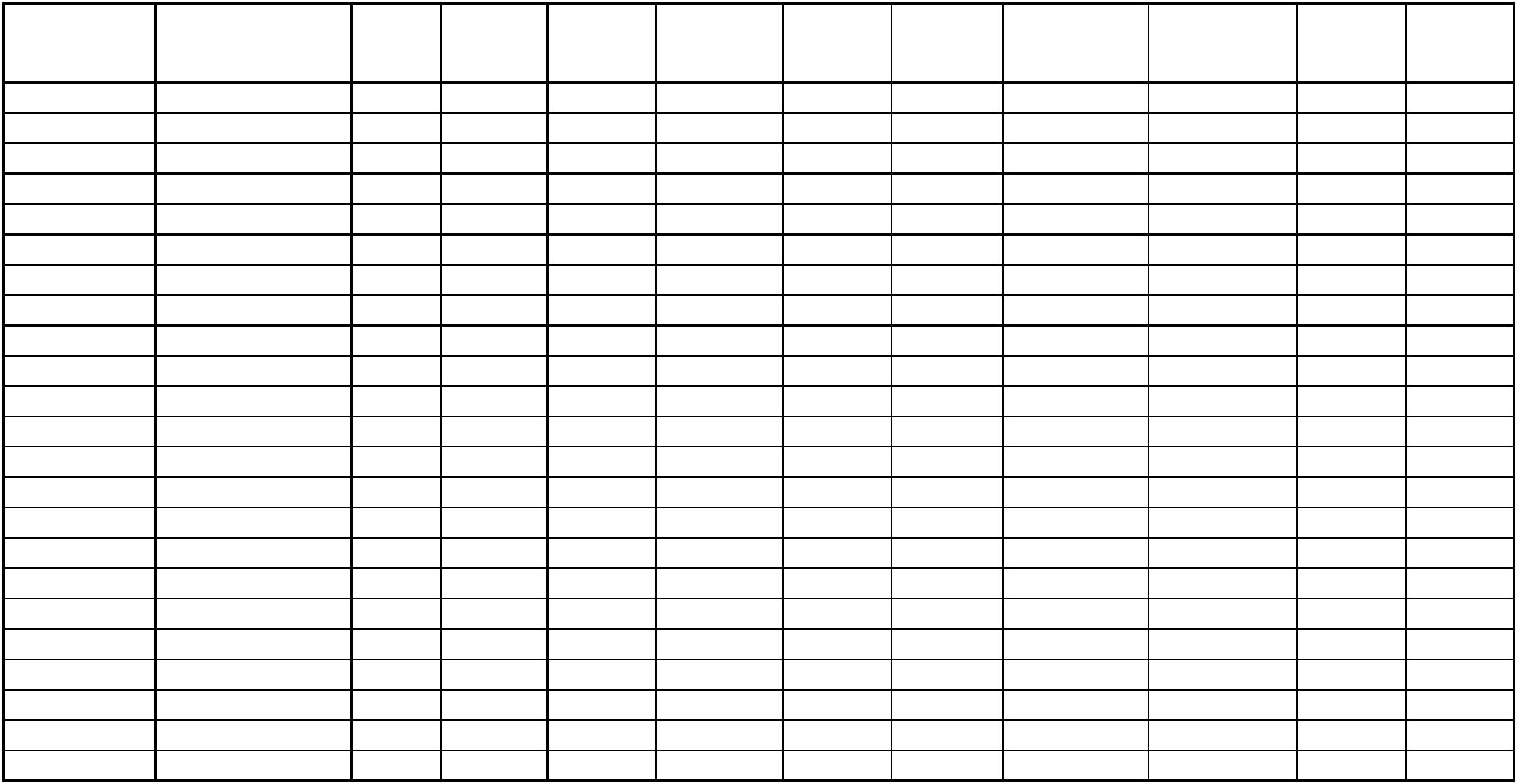
**

|  |  |  |  |  |  | **Alt (m)** | **Alt (m)** | **AI (mm mm-1)** | **AI (mm mm-1)** | **MAP (mm)** | **MAP (mm)** |
| --- | --- | --- | --- | --- | --- | --- | --- | --- | --- | --- | --- |
| **Genus** | **Species** | **Tax Grp** | **Fun Type n** |  | **Range (km2)** | **mean** | **range** | **mean** | **range** | **mean** | **range** |
| **Ananas** | **ananassoides** | **CAMEDB** | **camterr** | **190** | **12415878** | **412.24** | **1547.75** | **9944.85** | **17133.49** | **1697.42** | **2547.50** |
| **Ananas** | **bracteatus** | **CAMEDB** | **camterr** | **46** | **4025537** | **350.50** | **1046.00** | **10600.67** | **13583.65** | **1528.67** | **1519.00** |
| **Ananas** | **lucidus** | **CAMEDB** | **camterr** | **12** | **5186132** | **412.33** | **1118.00** | **15190.39** | **13960.21** | **2415.79** | **2310.92** |
| **Ananas** | **parguazensis** | **CAMEDB** | **camterr** | **47** | **3304945** | **323.09** | **1255.50** | **15027.37** | **12014.89** | **2568.67** | **1784.75** |
| **Ananas** | **sagenaria** | **CAMEDB** | **camterr** | **168** | **4707529** | **231.70** | **1377.58** | **8630.54** | **8268.96** | **1324.32** | **1307.08** |
| **Brewcaria** | **hohenbergioides** | **Nav** | **c3mes** | **7** | **24605** | **200.94** | **336.75** | **15846.41** | **4763.30** | **2898.64** | **786.25** |
| **Brewcaria** | **marahuacae** | **Nav** | **c3mes** | **13** | **817** | **1303.78** | **2002.42** | **20419.38** | **9009.57** | **3058.78** | **508.00** |
| **Brewcaria** | **reflexa** | **Nav** | **c3mes** | **12** | **53039** | **397.74** | **1833.33** | **18426.89** | **9413.03** | **3254.56** | **600.67** |
| **Brocchinia** | **delicatula** | **BL** | **c3mes** | **3** | **70** | **1723.34** | **400.07** | **23687.54** | **3205.47** | **3337.82** | **249.75** |
| **Brocchinia** | **hechtioides** | **BL** | **c3mes** | **32** | **848031** | **996.98** | **2244.75** | **18319.33** | **13288.97** | **2826.52** | **1522.50** |
| **Brocchinia** | **paniculata** | **BL** | **c3mes** | **28** | **354678** | **252.29** | **1297.67** | **19066.90** | **8010.36** | **3136.92** | **1204.75** |
| **Brocchinia** | **prismatica** | **BL** | **c3mes** | **15** | **12039** | **111.92** | **71.08** | **17644.27** | **1661.95** | **3218.32** | **493.92** |
| **Brocchinia** | **reducta** | **BL** | **c3mes** | **19** | **41059** | **1104.64** | **1847.50** | **17094.66** | **10105.76** | **2545.85** | **1419.08** |
| **Brocchinia** | **rupestris** | **BL** | **c3mes** | **3** | **267** | **450.89** | **422.42** | **11193.19** | **4018.83** | **1747.36** | **662.67** |
| **Brocchinia** | **tatei** | **BL** | **c3mes** | **30** | **199746** | **1072.97** | **2203.75** | **18182.63** | **12788.46** | **2806.91** | **1838.25** |
| **Brocchinia** | **vestita** | **BL** | **c3mes** | **8** | **6058** | **1034.59** | **919.00** | **20599.18** | **6578.93** | **3224.30** | **340.92** |
| **Bromelia** | **antiacantha** | **CAMEDB** | **camterr** | **73** | **2313053** | **228.36** | **1140.25** | **9192.34** | **13733.69** | **1287.63** | **1907.33** |
| **Bromelia** | **arenaria** | **CAMEDB** | **camterr** | **6** | **134453** | **562.63** | **479.00** | **4062.30** | **1011.37** | **741.08** | **235.00** |
| **Bromelia** | **balansae** | **CAMEDB** | **camterr** | **161** | **1070673** | **368.47** | **1404.00** | **9184.74** | **14951.13** | **1510.18** | **2397.50** |
| **Bromelia** | **binotii** | **CAMEDB** | **camterr** | **9** | **87714** | **275.43** | **888.08** | **8977.13** | **11178.29** | **1263.76** | **1436.08** |
| **Bromelia** | **braunii** | **CAMEDB** | **camterr** | **4** | **8630** | **471.79** | **250.17** | **7978.41** | **1289.93** | **1411.25** | **343.33** |
| **Bromelia** | **chrysantha** | **CAMEDB** | **camterr** | **11** | **368863** | **246.67** | **1157.50** | **7190.33** | **4383.91** | **1184.40** | **743.50** |
| **Bromelia** | **goyazensis** | **CAMEDB** | **camterr** | **5** | **328524** | **778.65** | **845.50** | **9011.29** | **7580.95** | **1517.83** | **1354.25** |

**
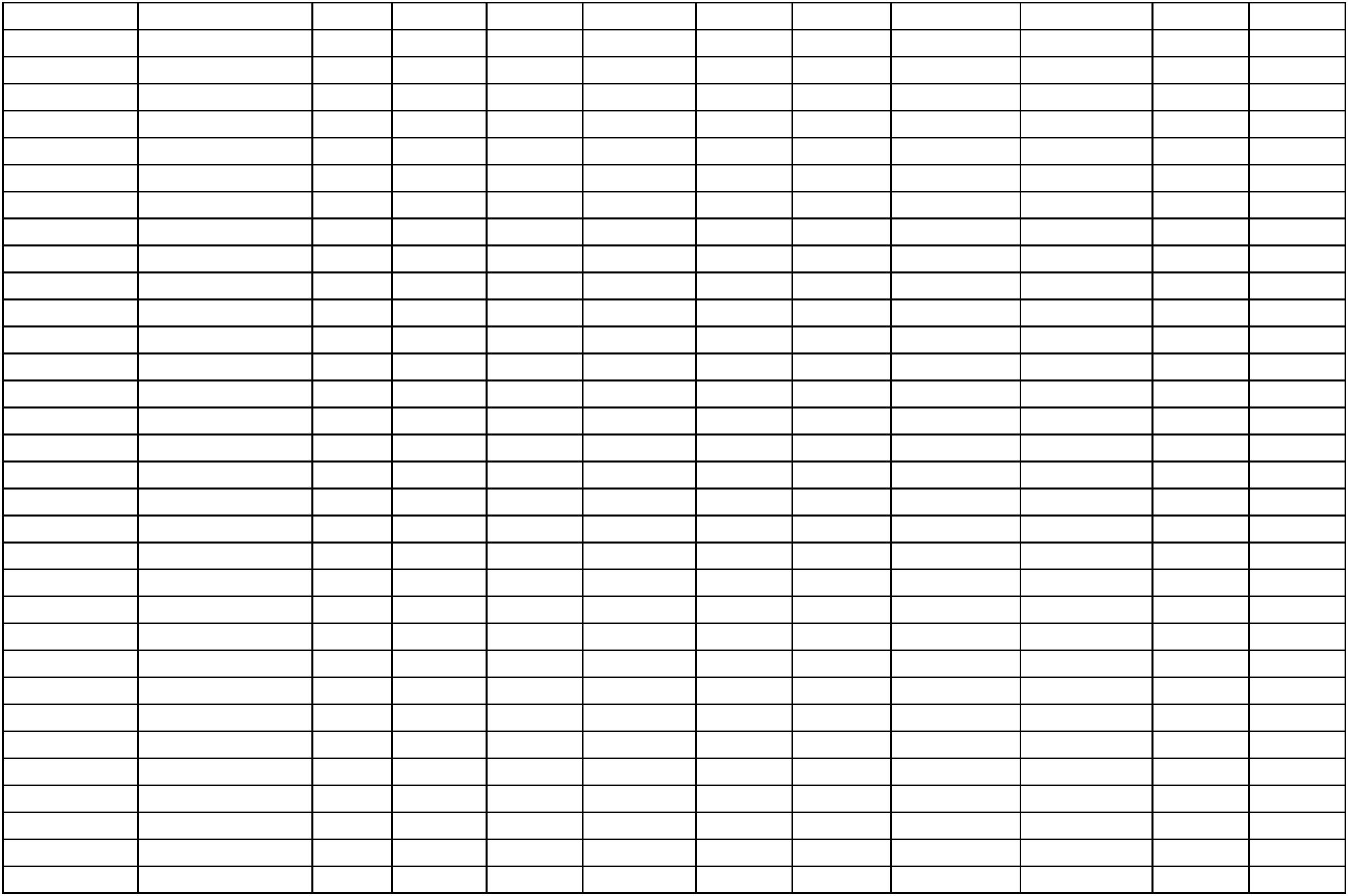
**

| **Bromelia** | **grandiflora** | **CAMEDB** | **camterr** | **3** | **196524** | **467.00** | **270.50** | **9008.72** | **4531.01** | **1624.69** | **1033.92** |
| --- | --- | --- | --- | --- | --- | --- | --- | --- | --- | --- | --- |
| **Bromelia** | **hemisphaerica** | **CAMEDB** | **camterr** | **5** | **141** | **1288.07** | **687.33** | **8109.26** | **3205.94** | **1411.55** | **379.00** |
| **Bromelia** | **ignaciana** | **CAMEDB** | **camterr** | **3** | **11875** | **303.89** | **98.83** | **7943.52** | **4547.04** | **1430.08** | **799.75** |
| **Bromelia** | **interior** | **CAMEDB** | **camterr** | **19** | **816844** | **565.04** | **1371.75** | **9513.13** | **6738.49** | **1583.53** | **832.25** |
| **Bromelia** | **irwinii** | **CAMEDB** | **camterr** | **6** | **8022** | **1143.96** | **1169.00** | **12238.50** | **2033.16** | **1893.83** | **435.25** |
| **Bromelia** | **karatas** | **CAMEDB** | **camterr** | **159** | **12996159** |  | **2266.25** | **10242.44** | **20949.70** | **1675.65** | **3642.08** |
| **Bromelia** | **lagopus** | **CAMEDB** | **camterr** | **6** | **322162** | **502.01** | **695.75** | **7875.33** | **5939.04** | **1258.75** | **1104.50** |
| **Bromelia** | **macedoi** | **CAMEDB** | **camterr** | **5** | **15015** | **590.63** | **677.25** | **9603.97** | **1840.29** | **1587.55** | **233.00** |
| **Bromelia** | **minima** | **CAMEDB** | **camterr** | **4** | **285** | **884.19** | **613.25** | **10342.46** | **3329.65** | **1658.19** | **317.75** |
| **Bromelia** | **pinguin** | **CAMEDB** | **camterr** | **208** | **9446329** | **199.27** | **2075.00** | **8505.14** | **35417.51** | **1401.48** | **5379.67** |
| **Bromelia** | **reversacantha** | **CAMEDB** | **camterr** | **6** | **54506** | **531.33** | **497.50** | **9652.74** | **2365.13** | **1650.54** | **695.92** |
| **Bromelia** | **serra** | **CAMEDB** | **camterr** | **67** | **3441607** | **639.14** | **1831.00** | **6675.42** | **11796.45** | **1080.74** | **1946.75** |
| **Bromelia** | **tarapotina** | **CAMEDB** | **camterr** | **3** | **1019** | **140.75** | **83.50** | **13826.31** | **7233.76** | **2385.92** | **1151.75** |
| **Bromelia** | **tubulosa** | **CAMEDB** | **camterr** | **16** | **1494568** | **182.47** | **462.00** | **14911.90** | **13349.93** | **2422.97** | **2105.50** |
| **Bromelia** | **unaensis** | **CAMEDB** | **camterr** | **8** | **103825** | **168.79** | **474.00** | **10161.93** | **7042.27** | **1407.65** | **679.70** |
| **Bromelia** | **villosa** | **CAMEDB** | **camterr** | **11** | **771662** | **453.30** | **724.75** | **8902.56** | **5959.39** | **1583.09** | **1255.50** |
| **Connellia** | **augustae** | **BL** | **c3mes** | **7** | **24971** | **1064.01** | **1604.10** | **13583.24** | **3281.03** | **2049.19** | **733.00** |
| **Connellia** | **caricifolia** | **BL** | **c3mes** | **3** | **3** | **1510.44** | **569.17** | **13749.71** | **2166.41** | **1928.00** | **272.00** |
| **Connellia** | **quelchii** | **Bl** | **c3mes** | **7** | **5264** | **1389.94** | **1597.25** | **14504.72** | **4271.94** | **2087.24** | **645.75** |
| **Cottendorfia** | **florida** | **Nav** | **c3mes** | **43** | **17151** | **994.75** | **1013.75** | **5824.18** | **1375.66** | **912.84** | **282.67** |
| **Cryptanthus** | **bahianus** | **CAMEDB** | **camterr** | **14** | **212380** | **368.44** | **704.75** | **6426.53** | **9446.66** | **910.51** | **1092.83** |
| **Cryptanthus** | **beuckeri** | **CAMEDB** | **camterr** | **20** | **32599** | **125.43** | **325.25** | **9442.64** | **7521.68** | **1313.98** | **851.00** |
| **Cryptanthus** | **capitellatus** | **CAMEDB** | **camterr** | **3** | **2** | **338.33** | **61.25** | **7758.50** | **22.25** | **1207.00** | **10.50** |
| **Cryptanthus** | **dianae** | **CAMEDB** | **camterr** | **11** | **7902** | **282.88** | **543.42** | **10902.51** | **5314.94** | **1497.70** | **634.67** |
| **Cryptanthus** | **ferrarius** | **CAMEDB** | **camterr** | **4** | **371** | **1187.81** | **227.50** | **10713.20** | **1920.39** | **1529.31** | **116.25** |
| **Cryptanthus** | **giganteus** | **CAMEDB** | **camterr** | **3** | **1** | **226.75** | **102.75** | **7581.15** | **125.85** | **1185.92** | **23.75** |
| **Cryptanthus** | **maritimus** | **CAMEDB** | **camterr** | **4** | **5145** | **42.83** | **127.25** | **9381.80** | **4726.90** | **1296.58** | **595.92** |
| **Cryptanthus** | **micrus** | **CAMEDB** | **camterr** | **3** | **5** | **950.31** | **151.83** | **8685.84** | **647.93** | **1318.72** | **68.50** |
| **Cryptanthus** | **pickelii** | **CAMEDB** | **camterr** | **6** | **9511** | **53.00** | **163.42** | **12019.93** | **8609.51** | **1614.81** | **1003.25** |
| **Cryptanthus** | **pseudopetiolatus** | **CAMEDB** | **camterr** | **31** | **17636** | **74.75** | **215.50** | **14112.89** | **10799.27** | **1832.94** | **1238.10** |
| **Cryptanthus** | **sanctaluciae** | **CAMEDB** | **camterr** | **8** | **57** | **446.63** | **545.67** | **8459.25** | **1369.40** | **1225.46** | **135.25** |
| **Cryptanthus** | **schwackeanus** | **CAMEDB** | **c3terr** | **25** | **57744** | **1072.99** | **949.25** | **10256.03** | **5163.50** | **1502.61** | **450.75** |
| **Cryptanthus** | **sergipensis** | **CAMEDB** | **camterr** | **12** | **3712** | **95.08** | **227.93** | **9418.55** | **6097.54** | **1401.58** | **934.00** |

**
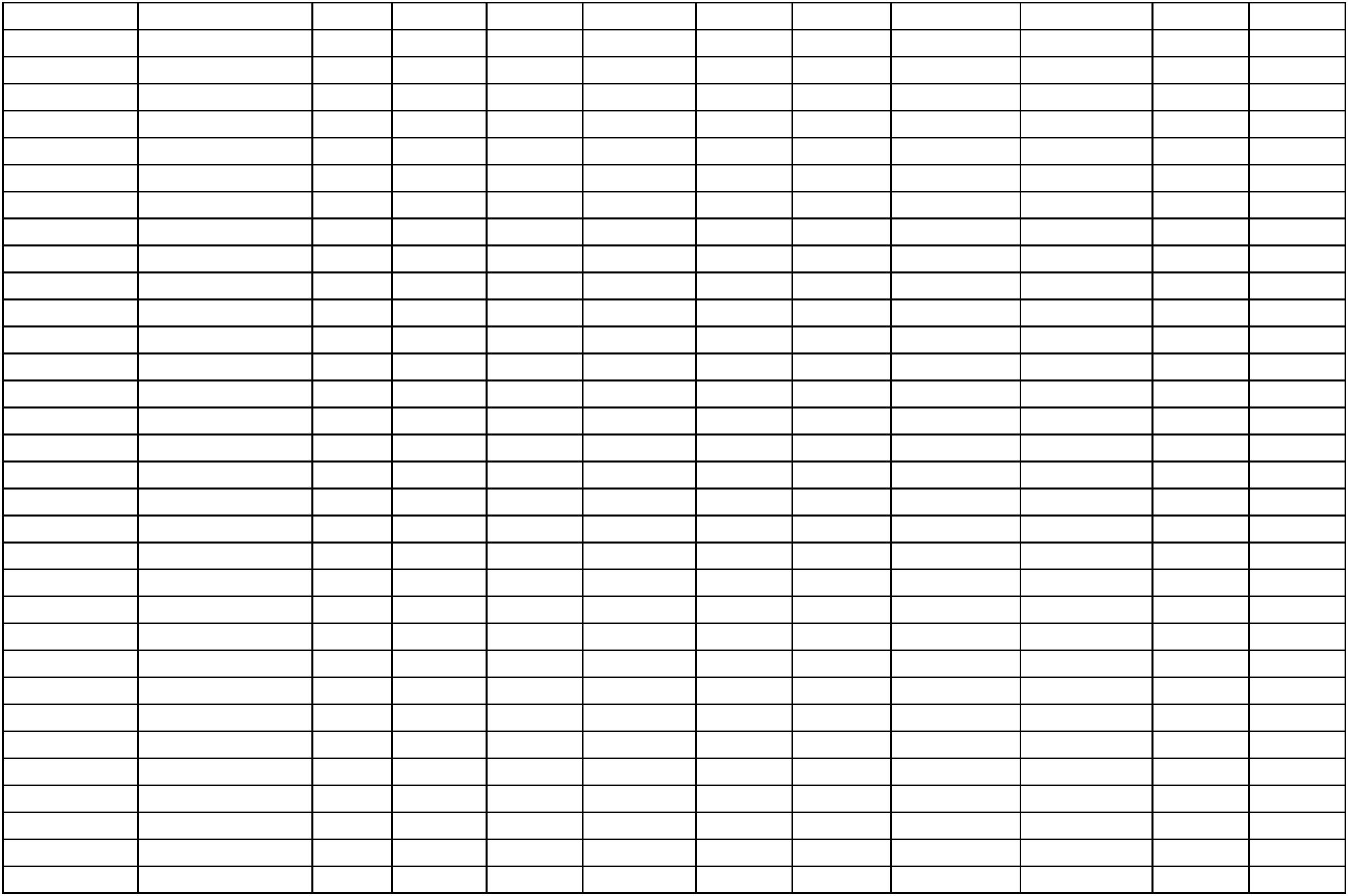
**

| **Cryptanthus** | **venecianus** | **CAMEDB** | **camterr** | **3** | **1** | **164.19** | **1.67** | **7250.63** | **20.55** | **1140.78** | **2.33** |
| --- | --- | --- | --- | --- | --- | --- | --- | --- | --- | --- | --- |
| **Cryptanthus** | **warren-loosei** | **CAMEDB** | **camterr** | **4** | **19913** | **272.13** | **512.50** | **7442.29** | **9834.40** | **1045.50** | **1245.00** |
| **Cryptanthus** | **zonatus** | **CAMEDB** | **camterr** | **4** | **37423** | **138.65** | **244.67** | **9491.98** | **3739.64** | **1417.08** | **608.00** |
| **Deinacanthon** | **urbanianum** | **CAMEDB** | **camterr** | **12** | **443417** | **320.12** | **753.00** | **3507.71** | **3707.84** | **592.43** | **525.17** |
| **Deuterocohnia** | **brevifolia** | **XC** | **camterr** | **4** | **3429** | **2276.50** | **658.25** | **2744.31** | **2404.04** | **407.75** | **412.50** |
| **Deuterocohnia** | **haumanii** | **XC** | **camterr** | **12** | **40796** | **1853.88** | **1716.50** | **1746.49** | **3263.63** | **244.85** | **414.75** |
| **Deuterocohnia** | **longipetala** | **XC** | **camterr** | **36** | **769394** | **1176.83** | **4130.25** | **3734.42** | **6497.86** | **596.52** | **1263.75** |
| **Deuterocohnia** | **lorentziana** | **XC** | **camterr** | **7** | **35772** | **2735.61** | **2880.50** | **1836.67** | **1688.06** | **256.80** | **253.00** |
| **Deuterocohnia** | **meziana** | **XC** | **camterr** | **20** | **395199** | **605.52** | **1948.50** | **5479.17** | **4988.49** | **924.68** | **882.83** |
| **Deuterocohnia** | **scapigera** | **XC** | **camterr** | **4** | **7119** | **2043.15** | **1128.75** | **4276.74** | **3089.96** | **637.98** | **419.83** |
| **Deuterocohnia** | **schreiteri** | **XC** | **camterr** | **10** | **25336** | **1748.73** | **1255.00** | **1724.98** | **2519.34** | **241.03** | **367.00** |
| **Deuterocohnia** | **seramisiana** | **XC** | **camterr** | **3** | **312** | **2529.81** | **1066.58** | **4061.36** | **290.85** | **585.00** | **45.50** |
| **Deuterocohnia** | **strobilifera** | **XC** | **camterr** | **10** | **51904** | **2918.81** | **3868.55** | **3946.08** | **16548.72** | **621.73** | **2834.55** |
| **Disteganthus** | **basilateralis** | **CAMEDB** | **camterr** | **25** | **9658** | **94.81** | **162.33** | **22345.97** | **7284.59** | **3491.51** | **956.25** |
| **Disteganthus** | **calatheoides** | **CAMEDB** | **camterr** | **7** | **8631** | **60.42** | **61.83** | **21489.71** | **6178.60** | **3324.49** | **960.67** |
| **Disteganthus** | **lateralis** | **CAMEDB** | **camterr** | **21** | **187812** | **136.46** | **288.58** | **17294.38** | **9769.31** | **2709.89** | **1601.00** |
| **Dyckia** | **affinis** | **XC** | **camterr** | **3** | **5544** | **98.50** | **76.50** | **8545.85** | **2842.09** | **1347.75** | **277.25** |
| **Dyckia** | **aurea** | **XC** | **camterr** | **5** | **35980** | **877.57** | **607.90** | **9250.57** | **3063.92** | **1516.90** | **395.25** |
| **Dyckia** | **brachyphylla** | **XC** | **camterr** | **4** | **876** | **1249.56** | **172.50** | **10137.78** | **1108.60** | **1452.50** | **128.65** |
| **Dyckia** | **brasiliana** | **XC** | **camterr** | **35** | **143806** | **936.24** | **949.67** | **9755.18** | **4680.75** | **1533.80** | **852.25** |
| **Dyckia** | **brevifolia** | **XC** | **camterr** | **11** | **139970** | **401.74** | **771.20** | **11474.87** | **4147.70** | **1634.38** | **448.00** |
| **Dyckia** | **burchellii** | **XC** | **camterr** | **13** | **351215** | **598.26** | **1084.00** | **8525.51** | **6136.33** | **1467.96** | **914.50** |
| **Dyckia** | **consimilis** | **XC** | **camterr** | **7** | **244** | **1113.21** | **1034.67** | **10472.95** | **1924.18** | **1611.05** | **788.50** |
| **Dyckia** | **dawsonii** | **XC** | **camterr** | **3** | **1** | **481.58** | **265.25** | **10519.26** | **2680.84** | **1779.33** | **380.75** |
| **Dyckia** | **distachya** | **XC** | **camterr** | **10** | **102796** | **326.33** | **754.00** | **11464.37** | **4748.12** | **1679.85** | **343.00** |
| **Dyckia** | **duckei** | **XC** | **camterr** | **8** | **208359** | **396.71** | **325.75** | **11363.77** | **3621.60** | **2082.55** | **901.17** |
| **Dyckia** | **dusenii** | **XC** | **camterr** | **9** | **14317** | **934.21** | **221.00** | **11671.51** | **4229.42** | **1571.85** | **278.50** |
| **Dyckia** | **elata** | **XC** | **camterr** | **3** | **29** | **1059.00** | **73.75** | **10753.19** | **600.60** | **1497.50** | **26.00** |
| **Dyckia** | **encholirioides** | **XC** | **camterr** | **11** | **255038** | **273.13** | **840.50** | **11982.22** | **9788.70** | **1619.22** | **1098.58** |
| **Dyckia** | **exserta** | **XC** | **camterr** | **3** | **14** | **310.92** | **83.50** | **8610.80** | **104.75** | **1399.81** | **5.42** |
| **Dyckia** | **ferox** | **XC** | **camterr** | **23** | **558065** | **142.75** | **565.95** | **8280.31** | **7723.94** | **1304.54** | **1154.00** |
| **Dyckia** | **ferruginea** | **XC** | **camterr** | **10** | **143689** | **296.40** | **273.00** | **7924.41** | **2173.64** | **1374.87** | **416.00** |
| **Dyckia** | **floribunda** | **XC** | **camterr** | **11** | **808950** | **795.32** | **3613.25** | **5485.11** | **8208.83** | **806.95** | **1283.50** |

**
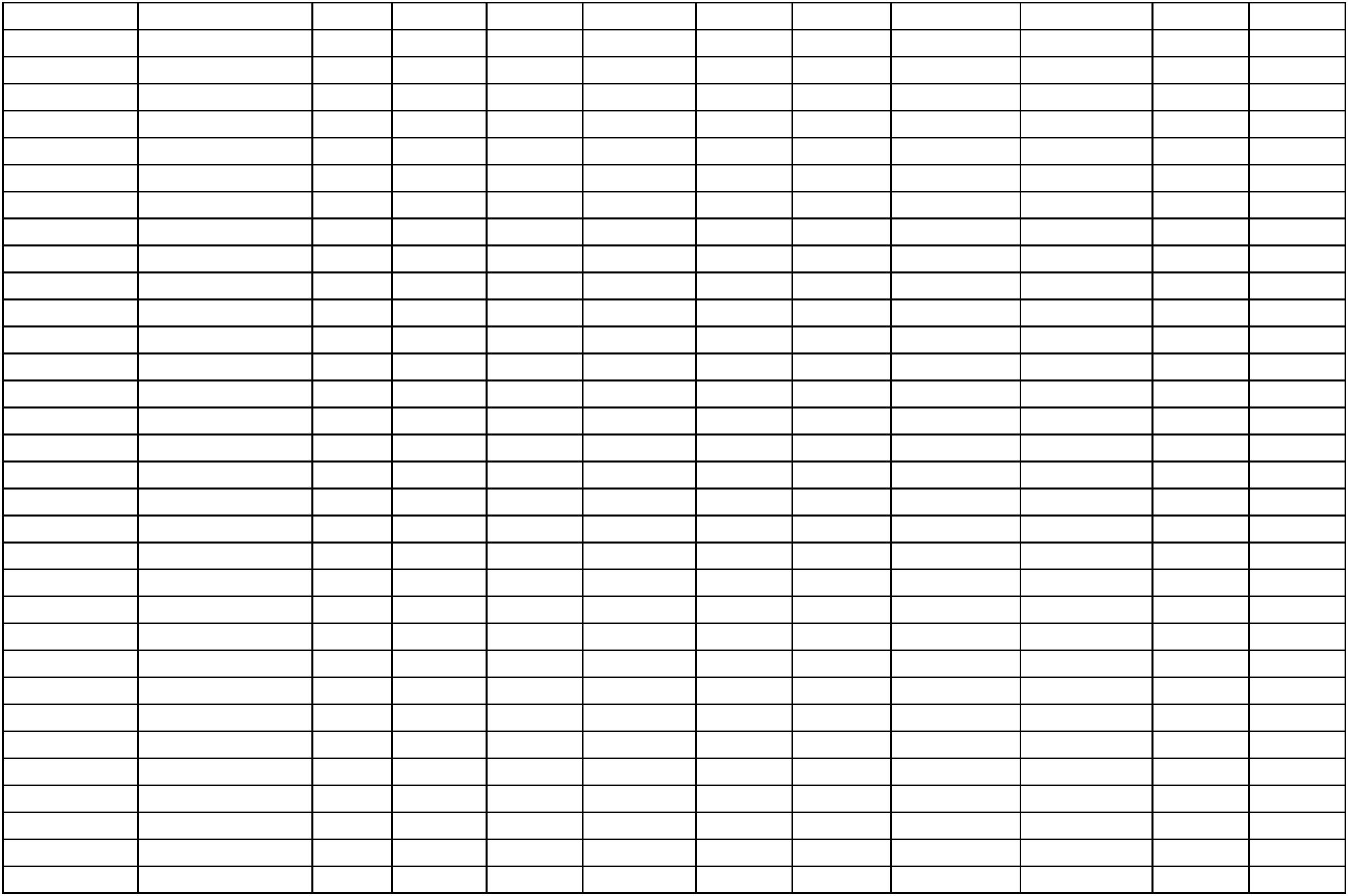
**

| **Dyckia** | **fosteriana** | **XC** | **camterr** | **3** | **16819** | **707.08** | **236.75** | **9343.95** | **1131.95** | **1357.92** | **176.00** |
| --- | --- | --- | --- | --- | --- | --- | --- | --- | --- | --- | --- |
| **Dyckia** | **goiana** | **XC** | **camterr** | **4** | **70284** | **778.73** | **866.25** | **9453.99** | **2518.31** | **1578.40** | **770.00** |
| **Dyckia** | **gracilis** | **XC** | **camterr** | **4** | **99378** | **377.81** | **463.00** | **10112.45** | **15264.90** | **1765.19** | **2523.50** |
| **Dyckia** | **grandidentata** | **XC** | **camterr** | **3** | **5** | **449.75** | **231.75** | **8654.19** | **384.29** | **1486.12** | **5.05** |
| **Dyckia** | **horridula** | **XC** | **camterr** | **5** | **39259** | **611.79** | **753.50** | **10095.63** | **1947.67** | **1722.83** | **622.00** |
| **Dyckia** | **ibiramensis** | **XC** | **camterr** | **5** | **18** | **336.32** | **141.40** | **11399.68** | **87.54** | **1538.98** | **18.60** |
| **Dyckia** | **leptostachya** | **XC** | **camterr** | **45** | **2968768** | **347.49** | **1083.25** | **8731.13** | **11375.26** | **1350.54** | **1059.25** |
| **Dyckia** | **limae** | **XC** | **camterr** | **8** | **10944** | **755.42** | **793.75** | **5726.21** | **2785.55** | **824.82** | **420.25** |
| **Dyckia** | **linearifolia** | **XC** | **camterr** | **10** | **18759** | **702.66** | **267.25** | **8759.23** | **1197.91** | **1281.37** | **190.08** |
| **Dyckia** | **macedoi** | **XC** | **camterr** | **9** | **40805** | **1057.60** | **326.00** | **9752.51** | **2985.59** | **1484.80** | **447.42** |
| **Dyckia** | **maracasensis** | **XC** | **camterr** | **3** | **49** | **944.94** | **3.92** | **5436.49** | **1345.78** | **751.50** | **62.25** |
| **Dyckia** | **maritima** | **XC** | **camterr** | **10** | **10728** | **134.33** | **717.30** | **12712.86** | **6453.45** | **1495.17** | **651.00** |
| **Dyckia** | **marnier-lapostollei** | **XC** | **camterr** | **6** | **44206** | **1061.02** | **471.25** | **10045.23** | **2012.81** | **1527.60** | **242.90** |
| **Dyckia** | **microcalyx** | **XC** | **camterr** | **13** | **74246** | **182.21** | **308.25** | **9371.02** | **4223.27** | **1473.13** | **556.50** |
| **Dyckia** | **minarum** | **XC** | **camterr** | **15** | **314702** | **835.81** | **1243.50** | **10166.85** | **8657.36** | **1510.48** | **945.50** |
| **Dyckia** | **niederleinii** | **XC** | **camterr** | **17** | **13763** | **185.68** | **464.75** | **10912.73** | **2194.60** | **1722.90** | **306.00** |
| **Dyckia** | **pernambucana** | **XC** | **camterr** | **4** | **63985** | **810.15** | **230.75** | **6137.46** | **1834.42** | **972.60** | **555.75** |
| **Dyckia** | **pulquinensis** | **XC** | **camterr** | **3** | **1373** | **1137.12** | **1419.30** | **8103.35** | **14425.05** | **1331.10** | **2491.30** |
| **Dyckia** | **pumila** | **XC** | **camterr** | **4** | **155745** | **646.55** | **586.00** | **9946.19** | **4017.18** | **1725.60** | **942.75** |
| **Dyckia** | **racemosa** | **XC** | **camterr** | **3** | **184** | **778.03** | **55.42** | **8321.05** | **1629.37** | **1421.83** | **166.00** |
| **Dyckia** | **ragonesei** | **XC** | **camterr** | **3** | **58458** | **152.92** | **174.00** | **7272.22** | **6455.11** | **1191.67** | **916.25** |
| **Dyckia** | **rariflora** | **XC** | **camterr** | **10** | **9226** | **914.62** | **503.25** | **9513.85** | **2424.84** | **1411.96** | **177.00** |
| **Dyckia** | **reitzii** | **XC** | **camterr** | **9** | **40980** | **976.37** | **410.00** | **13417.68** | **5517.48** | **1634.99** | **414.20** |
| **Dyckia** | **remotiflora** | **XC** | **camterr** | **9** | **201598** | **131.23** | **224.80** | **10170.02** | **2419.82** | **1423.22** | **643.75** |
| **Dyckia** | **saxatilis** | **XC** | **camterr** | **27** | **409151** | **1039.69** | **1117.25** | **9931.59** | **8009.81** | **1455.74** | **955.75** |
| **Dyckia** | **sordida** | **XC** | **camterr** | **7** | **5089** | **1110.26** | **371.25** | **9861.20** | **1801.71** | **1454.01** | **247.25** |
| **Dyckia** | **stenophylla** | **XC** | **camterr** | **8** | **612** | **675.73** | **1561.92** | **13794.13** | **10548.60** | **2268.54** | **1707.25** |
| **Dyckia** | **tenebrosa** | **XC** | **camterr** | **3** | **21** | **1207.28** | **125.65** | **10383.08** | **804.66** | **1516.17** | **43.00** |
| **Dyckia** | **tobatiensis** | **XC** | **camterr** | **5** | **1489** | **108.79** | **64.70** | **8840.00** | **2640.45** | **1370.51** | **238.80** |
| **Dyckia** | **trichostachya** | **XC** | **camterr** | **17** | **192337** | **601.83** | **1046.25** | **8567.30** | **3964.49** | **1294.50** | **576.08** |
| **Dyckia** | **tuberosa** | **XC** | **camterr** | **32** | **1096498** | **878.20** | **1140.00** | **9963.18** | **8456.87** | **1424.99** | **968.50** |
| **Dyckia** | **uleana** | **XC** | **camterr** | **4** | **124627** | **558.56** | **372.25** | **8180.43** | **502.03** | **1450.69** | **229.75** |
| **Dyckia** | **ursina** | **XC** | **camterr** | **3** | **4** | **755.83** | **0.25** | **8588.19** | **465.85** | **1355.42** | **64.25** |

**
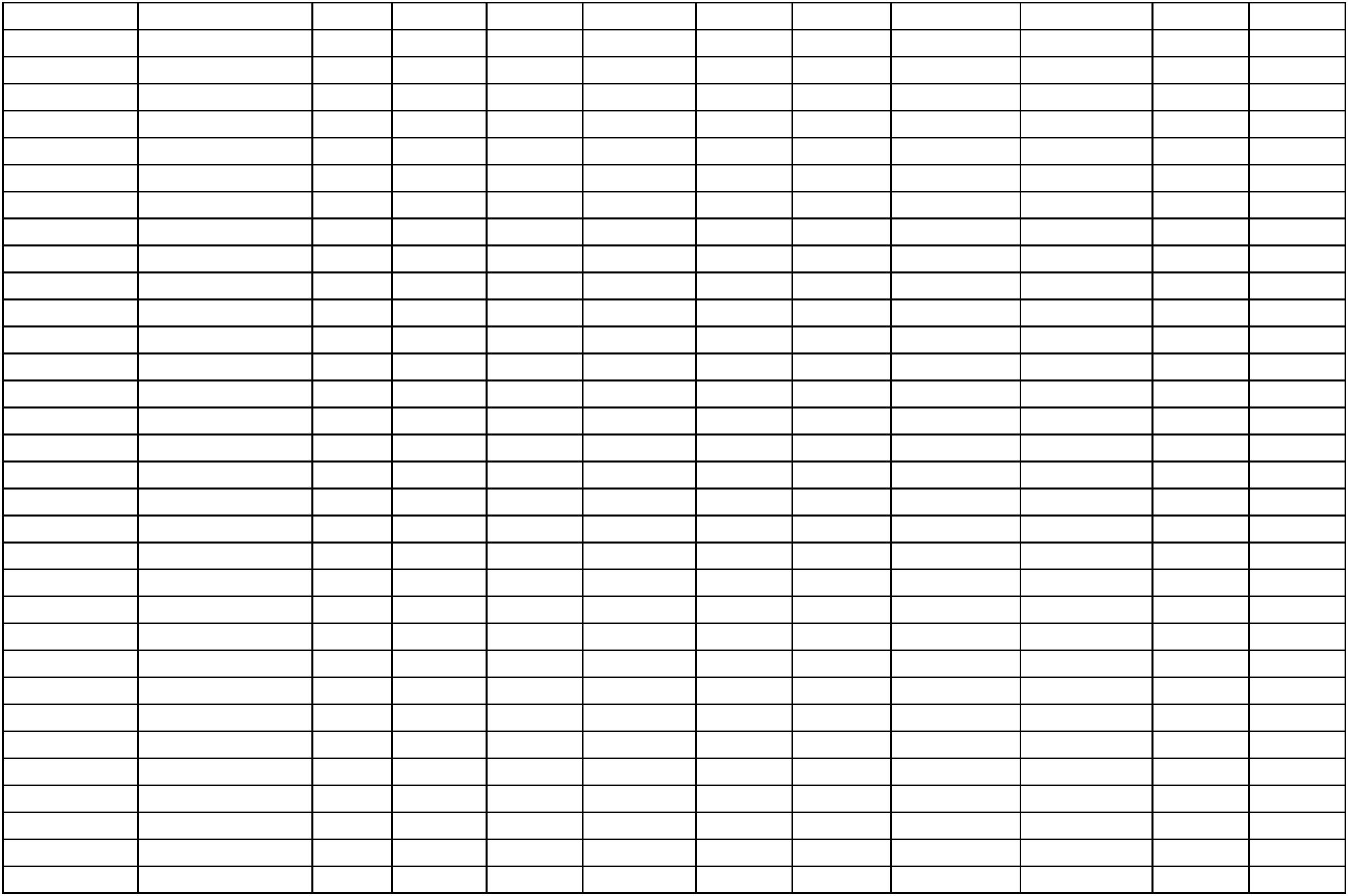
**

| **Dyckia** | **velascana** | **XC** | **camterr** | **9** | **126661** | **1836.53** | **3229.00** | **2184.87** | **2437.80** | **308.15** | **400.25** |
| --- | --- | --- | --- | --- | --- | --- | --- | --- | --- | --- | --- |
| **Dyckia** | **weddelliana** | **XC** | **camterr** | **22** | **834120** | **1542.20** | **2523.50** | **9060.68** | **12186.45** | **1446.57** | **2186.55** |
| **Encholirium** | **agavoides** | **XC** | **camterr** | **4** | **4** | **1128.19** | **154.25** | **9871.66** | **934.04** | **1488.13** | **25.50** |
| **Encholirium** | **biflorum** | **XC** | **camterr** | **3** | **22** | **1257.92** | **124.00** | **10397.27** | **213.03** | **1458.83** | **30.75** |
| **Encholirium** | **brachypodum** | **XC** | **camterr** | **15** | **37255** | **764.57** | **766.67** | **4741.39** | **2711.74** | **776.68** | **405.33** |
| **Encholirium** | **ctenophyllum** | **XC** | **camterr** | **5** | **77** | **965.05** | **514.75** | **9351.10** | **3473.74** | **1420.05** | **374.50** |
| **Encholirium** | **disjunctum** | **XC** | **camterr** | **3** | **57** | **761.58** | **479.50** | **7610.07** | **3741.55** | **1324.08** | **478.00** |
| **Encholirium** | **erectiflorum** | **XC** | **camterr** | **4** | **33973** | **296.88** | **510.67** | **6577.57** | **3828.23** | **1179.17** | **901.33** |
| **Encholirium** | **gracile** | **XC** | **camterr** | **6** | **3427** | **283.74** | **205.08** | **6813.21** | **1000.86** | **1104.08** | **115.00** |
| **Encholirium** | **heloisae** | **XC** | **camterr** | **8** | **3669** | **953.73** | **625.25** | **9692.12** | **2120.86** | **1471.95** | **281.00** |
| **Encholirium** | **horridum** | **XC** | **camterr** | **30** | **67600** | **296.20** | **740.25** | **7297.51** | **2697.39** | **1158.24** | **299.25** |
| **Encholirium** | **irwinii** | **XC** | **camterr** | **4** | **1906** | **823.25** | **180.50** | **6097.82** | **887.43** | **1024.63** | **215.50** |
| **Encholirium** | **longiflorum** | **XC** | **camterr** | **3** | **55** | **581.00** | **304.50** | **5282.51** | **367.10** | **962.67** | **10.00** |
| **Encholirium** | **luxor** | **XC** | **camterr** | **11** | **112390** | **801.11** | **613.00** | **8241.14** | **6290.37** | **1320.64** | **852.75** |
| **Encholirium** | **lymanianum** | **XC** | **camterr** | **4** | **734** | **318.75** | **440.25** | **8417.16** | **129.61** | **1424.00** | **91.50** |
| **Encholirium** | **magalhaensis** | **XC** | **camterr** | **4** | **66** | **1294.69** | **192.75** | **10248.54** | **1272.90** | **1441.50** | **100.50** |
| **Encholirium** | **maximum** | **XC** | **camterr** | **4** | **2289** | **750.81** | **513.25** | **4124.83** | **640.64** | **748.75** | **224.25** |
| **Encholirium** | **scrutor** | **XC** | **camterr** | **4** | **179** | **1075.94** | **256.00** | **9675.79** | **819.84** | **1419.63** | **44.75** |
| **Encholirium** | **spectabile** | **XC** | **camterr** | **83** | **1145429** | **515.84** | **1218.50** | **4640.60** | **10275.64** | **745.91** | **1777.25** |
| **Encholirium** | **subsecundum** | **XC** | **camterr** | **13** | **70724** | **945.49** | **1011.00** | **9286.64** | **6870.96** | **1395.72** | **1083.42** |
| **Fascicularia** | **bicolor** | **C3EDB** | **c3terr** | **11** | **54595** | **337.18** | **675.50** | **23607.15** | **27358.62** | **1901.89** | **1470.40** |
| **Fernseea** | **itatiaiae** | **C3EDB** | **c3terr** | **10** | **5869** | **1419.62** | **2164.50** | **14413.26** | **11370.78** | **1821.65** | **1393.75** |
| **Fosterella** | **albicans** | **PF** | **c3mes** | **23** | **179056** | **1490.06** | **1888.75** | **6129.65** | **9449.48** | **957.46** | **1543.17** |
| **Fosterella** | **caulescens** | **PF** | **c3mes** | **4** | **29** | **1299.23** | **451.67** | **11389.15** | **1139.66** | **1618.63** | **243.33** |
| **Fosterella** | **chaparensis** | **PF** | **c3mes** | **7** | **4878** | **1601.87** | **2132.75** | **12794.82** | **16078.65** | **2117.30** | **2927.50** |
| **Fosterella** | **christophii** | **PF** | **c3mes** | **7** | **83088** | **763.69** | **798.75** | **7305.37** | **3331.30** | **1171.13** | **445.75** |
| **Fosterella** | **cotacajensis** | **PF** | **c3mes** | **6** | **1793** | **2151.38** | **1165.75** | **8093.64** | **5134.54** | **1298.16** | **866.75** |
| **Fosterella** | **gracilis** | **PF** | **c3mes** | **9** | **61184** | **979.45** | **1426.13** | **9545.05** | **6368.45** | **1526.41** | **1099.83** |
| **Fosterella** | **graminea** | **PF** | **c3mes** | **5** | **6339** | **1883.80** | **3075.00** | **8715.19** | **5447.18** | **1246.00** | **1012.75** |
| **Fosterella** | **hatschbachii** | **PF** | **c3mes** | **15** | **53137** | **353.73** | **518.75** | **8459.56** | **1291.90** | **1437.84** | **228.75** |
| **Fosterella** | **heterophylla** | **PF** | **c3mes** | **3** | **1065** | **1506.42** | **1216.25** | **8690.41** | **2726.28** | **1359.00** | **485.25** |
| **Fosterella** | **kroemeri** | **PF** | **c3mes** | **3** | **146** | **1464.00** | **315.50** | **8711.72** | **2260.77** | **1379.17** | **343.75** |
| **Fosterella** | **micrantha** | **PF** | **c3mes** | **31** | **397256** | **487.81** | **1834.00** | **13612.43** | **18101.86** | **2287.85** | **3015.83** |

**
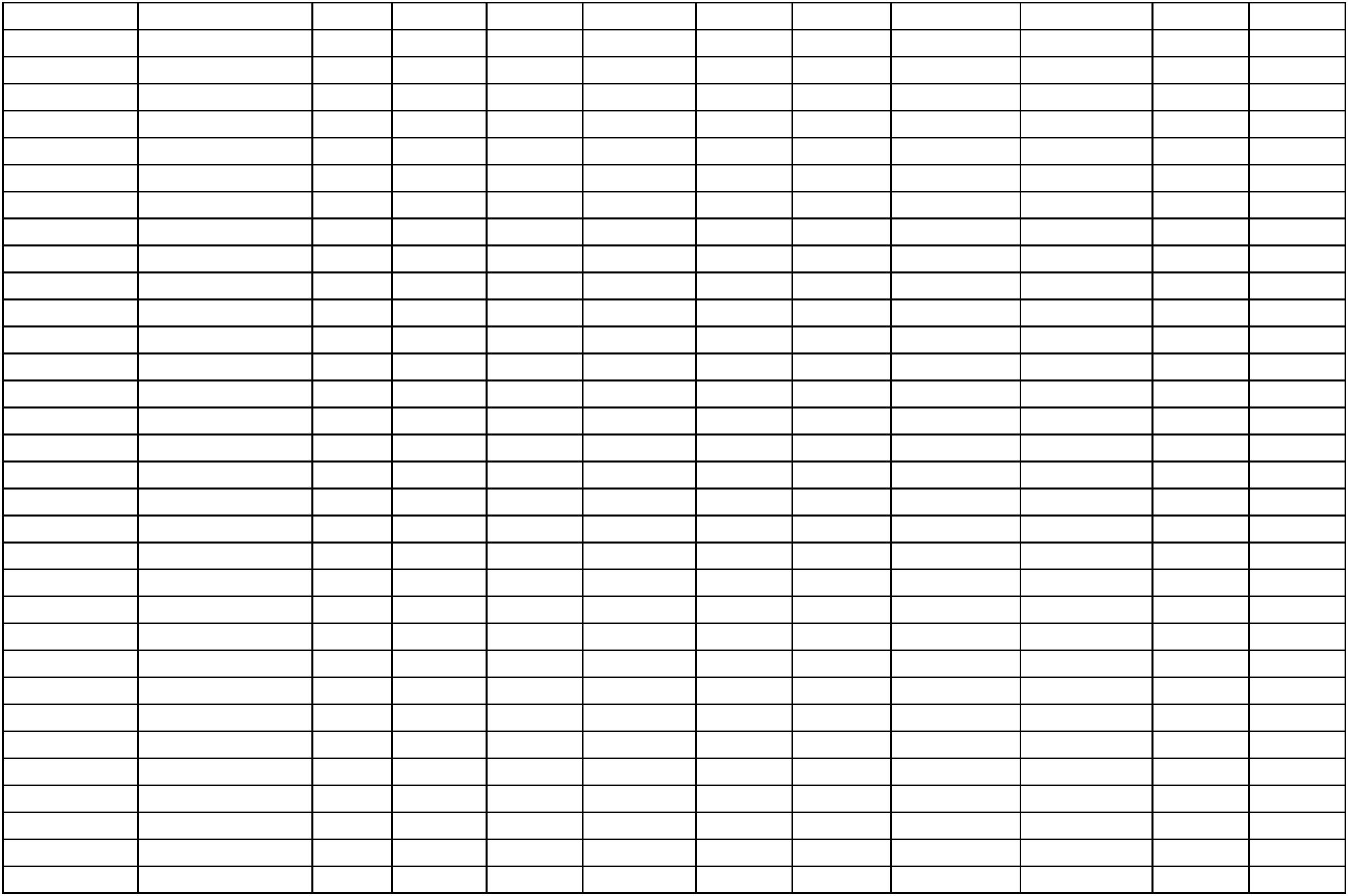
**

| **Fosterella** | **nicoliana** | **PF** | **c3mes** | **4** | **22363** | **653.94** | **1832.00** | **11433.40** | **8395.85** | **2015.46** | **1543.50** |
| --- | --- | --- | --- | --- | --- | --- | --- | --- | --- | --- | --- |
| **Fosterella** | **pearcei** | **PF** | **c3mes** | **70** | **1994894** | **1588.11** | **4262.80** | **17419.83** | **23623.94** | **2587.00** | **3645.50** |
| **Fosterella** | **penduliflora** | **PF** | **c3mes** | **31** | **808508** | **1181.15** | **3157.50** | **6349.54** | **9271.01** | **1029.77** | **1326.50** |
| **Fosterella** | **petiolata** | **PF** | **c3mes** | **11** | **23440** | **2018.45** | **1361.00** | **7841.75** | **3239.59** | **1155.98** | **420.50** |
| **Fosterella** | **robertreadii** | **PF** | **c3mes** | **4** | **79998** | **1407.98** | **1995.67** | **7619.81** | **2649.42** | **1332.15** | **665.67** |
| **Fosterella** | **rojasii** | **PF** | **c3mes** | **7** | **22221** | **443.98** | **1054.50** | **8713.55** | **554.51** | **1408.58** | **45.25** |
| **Fosterella** | **rusbyi** | **PF** | **c3mes** | **25** | **90009** | **1549.28** | **2358.30** | **9475.25** | **11377.40** | **1460.73** | **2040.80** |
| **Fosterella** | **schidosperma** | **PF** | **c3mes** | **7** | **708690** | **1642.51** | **3912.25** | **11021.53** | **10006.40** | **1827.40** | **1976.50** |
| **Fosterella** | **vasquezii** | **PF** | **c3mes** | **5** | **937** | **489.01** | **516.30** | **8182.47** | **1040.32** | **1463.04** | **107.00** |
| **Fosterella** | **villosula** | **PF** | **c3mes** | **12** | **31463** | **1223.63** | **1151.25** | **12897.19** | **11072.90** | **2036.94** | **2115.08** |
| **Fosterella** | **weberbaueri** | **PF** | **c3mes** | **11** | **117241** | **1371.01** | **1846.83** | **10909.82** | **14746.29** | **1776.74** | **2556.50** |
| **Fosterella** | **weddelliana** | **PF** | **c3mes** | **13** | **16840** | **1652.71** | **2426.50** | **8656.18** | **10655.97** | **1400.44** | **1869.75** |
| **Fosterella** | **windischii** | **PF** | **c3mes** | **4** | **242577** | **839.13** | **2193.00** | **9053.58** | **6394.58** | **1573.75** | **1272.75** |
| **Fosterella** | **yuvinkae** | **PF** | **c3mes** | **5** | **11067** | **398.29** | **448.25** | **8450.85** | **12031.61** | **1479.21** | **1954.05** |
| **Greigia** | **alborosea** | **C3EDB** | **c3terr** | **5** | **1258** | **1312.03** | **1476.50** | **9669.78** | **723.02** | **1378.82** | **523.50** |
| **Greigia** | **columbiana** | **C3EDB** | **c3terr** | **23** | **457236** | **2523.63** | **3950.25** | **22810.67** | **20889.71** | **2474.31** | **2269.75** |
| **Greigia** | **danielii** | **C3EDB** | **c3terr** | **24** | **39262** | **2685.79** | **2385.25** | **14645.95** | **15418.83** | **1684.20** | **1712.25** |
| **Greigia** | **kessleri** | **C3EDB** | **c3terr** | **3** | **8521** | **2796.06** | **1067.33** | **7242.34** | **3287.96** | **990.56** | **313.00** |
| **Greigia** | **leymebambana** | **C3EDB** | **c3terr** | **4** | **17643** | **2780.44** | **1223.50** | **8399.18** | **3296.90** | **1231.50** | **174.75** |
| **Greigia** | **mulfordii** | **C3EDB** | **c3terr** | **24** | **308001** | **2876.88** | **3523.50** | **12125.52** | **18036.76** | **1379.66** | **2053.50** |
| **Greigia** | **nubigena** | **C3EDB** | **c3terr** | **5** | **1102891** | **887.25** | **2076.75** | **7971.60** | **4160.61** | **1307.75** | **883.50** |
| **Greigia** | **oaxacana** | **C3EDB** | **c3terr** | **6** | **51985** | **1928.31** | **356.50** | **14879.04** | **4990.13** | **1997.87** | **598.20** |
| **Greigia** | **rohwederi** | **C3EDB** | **c3terr** | **3** | **3199** | **1373.83** | **1807.25** | **13857.12** | **4834.75** | **1905.25** | **105.00** |
| **Greigia** | **sanctae-martae** | **C3EDB** | **c3terr** | **3** | **43085** | **2338.64** | **1386.75** | **16252.97** | **8066.08** | **1971.89** | **1461.00** |
| **Greigia** | **sodiroana** | **C3EDB** | **c3terr** | **10** | **41583** | **2398.73** | **3202.25** | **10910.91** | **26915.47** | **1548.33** | **4828.00** |
| **Greigia** | **sphacelata** | **C3EDB** | **c3terr** | **5** | **58403** | **536.44** | **1561.05** | **18425.94** | **22696.37** | **1557.63** | **1084.00** |
| **Greigia** | **stenolepis** | **C3EDB** | **c3terr** | **7** | **133853** | **3037.01** | **1043.25** | **12875.17** | **12260.80** | **1345.77** | **1043.67** |
| **Greigia** | **steyermarkii** | **C3EDB** | **c3terr** | **15** | **149735** | **843.37** | **2052.58** | **15230.02** | **8521.71** | **2391.50** | **1407.33** |
| **Greigia** | **sylvicola** | **C3EDB** | **c3terr** | **50** | **21939** | **1989.76** | **2817.15** | **24573.60** | **20359.48** | **2904.99** | **2269.50** |
| **Greigia** | **van-hyningii** | **C3EDB** | **c3terr** | **6** | **3481** | **1969.17** | **954.75** | **11295.78** | **9801.19** | **1618.24** | **1417.00** |
| **Greigia** | **vilcabambae** | **C3EDB** | **c3terr** | **4** | **2501** | **1837.63** | **2365.75** | **7552.25** | **2957.58** | **1269.38** | **668.00** |
| **Greigia** | **vulcanica** | **C3EDB** | **c3terr** | **14** | **101905** | **3009.34** | **3370.00** | **15235.37** | **19144.40** | **1664.80** | **3035.50** |
| **Hechtia** | **bracteata** | **Hec** | **camterr** | **7** | **25666** | **2183.04** | **1174.25** | **4406.83** | **10501.34** | **645.94** | **1484.75** |

**
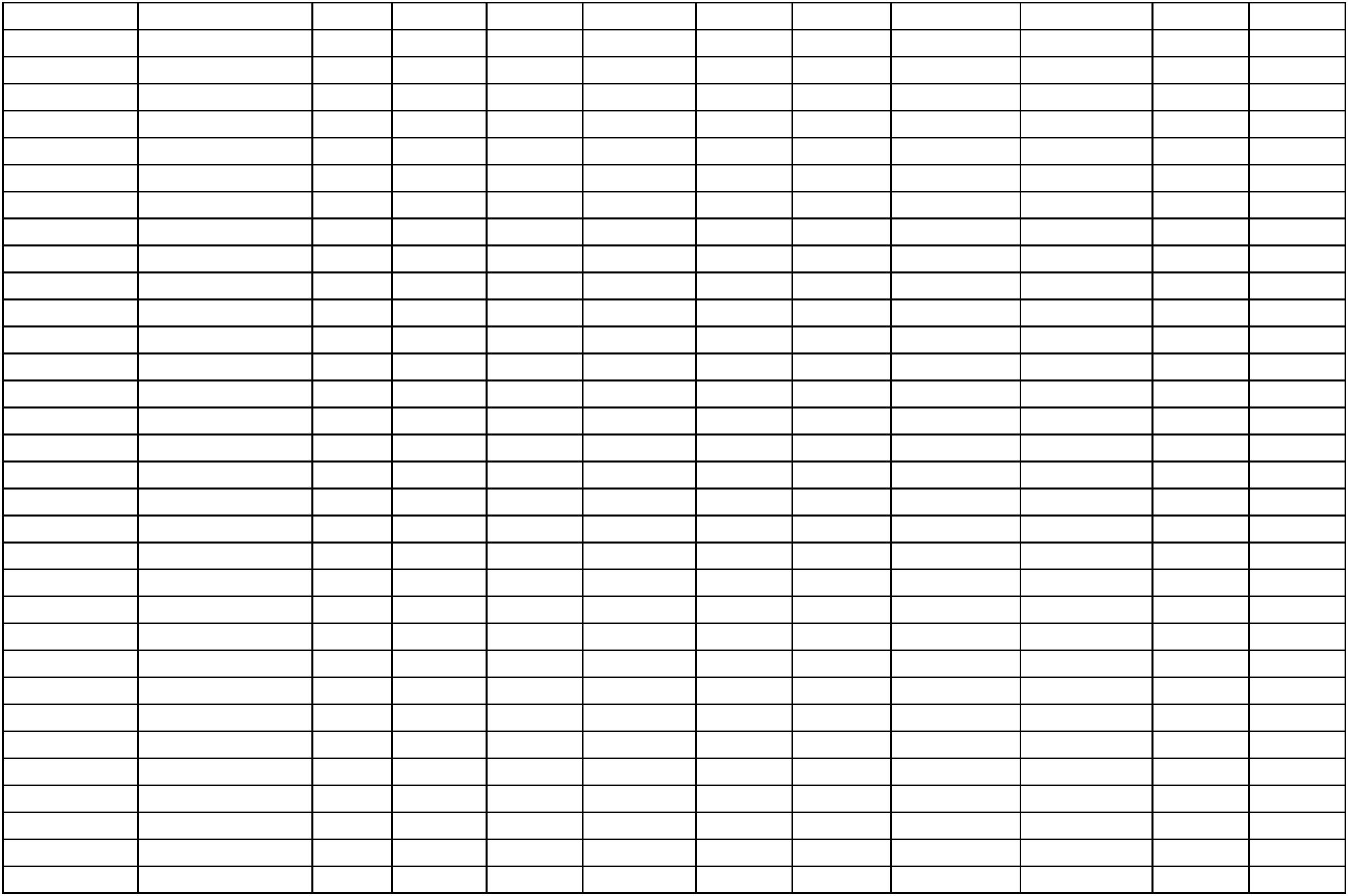
**

| **Hechtia** | **caudata** | **Hec** | **camterr** | **7** | **225** | **290.19** | **577.50** | **5253.51** | **1405.80** | **887.86** | **155.17** |
| --- | --- | --- | --- | --- | --- | --- | --- | --- | --- | --- | --- |
| **Hechtia** | **confusa** | **Hec** | **camterr** | **3** | **5628** | **2138.17** | **625.50** | **4589.92** | **2368.23** | **699.75** | **305.00** |
| **Hechtia** | **conzattiana** | **Hec** | **camterr** | **6** | **551** | **1245.83** | **1074.00** | **3669.57** | **5431.68** | **622.28** | **884.67** |
| **Hechtia** | **fragilis** | **Hec** | **camterr** | **5** | **193** | **1034.22** | **837.50** | **4789.97** | **4230.18** | **817.63** | **608.92** |
| **Hechtia** | **galeottii** | **Hec** | **camterr** | **5** | **511** | **1053.17** | **740.75** | **3941.64** | **3324.40** | **647.30** | **360.08** |
| **Hechtia** | **glabra** | **Hec** | **camterr** | **5** | **22976** | **385.90** | **1062.50** | **8004.68** | **5548.22** | **1276.60** | **828.00** |
| **Hechtia** | **glomerata** | **Hec** | **camterr** | **39** | **622887** | **1229.59** | **2494.25** | **4937.11** | **9505.88** | **813.11** | **1625.25** |
| **Hechtia** | **guatemalensis** | **Hec** | **camterr** | **44** | **81909** | **871.91** | **1103.25** | **8311.27** | **7062.47** | **1330.27** | **1218.42** |
| **Hechtia** | **jaliscana** | **Hec** | **camterr** | **5** | **9410** | **797.67** | **1605.17** | **4925.82** | **1997.28** | **905.68** | **261.58** |
| **Hechtia** | **laevis** | **Hec** | **camterr** | **5** | **345** | **339.50** | **428.50** | **4693.27** | **583.57** | **857.00** | **106.00** |
| **Hechtia** | **liebmannii** | **Hec** | **camterr** | **5** | **1014** | **1769.75** | **392.50** | **3133.13** | **1201.53** | **530.85** | **233.25** |
| **Hechtia** | **lundelliorum** | **Hec** | **camterr** | **5** | **5108** | **885.78** | **1395.50** | **9846.29** | **7467.58** | **1574.52** | **1034.40** |
| **Hechtia** | **lyman-smithii** | **Hec** | **camterr** | **5** | **103** | **848.50** | **368.50** | **2283.00** | **1066.73** | **455.15** | **232.25** |
| **Hechtia** | **montana** | **Hec** | **camterr** | **23** | **467492** | **632.63** | **2173.25** | **2517.03** | **3329.94** | **435.15** | **599.65** |
| **Hechtia** | **pedicellata** | **Hec** | **camterr** | **4** | **20** | **1529.31** | **126.25** | **5237.02** | **310.66** | **918.81** | **71.25** |
| **Hechtia** | **podantha** | **Hec** | **camterr** | **41** | **239284** | **1572.22** | **2764.75** | **3487.48** | **7291.32** | **579.41** | **1110.83** |
| **Hechtia** | **pringlei** | **Hec** | **camterr** | **7** | **1408** | **1398.77** | **1284.50** | **3792.97** | **2175.77** | **636.74** | **218.17** |
| **Hechtia** | **purpusii** | **Hec** | **camterr** | **3** | **273** | **1338.58** | **1106.50** | **8233.44** | **9337.93** | **1225.67** | **1292.50** |
| **Hechtia** | **rosea** | **Hec** | **camterr** | **22** | **11154** | **443.13** | **1572.67** | **5523.43** | **6819.63** | **934.30** | **1214.00** |
| **Hechtia** | **schottii** | **Hec** | **camterr** | **15** | **258986** | **216.19** | **972.25** | **6988.88** | **7693.11** | **1186.79** | **1009.50** |
| **Hechtia** | **sphaeroblasta** | **Hec** | **camterr** | **4** | **6575** | **1688.88** | **777.50** | **4044.36** | **2709.14** | **717.21** | **500.25** |
| **Hechtia** | **stenopetala** | **Hec** | **camterr** | **7** | **20613** | **621.83** | **1277.00** | **8189.92** | **12618.96** | **1258.74** | **1769.42** |
| **Hechtia** | **subalata** | **Hec** | **camterr** | **6** | **58108** | **957.69** | **1727.50** | **5096.35** | **5663.52** | **908.60** | **1048.42** |
| **Hechtia** | **texensis** | **Hec** | **camterr** | **17** | **212454** | **1081.69** | **1779.75** | **2703.68** | **5748.66** | **444.03** | **827.58** |
| **Hechtia** | **tillandsioides** | **Hec** | **camterr** | **5** | **7207** | **1037.33** | **1338.08** | **8975.30** | **5307.42** | **1428.10** | **899.25** |
| **Lindmania** | **arachnoidea** | **BL** | **c3mes** | **10** | **646** | **1267.33** | **2002.42** | **19907.46** | **14045.13** | **2958.95** | **1470.00** |
| **Lindmania** | **brachyphylla** | **BL** | **c3mes** | **6** | **6988** | **1370.67** | **1400.75** | **17281.57** | **4209.33** | **2554.01** | **1237.25** |
| **Lindmania** | **cylindrostachya** | **BL** | **c3mes** | **3** | **58** | **1271.25** | **326.00** | **19928.51** | **3482.00** | **3041.83** | **508.75** |
| **Lindmania** | **geniculata** | **BL** | **c3mes** | **7** | **42828** | **1256.11** | **2058.00** | **17057.32** | **9640.63** | **2575.94** | **1379.50** |
| **Lindmania** | **guianensis** | **BL** | **c3mes** | **7** | **3994** | **686.00** | **1308.33** | **16161.85** | **6365.66** | **2434.30** | **1103.67** |
| **Lindmania** | **holstii** | **BL** | **c3mes** | **4** | **5** | **1183.48** | **489.08** | **15606.23** | **357.44** | **2343.63** | **256.50** |
| **Lindmania** | **marahuacae** | **BL** | **c3mes** | **6** | **493** | **1423.90** | **2002.42** | **21094.49** | **9009.57** | **3082.82** | **508.00** |
| **Lindmania** | **navioides** | **BL** | **c3mes** | **3** | **3106** | **894.85** | **1321.00** | **16068.73** | **8425.12** | **2517.25** | **1284.00** |

**
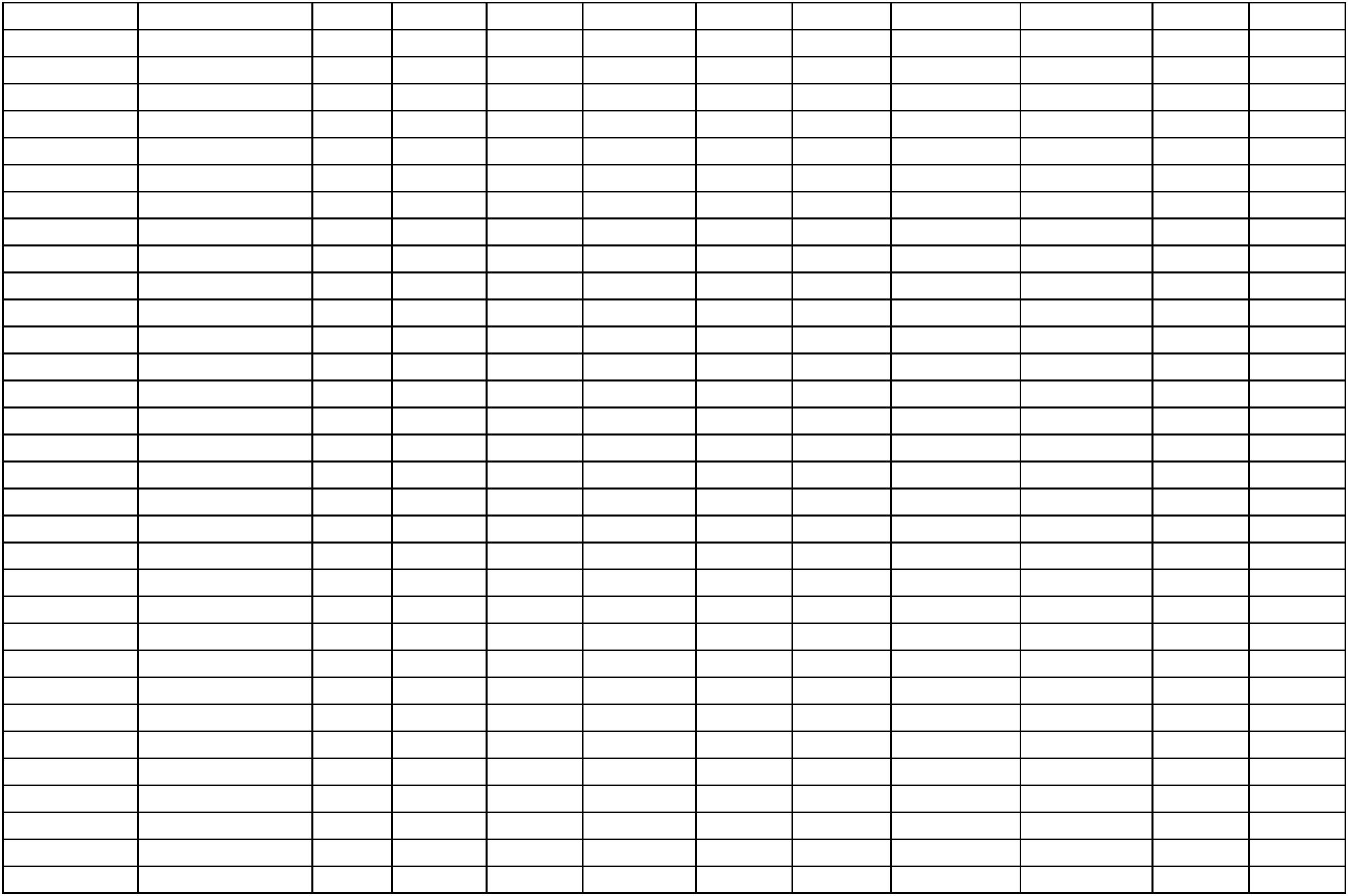
**

| **Lindmania** | **nubigena** | **BL** | **c3mes** | **3** | **839** | **1784.50** | **343.00** | **23876.13** | **2438.13** | **3348.75** | **299.75** |
| --- | --- | --- | --- | --- | --- | --- | --- | --- | --- | --- | --- |
| **Lindmania** | **serrulata** | **BL** | **c3mes** | **7** | **44673** | **1032.71** | **2107.58** | **17831.94** | **3162.02** | **2801.85** | **1171.75** |
| **Lindmania** | **steyermarkii** | **BL** | **c3mes** | **3** | **3** | **1691.22** | **1595.67** | **18538.19** | **281.01** | **2599.22** | **699.00** |
| **Lindmania** | **subsimplex** | **BL** | **c3mes** | **6** | **2848** | **1391.94** | **2120.75** | **17313.06** | **8797.30** | **2508.98** | **1284.00** |
| **Lindmania** | **thyrsoidea** | **BL** | **c3mes** | **3** | **3** | **1196.25** | **714.75** | **18374.21** | **2478.28** | **2897.75** | **106.92** |
| **Lindmania** | **wurdackii** | **BL** | **c3mes** | **6** | **67642** | **1197.28** | **2008.08** | **18473.80** | **5721.86** | **2804.03** | **948.42** |
| **Navia** | **acaulis** | **Nav** | **c3mes** | **32** | **136155** | **224.45** | **289.67** | **16423.67** | **5001.20** | **2727.49** | **840.25** |
| **Navia** | **affinis** | **Nav** | **c3mes** | **3** | **623** | **1700.31** | **469.17** | **23449.89** | **3295.27** | **3335.42** | **249.75** |
| **Navia** | **aliciae** | **Nav** | **c3mes** | **5** | **68** | **703.97** | **926.50** | **17577.62** | **9964.77** | **2937.04** | **1432.50** |
| **Navia** | **angustifolia** | **Nav** | **c3mes** | **4** | **8471** | **1564.88** | **518.42** | **20806.83** | **7081.93** | **3042.40** | **1239.50** |
| **Navia** | **caulescens** | **Nav** | **c3mes** | **15** | **74499** | **227.72** | **364.80** | **18323.17** | **6930.15** | **2952.35** | **997.70** |
| **Navia** | **connata** | **Nav** | **c3mes** | **3** | **1349** | **301.78** | **296.85** | **18770.97** | **9671.58** | **3236.50** | **1619.00** |
| **Navia** | **crispa** | **Nav** | **c3mes** | **3** | **3499** | **144.72** | **164.42** | **16154.69** | **3645.87** | **2844.58** | **137.75** |
| **Navia** | **duidae** | **Nav** | **c3mes** | **20** | **601723** | **990.88** | **1687.67** | **17962.56** | **12740.61** | **2759.84** | **1684.25** |
| **Navia** | **jauana** | **Nav** | **c3mes** | **4** | **24** | **1021.88** | **1088.00** | **21243.76** | **2178.96** | **3298.42** | **95.00** |
| **Navia** | **linearis** | **Nav** | **c3mes** | **3** | **8** | **1446.92** | **584.25** | **21160.14** | **2478.72** | **3102.83** | **130.50** |
| **Navia** | **luzuloides** | **Nav** | **c3mes** | **5** | **35** | **1627.25** | **296.25** | **22306.60** | **1131.32** | **3228.45** | **14.50** |
| **Navia** | **maguirei** | **Nav** | **c3mes** | **9** | **204** | **418.54** | **460.33** | **17492.58** | **3895.43** | **2744.04** | **475.92** |
| **Navia** | **nubicola** | **Nav** | **c3mes** | **11** | **13656** | **959.36** | **1776.67** | **19635.17** | **8980.08** | **3144.08** | **512.67** |
| **Navia** | **octopoides** | **Nav** | **c3mes** | **3** | **12** | **672.47** | **714.25** | **18384.88** | **2602.19** | **3139.75** | **76.75** |
| **Navia** | **ovoidea** | **Nav** | **c3mes** | **3** | **38** | **694.67** | **337.00** | **19185.23** | **655.95** | **3095.08** | **207.00** |
| **Navia** | **pauciflora** | **Nav** | **c3mes** | **3** | **399** | **1552.36** | **917.08** | **21274.17** | **2918.24** | **3194.36** | **85.08** |
| **Navia** | **pulvinata** | **Nav** | **c3mes** | **4** | **809** | **577.81** | **911.50** | **18460.75** | **4695.85** | **3148.13** | **644.25** |
| **Navia** | **scirpiflora** | **Nav** | **c3mes** | **3** | **16** | **1054.50** | **1088.00** | **21362.98** | **2178.96** | **3296.06** | **95.00** |
| **Navia** | **subpetiolata** | **Nav** | **c3mes** | **3** | **1** | **1552.67** | **579.25** | **22040.69** | **2088.62** | **3204.67** | **88.75** |
| **Navia** | **terramarae** | **Nav** | **c3mes** | **3** | **30** | **771.58** | **322.08** | **18568.89** | **1930.84** | **3082.44** | **122.67** |
| **Neoglaziovia** | **variegata** | **CAMEDB** | **camterr** | **139** | **911464** | **518.23** | **1269.33** | **4391.50** | **11296.34** | **702.09** | **1504.00** |
| **Ochagavia** | **carnea** | **C3EDB** | **c3terr** | **8** | **24332** | **881.64** | **1441.75** | **10003.43** | **7026.09** | **1163.46** | **669.50** |
| **Ochagavia** | **litoralis** | **C3EDB** | **c3terr** | **4** | **2269** | **146.27** | **318.92** | **9981.86** | **21779.51** | **908.35** | **1736.00** |
| **Orthophytum** | **albopictum** | **CAMEDB** | **camterr** | **13** | **1774** | **1099.46** | **298.42** | **5925.25** | **1177.29** | **915.51** | **188.27** |
| **Orthophytum** | **amoenum** | **CAMEDB** | **camterr** | **11** | **2439** | **858.39** | **508.42** | **5707.89** | **1522.76** | **914.27** | **287.25** |
| **Orthophytum** | **boudetianum** | **CAMEDB** | **camterr** | **5** | **33** | **728.57** | **278.58** | **8548.26** | **1255.64** | **1280.00** | **70.50** |
| **Orthophytum** | **burle-marxii** | **CAMEDB** | **camterr** | **21** | **7809** | **989.35** | **786.67** | **5866.63** | **1975.43** | **914.41** | **324.17** |

**
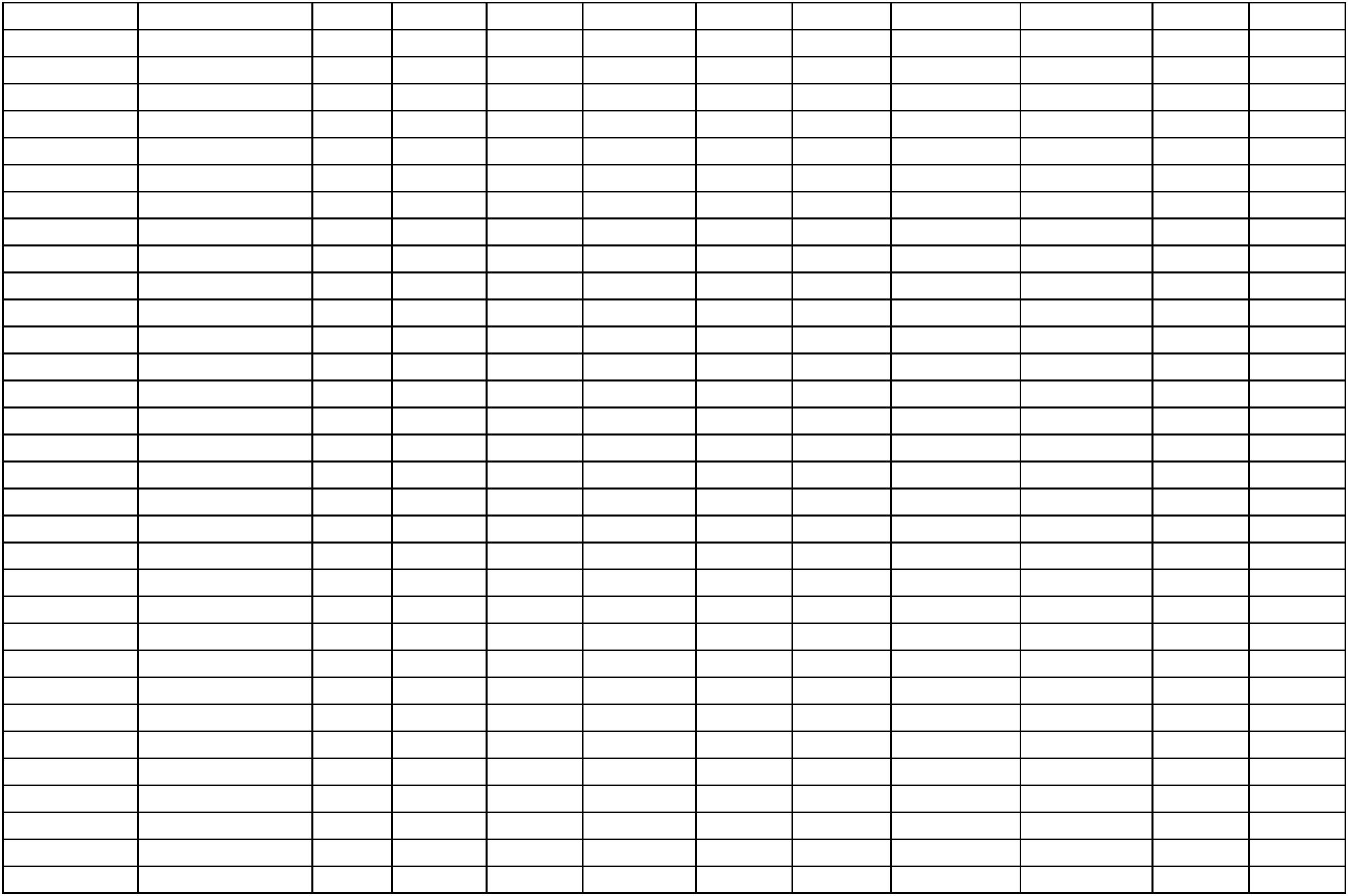
**

| **Orthophytum** | **compactum** | **CAMEDB** | **camterr** | **4** | **6074** | **321.73** | **452.08** | **6386.50** | **1483.58** | **1041.21** | **163.58** |
| --- | --- | --- | --- | --- | --- | --- | --- | --- | --- | --- | --- |
| **Orthophytum** | **diamantinense** | **CAMEDB** | **camterr** | **4** | **1387** | **1056.06** | **194.50** | **8990.40** | **3746.97** | **1346.02** | **446.25** |
| **Orthophytum** | **disjunctum** | **CAMEDB** | **camterr** | **41** | **249179** | **674.40** | **770.00** | **4887.09** | **5675.87** | **725.80** | **804.75** |
| **Orthophytum** | **foliosum** | **CAMEDB** | **camterr** | **16** | **115495** | **284.33** | **996.50** | **7460.55** | **4650.90** | **1158.27** | **747.25** |
| **Orthophytum** | **harleyi** | **CAMEDB** | **camterr** | **4** | **14** | **768.65** | **186.75** | **4856.63** | **932.12** | **815.19** | **96.25** |
| **Orthophytum** | **hatschbachii** | **CAMEDB** | **camterr** | **24** | **816** | **1222.86** | **663.33** | **6128.14** | **2017.58** | **922.31** | **263.33** |
| **Orthophytum** | **horridum** | **CAMEDB** | **camterr** | **7** | **2256** | **627.43** | **469.00** | **5625.91** | **1830.42** | **907.75** | **176.00** |
| **Orthophytum** | **jabrense** | **CAMEDB** | **camterr** | **3** | **61** | **436.16** | **144.67** | **5738.80** | **1056.88** | **890.48** | **155.73** |
| **Orthophytum** | **lemei** | **CAMEDB** | **camterr** | **6** | **3675** | **942.64** | **471.50** | **5121.39** | **2014.10** | **811.51** | **289.17** |
| **Orthophytum** | **leprosum** | **CAMEDB** | **camterr** | **5** | **22350** | **456.60** | **761.25** | **5090.81** | **1560.78** | **835.00** | **134.00** |
| **Orthophytum** | **magalhaesii** | **CAMEDB** | **camterr** | **13** | **5648** | **231.90** | **351.00** | **6963.87** | **688.62** | **1112.97** | **119.25** |
| **Orthophytum** | **maracasense** | **CAMEDB** | **camterr** | **26** | **114343** | **509.64** | **1320.25** | **4664.13** | **3269.00** | **726.27** | **484.75** |
| **Orthophytum** | **mello-barretoi** | **CAMEDB** | **camterr** | **6** | **5034** | **1018.56** | **619.50** | **9060.45** | **4783.41** | **1334.25** | **506.50** |
| **Orthophytum** | **navioides** | **CAMEDB** | **camterr** | **14** | **8244** | **947.59** | **923.00** | **5870.65** | **1453.88** | **928.12** | **273.40** |
| **Orthophytum** | **ophiuroides** | **CAMEDB** | **camterr** | **3** | **7** | **586.39** | **309.17** | **6387.26** | **1078.67** | **1072.19** | **189.25** |
| **Orthophytum** | **piranianum** | **CAMEDB** | **camterr** | **8** | **545** | **808.01** | **347.75** | **5820.41** | **741.43** | **963.17** | **65.25** |
| **Orthophytum** | **pseudovagans** | **CAMEDB** | **camterr** | **7** | **11** | **209.38** | **73.67** | **7250.61** | **26.83** | **1155.21** | **13.50** |
| **Orthophytum** | **riocontense** | **CAMEDB** | **camterr** | **4** | **1146** | **1120.69** | **383.75** | **5926.49** | **1477.29** | **918.50** | **194.75** |
| **Orthophytum** | **rubiginosum** | **CAMEDB** | **camterr** | **4** | **8** | **200.42** | **8.25** | **6931.96** | **54.62** | **1105.27** | **1.50** |
| **Orthophytum** | **saxicola** | **CAMEDB** | **camterr** | **27** | **182019** | **357.62** | **921.17** | **5112.07** | **10534.51** | **762.78** | **1322.50** |
| **Orthophytum** | **schulzianum** | **CAMEDB** | **camterr** | **6** | **1157** | **1061.63** | **475.00** | **9270.97** | **2449.29** | **1375.42** | **223.50** |
| **Orthophytum** | **triunfense** | **CAMEDB** | **camterr** | **3** | **4** | **850.44** | **94.67** | **6301.11** | **706.01** | **925.56** | **67.00** |
| **Orthophytum** | **ulei** | **CAMEDB** | **camterr** | **3** | **1** | **954.08** | **82.75** | **5975.24** | **54.61** | **942.67** | **19.00** |
| **Orthophytum** | **zanonii** | **CAMEDB** | **camterr** | **3** | **1** | **313.42** | **173.75** | **7505.14** | **301.29** | **1192.25** | **36.75** |
| **Pitcairnia** | **abyssicola** | **PF** | **c3mes** | **3** | **15** | **530.81** | **750.42** | **8130.27** | **1785.83** | **1201.25** | **297.75** |
| **Pitcairnia** | **aequatorialis** | **PF** | **c3mes** | **21** | **90188** | **1926.84** | **2704.00** | **8596.02** | **23625.19** | **1235.09** | **3559.83** |
| **Pitcairnia** | **alata** | **PF** | **c3mes** | **6** | **4149** | **2131.50** | **1246.33** | **11575.94** | **10407.85** | **1691.39** | **1821.75** |
| **Pitcairnia** | **albiflos** | **PF** | **c3mes** | **3** | **2** | **111.50** | **93.00** | **10166.38** | **417.48** | **1353.67** | **85.25** |
| **Pitcairnia** | **alborubra** | **PF** | **c3mes** | **3** | **8487** | **1334.17** | **832.25** | **20345.70** | **6326.13** | **3084.83** | **390.50** |
| **Pitcairnia** | **alexanderi** | **PF** | **c3mes** | **5** | **6215** | **913.15** | **749.50** | **16431.63** | **1564.08** | **2629.72** | **280.75** |
| **Pitcairnia** | **amblyosperma** | **PF** | **c3mes** | **4** | **6305** | **1061.27** | **2309.17** | **11437.02** | **14536.38** | **1795.85** | **2218.17** |
| **Pitcairnia** | **amboroensis** | **PF** | **c3mes** | **5** | **592** | **523.02** | **483.75** | **9119.00** | **2139.29** | **1481.65** | **387.50** |
| **Pitcairnia** | **angustifolia** | **PF** | **c3mes** | **39** | **110957** | **273.86** | **812.25** | **14063.30** | **16803.03** | **2048.42** | **2197.25** |

**
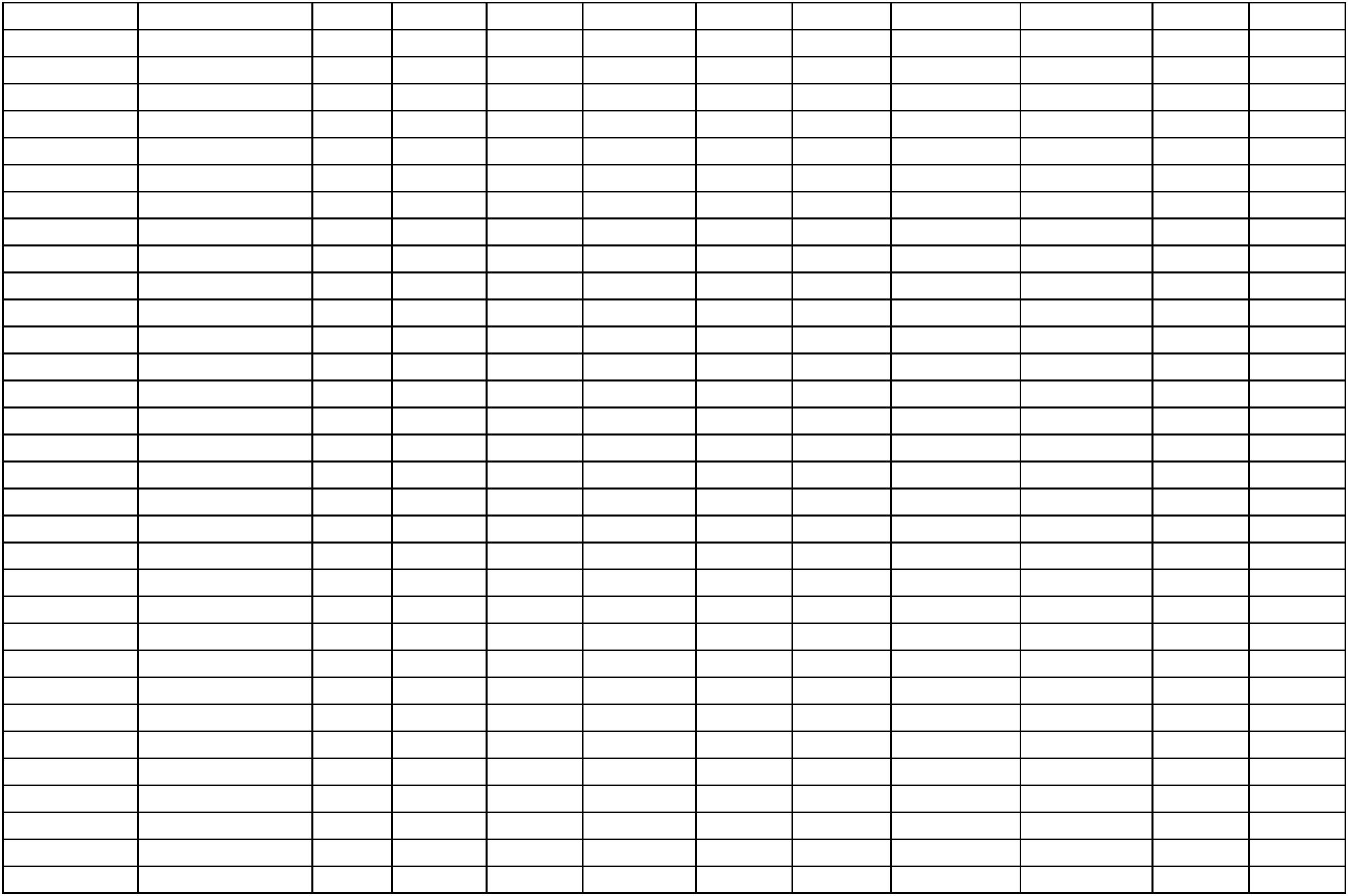
**

| **Pitcairnia** | **aphelandriflora** | **PF** | **c3mes** | **40** | **1688797** | **750.40** | **1237.25** | **17551.03** | **15220.22** | **2743.74** | **3212.50** |
| --- | --- | --- | --- | --- | --- | --- | --- | --- | --- | --- | --- |
| **Pitcairnia** | **archeri** | **PF** | **c3mes** | **10** | **154096** | **830.25** | **3400.67** | **25665.79** | **31861.08** | **4094.21** | **6328.00** |
| **Pitcairnia** | **arcuata** | **PF** | **c3mes** | **178** | **1155303** | **847.10** | **3120.50** | **20353.24** | **21954.36** | **3054.56** | **3541.33** |
| **Pitcairnia** | **arenaria** | **PF** | **c3mes** | **4** | **4164** | **1537.94** | **2150.50** | **8234.63** | **3075.48** | **1298.69** | **734.00** |
| **Pitcairnia** | **arida** | **PF** | **c3mes** | **4** | **1619** | **1219.56** | **282.00** | **15313.52** | **6469.32** | **2472.88** | **1281.00** |
| **Pitcairnia** | **armata** | **PF** | **c3mes** | **22** | **76941** | **240.85** | **822.58** | **13699.42** | **9833.38** | **2448.50** | **1892.92** |
| **Pitcairnia** | **atrorubens** | **PF** | **c3mes** | **112** | **315201** | **971.48** | **2817.75** | **21299.50** | **34589.74** | **3120.61** | **5678.50** |
| **Pitcairnia** | **azouryi** | **PF** | **c3mes** | **10** | **18817** | **223.12** | **498.17** | **6590.51** | **5059.75** | **1047.43** | **707.00** |
| **Pitcairnia** | **bakeri** | **PF** | **c3mes** | **46** | **57208** | **1446.58** | **3034.00** | **19633.67** | **24291.56** | **2905.79** | **3682.67** |
| **Pitcairnia** | **barbatostigma** | **PF** | **c3mes** | **7** | **502** | **245.22** | **304.07** | **7304.64** | **719.81** | **1162.48** | **115.75** |
| **Pitcairnia** | **barrigae** | **PF** | **c3mes** | **10** | **2025** | **864.39** | **1374.42** | **19296.59** | **10939.81** | **2794.38** | **1689.67** |
| **Pitcairnia** | **basincurva** | **PF** | **c3mes** | **5** | **200** | **1349.35** | **498.67** | **17914.95** | **1501.97** | **2788.90** | **177.00** |
| **Pitcairnia** | **bella** | **PF** | **c3mes** | **10** | **115848** | **1192.20** | **2104.75** | **21607.26** | **7232.98** | **3273.08** | **1666.67** |
| **Pitcairnia** | **bergii** | **PF** | **c3mes** | **3** | **1** | **1599.00** | **1364.25** | **6772.64** | **642.29** | **979.58** | **185.75** |
| **Pitcairnia** | **bicolor** | **PF** | **c3mes** | **13** | **14922** | **1461.83** | **1823.67** | **21635.31** | **28138.56** | **3287.99** | **5229.83** |
| **Pitcairnia** | **biflora** | **PF** | **c3mes** | **8** | **20896** | **1437.11** | **1240.33** | **15903.61** | **9745.57** | **2418.76** | **1430.67** |
| **Pitcairnia** | **billbergioides** | **PF** | **c3mes** | **4** | **17770** | **1584.27** | **1163.75** | **4896.25** | **4385.09** | **762.56** | **699.50** |
| **Pitcairnia** | **brachysperma** | **PF** | **c3mes** | **9** | **172888** | **1869.21** | **963.60** | **13865.06** | **11265.79** | **2069.68** | **1664.00** |
| **Pitcairnia** | **brackeana** | **PF** | **c3mes** | **4** | **1774** | **1966.04** | **1627.50** | **11095.79** | **5969.84** | **1721.48** | **1108.75** |
| **Pitcairnia** | **bradei** | **PF** | **c3mes** | **10** | **53123** | **937.13** | **352.50** | **6745.12** | **4302.38** | **1083.20** | **664.00** |
| **Pitcairnia** | **breedlovei** | **PF** | **c3mes** | **7** | **4230** | **947.69** | **1352.50** | **6248.47** | **5420.61** | **1069.81** | **755.67** |
| **Pitcairnia** | **brevicalycina** | **PF** | **c3mes** | **11** | **950568** | **1798.27** | **3716.33** | **9701.01** | **9974.81** | **1405.61** | **1773.25** |
| **Pitcairnia** | **brittoniana** | **PF** | **c3mes** | **110** | **3193534** | **1388.13** | **3426.75** | **21642.60** | **30557.38** | **3038.31** | **3753.00** |
| **Pitcairnia** | **bromeliifolia** | **PF** | **c3mes** | **3** | **10541** | **762.42** | **997.50** | **9550.78** | **1638.50** | **1529.33** | **242.00** |
| **Pitcairnia** | **brongniartiana** | **PF** | **c3mes** | **22** | **88409** | **1327.81** | **3243.67** | **19236.61** | **16639.94** | **2706.88** | **2983.58** |
| **Pitcairnia** | **brunnescens** | **PF** | **c3mes** | **13** | **57639** | **2085.47** | **1166.00** | **14117.03** | **10549.38** | **1951.58** | **1848.00** |
| **Pitcairnia** | **bulbosa** | **PF** | **c3mes** | **20** | **526435** | **119.40** | **220.25** | **16218.63** | **12305.22** | **2845.62** | **1941.25** |
| **Pitcairnia** | **calcicola** | **PF** | **c3mes** | **26** | **3744** | **174.25** | **522.50** | **10625.72** | **5633.90** | **1850.56** | **1061.00** |
| **Pitcairnia** | **calderonii** | **PF** | **c3mes** | **6** | **29704** | **1057.33** | **1238.25** | **10806.81** | **5190.75** | **1766.38** | **798.25** |
| **Pitcairnia** | **cardenasii** | **PF** | **c3mes** | **6** | **50507** | **1409.42** | **1120.00** | **5696.16** | **4011.04** | **921.25** | **795.75** |
| **Pitcairnia** | **caricifolia** | **PF** | **c3mes** | **52** | **4324498** | **298.70** | **1712.67** | **15439.62** | **12628.95** | **2630.27** | **2178.50** |
| **Pitcairnia** | **carinata** | **PF** | **c3mes** | **11** | **80548** | **983.67** | **1612.08** | **10226.27** | **5283.84** | **1399.98** | **446.50** |
| **Pitcairnia** | **chiapensis** | **PF** | **c3mes** | **3** | **6004** | **939.25** | **1098.25** | **8450.74** | **3263.12** | **1428.25** | **474.50** |

**
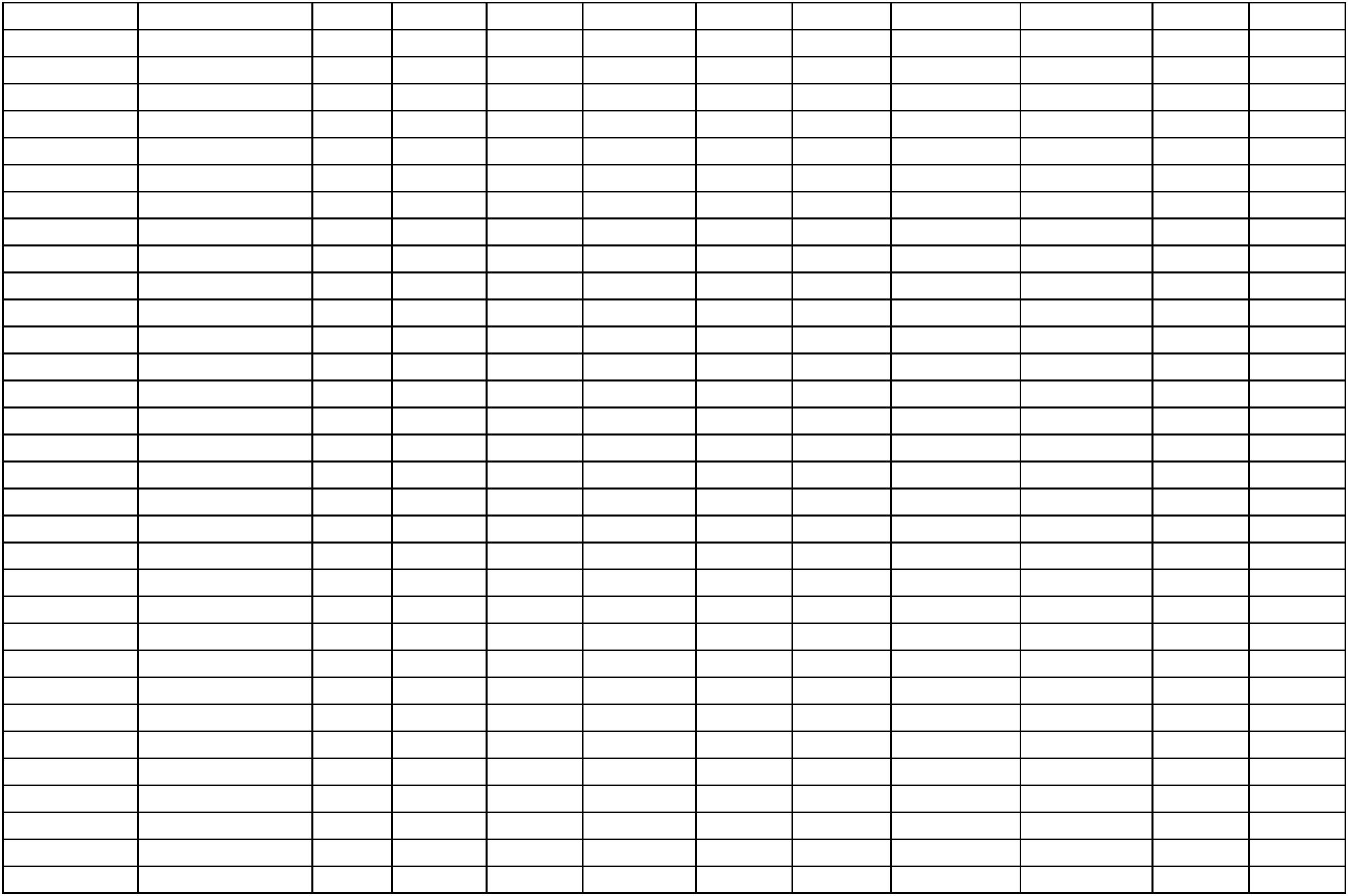
**

| **Pitcairnia** | **chiquitana** | **PF** | **c3mes** | **3** | **19** | **579.00** | **117.00** | **6566.64** | **84.30** | **1155.58** | **12.75** |
| --- | --- | --- | --- | --- | --- | --- | --- | --- | --- | --- | --- |
| **Pitcairnia** | **chiriquensis** | **PF** | **c3mes** | **6** | **3277** | **150.79** | **256.00** | **18529.89** | **4937.78** | **2995.17** | **883.00** |
| **Pitcairnia** | **clarkii** | **PF** | **c3mes** | **3** | **1** | **458.28** | **51.50** | **14996.65** | **142.32** | **2083.33** | **27.00** |
| **Pitcairnia** | **commixta** | **PF** | **c3mes** | **22** | **347525** | **1731.57** | **2877.25** | **14251.16** | **15459.61** | **2031.03** | **2061.42** |
| **Pitcairnia** | **compostelae** | **PF** | **c3mes** | **4** | **609** | **1396.21** | **809.00** | **6526.07** | **1437.84** | **1042.96** | **276.17** |
| **Pitcairnia** | **condorensis** | **PF** | **c3mes** | **4** | **5** | **1103.13** | **190.25** | **14112.14** | **238.44** | **2303.38** | **11.25** |
| **Pitcairnia** | **corallina** | **PF** | **c3mes** | **11** | **930306** | **633.36** | **1639.00** | **14667.10** | **11355.97** | **2419.45** | **1896.33** |
| **Pitcairnia** | **costata** | **PF** | **c3mes** | **3** | **2610** | **1641.75** | **2233.25** | **19207.31** | **15647.25** | **2682.67** | **2584.50** |
| **Pitcairnia** | **cremersii** | **PF** | **c3mes** | **3** | **27** | **185.89** | **29.67** | **16429.75** | **1779.88** | **2602.94** | **187.50** |
| **Pitcairnia** | **crinita** | **PF** | **c3mes** | **3** | **1** | **67.92** | **4.75** | **15221.46** | **119.23** | **2557.25** | **18.75** |
| **Pitcairnia** | **ctenophylla** | **PF** | **c3mes** | **14** | **34514** | **884.70** | **1281.42** | **18101.79** | **7420.34** | **2866.10** | **1489.50** |
| **Pitcairnia** | **cubensis** | **PF** | **c3mes** | **9** | **29289** | **231.93** | **885.50** | **10912.33** | **8258.26** | **1645.74** | **1013.17** |
| **Pitcairnia** | **curvidens** | **PF** | **c3mes** | **4** | **28094** | **1184.63** | **991.00** | **10822.33** | **6040.91** | **1531.69** | **573.25** |
| **Pitcairnia** | **cuzcoensis** | **PF** | **c3mes** | **4** | **39812** | **1719.50** | **3732.00** | **8170.20** | **2719.34** | **1374.77** | **849.25** |
| **Pitcairnia** | **cylindrostachya** | **PF** | **c3mes** | **4** | **6262** | **1293.93** | **591.75** | **7286.97** | **4031.30** | **1258.66** | **616.00** |
| **Pitcairnia** | **decidua** | **PF** | **c3mes** | **30** | **40012** | **816.99** | **1687.42** | **9115.82** | **5752.46** | **1311.87** | **510.17** |
| **Pitcairnia** | **dendroidea** | **PF** | **c3mes** | **14** | **38900** | **2215.21** | **2102.25** | **12845.50** | **15102.79** | **1730.96** | **2028.08** |
| **Pitcairnia** | **deroosei** | **PF** | **c3mes** | **4** | **260** | **1729.48** | **600.25** | **12885.58** | **5705.63** | **1832.63** | **859.75** |
| **Pitcairnia** | **diffusa** | **PF** | **c3mes** | **51** | **1901938** | **1797.65** | **3561.75** | **15172.76** | **20841.13** | **2123.39** | **2949.75** |
| **Pitcairnia** | **dodsonii** | **PF** | **c3mes** | **24** | **16236** | **1938.63** | **2054.50** | **15820.95** | **20476.55** | **2356.71** | **3190.00** |
| **Pitcairnia** | **domingensis** | **PF** | **c3mes** | **4** | **695** | **154.10** | **241.40** | **10646.87** | **7281.65** | **1729.65** | **995.25** |
| **Pitcairnia** | **echinata** | **PF** | **c3mes** | **24** | **1289807** | **997.75** | **3629.25** | **15511.05** | **31095.33** | **2480.55** | **5107.17** |
| **Pitcairnia** | **elizabethae** | **PF** | **c3mes** | **4** | **460** | **1246.06** | **1192.00** | **10915.58** | **4100.04** | **1555.06** | **364.25** |
| **Pitcairnia** | **elongata** | **PF** | **c3mes** | **15** | **946258** | **1175.84** | **3006.58** | **11759.55** | **32329.88** | **1869.96** | **5148.17** |
| **Pitcairnia** | **elvirae** | **PF** | **c3mes** | **7** | **16084** | **1165.52** | **2542.75** | **21023.21** | **20720.56** | **3249.56** | **3311.25** |
| **Pitcairnia** | **encholirioides** | **PF** | **c3mes** | **5** | **287** | **585.47** | **325.00** | **9930.35** | **1728.39** | **1405.65** | **60.50** |
| **Pitcairnia** | **ensifolia** | **PF** | **c3mes** | **9** | **72150** | **725.39** | **423.75** | **10077.41** | **3997.93** | **1610.49** | **381.75** |
| **Pitcairnia** | **ferrell-ingramiae** | **PF** | **c3mes** | **29** | **167789** | **902.21** | **3249.50** | **20163.50** | **14030.40** | **2914.98** | **2446.25** |
| **Pitcairnia** | **filispina** | **PF** | **c3mes** | **4** | **1379** | **1134.06** | **498.25** | **18408.27** | **2194.43** | **2954.46** | **297.67** |
| **Pitcairnia** | **flammea** | **PF** | **c3mes** | **126** | **622317** | **667.78** | **1698.50** | **10662.09** | **15745.29** | **1454.80** | **1775.75** |
| **Pitcairnia** | **flexuosa** | **PF** | **c3mes** | **11** | **202974** | **1073.02** | **2155.50** | **9014.46** | **8739.85** | **1479.89** | **1063.25** |
| **Pitcairnia** | **fluvialis** | **PF** | **c3mes** | **3** | **4** | **608.61** | **412.50** | **16268.99** | **3800.04** | **2693.06** | **461.50** |
| **Pitcairnia** | **fruticosa** | **PF** | **c3mes** | **3** | **48** | **1801.14** | **253.58** | **17739.84** | **645.73** | **2534.36** | **101.33** |

**
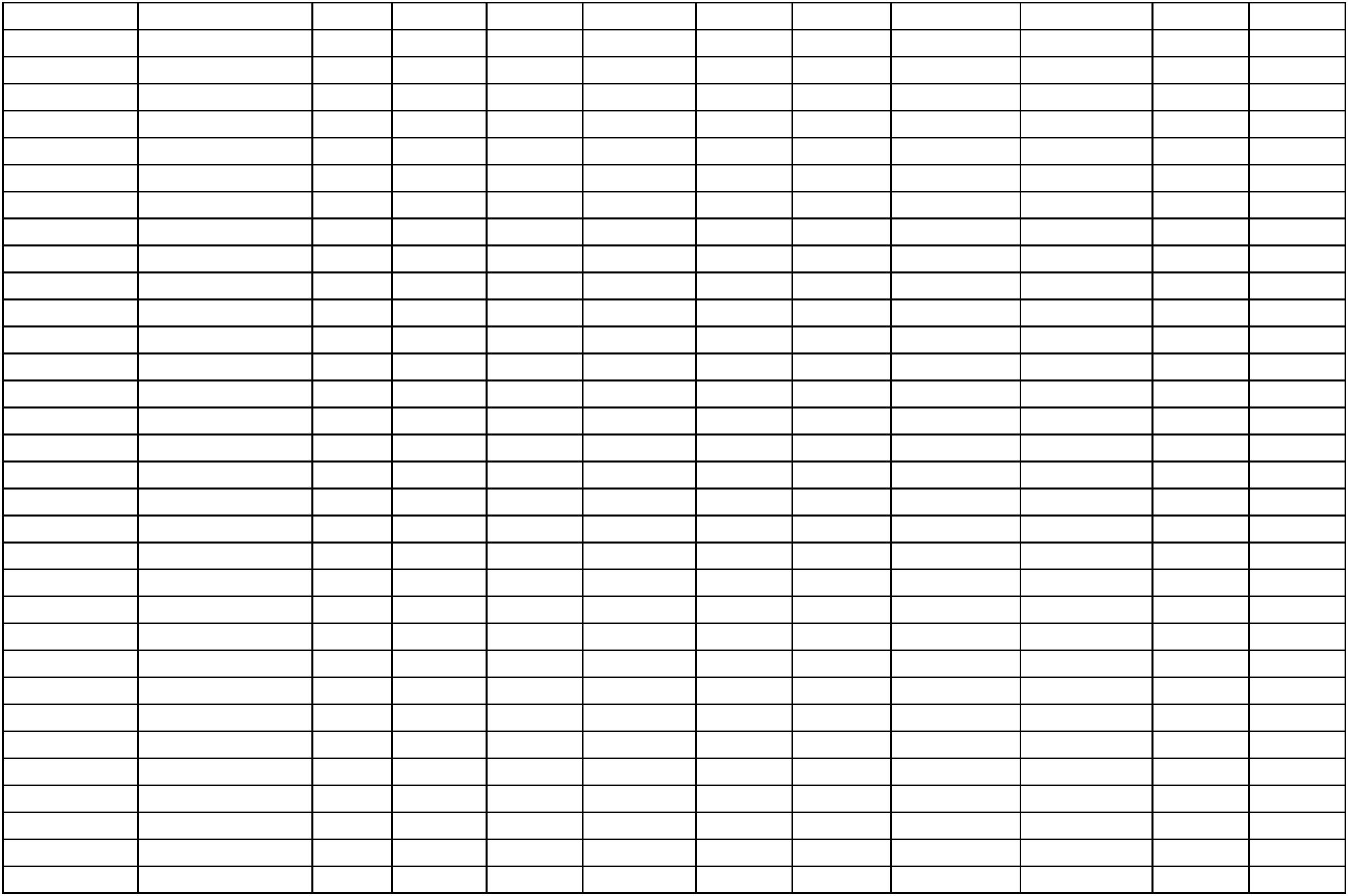
**

| **Pitcairnia** | **fuertesii** | **PF** | **c3mes** | **6** | **6249** | **753.10** | **1190.00** | **8813.40** | **5616.48** | **1376.21** | **826.75** |
| --- | --- | --- | --- | --- | --- | --- | --- | --- | --- | --- | --- |
| **Pitcairnia** | **funkiae** | **PF** | **c3mes** | **6** | **2612** | **623.04** | **204.75** | **22110.87** | **11143.78** | **3457.33** | **1559.25** |
| **Pitcairnia** | **fusca** | **PF** | **c3mes** | **24** | **41567** | **2104.69** | **1922.58** | **10728.99** | **11918.35** | **1524.10** | **1820.75** |
| **Pitcairnia** | **geyskesii** | **PF** | **c3mes** | **7** | **34361** | **267.45** | **265.00** | **12820.97** | **6024.40** | **2085.17** | **933.08** |
| **Pitcairnia** | **glaziovii** | **PF** | **c3mes** | **7** | **7159** | **947.46** | **888.00** | **11670.35** | **5716.96** | **1645.40** | **860.75** |
| **Pitcairnia** | **goudae** | **PF** | **c3mes** | **4** | **52** | **1474.48** | **922.92** | **21622.06** | **17196.47** | **3110.73** | **2590.33** |
| **Pitcairnia** | **guzmanioides** | **PF** | **c3mes** | **34** | **2216878** | **1828.89** | **2421.75** | **14902.92** | **32224.77** | **2024.50** | **3512.50** |
| **Pitcairnia** | **halophila** | **PF** | **c3mes** | **38** | **42844** | **115.55** | **1266.00** | **19646.83** | **17944.68** | **3450.80** | **3380.50** |
| **Pitcairnia** | **harlingii** | **PF** | **c3mes** | **7** | **80043** | **650.26** | **1108.67** | **25266.85** | **25156.76** | **4076.33** | **4354.00** |
| **Pitcairnia** | **harrylutheri** | **PF** | **c3mes** | **9** | **12434** | **1184.05** | **1668.83** | **16989.43** | **5624.44** | **2675.77** | **762.67** |
| **Pitcairnia** | **heterophylla** | **PF** | **c3mes** | **201** | **5547860** | **1203.54** | **2567.33** | **13225.56** | **32281.90** | **1971.46** | **3953.75** |
| **Pitcairnia** | **hintoniana** | **PF** | **c3mes** | **5** | **1287** | **1251.05** | **1448.00** | **7456.77** | **3105.25** | **1305.00** | **343.50** |
| **Pitcairnia** | **hirtzii** | **PF** | **c3mes** | **15** | **7460** | **1564.99** | **989.33** | **16325.32** | **11960.55** | **2526.35** | **1998.33** |
| **Pitcairnia** | **hooveri** | **PF** | **c3mes** | **12** | **4877** | **2300.31** | **2355.25** | **11810.84** | **10760.23** | **1533.27** | **1947.75** |
| **Pitcairnia** | **imbricata** | **PF** | **c3mes** | **78** | **912556** | **755.54** | **1831.58** | **15025.73** | **28407.89** | **2256.33** | **4120.00** |
| **Pitcairnia** | **integrifolia** | **PF** | **c3mes** | **4** | **477460** | **465.60** | **895.92** | **12712.34** | **13128.84** | **1946.19** | **1690.33** |
| **Pitcairnia** | **irwiniana** | **PF** | **c3mes** | **11** | **57423** | **797.19** | **483.50** | **9620.60** | **5551.51** | **1575.32** | **647.75** |
| **Pitcairnia** | **juncoides** | **PF** | **c3mes** | **38** | **65982** | **114.23** | **141.00** | **17029.82** | **8338.44** | **3106.69** | **1674.25** |
| **Pitcairnia** | **kalbreyeri** | **PF** | **c3mes** | **51** | **1272730** | **1530.86** | **2368.92** | **21308.24** | **30791.20** | **2937.99** | **3518.75** |
| **Pitcairnia** | **karwinskyana** | **PF** | **c3mes** | **45** | **536915** | **1621.99** | **1888.25** | **6993.75** | **9333.78** | **1133.11** | **1353.00** |
| **Pitcairnia** | **kniphofioides** | **PF** | **c3mes** | **3** | **74** | **1660.92** | **381.25** | **10614.47** | **2375.75** | **1600.08** | **322.75** |
| **Pitcairnia** | **kressii** | **PF** | **c3mes** | **7** | **6855** | **1131.02** | **338.75** | **20383.71** | **4552.84** | **2980.40** | **529.50** |
| **Pitcairnia** | **lanuginosa** | **PF** | **c3mes** | **70** | **5046430** | **868.84** | **2362.25** | **10225.25** | **13279.84** | **1684.58** | **2307.50** |
| **Pitcairnia** | **lehmannii** | **PF** | **c3mes** | **35** | **234024** | **1792.15** | **4020.67** | **17026.47** | **22709.63** | **2467.08** | **3632.58** |
| **Pitcairnia** | **leprosa** | **PF** | **c3mes** | **6** | **825** | **1152.42** | **1486.00** | **7327.79** | **5137.09** | **1222.75** | **390.75** |
| **Pitcairnia** | **lignosa** | **PF** | **c3mes** | **4** | **41679** | **1696.08** | **1934.67** | **8396.27** | **5408.10** | **1216.69** | **1218.00** |
| **Pitcairnia** | **longipes** | **PF** | **c3mes** | **3** | **1246** | **198.06** | **312.17** | **26928.97** | **32237.11** | **4282.94** | **4553.00** |
| **Pitcairnia** | **longissimiflora** | **PF** | **c3mes** | **3** | **29** | **977.50** | **845.50** | **6916.71** | **4636.67** | **1120.25** | **797.75** |
| **Pitcairnia** | **luteyniorum** | **PF** | **c3mes** | **8** | **10186** | **2083.58** | **3034.00** | **13413.15** | **11751.32** | **1772.85** | **2207.33** |
| **Pitcairnia** | **lymansmithiana** | **PF** | **c3mes** | **8** | **27765** | **1258.56** | **628.50** | **23968.56** | **10243.34** | **3434.63** | **1189.00** |
| **Pitcairnia** | **macarenensis** | **PF** | **c3mes** | **6** | **104574** | **525.60** | **720.67** | **16936.48** | **12885.70** | **2837.31** | **2072.00** |
| **Pitcairnia** | **macranthera** | **PF** | **c3mes** | **24** | **109059** | **1321.15** | **2017.42** | **22201.36** | **41182.25** | **3335.58** | **6926.00** |
| **Pitcairnia** | **maidifolia** | **PF** | **c3mes** | **138** | **4552314** | **908.58** | **2751.08** | **16353.23** | **31698.85** | **2542.84** | **4332.50** |

**
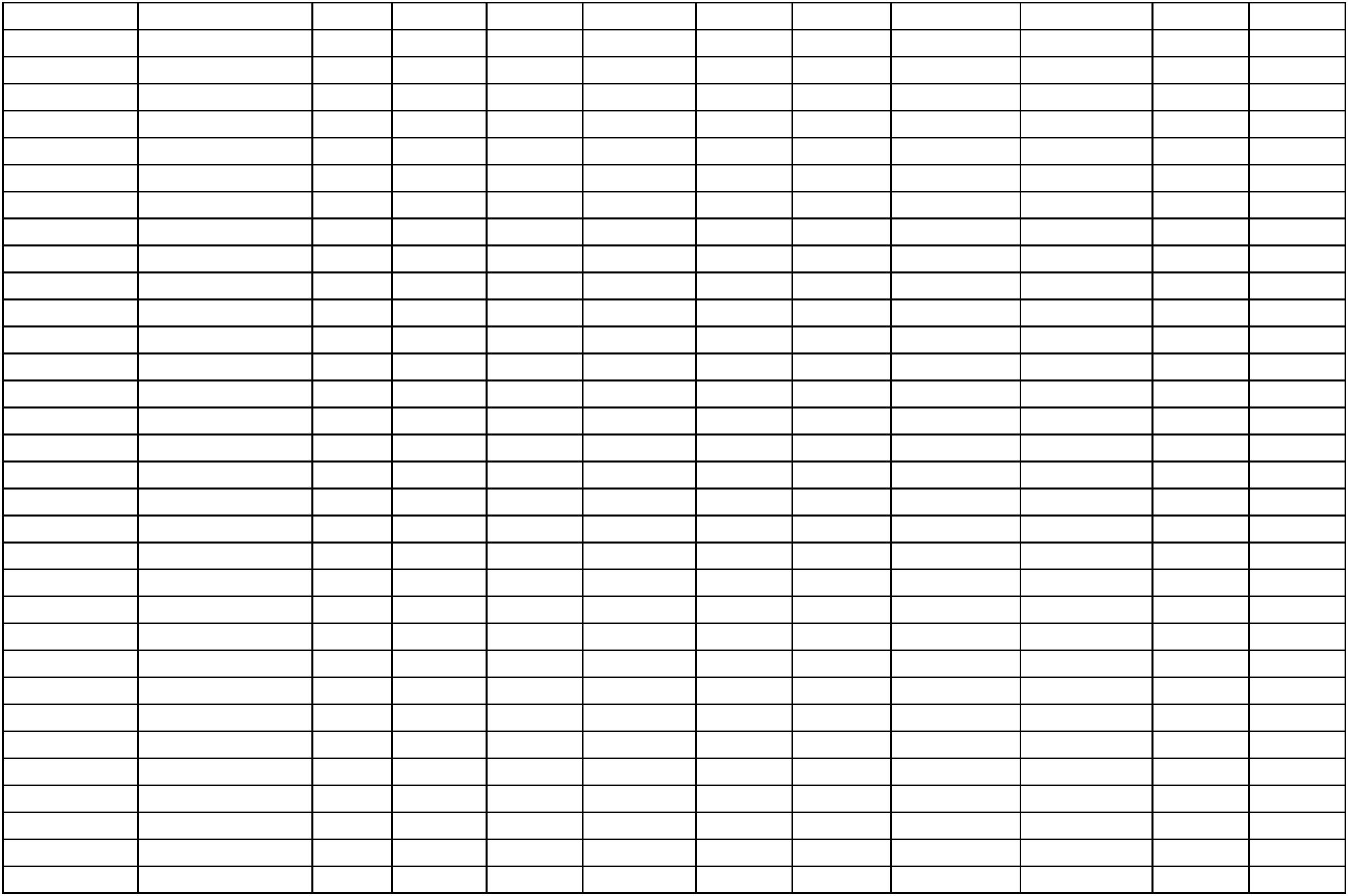
**

| **Pitcairnia** | **marinii** | **PF** | **c3mes** | **3** | **21** | **1296.53** | **345.67** | **16974.62** | **127.45** | **2697.61** | **73.00** |
| --- | --- | --- | --- | --- | --- | --- | --- | --- | --- | --- | --- |
| **Pitcairnia** | **megasepala** | **PF** | **c3mes** | **43** | **394131** | **520.06** | **2262.25** | **17548.40** | **20550.03** | **2880.31** | **3503.00** |
| **Pitcairnia** | **melanopoda** | **PF** | **c3mes** | **3** | **25** | **1661.76** | **344.33** | **8493.19** | **438.37** | **1346.62** | **63.47** |
| **Pitcairnia** | **meridensis** | **PF** | **c3mes** | **7** | **95308** | **1653.32** | **2670.25** | **14836.71** | **14421.04** | **2145.56** | **2221.25** |
| **Pitcairnia** | **micheliana** | **PF** | **c3mes** | **11** | **3678** | **1147.11** | **615.75** | **4535.62** | **3360.96** | **898.67** | **516.75** |
| **Pitcairnia** | **minicorallina** | **PF** | **c3mes** | **3** | **22** | **511.78** | **230.67** | **13528.10** | **3852.64** | **2279.22** | **612.67** |
| **Pitcairnia** | **mituensis** | **PF** | **c3mes** | **3** | **1** | **165.75** | **31.50** | **21536.48** | **392.31** | **3487.83** | **66.50** |
| **Pitcairnia** | **modesta** | **PF** | **c3mes** | **3** | **127** | **1119.25** | **604.00** | **7524.80** | **849.98** | **1343.58** | **297.75** |
| **Pitcairnia** | **mucida** | **PF** | **c3mes** | **3** | **1788** | **1251.83** | **870.17** | **18737.32** | **7594.57** | **2892.42** | **1528.08** |
| **Pitcairnia** | **multiflora** | **PF** | **c3mes** | **15** | **138802** | **775.56** | **1167.25** | **17225.90** | **16530.36** | **2540.17** | **2197.00** |
| **Pitcairnia** | **multiramosa** | **PF** | **c3mes** | **6** | **6186** | **1332.10** | **1627.80** | **7697.31** | **12750.93** | **1242.66** | **2190.05** |
| **Pitcairnia** | **neillii** | **PF** | **c3mes** | **7** | **437** | **1229.21** | **1252.25** | **15774.40** | **8306.35** | **2472.90** | **1329.42** |
| **Pitcairnia** | **nigra** | **PF** | **c3mes** | **62** | **487730** | **1758.64** | **2466.00** | **14963.76** | **25997.82** | **2091.97** | **3794.08** |
| **Pitcairnia** | **nobilis** | **PF** | **c3mes** | **3** | **172** | **599.69** | **342.58** | **7440.90** | **2351.31** | **1368.33** | **375.67** |
| **Pitcairnia** | **nubigena** | **PF** | **c3mes** | **4** | **5475** | **1610.19** | **2171.25** | **9336.77** | **2175.49** | **1349.44** | **688.25** |
| **Pitcairnia** | **nuda** | **PF** | **c3mes** | **16** | **104252** | **276.93** | **392.00** | **14043.83** | **10105.05** | **2284.04** | **2031.25** |
| **Pitcairnia** | **oaxacana** | **PF** | **c3mes** | **9** | **143427** | **911.59** | **1808.75** | **7088.89** | **2235.28** | **1243.40** | **548.20** |
| **Pitcairnia** | **oblongifolia** | **PF** | **c3mes** | **3** | **11** | **1628.69** | **1364.25** | **7416.14** | **1972.67** | **1106.97** | **567.92** |
| **Pitcairnia** | **occidentalis** | **PF** | **c3mes** | **9** | **60277** | **809.95** | **2198.50** | **19400.57** | **10775.81** | **3211.25** | **2206.00** |
| **Pitcairnia** | **palmeri** | **PF** | **c3mes** | **17** | **225469** | **1519.72** | **1893.50** | **5746.86** | **6393.05** | **941.87** | **987.25** |
| **Pitcairnia** | **palmoides** | **PF** | **c3mes** | **14** | **87449** | **1254.30** | **2942.00** | **17007.36** | **21756.75** | **2556.25** | **3331.25** |
| **Pitcairnia** | **paniculata** | **PF** | **c3mes** | **40** | **720752** | **1961.57** | **3582.30** | **8606.94** | **13424.36** | **1359.65** | **2236.55** |
| **Pitcairnia** | **paraguayensis** | **PF** | **c3mes** | **3** | **302** | **310.17** | **61.50** | **8722.98** | **308.14** | **1413.72** | **44.00** |
| **Pitcairnia** | **patentiflora** | **PF** | **c3mes** | **41** | **625420** | **496.86** | **1842.92** | **17053.96** | **12380.47** | **2798.64** | **1186.75** |
| **Pitcairnia** | **pavonii** | **PF** | **c3mes** | **13** | **275165** | **2188.42** | **3159.42** | **6956.23** | **12651.02** | **937.59** | **1621.67** |
| **Pitcairnia** | **peruana** | **PF** | **c3mes** | **3** | **13** | **2022.00** | **861.75** | **8317.50** | **643.39** | **1249.42** | **134.50** |
| **Pitcairnia** | **petraea** | **PF** | **c3mes** | **3** | **19** | **2273.14** | **165.75** | **7924.11** | **563.63** | **1030.67** | **61.00** |
| **Pitcairnia** | **pomacochae** | **PF** | **c3mes** | **12** | **48389** | **1563.51** | **1747.75** | **8464.49** | **9014.72** | **1418.17** | **1499.00** |
| **Pitcairnia** | **poortmanii** | **PF** | **c3mes** | **9** | **63041** | **1220.93** | **1581.50** | **13038.07** | **10732.64** | **2057.52** | **1399.50** |
| **Pitcairnia** | **prolifera** | **PF** | **c3mes** | **3** | **17** | **1263.69** | **513.75** | **6143.39** | **958.59** | **1053.11** | **86.50** |
| **Pitcairnia** | **pruinosa** | **PF** | **c3mes** | **16** | **118161** | **115.16** | **268.42** | **14022.97** | **8554.37** | **2517.47** | **1674.25** |
| **Pitcairnia** | **puberula** | **PF** | **c3mes** | **4** | **96230** | **1149.50** | **620.42** | **11495.96** | **10872.69** | **1727.31** | **1260.42** |
| **Pitcairnia** | **pulverulenta** | **PF** | **c3mes** | **8** | **109345** | **1449.07** | **2210.00** | **8529.12** | **7298.02** | **1460.50** | **1382.33** |

**
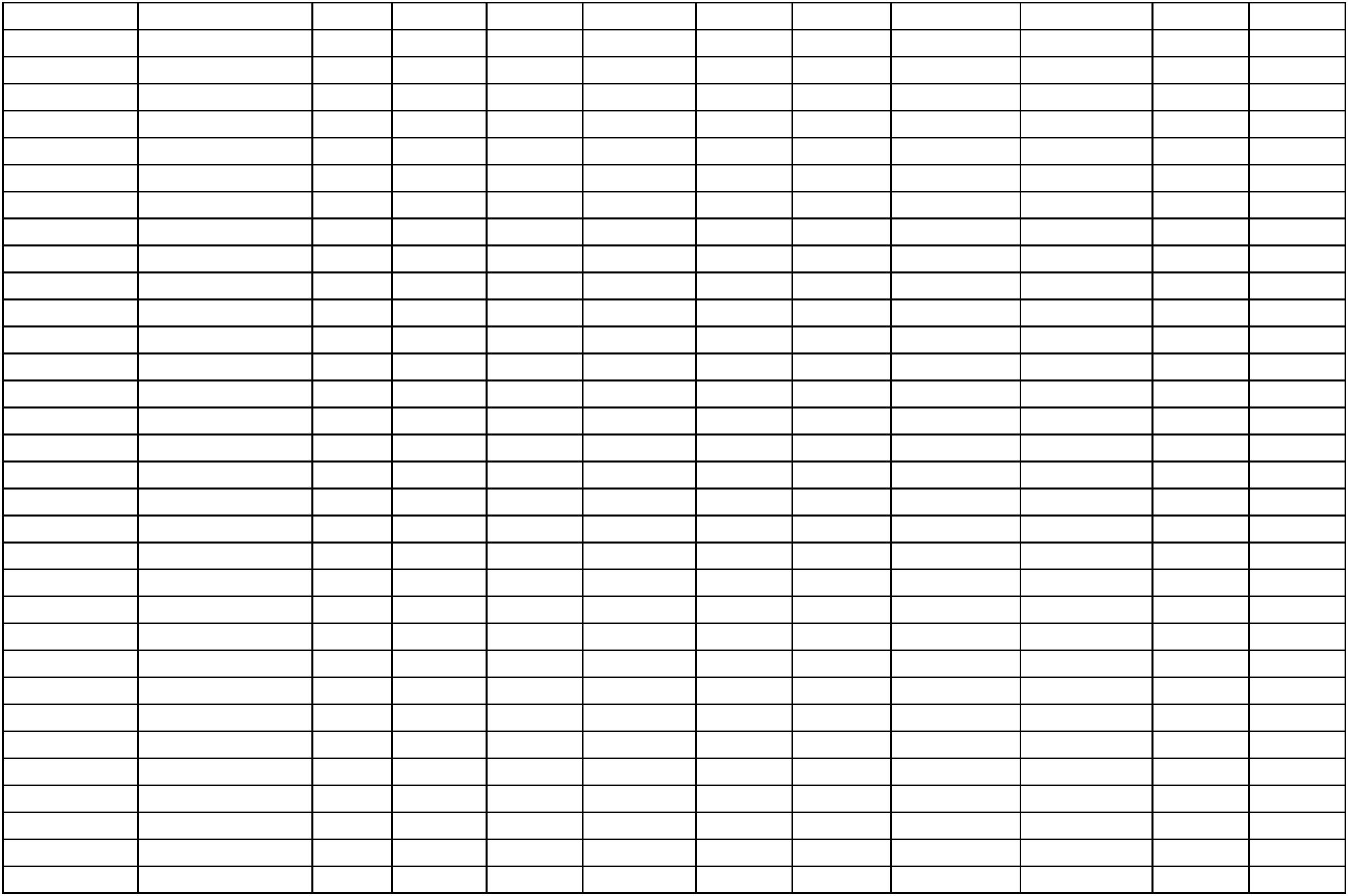
**

| **Pitcairnia** | **pungens** | **PF** | **c3mes** | **142** | **1190279** | **2498.44** | **4569.33** | **8930.81** | **17966.43** | **1158.01** | **2771.08** |
| --- | --- | --- | --- | --- | --- | --- | --- | --- | --- | --- | --- |
| **Pitcairnia** | **punicea** | **PF** | **c3mes** | **28** | **82543** | **362.58** | **1651.58** | **16925.89** | **12815.00** | **2720.13** | **2202.25** |
| **Pitcairnia** | **quesnelioides** | **PF** | **c3mes** | **4** | **45** | **343.78** | **46.13** | **18887.33** | **3362.05** | **3307.25** | **641.00** |
| **Pitcairnia** | **recurvata** | **PF** | **c3mes** | **16** | **161413** | **489.79** | **1570.67** | **15141.49** | **18934.01** | **2302.54** | **2633.00** |
| **Pitcairnia** | **reflexiflora** | **PF** | **c3mes** | **6** | **6731** | **1374.17** | **1708.75** | **9552.56** | **8890.73** | **1466.97** | **1433.00** |
| **Pitcairnia** | **ringens** | **PF** | **c3mes** | **32** | **519107** | **1506.25** | **2307.25** | **9363.73** | **12505.73** | **1399.40** | **1521.50** |
| **Pitcairnia** | **riparia** | **PF** | **c3mes** | **52** | **669929** | **1693.75** | **2942.50** | **14115.16** | **23969.16** | **2140.96** | **3589.33** |
| **Pitcairnia** | **roseana** | **PF** | **c3mes** | **11** | **77199** | **1209.85** | **1409.75** | **5675.72** | **2006.40** | **974.72** | **545.50** |
| **Pitcairnia** | **rubiginosa** | **PF** | **c3mes** | **11** | **2116708** | **252.05** | **887.25** | **19212.54** | **13727.01** | **3063.33** | **2060.67** |
| **Pitcairnia** | **rundelliana** | **PF** | **c3mes** | **13** | **5091** | **1181.61** | **1691.50** | **19155.71** | **10793.74** | **2673.49** | **1490.25** |
| **Pitcairnia** | **samuelssonii** | **PF** | **c3mes** | **3** | **240** | **652.44** | **607.67** | **8487.14** | **1908.70** | **1517.42** | **383.42** |
| **Pitcairnia** | **sastrei** | **PF** | **c3mes** | **8** | **14364** | **209.02** | **185.00** | **15583.56** | **7694.88** | **2498.35** | **1116.50** |
| **Pitcairnia** | **saxicola** | **PF** | **c3mes** | **8** | **219816** | **1111.16** | **1839.50** | **15421.20** | **9965.85** | **2412.26** | **1822.50** |
| **Pitcairnia** | **scandens** | **PF** | **c3mes** | **6** | **197174** | **1658.33** | **2617.25** | **9357.60** | **7812.02** | **1464.92** | **1445.50** |
| **Pitcairnia** | **sceptriformis** | **PF** | **c3mes** | **24** | **173543** | **1385.77** | **2280.25** | **19604.41** | **20035.81** | **2993.65** | **3107.25** |
| **Pitcairnia** | **sceptrigera** | **PF** | **c3mes** | **20** | **51705** | **848.13** | **3394.58** | **14624.96** | **15935.97** | **2117.14** | **2459.00** |
| **Pitcairnia** | **schultzei** | **PF** | **c3mes** | **7** | **576482** | **324.04** | **960.25** | **12592.40** | **12776.60** | **2140.05** | **2600.17** |
| **Pitcairnia** | **semaphora** | **PF** | **c3mes** | **6** | **1467** | **1578.97** | **977.75** | **22972.35** | **5515.84** | **3320.40** | **1258.17** |
| **Pitcairnia** | **simulans** | **PF** | **c3mes** | **27** | **54760** | **1415.11** | **3195.67** | **16980.55** | **20256.73** | **2386.91** | **2944.50** |
| **Pitcairnia** | **sodiroi** | **PF** | **c3mes** | **34** | **5493** | **2173.44** | **2643.42** | **12077.52** | **11507.98** | **1726.93** | **1987.75** |
| **Pitcairnia** | **spectabilis** | **PF** | **c3mes** | **26** | **34469** | **1580.71** | **3560.58** | **18711.83** | **26776.25** | **2625.80** | **4543.25** |
| **Pitcairnia** | **sprucei** | **PF** | **c3mes** | **54** | **1811310** | **123.41** | **416.67** | **16915.59** | **7387.28** | **2804.49** | **1191.05** |
| **Pitcairnia** | **squarrosa** | **PF** | **c3mes** | **6** | **9914** | **1141.65** | **2093.83** | **18325.38** | **14272.48** | **2575.89** | **2266.00** |
| **Pitcairnia** | **staminea** | **PF** | **c3mes** | **7** | **14505** | **164.35** | **629.25** | **9425.30** | **3646.92** | **1265.20** | **573.17** |
| **Pitcairnia** | **stenophylla** | **PF** | **c3mes** | **4** | **98867** | **922.48** | **1467.42** | **9980.17** | **4260.81** | **1634.79** | **114.17** |
| **Pitcairnia** | **stevensonii** | **PF** | **c3mes** | **7** | **11586** | **756.98** | **680.17** | **16708.01** | **16602.08** | **2475.45** | **2151.50** |
| **Pitcairnia** | **suaveolens** | **PF** | **c3mes** | **6** | **5030** | **541.19** | **860.00** | **11969.16** | **6235.87** | **1571.31** | **553.83** |
| **Pitcairnia** | **susannae** | **PF** | **c3mes** | **3** | **32** | **561.53** | **744.42** | **20824.31** | **7100.57** | **3058.83** | **827.50** |
| **Pitcairnia** | **tarapotensis** | **PF** | **c3mes** | **8** | **110527** | **1617.58** | **3849.58** | **8605.53** | **4811.74** | **1337.44** | **582.67** |
| **Pitcairnia** | **tillandsioides** | **PF** | **c3mes** | **3** | **1076** | **1347.97** | **1071.58** | **8401.58** | **956.58** | **1380.47** | **136.92** |
| **Pitcairnia** | **tillii** | **PF** | **c3mes** | **4** | **4** | **410.98** | **460.67** | **21000.93** | **3367.29** | **3127.52** | **476.33** |
| **Pitcairnia** | **torresiana** | **PF** | **c3mes** | **4** | **711773** | **394.81** | **483.50** | **8989.07** | **4585.00** | **1631.65** | **872.42** |
| **Pitcairnia** | **trianae** | **PF** | **c3mes** | **74** | **4085137** | **2079.22** | **2959.50** | **11470.33** | **20488.42** | **1606.67** | **2513.25** |

**
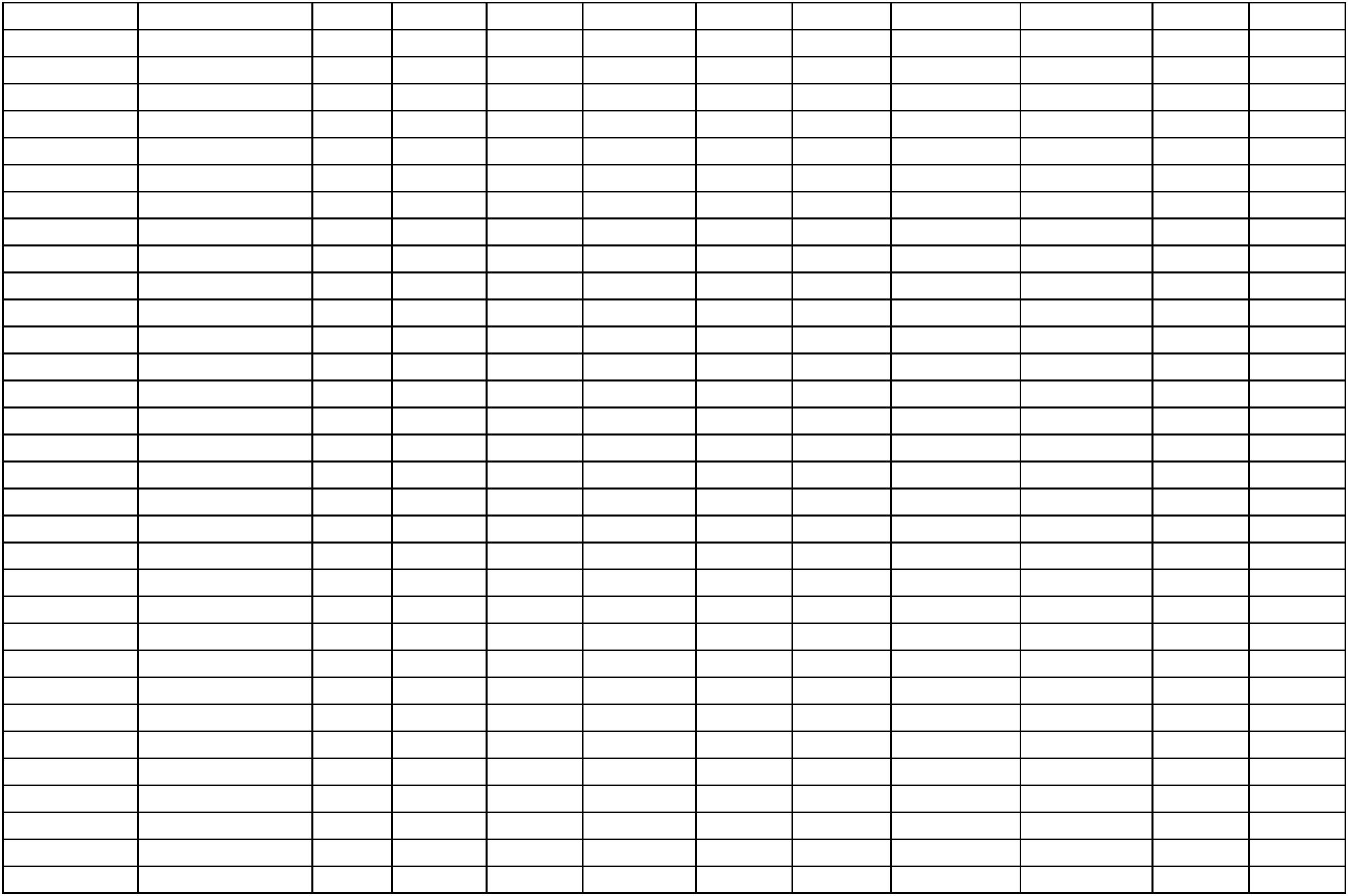
**

| **Pitcairnia** | **truncata** | **PF** | **c3mes** | **5** | **132761** | **1874.46** | **3765.25** | **8509.97** | **2834.03** | **1335.94** | **907.75** |
| --- | --- | --- | --- | --- | --- | --- | --- | --- | --- | --- | --- |
| **Pitcairnia** | **turbinella** | **PF** | **c3mes** | **20** | **303621** | **351.67** | **2772.75** | **17645.48** | **14672.17** | **2903.84** | **2695.75** |
| **Pitcairnia** | **uaupensis** | **PF** | **c3mes** | **30** | **1008843** | **184.64** | **496.75** | **18126.09** | **7272.06** | **3078.74** | **1040.25** |
| **Pitcairnia** | **ulei** | **PF** | **c3mes** | **10** | **43135** | **1048.77** | **599.08** | **9688.58** | **6149.78** | **1486.63** | **837.25** |
| **Pitcairnia** | **undulata** | **PF** | **c3mes** | **4** | **4231** | **214.88** | **306.25** | **20102.51** | **6569.72** | **3353.38** | **1185.00** |
| **Pitcairnia** | **unilateralis** | **PF** | **c3mes** | **3** | **137** | **96.75** | **201.75** | **5415.06** | **1773.72** | **879.08** | **220.25** |
| **Pitcairnia** | **valerioi** | **PF** | **c3mes** | **73** | **69971** | **861.87** | **1340.25** | **21665.22** | **18226.45** | **3133.42** | **2625.00** |
| **Pitcairnia** | **vallisoletana** | **PF** | **c3mes** | **3** | **109** | **2144.42** | **317.00** | **6358.70** | **2144.31** | **961.75** | **282.50** |
| **Pitcairnia** | **wendlandii** | **PF** | **c3mes** | **43** | **304946** | **1021.91** | **2211.25** | **20599.13** | **21600.65** | **2999.44** | **2627.75** |
| **Puya** | **aequatorialis** | **Puy** | **c3terr** | **12** | **40893** | **3048.27** | **3352.25** | **7517.22** | **11136.06** | **1030.88** | **1751.25** |
| **Puya** | **alpestris** | **Puy** | **c3terr** | **30** | **124554** | **2275.38** | **55.75** | **5765.97** | **12987.84** | **663.71** | **1439.25** |
| **Puya** | **angelensis** | **Puy** | **c3terr** | **3** | **56** | **1927.13** | **3420.25** | **7997.90** | **2805.12** | **889.89** | **179.67** |
| **Puya** | **angulonis** | **Puy** | **c3terr** | **6** | **5641** | **1659.56** | **4034.75** | **6612.94** | **6322.15** | **872.49** | **718.75** |
| **Puya** | **angusta** | **Puy** | **c3camterr** | **5** | **2699** | **2628.83** | **129.00** | **9052.24** | **2405.66** | **955.42** | **529.92** |
| **Puya** | **argentea** | **Puy** | **c3terr** | **8** | **145085** | **1893.28** | **2593.42** | **16943.12** | **8543.61** | **2602.78** | **1327.00** |
| **Puya** | **aristeguietae** | **Puy** | **c3terr** | **3** | **1705** | **1524.13** | **2672.25** | **21094.85** | **22140.47** | **1741.69** | **1373.75** |
| **Puya** | **atra** | **Puy** | **c3terr** | **6** | **10472** | **2290.72** | **3649.33** | **3341.47** | **2663.03** | **483.08** | **418.75** |
| **Puya** | **berteroana** | **Puy** | **c3terr** | **7** | **14434** | **1530.83** | **1889.00** | **6735.95** | **2893.03** | **938.26** | **364.25** |
| **Puya** | **berteroniana** | **Puy** | **camterr** | **7** | **10485** | **2376.29** | **2520.50** | **5527.32** | **8306.24** | **662.28** | **988.75** |
| **Puya** | **bicolor** | **Puy** | **c3terr** | **4** | **1812** | **3999.71** | **1257.92** | **8668.53** | **7091.00** | **1250.31** | **1476.00** |
| **Puya** | **boliviensis** | **Puy** | **c3camterr** | **15** | **424365** | **2532.33** | **2922.80** | **3328.68** | **20686.25** | **561.04** | **3504.75** |
| **Puya** | **brackeana** | **Puy** | **c3terr** | **4** | **21** | **3019.08** | **774.25** | **9147.59** | **2625.84** | **986.54** | **199.50** |
| **Puya** | **brittoniana** | **Puy** | **c3terr** | **5** | **840** | **2693.50** | **509.50** | **8114.63** | **4113.57** | **1028.90** | **594.25** |
| **Puya** | **cajasensis** | **Puy** | **c3terr** | **6** | **23245** | **4161.17** | **30.33** | **8515.38** | **6065.14** | **947.74** | **424.83** |
| **Puya** | **cardenasii** | **Puy** | **c3camterr** | **3** | **8426** | **1835.88** | **3237.75** | **9255.15** | **13454.80** | **1447.77** | **2381.55** |
| **Puya** | **castellanosii** | **Puy** | **camterr** | **6** | **386** | **1864.38** | **691.25** | **1161.20** | **336.29** | **157.14** | **58.00** |
| **Puya** | **chilensis** | **Puy** | **camterr** | **17** | **122233** | **1731.23** | **2481.80** | **5527.32** | **8306.24** | **662.28** | **988.75** |
| **Puya** | **claudiae** | **Puy** | **c3terr** | **3** | **505** | **1703.29** | **1414.58** | **5294.23** | **2337.76** | **782.83** | **358.00** |
| **Puya** | **clava-herculis** | **Puy** | **c3terr** | **26** | **73358** | **3007.82** | **1527.25** | **12880.64** | **10966.61** | **1176.05** | **674.42** |
| **Puya** | **coerulea** | **Puy** | **c3camterr** | **18** | **41876** | **3083.68** | **1716.92** | **5961.03** | **5951.94** | **663.13** | **673.58** |
| **Puya** | **cryptantha** | **Puy** | **c3terr** | **6** | **35756** | **1950.13** | **3524.25** | **15249.33** | **15175.90** | **1672.67** | **1500.67** |
| **Puya** | **ctenorhyncha** | **Puy** | **camterr** | **4** | **681** | **1452.75** | **583.50** | **5644.87** | **1028.22** | **678.04** | **149.25** |
| **Puya** | **cuevae** | **Puy** | **c3terr** | **5** | **2208** | **1670.08** | **270.75** | **8222.91** | **2231.81** | **1072.85** | **189.67** |

**
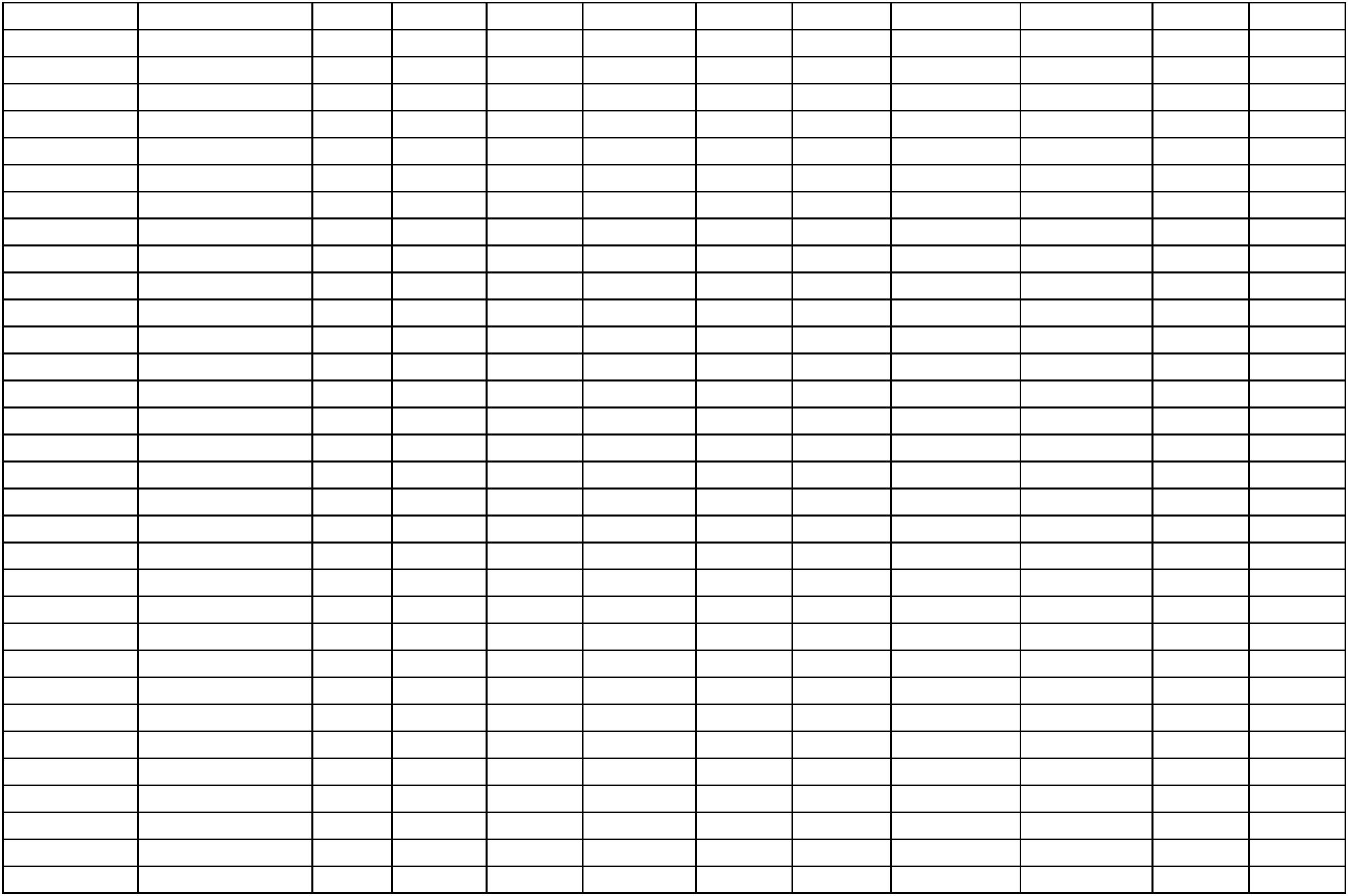
**

| **Puya** | **cylindrica** | **Puy** | **camterr** | **3** | **40820** | **2338.64** | **1386.75** | **6486.17** | **7442.45** | **1117.92** | **1433.50** |
| --- | --- | --- | --- | --- | --- | --- | --- | --- | --- | --- | --- |
| **Puya** | **dasylirioides** | **Puy** | **c3terr** | **31** | **2594** | **2012.67** | **3649.33** | **26144.57** | **11961.66** | **2774.44** | **943.08** |
| **Puya** | **densiflora*** | **Puy** | **camterr** | **3** | **258** | **2981.25** | **447.75** | **6740.63** | **1367.27** | **846.89** | **193.58** |
| **Puya** | **dyckioides** | **Puy** | **c3camterr** | **16** | **26881** | **637.86** | **1555.85** | **2721.38** | **3848.70** | **375.42** | **618.75** |
| **Puya** | **entre-riosensis** | **Puy** | **c3terr** | **3** | **100** | **1985.01** | **1300.25** | **4390.03** | **490.60** | **717.00** | **105.75** |
| **Puya** | **eryngioides** | **Puy** | **c3terr** | **15** | **3210** | **2657.21** | **3169.33** | **8385.04** | **5340.99** | **1092.01** | **660.25** |
| **Puya** | **exigua** | **Puy** | **c3terr** | **6** | **5238** | **3596.67** | **1196.00** | **9755.62** | **12712.55** | **1253.68** | **2142.25** |
| **Puya** | **ferreyrae** | **Puy** | **camterr** | **5** | **50996** | **1759.68** | **565.42** | **8825.88** | **11871.57** | **1420.10** | **2182.25** |
| **Puya** | **ferruginea** | **Puy** | **c3terr** | **64** | **1160484** | **2674.51** | **2090.50** | **7078.54** | **20390.03** | **1050.05** | **3117.50** |
| **Puya** | **floccosa** | **Puy** | **c3camterr** |  | **1420919** | **2884.58** | **1480.33** | **16011.79** | **21956.64** | **2373.79** | **2440.17** |
| **Puya** | **fulgens** | **Puy** | **c3terr** | **4** | **23891** | **1121.13** | **1896.50** | **11385.54** | **7741.87** | **1792.71** | **1527.17** |
| **Puya** | **glandulosa** | **Puy** | **c3camterr** | **3** | **19921** | **1444.88** | **2513.75** | **5682.07** | **6792.06** | **928.83** | **1320.00** |
| **Puya** | **glaucovirens** | **Puy** | **c3terr** | **3** | **59185** | **464.75** | **971.67** | **11440.18** | **6110.36** | **1792.67** | **1487.00** |
| **Puya** | **glomerifera** | **Puy** | **c3terr** | **22** | **29841** | **3785.98** | **726.25** | **10227.83** | **13741.06** | **1133.88** | **2341.33** |
| **Puya** | **goudotiana** | **Puy** | **c3terr** | **29** | **20407** | **3622.61** | **4822.25** | **7948.00** | **13330.57** | **1072.46** | **2423.30** |
| **Puya** | **harmsii** | **Puy** | **camterr** | **7** | **19485** | **3786.58** | **199.75** | **2415.52** | **4678.58** | **335.46** | **752.50** |
| **Puya** | **herrerae** | **Puy** | **c3camterr** | **8** | **140616** | **3938.87** | **655.40** | **7293.98** | **5138.59** | **1040.00** | **1148.25** |
| **Puya** | **herzogii** | **Puy** | **c3terr** | **12** | **180042** | **2385.75** | **492.25** | **8317.94** | **12458.34** | **1334.76** | **2290.30** |
| **Puya** | **hirtzii** | **Puy** | **c3terr** | **3** | **74** | **1796.58** | **2864.75** | **8670.50** | **7753.51** | **1246.06** | **984.67** |
| **Puya** | **hofstenii** | **Puy** | **c3terr** | **3** | **93220** | **2154.44** | **3281.75** | **7900.91** | **15589.15** | **1296.43** | **2664.55** |
| **Puya** | **humilis** | **Puy** | **c3camterr** | **8** | **23799** | **1240.82** | **3087.20** | **8704.11** | **17509.58** | **1370.29** | **3146.50** |
| **Puya** | **hutchisonii** | **Puy** | **c3terr** | **7** | **32654** | **1769.67** | **576.50** | **8562.95** | **1735.30** | **1304.71** | **656.75** |
| **Puya** | **joergensenii** | **Puy** | **c3terr** | **4** | **1** | **2912.03** | **4351.00** | **6989.87** | **519.60** | **792.31** | **64.58** |
| **Puya** | **killipii** | **Puy** | **c3terr** | **6** | **1007** | **3626.50** | **502.00** | **12195.43** | **5307.17** | **1168.90** | **148.83** |
| **Puya** | **kuntzeana** | **Puy** | **c3terr** | **12** | **320007** | **3000.06** | **454.00** | **9161.98** | **11634.08** | **1454.15** | **2132.00** |
| **Puya** | **lanata** | **Puy** | **camterr** | **5** | **24412** | **1568.58** | **515.75** | **6338.88** | **4142.58** | **979.98** | **1051.75** |
| **Puya** | **lasiopoda** | **Puy** | **c3terr** | **3** | **23829** | **2957.25** | **3255.25** | **9335.14** | **6299.80** | **1402.11** | **832.08** |
| **Puya** | **leptostachya** | **Puy** | **c3terr** | **4** | **30009** | **2913.05** | **73.33** | **6511.82** | **2830.30** | **991.67** | **643.50** |
| **Puya** | **lilloi** | **Puy** | **c3terr** | **27** | **71904** | **2699.88** | **791.75** | **3986.62** | **4485.17** | **564.12** | **661.75** |
| **Puya** | **lineata*** | **Puy** | **c3terr** | **5** | **51784** | **2384.63** | **2956.50** | **14230.23** | **6623.14** | **1656.28** | **1246.75** |
| **Puya** | **longispina** | **Puy** | **c3terr** | **4** | **91** | **3968.88** | **4066.00** | **14071.28** | **4390.23** | **1211.83** | **330.00** |
| **Puya** | **maculata** | **Puy** | **c3terr** | **12** | **15778** | **3003.72** | **1804.67** | **9832.13** | **13051.01** | **1193.22** | **2100.25** |
| **Puya** | **micrantha** | **Puy** | **c3terr** | **6** | **12848** | **3249.60** | **3594.25** | **6620.25** | **18272.94** | **1086.63** | **3142.25** |

**
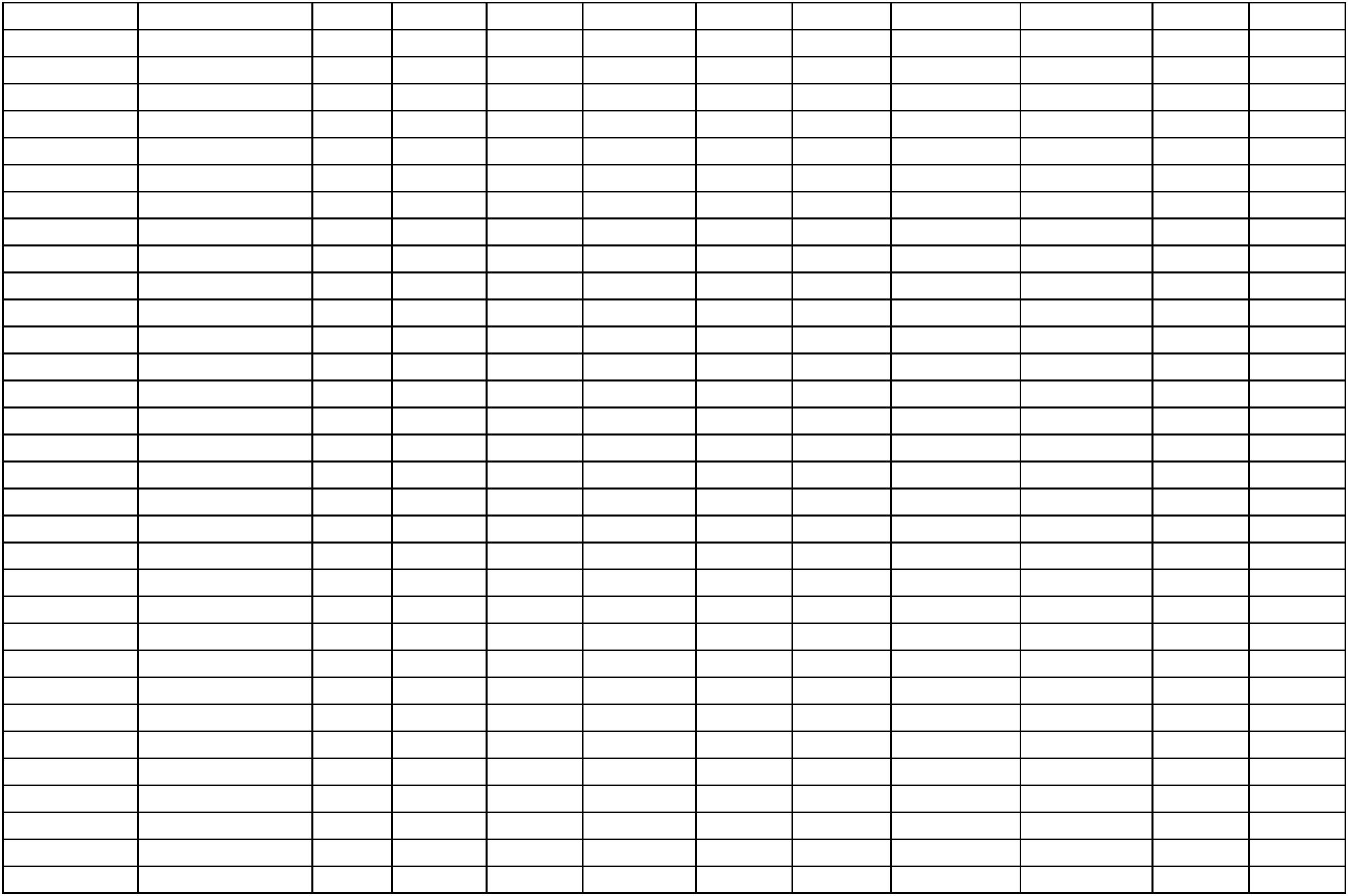
**

| **Puya** | **mirabilis** | **Puy** | **c3terr** | **19** | **168088** | **3915.61** | **247.00** | **4967.42** | **15394.19** | **769.16** | **2655.20** |
| --- | --- | --- | --- | --- | --- | --- | --- | --- | --- | --- | --- |
| **Puya** | **mollis** | **Puy** | **c3terr** | **8** | **46347** | **1826.18** | **4434.75** | **7948.00** | **13330.57** | **1072.46** | **2423.30** |
| **Puya** | **nana** | **Puy** | **c3terr** | **9** | **2189** | **2581.31** | **389.50** | **5243.98** | **2380.04** | **777.28** | **363.75** |
| **Puya** | **navarroana** | **Puy** | **c3terr** | **6** | **917** | **2325.23** | **3494.75** | **9302.05** | **6852.92** | **1184.51** | **1342.50** |
| **Puya** | **nitida** | **Puy** | **c3terr** | **29** | **229183** | **3612.18** | **1935.58** | **12339.19** | **19734.08** | **1298.53** | **1984.00** |
| **Puya** | **novarae** | **Puy** | **c3terr** | **3** | **58** | **1183.17** | **2386.75** | **2234.31** | **171.17** | **300.17** | **42.50** |
| **Puya** | **nutans** | **Puy** | **c3terr** | **6** | **865** | **3312.25** | **1088.00** | **7628.06** | **3175.11** | **810.36** | **279.83** |
| **Puya** | **obconica** | **Puy** | **c3terr** | **9** | **4707** | **2678.43** | **292.50** | **9768.29** | **11713.62** | **1383.20** | **1879.75** |
| **Puya** | **ochroleuca** | **Puy** | **c3terr** | **3** | **11** | **1952.33** | **2857.25** | **22181.04** | **949.06** | **2308.25** | **225.00** |
| **Puya** | **olivacea** | **Puy** | **c3camterr** | **6** | **9596** | **2196.38** | **4016.75** | **6449.11** | **14049.50** | **1026.04** | **2428.55** |
| **Puya** | **parviflora** | **Puy** | **c3terr** | **4** | **149** | **1634.33** | **2892.67** | **8199.70** | **815.28** | **1076.88** | **182.25** |
| **Puya** | **pattersoniae** | **Puy** | **c3terr** | **5** | **508** | **1992.67** | **1946.08** | **6329.04** | **1037.78** | **766.25** | **147.33** |
| **Puya** | **pearcei** | **Puy** | **c3terr** | **13** | **42831** | **2223.14** | **1152.33** | **8570.94** | **6753.94** | **1227.91** | **871.58** |
| **Puya** | **pichinchae** | **Puy** | **c3terr** | **5** | **541** | **3649.92** | **315.33** | **9353.82** | **7486.55** | **1344.08** | **1285.75** |
| **Puya** | **pygmaea** | **Puy** | **c3terr** | **19** | **18036** | **3089.35** | **370.75** | **8432.65** | **13792.58** | **967.85** | **2282.50** |
| **Puya** | **raimondii** | **Puy** | **c3terr** | **11** | **360046** | **2246.33** | **815.00** | **5787.67** | **7273.55** | **685.77** | **1193.25** |
| **Puya** | **reducta** | **Puy** | **c3terr** | **18** | **2666334** | **2823.33** | **1348.67** | **13561.39** | **14944.33** | **1970.75** | **2405.00** |
| **Puya** | **retrorsa** | **Puy** | **c3terr** | **7** | **1632** | **3024.02** | **1036.83** | **8010.27** | **8323.00** | **821.80** | **462.00** |
| **Puya** | **robin-fosteri** | **Puy** | **c3terr** | **8** | **18712** | **1712.82** | **2438.50** | **9126.34** | **8152.97** | **1435.50** | **1571.50** |
| **Puya** | **roldanii** | **Puy** | **c3terr** | **4** | **63** | **2581.43** | **989.75** | **17770.39** | **10548.43** | **2207.33** | **744.58** |
| **Puya** | **roseana** | **Puy** | **c3terr** | **3** | **55** | **1356.00** | **2336.00** | **6815.80** | **2257.26** | **780.69** | **188.42** |
| **Puya** | **rusbyi** | **Puy** | **c3terr** | **10** | **124234** | **1646.13** | **2906.25** | **10408.33** | **10618.16** | **1585.23** | **1896.30** |
| **Puya** | **sanctae-crucis** | **Puy** | **c3terr** | **9** | **11655** | **1316.25** | **2198.50** | **5295.93** | **2441.93** | **814.71** | **368.17** |
| **Puya** | **sanctae-martae** | **Puy** | **c3terr** | **3** | **43085** | **2886.67** | **136.00** | **16252.97** | **8066.08** | **1971.89** | **1461.00** |
| **Puya** | **santosii** | **Puy** | **c3terr** | **54** | **36532** | **2394.24** | **4053.50** | **15111.01** | **1491.01** | **1916.38** | **920.75** |
| **Puya** | **smithii** | **Puy** | **c3camterr** | **9** | **17454** | **1499.33** | **3265.25** | **3709.17** | **3487.57** | **527.16** | **542.80** |
| **Puya** | **sodiroana** | **Puy** | **c3terr** | **4** | **1** | **2216.03** | **3364.05** | **7779.71** | **598.33** | **1054.50** | **1.00** |
| **Puya** | **spathacea** | **Puy** | **c3terr** | **34** | **252316** | **4303.88** | **473.75** | **3877.71** | **4548.36** | **544.19** | **699.93** |
| **Puya** | **stenothyrsa** | **Puy** | **camterr** | **7** | **43434** | **2933.93** | **2130.58** | **7840.06** | **7880.16** | **1055.63** | **1105.40** |
| **Puya** | **thomasiana** | **Puy** | **c3terr** | **6** | **5680** | **3633.21** | **234.92** | **7454.81** | **1674.12** | **1045.67** | **430.42** |
| **Puya** | **tillii** | **Puy** | **c3terr** | **4** | **738** | **1322.04** | **2538.00** | **13548.20** | **6379.78** | **1846.60** | **996.75** |
| **Puya** | **trianae** | **Puy** | **c3terr** | **60** | **102328** | **2424.48** | **3563.80** | **14880.14** | **17241.64** | **1420.34** | **1669.33** |
| **Puya** | **tuberosa** | **Puy** | **c3terr** | **16** | **52821** | **2311.16** | **3878.75** | **4988.89** | **2912.37** | **728.82** | **508.25** |

**
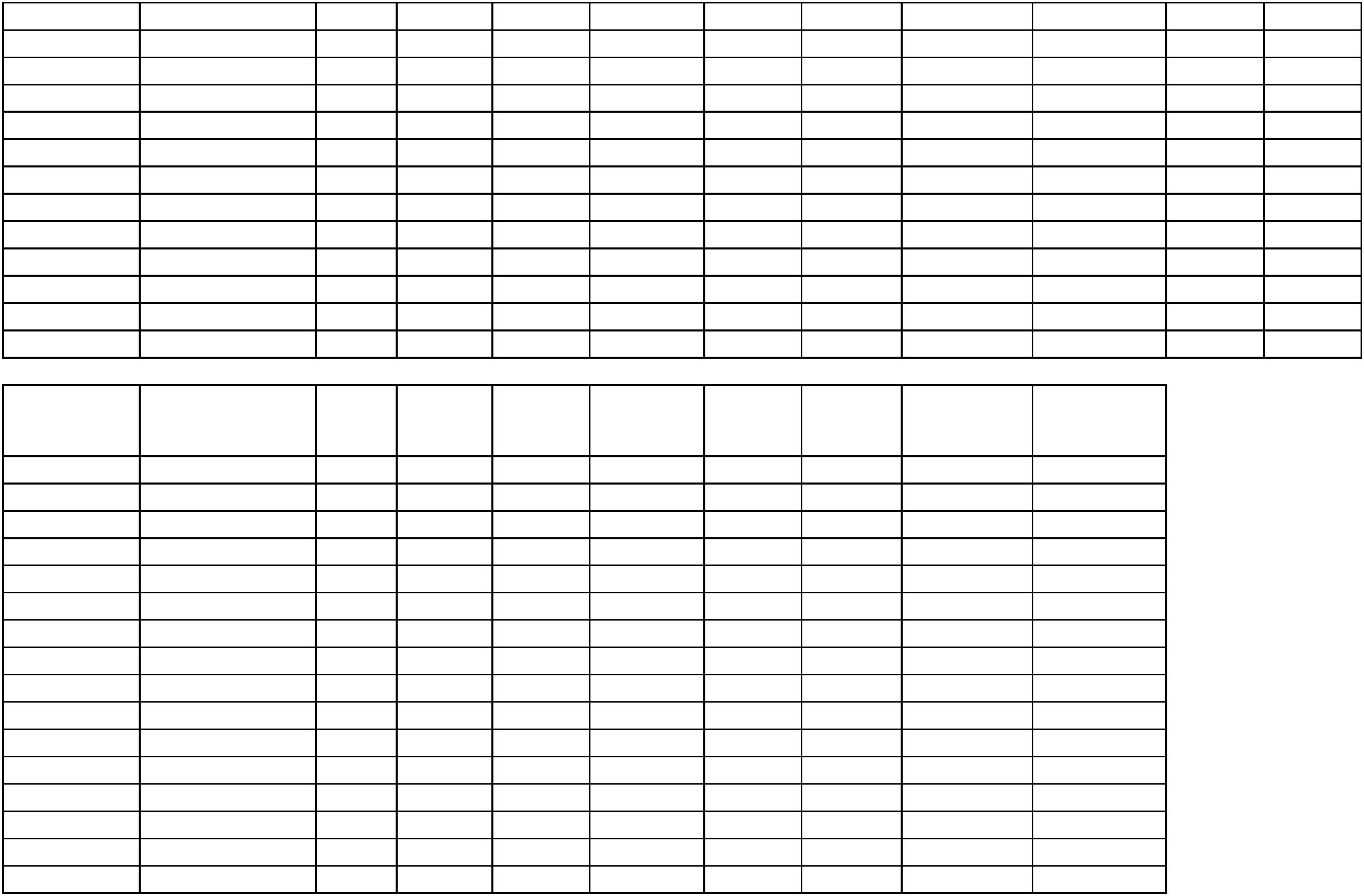
**

| **Puya** | **tunarensis** | **Puy** | **camterr** | **9** | **8356** | **2286.60** | **1803.58** | **5346.02** | **2993.98** | **723.53** | **494.50** |
| --- | --- | --- | --- | --- | --- | --- | --- | --- | --- | --- | --- |
| **Puya** | **venusta** | **Puy** | **camterr** | **11** | **20652** | **1504.14** | **2612.30** | **2764.29** | **7156.05** | **341.16** | **868.50** |
| **Puya** | **vestita** | **Puy** | **c3terr** | **3** | **130** | **1857.44** | **2563.50** | **9004.62** | **278.25** | **817.97** | **135.00** |
| **Puya** | **volcanensis** | **Puy** | **c3terr** | **5** | **8335** | **1405.48** | **4451.20** | **2036.50** | **1285.48** | **279.23** | **198.33** |
| **Puya** | **weberbaueri** | **Puy** | **c3terr** | **9** | **209447** | **1636.27** | **2170.40** | **9422.40** | **9650.13** | **1433.91** | **1958.67** |
| **Puya** | **weberiana** | **Puy** | **camterr** | **8** | **63098** | **461.76** | **1742.60** | **2111.81** | **3320.50** | **285.23** | **510.25** |
| **Puya** | **weddelliana** | **Puy** | **c3terr** | **3** | **87180** | **1486.71** | **1799.30** | **10562.94** | **12186.45** | **1650.85** | **2186.55** |
| **Puya** | **wrightii** | **Puy** | **camterr** | **3** | **63446** | **2327.13** | **2689.92** | **7296.28** | **4709.20** | **1171.17** | **895.50** |
| **Puya** | **yakespala** | **Puy** | **c3terr** | **3** | **573** | **1068.50** | **1761.00** | **1888.07** | **1223.12** | **247.02** | **224.75** |
| **Sequencia** | **serrata** | **Nav** | **c3mes** | **5** | **169** | **236.23** | **47.67** | **18656.77** | **170.60** | **3025.17** | **35.33** |
| **Steyerbromelia** | **deflexa** | **Nav** | **c3mes** | **3** | **61** | **1028.97** | **739.33** | **19528.40** | **2177.25** | **3171.50** | **64.50** |
| **Steyerbromelia** | **discolor** | **Nav** | **c3mes** | **9** | **255** | **1397.10** | **1835.42** | **20952.33** | **8014.53** | **3099.66** | **368.25** |
| **Steyerbromelia** | **ramosa** | **Nav** | **c3mes** | **4** | **14789** | **1193.10** | **1195.00** | **20847.73** | **2907.56** | **3209.92** | **249.50** |
|  |  |  |  | **Pseas (%)** | **Pseas (%)** | **Pdry (mm)** | **Pdry (mm)** | **AET/PET (mm** | **AET/PET (mm** |  |  |
| **Genus** | **Species** | **Tax Grp** | **Fun Type** | **mean** | **range** | **mean** | **range** | **mm-1) mean** | **mm-1) range** |  |  |
| **Ananas** | **ananassoides** | **CAMEDB** | **camterr** | **64.60** | **86.00** | **23.83** | **102.25** | **0.71** | **0.59** |  |  |
| **Ananas** | **bracteatus** | **CAMEDB** | **camterr** | **43.41** | **78.75** | **59.42** | **107.50** | **0.80** | **0.62** |  |  |
| **Ananas** | **lucidus** | **CAMEDB** | **camterr** | **50.88** | **83.33** | **79.47** | **209.25** | **0.84** | **0.29** |  |  |
| **Ananas** | **parguazensis** | **CAMEDB** | **camterr** | **53.49** | **62.50** | **67.55** | **194.50** | **0.85** | **0.35** |  |  |
| **Ananas** | **sagenaria** | **CAMEDB** | **camterr** | **41.40** | **71.25** | **48.96** | **101.33** | **0.71** | **0.52** |  |  |
| **Brewcaria** | **hohenbergioides** | **Nav** | **c3mes** | **61.69** | **29.75** | **46.55** | **84.33** | **0.83** | **0.20** |  |  |
| **Brewcaria** | **marahuacae** | **Nav** | **c3mes** | **43.24** | **4.75** | **102.62** | **17.75** | **0.97** | **0.06** |  |  |
| **Brewcaria** | **reflexa** | **Nav** | **c3mes** | **40.95** | **40.00** | **122.97** | **151.25** | **0.92** | **0.14** |  |  |
| **Brocchinia** | **delicatula** | **BL** | **c3mes** | **30.53** | **16.25** | **168.13** | **80.08** | **0.99** | **0.01** |  |  |
| **Brocchinia** | **hechtioides** | **BL** | **c3mes** | **46.63** | **38.50** | **80.40** | **117.33** | **0.93** | **0.25** |  |  |
| **Brocchinia** | **paniculata** | **BL** | **c3mes** | **31.94** | **48.00** | **137.45** | **208.25** | **0.95** | **0.22** |  |  |
| **Brocchinia** | **prismatica** | **BL** | **c3mes** | **51.02** | **19.00** | **79.59** | **88.00** | **0.90** | **0.11** |  |  |
| **Brocchinia** | **reducta** | **BL** | **c3mes** | **51.65** | **26.25** | **66.53** | **63.75** | **0.91** | **0.12** |  |  |
| **Brocchinia** | **rupestris** | **BL** | **c3mes** | **36.22** | **5.67** | **80.28** | **41.42** | **0.85** | **0.18** |  |  |
| **Brocchinia** | **tatei** | **BL** | **c3mes** | **47.69** | **49.83** | **88.93** | **172.58** | **0.92** | **0.20** |  |  |
| **Brocchinia** | **vestita** | **BL** | **c3mes** | **26.38** | **24.08** | **177.70** | **102.33** | **0.99** | **0.03** |  |  |


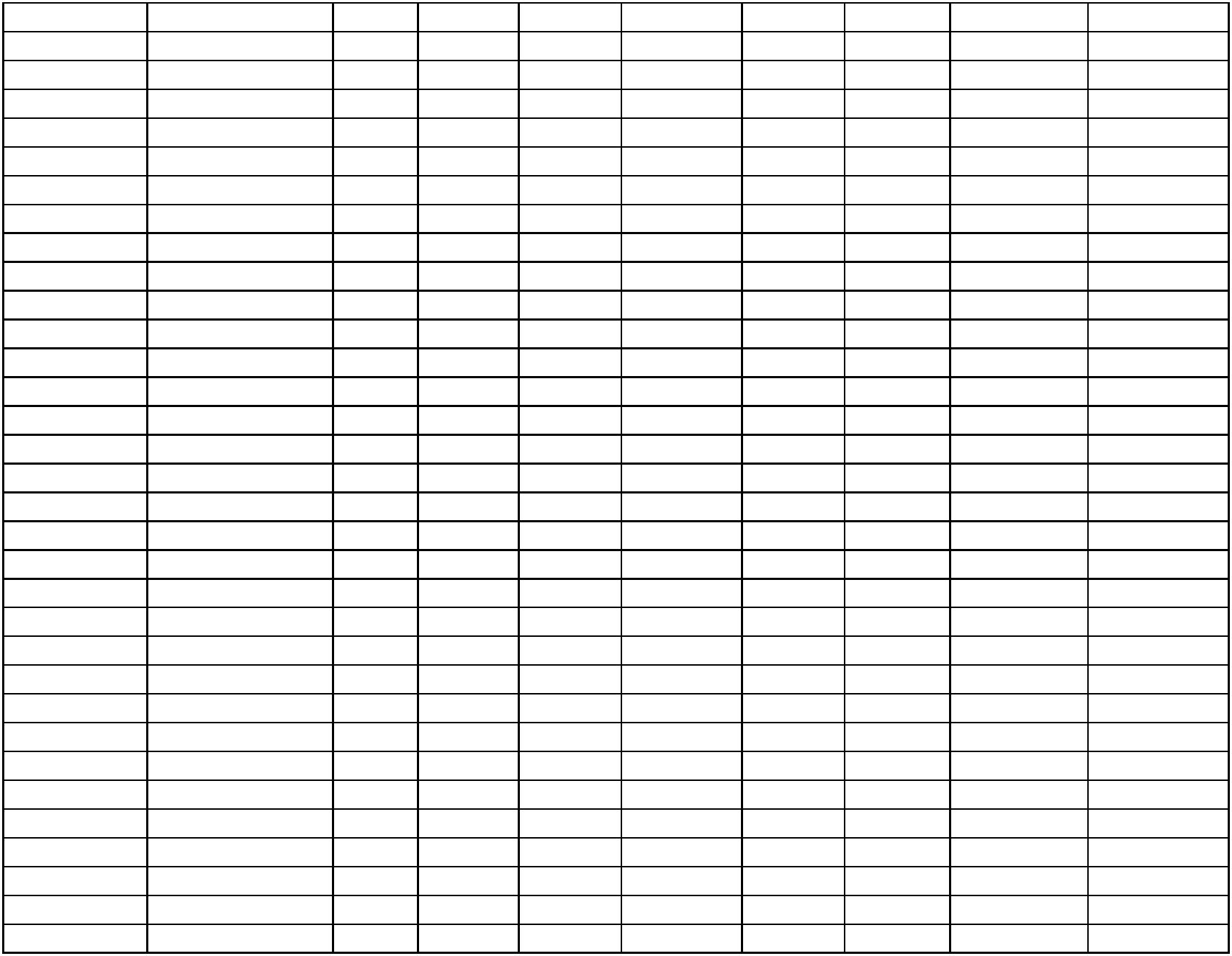


| **Bromelia** | **antiacantha** | **CAMEDB** | **camterr** | **43.82** | **74.42** | **49.40** | **100.83** | **0.74** | **0.69** |
| --- | --- | --- | --- | --- | --- | --- | --- | --- | --- |
| **Bromelia** | **arenaria** | **CAMEDB** | **camterr** | **80.95** | **56.75** | **2.79** | **11.25** | **0.34** | **0.09** |
| **Bromelia** | **balansae** | **CAMEDB** | **camterr** | **51.45** | **82.00** | **38.69** | **160.25** | **0.71** | **0.78** |
| **Bromelia** | **binotii** | **CAMEDB** | **camterr** | **41.60** | **32.33** | **55.07** | **96.67** | **0.71** | **0.55** |
| **Bromelia** | **braunii** | **CAMEDB** | **camterr** | **84.58** | **3.00** | **1.67** | **3.00** | **0.63** | **0.02** |
| **Bromelia** | **chrysantha** | **CAMEDB** | **camterr** | **64.10** | **44.83** | **15.86** | **35.83** | **0.58** | **0.33** |
| **Bromelia** | **goyazensis** | **CAMEDB** | **camterr** | **77.86** | **19.73** | **5.52** | **12.33** | **0.64** | **0.41** |
| **Bromelia** | **grandiflora** | **CAMEDB** | **camterr** | **83.69** | **18.08** | **3.53** | **0.75** | **0.65** | **0.15** |
| **Bromelia** | **hemisphaerica** | **CAMEDB** | **camterr** | **104.67** | **8.75** | **4.02** | **3.33** | **0.56** | **0.12** |
| **Bromelia** | **ignaciana** | **CAMEDB** | **camterr** | **65.72** | **10.17** | **14.89** | **5.00** | **0.62** | **0.28** |
| **Bromelia** | **interior** | **CAMEDB** | **camterr** | **77.45** | **27.70** | **7.32** | **24.92** | **0.68** | **0.25** |
| **Bromelia** | **irwinii** | **CAMEDB** | **camterr** | **79.04** | **8.00** | **4.38** | **2.00** | **0.76** | **0.08** |
| **Bromelia** | **karatas** | **CAMEDB** | **camterr** | **73.78** | **103.75** | **27.18** | **194.50** | **0.65** | **0.77** |
| **Bromelia** | **lagopus** | **CAMEDB** | **camterr** | **69.09** | **62.00** | **16.83** | **70.75** | **0.60** | **0.39** |
| **Bromelia** | **macedoi** | **CAMEDB** | **camterr** | **82.73** | **8.25** | **3.00** | **6.00** | **0.69** | **0.09** |
| **Bromelia** | **minima** | **CAMEDB** | **camterr** | **82.44** | **2.75** | **3.06** | **2.25** | **0.71** | **0.10** |
| **Bromelia** | **pinguin** | **CAMEDB** | **camterr** | **81.58** | **90.75** | **18.66** | **181.67** | **0.60** | **0.84** |
| **Bromelia** | **reversacantha** | **CAMEDB** | **camterr** | **81.06** | **8.75** | **3.15** | **0.67** | **0.69** | **0.02** |
| **Bromelia** | **serra** | **CAMEDB** | **camterr** | **62.39** | **72.83** | **22.23** | **78.00** | **0.54** | **0.68** |
| **Bromelia** | **tarapotina** | **CAMEDB** | **camterr** | **26.08** | **24.50** | **122.83** | **113.75** | **0.93** | **0.21** |
| **Bromelia** | **tubulosa** | **CAMEDB** | **camterr** | **43.87** | **36.67** | **82.98** | **109.25** | **0.88** | **0.32** |
| **Bromelia** | **unaensis** | **CAMEDB** | **camterr** | **37.45** | **51.33** | **63.51** | **84.53** | **0.78** | **0.33** |
| **Bromelia** | **villosa** | **CAMEDB** | **camterr** | **77.33** | **28.25** | **5.70** | **20.00** | **0.66** | **0.28** |
| **Connellia** | **augustae** | **BL** | **c3mes** | **52.20** | **42.50** | **54.55** | **73.65** | **0.86** | **0.16** |
| **Connellia** | **caricifolia** | **BL** | **c3mes** | **52.75** | **1.50** | **45.92** | **1.50** | **0.86** | **0.05** |
| **Connellia** | **quelchii** | **Bl** | **c3mes** | **54.99** | **25.50** | **46.95** | **40.75** | **0.87** | **0.12** |
| **Cottendorfia** | **florida** | **Nav** | **c3mes** | **56.58** | **35.00** | **20.09** | **14.92** | **0.75** | **0.58** |
| **Cryptanthus** | **bahianus** | **CAMEDB** | **camterr** | **41.48** | **41.50** | **33.33** | **80.33** | **0.53** | **0.60** |
| **Cryptanthus** | **beuckeri** | **CAMEDB** | **camterr** | **31.22** | **46.00** | **64.79** | **89.75** | **0.77** | **0.41** |
| **Cryptanthus** | **capitellatus** | **CAMEDB** | **camterr** | **57.58** | **1.25** | **32.00** | **0.75** | **0.66** | **0.00** |
| **Cryptanthus** | **dianae** | **CAMEDB** | **camterr** | **61.08** | **10.00** | **33.57** | **22.00** | **0.71** | **0.13** |
| **Cryptanthus** | **ferrarius** | **CAMEDB** | **camterr** | **82.35** | **4.58** | **13.90** | **3.08** | **0.77** | **0.05** |
| **Cryptanthus** | **giganteus** | **CAMEDB** | **camterr** | **59.08** | **1.25** | **30.58** | **1.75** | **0.64** | **0.65** |

**
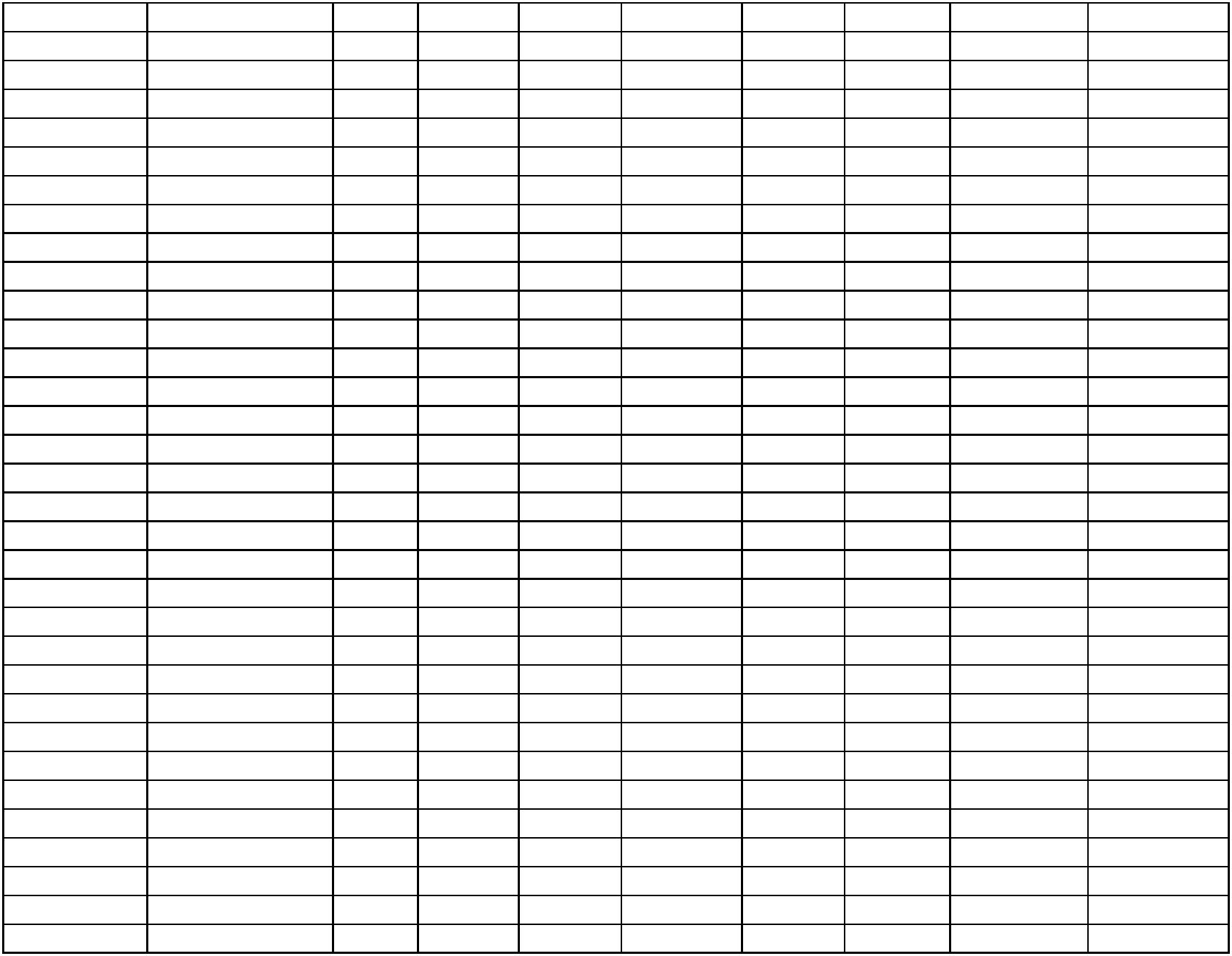
**

| **Cryptanthus** | **maritimus** | **CAMEDB** | **camterr** | **32.79** | **34.42** | **67.44** | **62.75** | **0.76** | **0.31** |
| --- | --- | --- | --- | --- | --- | --- | --- | --- | --- |
| **Cryptanthus** | **micrus** | **CAMEDB** | **camterr** | **86.31** | **0.92** | **8.86** | **1.17** | **0.71** | **0.73** |
| **Cryptanthus** | **pickelii** | **CAMEDB** | **camterr** | **60.99** | **10.33** | **37.42** | **24.58** | **0.74** | **0.24** |
| **Cryptanthus** | **pseudopetiolatus** | **CAMEDB** | **camterr** | **17.89** | **27.17** | **110.87** | **81.65** | **0.94** | **0.33** |
| **Cryptanthus** | **sanctaluciae** | **CAMEDB** | **camterr** | **51.03** | **1.93** | **40.25** | **1.75** | **0.72** | **0.12** |
| **Cryptanthus** | **schwackeanus** | **CAMEDB** | **c3terr** | **83.64** | **11.00** | **12.52** | **8.00** | **0.75** | **0.21** |
| **Cryptanthus** | **sergipensis** | **CAMEDB** | **camterr** | **54.60** | **11.67** | **47.54** | **38.00** | **0.67** | **0.33** |
| **Cryptanthus** | **venecianus** | **CAMEDB** | **camterr** | **55.17** | **0.83** | **33.14** | **0.92** | **0.61** | **0.61** |
| **Cryptanthus** | **warren-loosei** | **CAMEDB** | **camterr** | **40.88** | **21.00** | **40.13** | **55.50** | **0.55** | **0.53** |
| **Cryptanthus** | **zonatus** | **CAMEDB** | **camterr** | **53.40** | **33.08** | **34.33** | **18.67** | **0.68** | **0.09** |
| **Deinacanthon** | **urbanianum** | **CAMEDB** | **camterr** | **65.99** | **34.80** | **8.37** | **12.25** | **0.30** | **0.31** |
| **Deuterocohnia** | **brevifolia** | **XC** | **camterr** | **105.25** | **22.00** | **1.19** | **1.75** | **0.23** | **0.20** |
| **Deuterocohnia** | **haumanii** | **XC** | **camterr** | **109.25** | **27.42** | **0.60** | **2.25** | **0.15** | **0.28** |
| **Deuterocohnia** | **longipetala** | **XC** | **camterr** | **82.29** | **66.25** | **6.63** | **23.75** | **0.32** | **0.54** |
| **Deuterocohnia** | **lorentziana** | **XC** | **camterr** | **103.73** | **22.25** | **1.21** | **4.25** | **0.16** | **0.14** |
| **Deuterocohnia** | **meziana** | **XC** | **camterr** | **60.75** | **46.50** | **19.95** | **41.50** | **0.46** | **0.42** |
| **Deuterocohnia** | **scapigera** | **XC** | **camterr** | **85.19** | **21.92** | **5.42** | **8.00** | **0.36** | **0.26** |
| **Deuterocohnia** | **schreiteri** | **XC** | **camterr** | **103.20** | **48.10** | **1.28** | **4.25** | **0.15** | **0.21** |
| **Deuterocohnia** | **seramisiana** | **XC** | **camterr** | **92.75** | **1.25** | **3.06** | **1.67** | **0.34** | **0.03** |
| **Deuterocohnia** | **strobilifera** | **XC** | **camterr** | **99.54** | **79.25** | **9.79** | **97.60** | **0.28** | **0.86** |
| **Disteganthus** | **basilateralis** | **CAMEDB** | **camterr** | **47.71** | **11.25** | **81.25** | **42.25** | **0.92** | **0.08** |
| **Disteganthus** | **calatheoides** | **CAMEDB** | **camterr** | **50.58** | **11.83** | **66.50** | **45.83** | **0.91** | **0.10** |
| **Disteganthus** | **lateralis** | **CAMEDB** | **camterr** | **47.48** | **20.08** | **65.19** | **73.33** | **0.89** | **0.17** |
| **Dyckia** | **affinis** | **XC** | **camterr** | **35.25** | **7.25** | **45.75** | **36.75** | **0.72** | **0.24** |
| **Dyckia** | **aurea** | **XC** | **camterr** | **75.84** | **14.95** | **8.15** | **9.00** | **0.70** | **0.07** |
| **Dyckia** | **brachyphylla** | **XC** | **camterr** | **85.03** | **3.95** | **8.09** | **2.45** | **0.76** | **0.04** |
| **Dyckia** | **brasiliana** | **XC** | **camterr** | **77.70** | **11.00** | **5.30** | **7.50** | **0.72** | **0.16** |
| **Dyckia** | **brevifolia** | **XC** | **camterr** | **25.34** | **34.25** | **88.47** | **90.50** | **0.90** | **0.25** |
| **Dyckia** | **burchellii** | **XC** | **camterr** | **78.79** | **29.00** | **6.40** | **24.25** | **0.64** | **0.32** |
| **Dyckia** | **consimilis** | **XC** | **camterr** | **83.76** | **12.33** | **11.45** | **10.83** | **0.74** | **0.03** |
| **Dyckia** | **dawsonii** | **XC** | **camterr** | **83.58** | **4.75** | **4.00** | **0.10** | **0.69** | **0.07** |
| **Dyckia** | **distachya** | **XC** | **camterr** | **17.16** | **22.75** | **102.00** | **71.50** | **0.87** | **0.16** |
| **Dyckia** | **duckei** | **XC** | **camterr** | **66.73** | **7.50** | **14.58** | **11.50** | **0.73** | **0.04** |

**
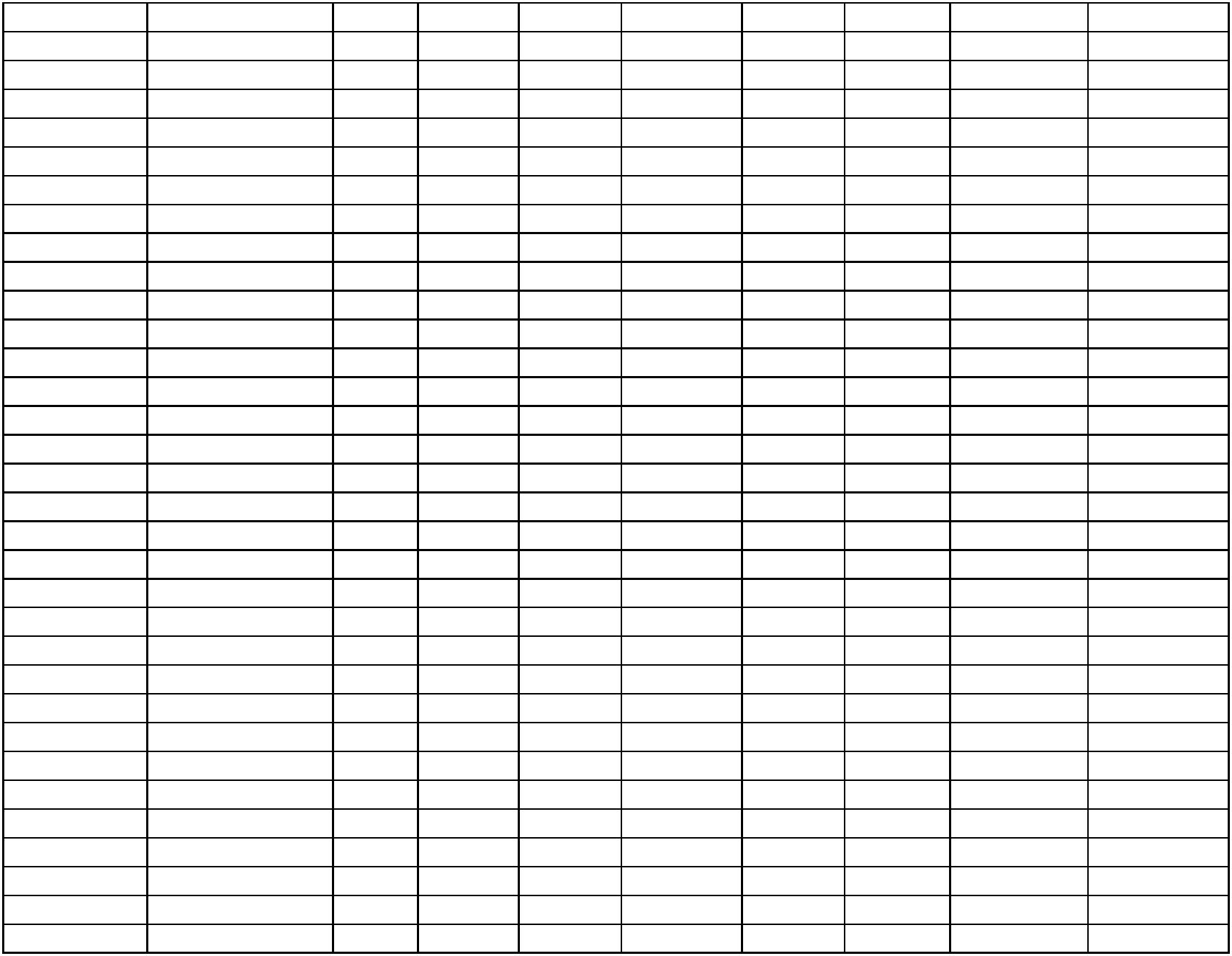
**

| **Dyckia** | **dusenii** | **XC** | **camterr** | **28.56** | **6.83** | **79.13** | **16.25** | **0.93** | **0.15** |
| --- | --- | --- | --- | --- | --- | --- | --- | --- | --- |
| **Dyckia** | **elata** | **XC** | **camterr** | **82.42** | **0.25** | **13.92** | **0.25** | **0.77** | **0.02** |
| **Dyckia** | **encholirioides** | **XC** | **camterr** | **42.11** | **57.00** | **59.54** | **81.42** | **0.87** | **0.27** |
| **Dyckia** | **exserta** | **XC** | **camterr** | **34.08** | **0.25** | **43.00** | **0.10** | **0.73** | **0.01** |
| **Dyckia** | **ferox** | **XC** | **camterr** | **37.69** | **50.50** | **45.42** | **92.00** | **0.69** | **0.58** |
| **Dyckia** | **ferruginea** | **XC** | **camterr** | **58.45** | **18.75** | **27.55** | **18.25** | **0.67** | **0.18** |
| **Dyckia** | **floribunda** | **XC** | **camterr** | **61.30** | **90.00** | **21.68** | **55.25** | **0.47** | **0.69** |
| **Dyckia** | **fosteriana** | **XC** | **camterr** | **33.50** | **14.75** | **59.50** | **18.50** | **0.79** | **0.10** |
| **Dyckia** | **goiana** | **XC** | **camterr** | **81.00** | **12.75** | **4.98** | **3.50** | **0.70** | **0.06** |
| **Dyckia** | **gracilis** | **XC** | **camterr** | **56.38** | **16.75** | **42.13** | **100.25** | **0.65** | **0.52** |
| **Dyckia** | **grandidentata** | **XC** | **camterr** | **59.42** | **4.25** | **29.48** | **6.35** | **0.73** | **0.03** |
| **Dyckia** | **horridula** | **XC** | **camterr** | **72.72** | **15.00** | **6.43** | **7.75** | **0.72** | **0.05** |
| **Dyckia** | **ibiramensis** | **XC** | **camterr** | **20.98** | **0.70** | **87.97** | **0.85** | **0.92** | **0.01** |
| **Dyckia** | **leptostachya** | **XC** | **camterr** | **47.25** | **73.67** | **41.18** | **98.25** | **0.69** | **0.68** |
| **Dyckia** | **limae** | **XC** | **camterr** | **57.47** | **9.75** | **17.88** | **27.25** | **0.48** | **0.21** |
| **Dyckia** | **linearifolia** | **XC** | **camterr** | **56.03** | **30.50** | **36.32** | **29.50** | **0.73** | **0.07** |
| **Dyckia** | **macedoi** | **XC** | **camterr** | **84.51** | **6.67** | **7.74** | **7.50** | **0.74** | **0.05** |
| **Dyckia** | **maracasensis** | **XC** | **camterr** | **43.33** | **1.00** | **21.89** | **4.17** | **0.46** | **0.11** |
| **Dyckia** | **maritima** | **XC** | **camterr** | **11.80** | **10.25** | **97.89** | **53.20** | **0.90** | **0.15** |
| **Dyckia** | **marnier-lapostollei** | **XC** | **camterr** | **76.38** | **13.00** | **7.63** | **9.00** | **0.74** | **0.06** |
| **Dyckia** | **microcalyx** | **XC** | **camterr** | **26.23** | **24.25** | **66.54** | **76.25** | **0.78** | **0.33** |
| **Dyckia** | **minarum** | **XC** | **camterr** | **64.47** | **61.50** | **31.99** | **73.25** | **0.77** | **0.32** |
| **Dyckia** | **niederleinii** | **XC** | **camterr** | **18.59** | **10.00** | **100.96** | **28.00** | **0.86** | **0.10** |
| **Dyckia** | **pernambucana** | **XC** | **camterr** | **75.10** | **20.75** | **9.73** | **13.67** | **0.52** | **0.15** |
| **Dyckia** | **pulquinensis** | **XC** | **camterr** | **70.08** | **32.00** | **37.39** | **91.27** | **0.51** | **0.68** |
| **Dyckia** | **pumila** | **XC** | **camterr** | **73.40** | **21.00** | **9.35** | **14.25** | **0.71** | **0.09** |
| **Dyckia** | **racemosa** | **XC** | **camterr** | **82.00** | **6.00** | **3.58** | **5.50** | **0.66** | **0.08** |
| **Dyckia** | **ragonesei** | **XC** | **camterr** | **39.50** | **27.25** | **40.08** | **69.25** | **0.62** | **0.55** |
| **Dyckia** | **rariflora** | **XC** | **camterr** | **84.33** | **7.00** | **11.32** | **7.75** | **0.74** | **0.09** |
| **Dyckia** | **reitzii** | **XC** | **camterr** | **26.78** | **26.00** | **87.56** | **61.75** | **0.95** | **0.18** |
| **Dyckia** | **remotiflora** | **XC** | **camterr** | **13.67** | **15.25** | **87.25** | **48.00** | **0.80** | **0.11** |
| **Dyckia** | **saxatilis** | **XC** | **camterr** | **79.02** | **29.75** | **13.87** | **27.50** | **0.73** | **0.31** |
| **Dyckia** | **sordida** | **XC** | **camterr** | **84.73** | **4.58** | **9.23** | **3.42** | **0.75** | **0.06** |

**
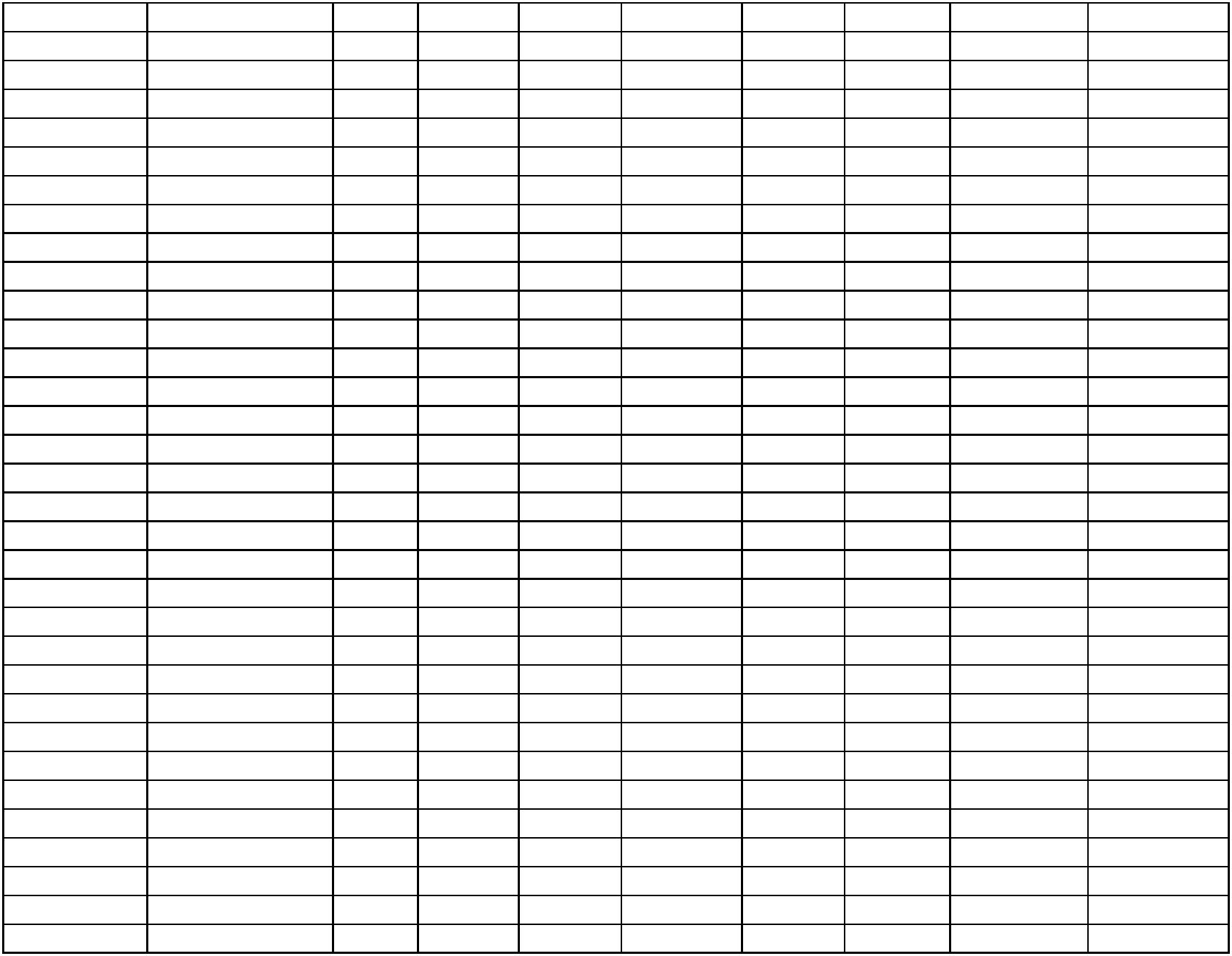
**

| **Dyckia** | **stenophylla** | **XC** | **camterr** | **52.32** | **72.83** | **68.68** | **177.58** | **0.84** | **0.35** |
| --- | --- | --- | --- | --- | --- | --- | --- | --- | --- |
| **Dyckia** | **tenebrosa** | **XC** | **camterr** | **84.03** | **4.10** | **13.62** | **3.15** | **0.75** | **0.03** |
| **Dyckia** | **tobatiensis** | **XC** | **camterr** | **35.12** | **6.50** | **44.22** | **23.00** | **0.75** | **0.22** |
| **Dyckia** | **trichostachya** | **XC** | **camterr** | **61.76** | **32.90** | **27.94** | **35.25** | **0.70** | **0.17** |
| **Dyckia** | **tuberosa** | **XC** | **camterr** | **47.79** | **76.00** | **48.39** | **114.75** | **0.78** | **0.42** |
| **Dyckia** | **uleana** | **XC** | **camterr** | **82.50** | **6.00** | **4.75** | **7.00** | **0.65** | **0.02** |
| **Dyckia** | **ursina** | **XC** | **camterr** | **88.50** | **1.50** | **7.42** | **0.25** | **0.70** | **0.02** |
| **Dyckia** | **velascana** | **XC** | **camterr** | **95.39** | **42.00** | **2.38** | **8.25** | **0.19** | **0.20** |
| **Dyckia** | **weddelliana** | **XC** | **camterr** | **67.09** | **36.75** | **25.08** | **95.85** | **0.69** | **0.49** |
| **Encholirium** | **agavoides** | **XC** | **camterr** | **83.19** | **0.75** | **8.56** | **0.75** | **0.75** | **0.04** |
| **Encholirium** | **biflorum** | **XC** | **camterr** | **86.08** | **0.75** | **8.58** | **0.75** | **0.77** | **0.01** |
| **Encholirium** | **brachypodum** | **XC** | **camterr** | **75.89** | **54.17** | **8.97** | **28.00** | **0.40** | **0.23** |
| **Encholirium** | **ctenophyllum** | **XC** | **camterr** | **83.75** | **2.25** | **8.10** | **2.00** | **0.72** | **0.18** |
| **Encholirium** | **disjunctum** | **XC** | **camterr** | **82.58** | **7.00** | **2.58** | **4.00** | **0.60** | **0.26** |
| **Encholirium** | **erectiflorum** | **XC** | **camterr** | **88.67** | **40.67** | **7.83** | **8.00** | **0.49** | **0.18** |
| **Encholirium** | **gracile** | **XC** | **camterr** | **62.54** | **9.00** | **26.46** | **7.75** | **0.58** | **0.08** |
| **Encholirium** | **heloisae** | **XC** | **camterr** | **85.86** | **6.75** | **8.33** | **2.00** | **0.74** | **0.09** |
| **Encholirium** | **horridum** | **XC** | **camterr** | **60.59** | **47.83** | **28.40** | **38.50** | **0.62** | **0.23** |
| **Encholirium** | **irwinii** | **XC** | **camterr** | **89.19** | **5.00** | **5.31** | **1.25** | **0.51** | **0.08** |
| **Encholirium** | **longiflorum** | **XC** | **camterr** | **91.17** | **0.50** | **0.58** | **1.75** | **0.45** | **0.03** |
| **Encholirium** | **luxor** | **XC** | **camterr** | **82.14** | **20.25** | **4.09** | **6.75** | **0.62** | **0.40** |
| **Encholirium** | **lymanianum** | **XC** | **camterr** | **49.63** | **8.00** | **31.44** | **5.75** | **0.71** | **0.01** |
| **Encholirium** | **magalhaensis** | **XC** | **camterr** | **85.81** | **2.00** | **8.13** | **2.50** | **0.77** | **0.04** |
| **Encholirium** | **maximum** | **XC** | **camterr** | **86.75** | **5.25** | **0.75** | **1.75** | **0.35** | **0.05** |
| **Encholirium** | **scrutor** | **XC** | **camterr** | **86.25** | **0.50** | **8.13** | **0.75** | **0.75** | **0.03** |
| **Encholirium** | **spectabile** | **XC** | **camterr** | **70.25** | **106.17** | **12.32** | **106.25** | **0.38** | **0.79** |
| **Encholirium** | **subsecundum** | **XC** | **camterr** | **82.62** | **47.17** | **9.11** | **16.67** | **0.70** | **0.43** |
| **Fascicularia** | **bicolor** | **C3EDB** | **c3terr** | **57.46** | **55.83** | **59.62** | **89.00** | **0.84** | **0.49** |
| **Fernseea** | **itatiaiae** | **C3EDB** | **c3terr** | **66.15** | **44.25** | **30.42** | **16.00** | **0.83** | **0.31** |
| **Fosterella** | **albicans** | **PF** | **c3mes** | **79.97** | **42.25** | **12.18** | **53.75** | **0.49** | **0.53** |
| **Fosterella** | **caulescens** | **PF** | **c3mes** | **54.17** | **4.00** | **39.50** | **17.67** | **0.84** | **0.04** |
| **Fosterella** | **chaparensis** | **PF** | **c3mes** | **61.76** | **17.25** | **53.87** | **103.50** | **0.77** | **0.42** |
| **Fosterella** | **christophii** | **PF** | **c3mes** | **51.94** | **10.33** | **29.42** | **28.00** | **0.62** | **0.27** |

**
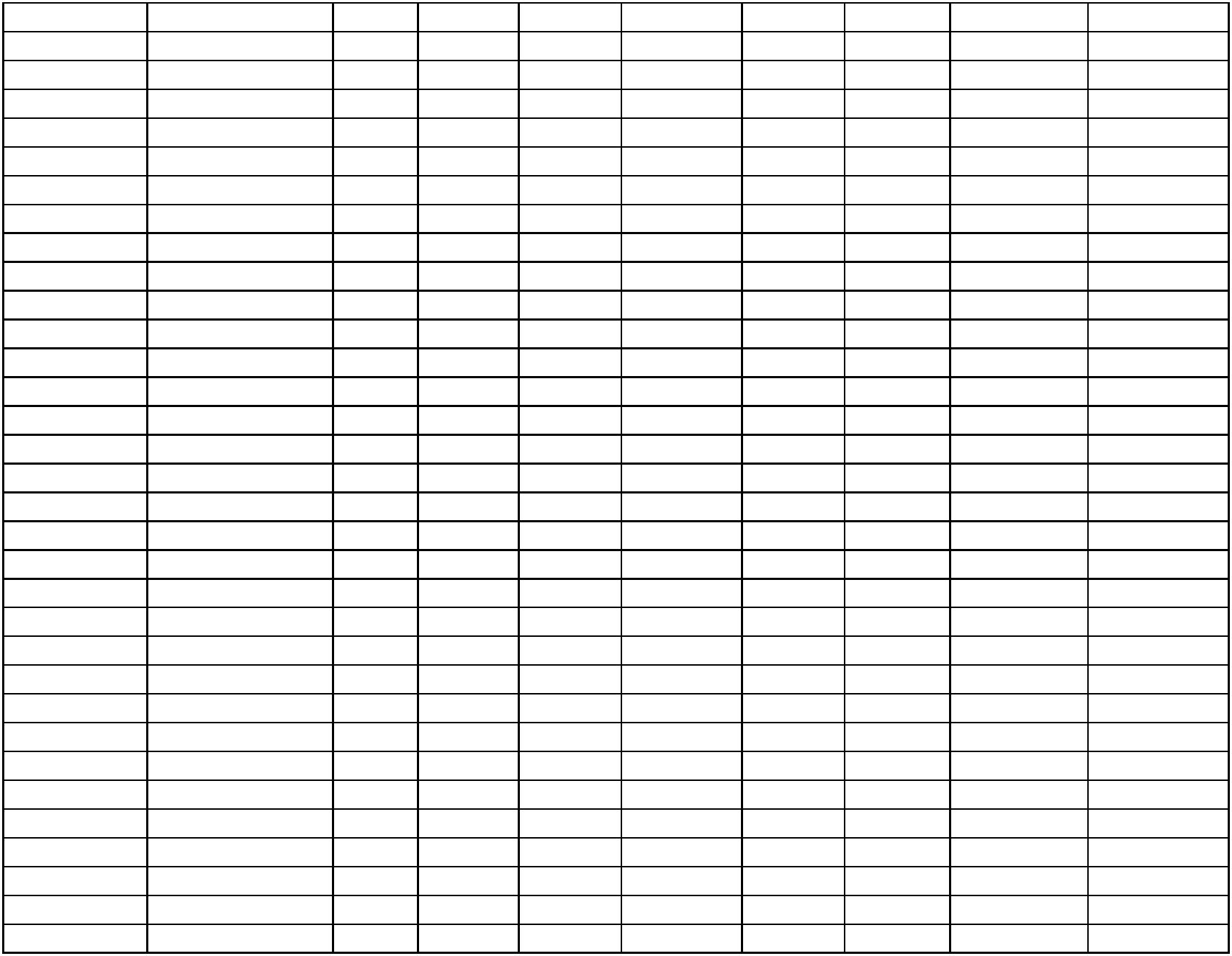
**

| **Fosterella** | **cotacajensis** | **PF** | **c3mes** | **68.95** | **16.75** | **23.09** | **29.50** | **0.65** | **0.30** |
| --- | --- | --- | --- | --- | --- | --- | --- | --- | --- |
| **Fosterella** | **gracilis** | **PF** | **c3mes** | **60.43** | **37.83** | **38.83** | **60.33** | **0.73** | **0.42** |
| **Fosterella** | **graminea** | **PF** | **c3mes** | **60.95** | **20.00** | **24.85** | **38.00** | **0.70** | **0.31** |
| **Fosterella** | **hatschbachii** | **PF** | **c3mes** | **54.73** | **18.75** | **28.70** | **23.00** | **0.71** | **0.06** |
| **Fosterella** | **heterophylla** | **PF** | **c3mes** | **60.00** | **11.25** | **25.17** | **15.75** | **0.71** | **0.17** |
| **Fosterella** | **kroemeri** | **PF** | **c3mes** | **59.17** | **5.50** | **25.58** | **11.50** | **0.71** | **0.13** |
| **Fosterella** | **micrantha** | **PF** | **c3mes** | **84.54** | **60.00** | **26.77** | **82.33** | **0.71** | **0.52** |
| **Fosterella** | **nicoliana** | **PF** | **c3mes** | **41.08** | **25.33** | **72.00** | **77.33** | **0.83** | **0.31** |
| **Fosterella** | **pearcei** | **PF** | **c3mes** | **36.26** | **59.00** | **117.74** | **235.67** | **0.91** | **0.63** |
| **Fosterella** | **penduliflora** | **PF** | **c3mes** | **71.75** | **56.50** | **15.36** | **44.50** | **0.53** | **0.64** |
| **Fosterella** | **petiolata** | **PF** | **c3mes** | **65.89** | **16.25** | **17.29** | **14.75** | **0.65** | **0.24** |
| **Fosterella** | **robertreadii** | **PF** | **c3mes** | **59.27** | **28.33** | **28.29** | **41.33** | **0.64** | **0.22** |
| **Fosterella** | **rojasii** | **PF** | **c3mes** | **37.43** | **25.25** | **41.18** | **20.25** | **0.73** | **0.03** |
| **Fosterella** | **rusbyi** | **PF** | **c3mes** | **59.58** | **22.50** | **31.86** | **84.85** | **0.73** | **0.42** |
| **Fosterella** | **schidosperma** | **PF** | **c3mes** | **57.14** | **43.00** | **42.11** | **78.58** | **0.76** | **0.40** |
| **Fosterella** | **vasquezii** | **PF** | **c3mes** | **67.54** | **1.00** | **13.50** | **1.00** | **0.67** | **0.05** |
| **Fosterella** | **villosula** | **PF** | **c3mes** | **55.30** | **12.25** | **55.72** | **86.08** | **0.85** | **0.28** |
| **Fosterella** | **weberbaueri** | **PF** | **c3mes** | **55.28** | **30.00** | **47.44** | **93.67** | **0.74** | **0.58** |
| **Fosterella** | **weddelliana** | **PF** | **c3mes** | **63.60** | **25.75** | **27.94** | **79.75** | **0.68** | **0.45** |
| **Fosterella** | **windischii** | **PF** | **c3mes** | **67.69** | **7.25** | **15.63** | **10.75** | **0.67** | **0.26** |
| **Fosterella** | **yuvinkae** | **PF** | **c3mes** | **51.30** | **3.75** | **36.07** | **79.60** | **0.61** | **0.48** |
| **Greigia** | **alborosea** | **C3EDB** | **c3terr** | **50.97** | **45.17** | **31.48** | **28.92** | **0.75** | **0.13** |
| **Greigia** | **columbiana** | **C3EDB** | **c3terr** | **49.47** | **46.00** | **53.09** | **69.75** | **0.92** | **0.23** |
| **Greigia** | **danielii** | **C3EDB** | **c3terr** | **38.14** | **18.00** | **56.12** | **97.50** | **0.89** | **0.26** |
| **Greigia** | **kessleri** | **C3EDB** | **c3terr** | **73.11** | **8.00** | **11.92** | **3.25** | **0.59** | **0.24** |
| **Greigia** | **leymebambana** | **C3EDB** | **c3terr** | **55.29** | **26.00** | **28.38** | **22.75** | **0.68** | **0.20** |
| **Greigia** | **mulfordii** | **C3EDB** | **c3terr** | **31.65** | **38.00** | **58.19** | **152.58** | **0.84** | **0.38** |
| **Greigia** | **nubigena** | **C3EDB** | **c3terr** | **53.85** | **52.25** | **27.82** | **43.25** | **0.62** | **0.28** |
| **Greigia** | **oaxacana** | **C3EDB** | **c3terr** | **73.61** | **29.75** | **43.41** | **35.00** | **0.83** | **0.14** |
| **Greigia** | **rohwederi** | **C3EDB** | **c3terr** | **80.53** | **19.83** | **14.72** | **21.08** | **0.75** | **0.17** |
| **Greigia** | **sanctae-martae** | **C3EDB** | **c3terr** | **51.61** | **33.00** | **39.92** | **8.50** | **0.90** | **0.07** |
| **Greigia** | **sodiroana** | **C3EDB** | **c3terr** | **31.29** | **22.75** | **75.84** | **269.67** | **0.73** | **0.49** |
| **Greigia** | **sphacelata** | **C3EDB** | **c3terr** | **69.14** | **44.40** | **39.48** | **68.85** | **0.73** | **0.44** |

**
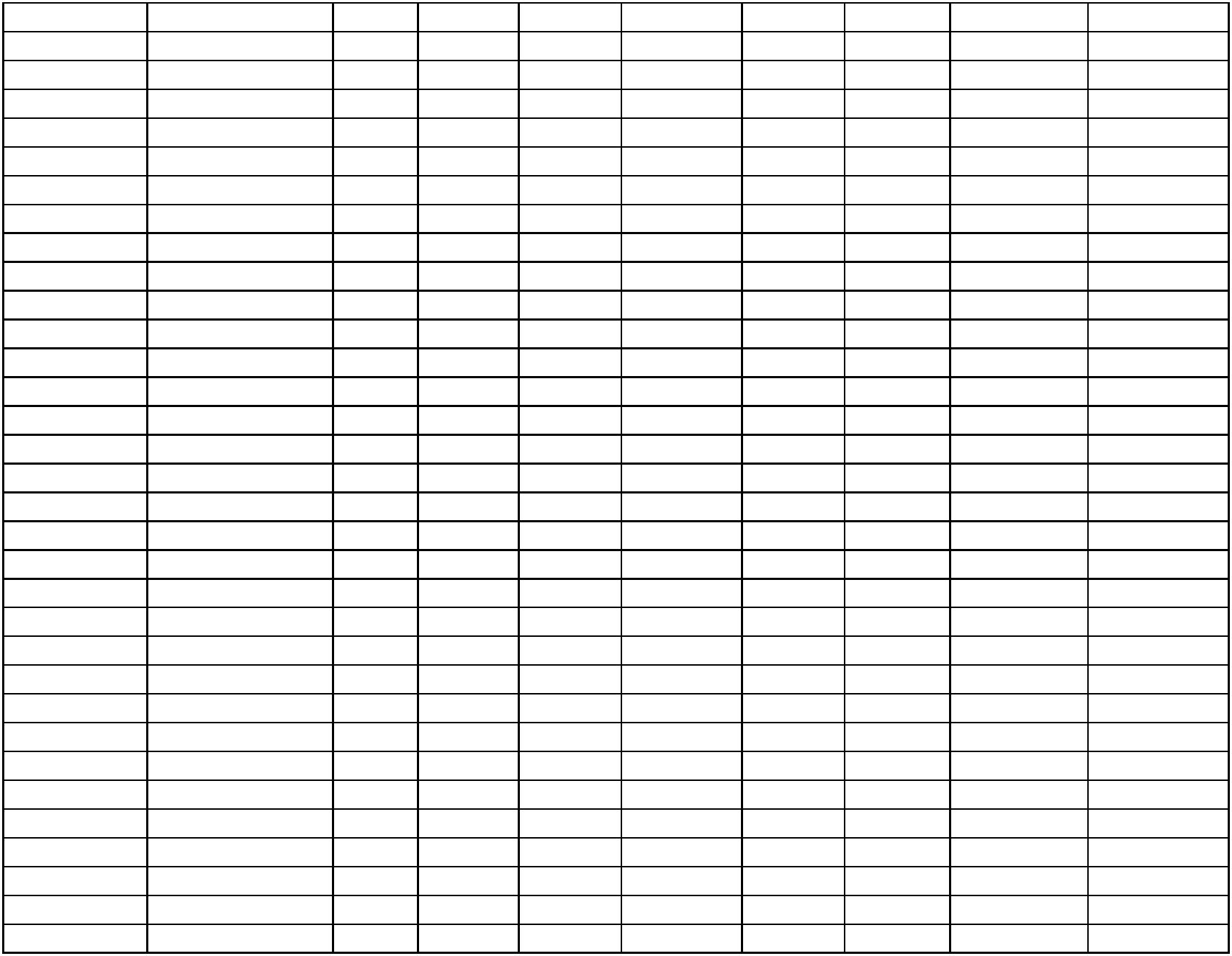
**

| **Greigia** | **stenolepis** | **C3EDB** | **c3terr** | **47.93** | **39.00** | **31.65** | **38.83** | **0.81** | **0.47** |
| --- | --- | --- | --- | --- | --- | --- | --- | --- | --- |
| **Greigia** | **steyermarkii** | **C3EDB** | **c3terr** | **53.58** | **51.00** | **61.88** | **101.25** | **0.88** | **0.31** |
| **Greigia** | **sylvicola** | **C3EDB** | **c3terr** | **54.76** | **55.25** | **59.30** | **132.75** | **0.94** | **0.32** |
| **Greigia** | **van-hyningii** | **C3EDB** | **c3terr** | **75.21** | **19.00** | **31.22** | **36.75** | **0.71** | **0.48** |
| **Greigia** | **vilcabambae** | **C3EDB** | **c3terr** | **50.88** | **14.25** | **32.88** | **33.50** | **0.63** | **0.25** |
| **Greigia** | **vulcanica** | **C3EDB** | **c3terr** | **31.27** | **60.08** | **75.05** | **193.75** | **0.88** | **0.39** |
| **Hechtia** | **bracteata** | **Hec** | **camterr** | **76.46** | **10.75** | **10.19** | **29.00** | **0.33** | **0.59** |
| **Hechtia** | **caudata** | **Hec** | **camterr** | **108.46** | **14.25** | **3.39** | **5.50** | **0.44** | **0.12** |
| **Hechtia** | **confusa** | **Hec** | **camterr** | **86.58** | **10.00** | **8.33** | **10.00** | **0.39** | **0.19** |
| **Hechtia** | **conzattiana** | **Hec** | **camterr** | **94.21** | **13.33** | **5.94** | **13.17** | **0.29** | **0.38** |
| **Hechtia** | **fragilis** | **Hec** | **camterr** | **91.67** | **8.33** | **8.85** | **9.92** | **0.38** | **0.28** |
| **Hechtia** | **galeottii** | **Hec** | **camterr** | **93.30** | **11.92** | **6.17** | **10.25** | **0.33** | **0.28** |
| **Hechtia** | **glabra** | **Hec** | **camterr** | **85.15** | **20.50** | **22.35** | **25.25** | **0.61** | **0.25** |
| **Hechtia** | **glomerata** | **Hec** | **camterr** | **79.65** | **43.00** | **12.17** | **20.25** | **0.40** | **0.57** |
| **Hechtia** | **guatemalensis** | **Hec** | **camterr** | **82.89** | **38.25** | **9.24** | **21.83** | **0.61** | **0.32** |
| **Hechtia** | **jaliscana** | **Hec** | **camterr** | **109.45** | **17.50** | **3.60** | **4.75** | **0.41** | **0.14** |
| **Hechtia** | **laevis** | **Hec** | **camterr** | **108.90** | **1.50** | **1.60** | **1.00** | **0.39** | **0.04** |
| **Hechtia** | **liebmannii** | **Hec** | **camterr** | **92.10** | **9.50** | **3.85** | **2.75** | **0.26** | **0.10** |
| **Hechtia** | **lundelliorum** | **Hec** | **camterr** | **80.18** | **20.40** | **33.96** | **37.45** | **0.68** | **0.30** |
| **Hechtia** | **lyman-smithii** | **Hec** | **camterr** | **96.15** | **1.50** | **3.20** | **2.75** | **0.19** | **0.09** |
| **Hechtia** | **montana** | **Hec** | **camterr** | **102.24** | **60.50** | **2.92** | **8.75** | **0.21** | **0.28** |
| **Hechtia** | **pedicellata** | **Hec** | **camterr** | **111.31** | **1.25** | **4.88** | **0.50** | **0.44** | **0.03** |
| **Hechtia** | **podantha** | **Hec** | **camterr** | **91.59** | **36.35** | **4.99** | **17.83** | **0.29** | **0.52** |
| **Hechtia** | **pringlei** | **Hec** | **camterr** | **93.76** | **8.58** | **4.62** | **4.00** | **0.32** | **0.18** |
| **Hechtia** | **purpusii** | **Hec** | **camterr** | **75.42** | **4.00** | **29.17** | **36.25** | **0.58** | **0.60** |
| **Hechtia** | **rosea** | **Hec** | **camterr** | **106.88** | **20.33** | **3.24** | **6.33** | **0.44** | **0.32** |
| **Hechtia** | **schottii** | **Hec** | **camterr** | **74.02** | **36.25** | **21.15** | **32.25** | **0.56** | **0.33** |
| **Hechtia** | **sphaeroblasta** | **Hec** | **camterr** | **96.42** | **11.00** | **4.58** | **2.00** | **0.34** | **0.23** |
| **Hechtia** | **stenopetala** | **Hec** | **camterr** | **82.37** | **26.25** | **25.01** | **40.75** | **0.59** | **0.39** |
| **Hechtia** | **subalata** | **Hec** | **camterr** | **111.25** | **11.75** | **2.58** | **4.00** | **0.40** | **0.32** |
| **Hechtia** | **texensis** | **Hec** | **camterr** | **72.95** | **24.00** | **8.44** | **17.33** | **0.23** | **0.47** |
| **Hechtia** | **tillandsioides** | **Hec** | **camterr** | **80.75** | **17.50** | **31.20** | **37.00** | **0.67** | **0.27** |
| **Lindmania** | **arachnoidea** | **BL** | **c3mes** | **47.24** | **44.50** | **94.20** | **97.75** | **0.97** | **0.05** |

**
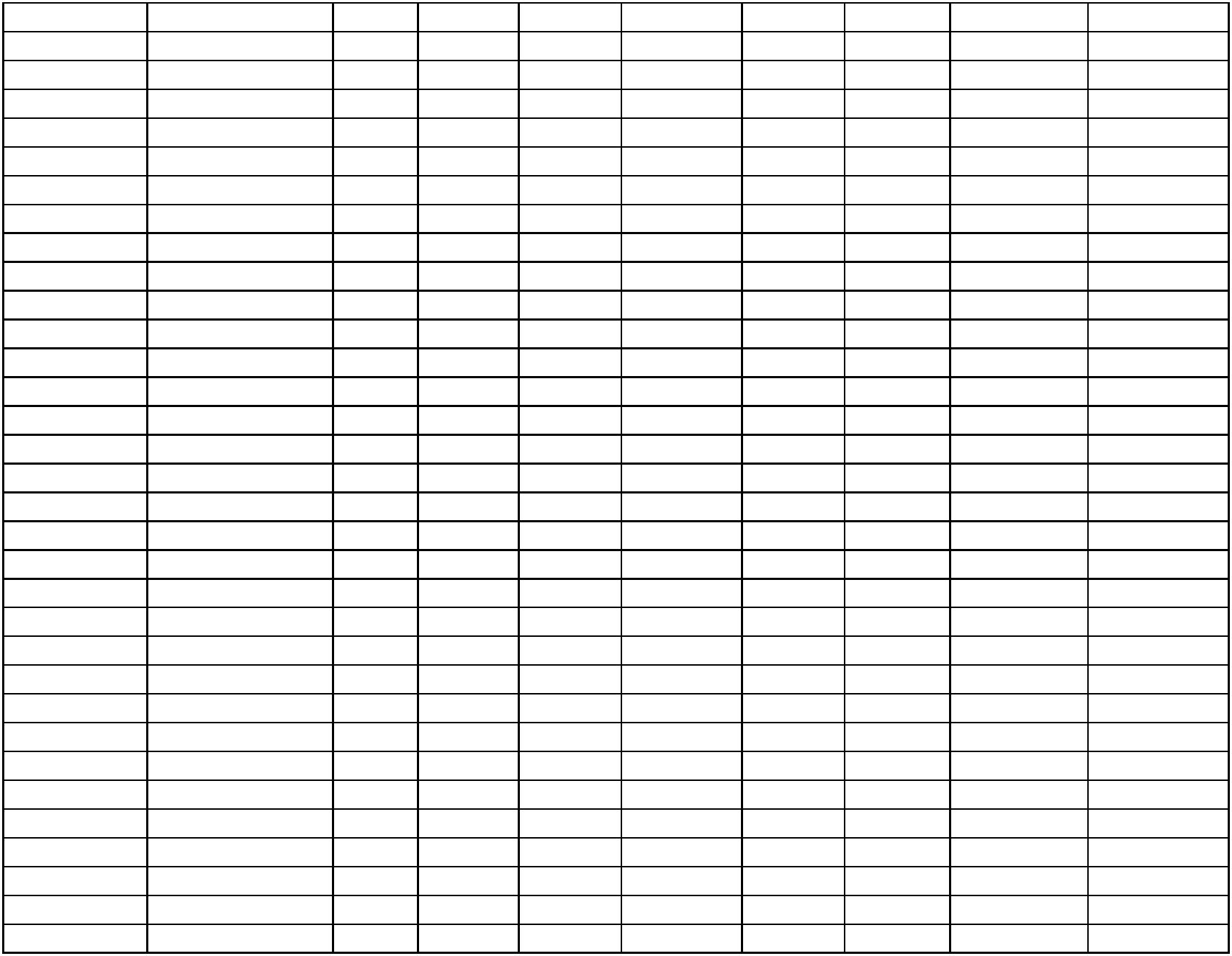
**

| **Lindmania** | **brachyphylla** | **BL** | **c3mes** | **55.28** | **5.83** | **54.19** | **23.58** | **0.90** | **0.05** |
| --- | --- | --- | --- | --- | --- | --- | --- | --- | --- |
| **Lindmania** | **cylindrostachya** | **BL** | **c3mes** | **64.08** | **22.00** | **42.25** | **42.25** | **0.87** | **0.13** |
| **Lindmania** | **geniculata** | **BL** | **c3mes** | **65.39** | **30.50** | **35.60** | **34.25** | **0.87** | **0.11** |
| **Lindmania** | **guianensis** | **BL** | **c3mes** | **45.52** | **6.33** | **84.00** | **48.33** | **0.93** | **0.09** |
| **Lindmania** | **holstii** | **BL** | **c3mes** | **58.21** | **1.17** | **48.75** | **7.00** | **0.88** | **0.01** |
| **Lindmania** | **marahuacae** | **BL** | **c3mes** | **43.06** | **3.33** | **104.43** | **7.25** | **0.97** | **0.05** |
| **Lindmania** | **navioides** | **BL** | **c3mes** | **65.43** | **33.00** | **40.74** | **48.50** | **0.90** | **0.07** |
| **Lindmania** | **nubigena** | **BL** | **c3mes** | **31.75** | **18.75** | **163.75** | **96.25** | **0.98** | **0.01** |
| **Lindmania** | **serrulata** | **BL** | **c3mes** | **53.93** | **23.50** | **66.98** | **91.25** | **0.91** | **0.10** |
| **Lindmania** | **steyermarkii** | **BL** | **c3mes** | **57.33** | **7.00** | **48.56** | **7.33** | **0.91** | **0.04** |
| **Lindmania** | **subsimplex** | **BL** | **c3mes** | **60.62** | **34.00** | **44.59** | **50.92** | **0.91** | **0.07** |
| **Lindmania** | **thyrsoidea** | **BL** | **c3mes** | **70.33** | **2.33** | **30.25** | **4.42** | **0.83** | **0.04** |
| **Lindmania** | **wurdackii** | **BL** | **c3mes** | **50.36** | **17.17** | **73.47** | **58.67** | **0.93** | **0.12** |
| **Navia** | **acaulis** | **Nav** | **c3mes** | **40.10** | **36.75** | **62.57** | **108.75** | **0.92** | **0.21** |
| **Navia** | **affinis** | **Nav** | **c3mes** | **30.36** | **16.75** | **168.94** | **80.08** | **0.99** | **0.01** |
| **Navia** | **aliciae** | **Nav** | **c3mes** | **51.75** | **42.50** | **79.50** | **87.92** | **0.95** | **0.04** |
| **Navia** | **angustifolia** | **Nav** | **c3mes** | **35.50** | **10.08** | **104.15** | **59.75** | **0.98** | **0.02** |
| **Navia** | **caulescens** | **Nav** | **c3mes** | **32.68** | **41.25** | **114.19** | **179.75** | **0.92** | **0.18** |
| **Navia** | **connata** | **Nav** | **c3mes** | **39.13** | **5.50** | **108.38** | **32.75** | **0.94** | **0.12** |
| **Navia** | **crispa** | **Nav** | **c3mes** | **50.31** | **49.25** | **80.42** | **145.25** | **0.80** | **0.01** |
| **Navia** | **duidae** | **Nav** | **c3mes** | **44.58** | **50.83** | **98.09** | **169.42** | **0.93** | **0.19** |
| **Navia** | **jauana** | **Nav** | **c3mes** | **50.15** | **4.00** | **72.48** | **9.75** | **0.95** | **0.01** |
| **Navia** | **linearis** | **Nav** | **c3mes** | **42.86** | **1.42** | **104.31** | **1.75** | **0.97** | **0.01** |
| **Navia** | **luzuloides** | **Nav** | **c3mes** | **50.90** | **5.25** | **70.90** | **13.50** | **0.95** | **0.01** |
| **Navia** | **maguirei** | **Nav** | **c3mes** | **51.57** | **5.75** | **46.70** | **8.15** | **0.90** | **0.03** |
| **Navia** | **nubicola** | **Nav** | **c3mes** | **36.20** | **23.00** | **130.52** | **109.00** | **0.97** | **0.06** |
| **Navia** | **octopoides** | **Nav** | **c3mes** | **46.00** | **4.50** | **88.72** | **16.50** | **0.94** | **0.04** |
| **Navia** | **ovoidea** | **Nav** | **c3mes** | **69.58** | **2.00** | **36.17** | **5.00** | **0.86** | **0.01** |
| **Navia** | **pauciflora** | **Nav** | **c3mes** | **43.31** | **3.17** | **105.64** | **15.25** | **0.97** | **0.02** |
| **Navia** | **pulvinata** | **Nav** | **c3mes** | **43.63** | **2.25** | **99.50** | **12.50** | **0.95** | **0.04** |
| **Navia** | **scirpiflora** | **Nav** | **c3mes** | **50.53** | **3.75** | **71.56** | **9.17** | **0.95** | **0.00** |
| **Navia** | **subpetiolata** | **Nav** | **c3mes** | **53.92** | **2.75** | **64.00** | **4.50** | **0.94** | **0.03** |
| **Navia** | **terramarae** | **Nav** | **c3mes** | **26.58** | **1.00** | **163.53** | **1.25** | **1.00** | **0.00** |

**
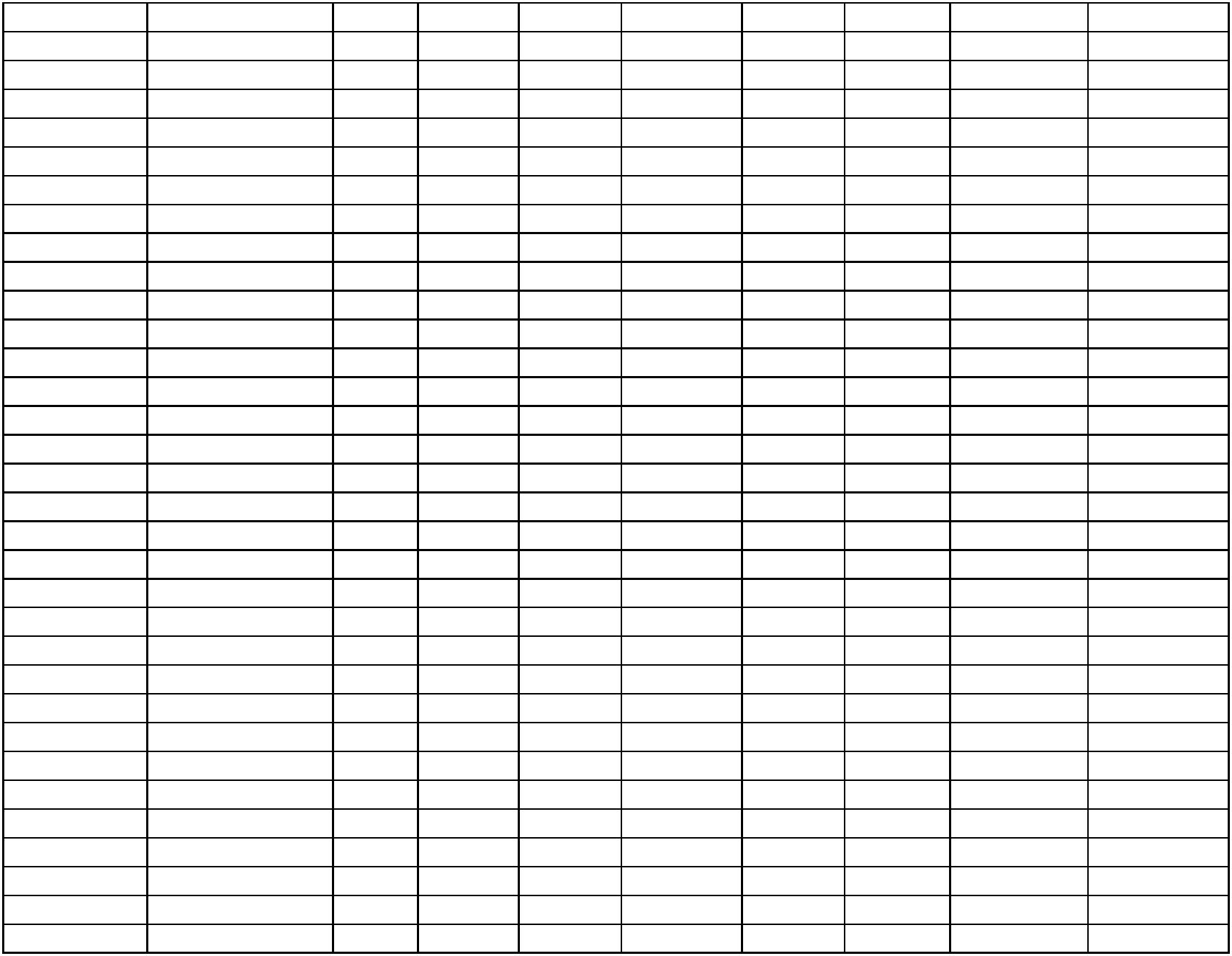
**

| **Neoglaziovia** | **variegata** | **CAMEDB** | **camterr** | **72.19** | **75.42** | **10.31** | **74.75** | **0.36** | **0.65** |
| --- | --- | --- | --- | --- | --- | --- | --- | --- | --- |
| **Ochagavia** | **carnea** | **C3EDB** | **c3terr** | **84.50** | **28.00** | **16.79** | **24.00** | **0.54** | **0.17** |
| **Ochagavia** | **litoralis** | **C3EDB** | **c3terr** | **93.60** | **54.00** | **16.67** | **62.25** | **0.51** | **0.50** |
| **Orthophytum** | **albopictum** | **CAMEDB** | **camterr** | **57.50** | **12.17** | **21.22** | **7.90** | **0.50** | **0.10** |
| **Orthophytum** | **amoenum** | **CAMEDB** | **camterr** | **56.27** | **23.75** | **18.92** | **12.25** | **0.48** | **0.13** |
| **Orthophytum** | **boudetianum** | **CAMEDB** | **camterr** | **58.08** | **8.25** | **31.28** | **11.75** | **0.72** | **0.11** |
| **Orthophytum** | **burle-marxii** | **CAMEDB** | **camterr** | **56.57** | **14.58** | **20.02** | **9.17** | **0.50** | **0.17** |
| **Orthophytum** | **compactum** | **CAMEDB** | **camterr** | **64.98** | **36.25** | **24.15** | **28.25** | **0.54** | **0.13** |
| **Orthophytum** | **diamantinense** | **CAMEDB** | **camterr** | **87.46** | **6.75** | **7.65** | **5.50** | **0.70** | **0.23** |
| **Orthophytum** | **disjunctum** | **CAMEDB** | **camterr** | **62.56** | **43.67** | **12.87** | **23.75** | **0.41** | **0.42** |
| **Orthophytum** | **foliosum** | **CAMEDB** | **camterr** | **59.33** | **46.00** | **28.70** | **26.75** | **0.62** | **0.29** |
| **Orthophytum** | **harleyi** | **CAMEDB** | **camterr** | **87.17** | **6.25** | **3.04** | **4.00** | **0.41** | **0.08** |
| **Orthophytum** | **hatschbachii** | **CAMEDB** | **camterr** | **63.91** | **30.17** | **19.56** | **21.83** | **0.52** | **0.17** |
| **Orthophytum** | **horridum** | **CAMEDB** | **camterr** | **80.14** | **24.75** | **10.64** | **16.75** | **0.48** | **0.16** |
| **Orthophytum** | **jabrense** | **CAMEDB** | **camterr** | **95.27** | **55.08** | **8.16** | **16.93** | **0.48** | **0.09** |
| **Orthophytum** | **lemei** | **CAMEDB** | **camterr** | **65.94** | **20.50** | **14.33** | **13.50** | **0.43** | **0.17** |
| **Orthophytum** | **leprosum** | **CAMEDB** | **camterr** | **72.70** | **33.25** | **14.00** | **19.75** | **0.43** | **0.13** |
| **Orthophytum** | **magalhaesii** | **CAMEDB** | **camterr** | **58.82** | **14.00** | **29.78** | **8.75** | **0.59** | **0.06** |
| **Orthophytum** | **maracasense** | **CAMEDB** | **camterr** | **52.26** | **54.50** | **16.78** | **21.08** | **0.39** | **0.27** |
| **Orthophytum** | **mello-barretoi** | **CAMEDB** | **camterr** | **85.25** | **7.50** | **8.79** | **6.50** | **0.70** | **0.29** |
| **Orthophytum** | **navioides** | **CAMEDB** | **camterr** | **53.97** | **24.67** | **21.24** | **11.33** | **0.50** | **0.12** |
| **Orthophytum** | **ophiuroides** | **CAMEDB** | **camterr** | **52.86** | **1.33** | **22.08** | **3.25** | **0.54** | **0.09** |
| **Orthophytum** | **piranianum** | **CAMEDB** | **camterr** | **90.41** | **0.50** | **5.28** | **1.17** | **0.49** | **0.06** |
| **Orthophytum** | **pseudovagans** | **CAMEDB** | **camterr** | **60.95** | **2.17** | **27.86** | **1.00** | **0.61** | **0.00** |
| **Orthophytum** | **riocontense** | **CAMEDB** | **camterr** | **67.15** | **30.50** | **17.56** | **20.75** | **0.50** | **0.12** |
| **Orthophytum** | **rubiginosum** | **CAMEDB** | **camterr** | **57.96** | **2.00** | **30.10** | **1.58** | **0.59** | **0.00** |
| **Orthophytum** | **saxicola** | **CAMEDB** | **camterr** | **52.55** | **65.25** | **21.37** | **72.25** | **0.42** | **0.59** |
| **Orthophytum** | **schulzianum** | **CAMEDB** | **camterr** | **86.54** | **2.50** | **7.42** | **3.50** | **0.72** | **0.11** |
| **Orthophytum** | **triunfense** | **CAMEDB** | **camterr** | **75.22** | **8.67** | **13.97** | **3.25** | **0.53** | **0.06** |
| **Orthophytum** | **ulei** | **CAMEDB** | **camterr** | **58.67** | **0.25** | **20.58** | **0.25** | **0.51** | **0.00** |
| **Orthophytum** | **zanonii** | **CAMEDB** | **camterr** | **63.67** | **0.25** | **26.42** | **0.50** | **0.63** | **0.02** |
| **Pitcairnia** | **abyssicola** | **PF** | **c3mes** | **58.08** | **9.75** | **29.06** | **2.00** | **0.69** | **0.15** |
| **Pitcairnia** | **aequatorialis** | **PF** | **c3mes** | **58.35** | **67.67** | **43.75** | **243.08** | **0.59** | **0.76** |

**
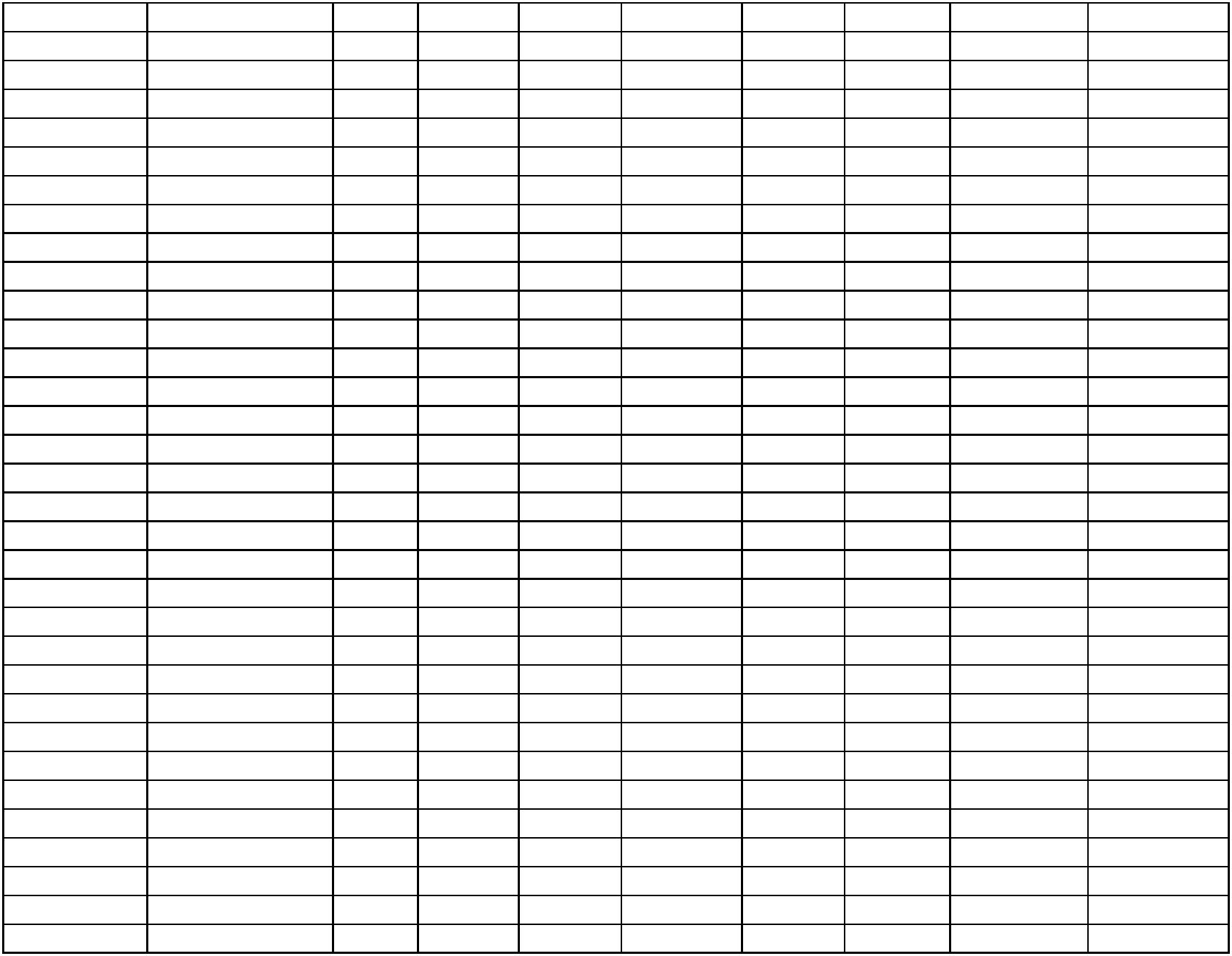
**

| **Pitcairnia** | **alata** | **PF** | **c3mes** | **32.28** | **21.67** | **87.64** | **128.00** | **0.82** | **0.26** |
| --- | --- | --- | --- | --- | --- | --- | --- | --- | --- |
| **Pitcairnia** | **albiflos** | **PF** | **c3mes** | **35.92** | **0.50** | **54.75** | **0.75** | **0.85** | **0.02** |
| **Pitcairnia** | **alborubra** | **PF** | **c3mes** | **38.92** | **4.00** | **100.42** | **29.50** | **0.98** | **0.02** |
| **Pitcairnia** | **alexanderi** | **PF** | **c3mes** | **18.60** | **9.50** | **162.32** | **44.58** | **1.00** | **0.01** |
| **Pitcairnia** | **amblyosperma** | **PF** | **c3mes** | **68.02** | **11.75** | **48.19** | **62.67** | **0.73** | **0.47** |
| **Pitcairnia** | **amboroensis** | **PF** | **c3mes** | **52.32** | **14.25** | **35.78** | **14.25** | **0.74** | **0.12** |
| **Pitcairnia** | **angustifolia** | **PF** | **c3mes** | **37.32** | **33.00** | **78.27** | **129.00** | **1.00** | **0.01** |
| **Pitcairnia** | **aphelandriflora** | **PF** | **c3mes** | **38.20** | **58.67** | **103.20** | **248.25** | **0.93** | **0.21** |
| **Pitcairnia** | **archeri** | **PF** | **c3mes** | **23.90** | **25.00** | **211.38** | **386.83** | **0.98** | **0.10** |
| **Pitcairnia** | **arcuata** | **PF** | **c3mes** | **42.41** | **61.50** | **104.29** | **278.50** | **0.93** | **0.38** |
| **Pitcairnia** | **arenaria** | **PF** | **c3mes** | **29.44** | **20.25** | **58.50** | **39.75** | **0.69** | **0.26** |
| **Pitcairnia** | **arida** | **PF** | **c3mes** | **34.75** | **13.75** | **88.06** | **88.50** | **0.93** | **0.14** |
| **Pitcairnia** | **armata** | **PF** | **c3mes** | **72.66** | **48.42** | **26.84** | **100.00** | **0.76** | **0.34** |
| **Pitcairnia** | **atrorubens** | **PF** | **c3mes** | **52.16** | **67.00** | **72.05** | **439.25** | **0.91** | **0.33** |
| **Pitcairnia** | **azouryi** | **PF** | **c3mes** | **62.01** | **33.33** | **22.73** | **25.25** | **0.56** | **0.43** |
| **Pitcairnia** | **bakeri** | **PF** | **c3mes** | **23.58** | **43.00** | **167.54** | **241.33** | **0.96** | **0.56** |
| **Pitcairnia** | **barbatostigma** | **PF** | **c3mes** | **60.95** | **6.75** | **28.04** | **4.75** | **0.62** | **0.06** |
| **Pitcairnia** | **barrigae** | **PF** | **c3mes** | **35.30** | **52.75** | **98.93** | **118.67** | **0.95** | **0.19** |
| **Pitcairnia** | **basincurva** | **PF** | **c3mes** | **42.52** | **2.67** | **77.92** | **12.00** | **0.94** | **0.04** |
| **Pitcairnia** | **bella** | **PF** | **c3mes** | **34.88** | **25.75** | **109.02** | **176.50** | **0.97** | **0.05** |
| **Pitcairnia** | **bergii** | **PF** | **c3mes** | **77.17** | **7.25** | **7.58** | **1.25** | **0.56** | **0.03** |
| **Pitcairnia** | **bicolor** | **PF** | **c3mes** | **27.52** | **26.50** | **164.50** | **351.17** | **0.99** | **0.05** |
| **Pitcairnia** | **biflora** | **PF** | **c3mes** | **22.82** | **26.00** | **134.34** | **98.58** | **0.94** | **0.26** |
| **Pitcairnia** | **billbergioides** | **PF** | **c3mes** | **90.79** | **100.50** | **14.13** | **48.00** | **0.40** | **0.37** |
| **Pitcairnia** | **brachysperma** | **PF** | **c3mes** | **37.52** | **31.00** | **88.20** | **144.80** | **0.88** | **0.27** |
| **Pitcairnia** | **brackeana** | **PF** | **c3mes** | **49.02** | **13.67** | **47.75** | **80.33** | **0.79** | **0.22** |
| **Pitcairnia** | **bradei** | **PF** | **c3mes** | **87.92** | **14.00** | **5.39** | **3.50** | **0.55** | **0.25** |
| **Pitcairnia** | **breedlovei** | **PF** | **c3mes** | **95.27** | **17.00** | **6.65** | **12.25** | **0.49** | **0.26** |
| **Pitcairnia** | **brevicalycina** | **PF** | **c3mes** | **50.98** | **41.33** | **33.66** | **57.33** | **0.74** | **0.32** |
| **Pitcairnia** | **brittoniana** | **PF** | **c3mes** | **46.15** | **60.75** | **86.51** | **241.42** | **0.93** | **0.29** |
| **Pitcairnia** | **bromeliifolia** | **PF** | **c3mes** | **46.00** | **23.00** | **45.50** | **22.25** | **0.76** | **0.03** |
| **Pitcairnia** | **brongniartiana** | **PF** | **c3mes** | **32.88** | **56.67** | **99.98** | **169.25** | **0.94** | **0.19** |
| **Pitcairnia** | **brunnescens** | **PF** | **c3mes** | **30.73** | **47.50** | **88.95** | **140.75** | **0.91** | **0.22** |

**
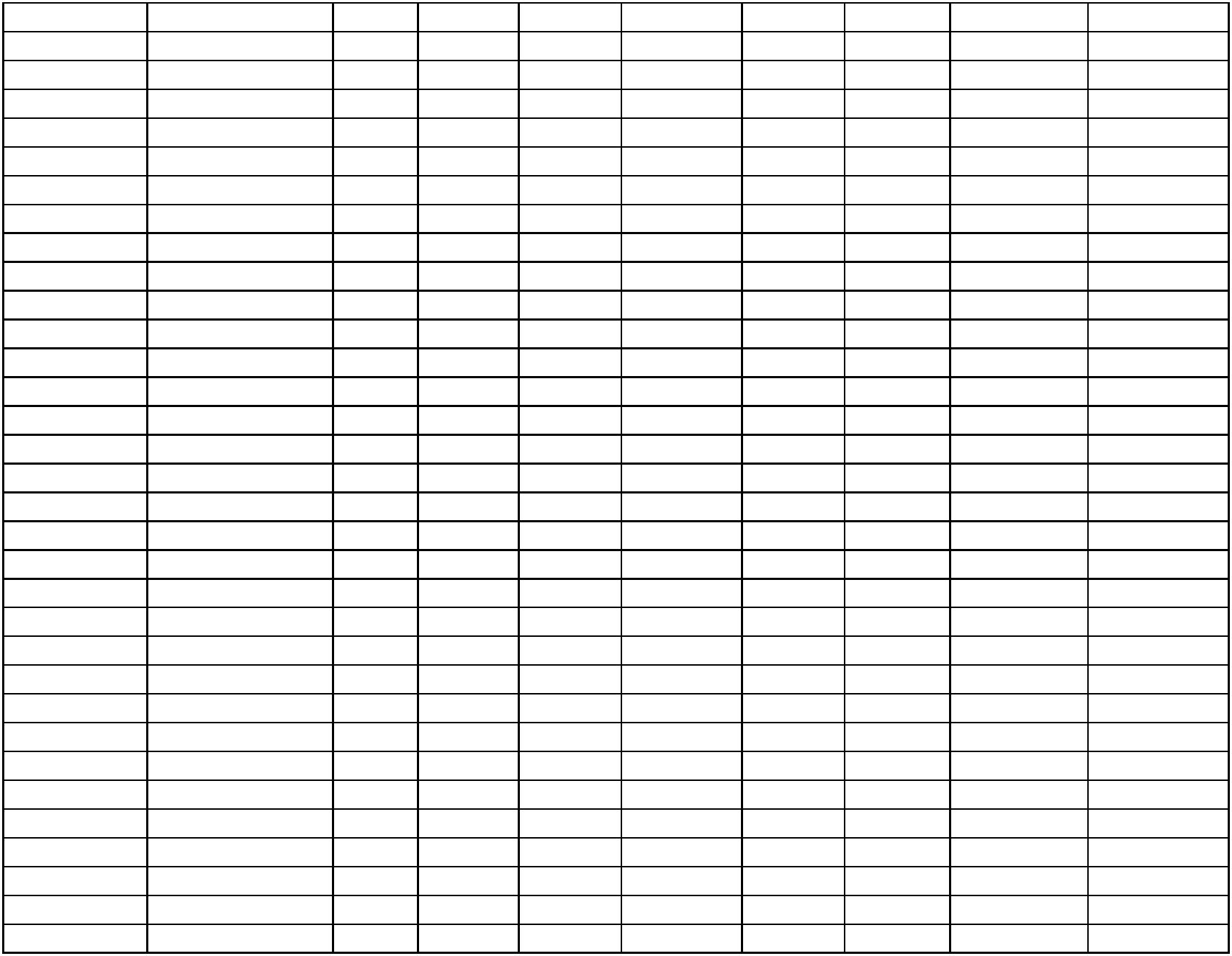
**

| **Pitcairnia** | **bulbosa** | **PF** | **c3mes** | **47.81** | **60.50** | **92.31** | **206.50** | **0.89** | **0.34** |
| --- | --- | --- | --- | --- | --- | --- | --- | --- | --- |
| **Pitcairnia** | **calcicola** | **PF** | **c3mes** | **88.68** | **13.67** | **2.77** | **4.67** | **0.67** | **0.11** |
| **Pitcairnia** | **calderonii** | **PF** | **c3mes** | **77.06** | **24.58** | **17.82** | **29.33** | **0.72** | **0.17** |
| **Pitcairnia** | **cardenasii** | **PF** | **c3mes** | **77.33** | **30.00** | **8.75** | **7.75** | **0.48** | **0.31** |
| **Pitcairnia** | **caricifolia** | **PF** | **c3mes** | **48.07** | **67.00** | **64.86** | **203.75** | **0.88** | **0.35** |
| **Pitcairnia** | **carinata** | **PF** | **c3mes** | **56.89** | **61.50** | **34.63** | **66.50** | **0.80** | **0.26** |
| **Pitcairnia** | **chiapensis** | **PF** | **c3mes** | **74.83** | **55.00** | **22.67** | **42.75** | **0.65** | **0.23** |
| **Pitcairnia** | **chiquitana** | **PF** | **c3mes** | **51.17** | **1.25** | **22.58** | **0.75** | **0.56** | **0.01** |
| **Pitcairnia** | **chiriquensis** | **PF** | **c3mes** | **63.29** | **10.75** | **34.92** | **39.25** | **0.84** | **0.06** |
| **Pitcairnia** | **clarkii** | **PF** | **c3mes** | **77.58** | **1.00** | **33.11** | **1.67** | **0.81** | **0.00** |
| **Pitcairnia** | **commixta** | **PF** | **c3mes** | **49.60** | **53.67** | **54.77** | **152.58** | **0.84** | **0.30** |
| **Pitcairnia** | **compostelae** | **PF** | **c3mes** | **106.35** | **15.25** | **2.73** | **1.75** | **0.52** | **0.08** |
| **Pitcairnia** | **condorensis** | **PF** | **c3mes** | **14.00** | **0.10** | **151.04** | **1.42** | **0.99** | **0.00** |
| **Pitcairnia** | **corallina** | **PF** | **c3mes** | **22.36** | **30.33** | **136.24** | **141.00** | **0.94** | **0.30** |
| **Pitcairnia** | **costata** | **PF** | **c3mes** | **24.58** | **7.00** | **108.67** | **106.50** | **0.93** | **0.18** |
| **Pitcairnia** | **cremersii** | **PF** | **c3mes** | **45.42** | **4.00** | **64.14** | **13.75** | **0.89** | **0.04** |
| **Pitcairnia** | **crinita** | **PF** | **c3mes** | **47.67** | **0.25** | **90.25** | **0.75** | **0.89** | **0.00** |
| **Pitcairnia** | **ctenophylla** | **PF** | **c3mes** | **66.73** | **36.75** | **41.86** | **87.50** | **0.86** | **0.18** |
| **Pitcairnia** | **cubensis** | **PF** | **c3mes** | **43.06** | **42.25** | **69.89** | **97.25** | **0.82** | **0.37** |
| **Pitcairnia** | **curvidens** | **PF** | **c3mes** | **79.94** | **10.50** | **13.94** | **13.75** | **0.77** | **0.17** |
| **Pitcairnia** | **cuzcoensis** | **PF** | **c3mes** | **55.38** | **71.17** | **44.33** | **93.00** | **0.69** | **0.23** |
| **Pitcairnia** | **cylindrostachya** | **PF** | **c3mes** | **109.15** | **13.35** | **3.84** | **2.75** | **0.53** | **0.15** |
| **Pitcairnia** | **decidua** | **PF** | **c3mes** | **58.94** | **29.75** | **31.06** | **29.00** | **0.74** | **0.29** |
| **Pitcairnia** | **dendroidea** | **PF** | **c3mes** | **43.39** | **56.50** | **50.90** | **71.67** | **0.83** | **0.26** |
| **Pitcairnia** | **deroosei** | **PF** | **c3mes** | **37.77** | **9.00** | **47.42** | **31.50** | **0.88** | **0.14** |
| **Pitcairnia** | **diffusa** | **PF** | **c3mes** | **32.95** | **69.17** | **91.38** | **228.00** | **0.89** | **0.54** |
| **Pitcairnia** | **dodsonii** | **PF** | **c3mes** | **24.28** | **16.25** | **130.02** | **204.50** | **0.93** | **0.50** |
| **Pitcairnia** | **domingensis** | **PF** | **c3mes** | **32.22** | **32.13** | **78.30** | **81.07** | **0.83** | **0.48** |
| **Pitcairnia** | **echinata** | **PF** | **c3mes** | **30.81** | **43.00** | **120.97** | **252.75** | **0.89** | **0.40** |
| **Pitcairnia** | **elizabethae** | **PF** | **c3mes** | **62.44** | **7.00** | **33.06** | **4.25** | **0.79** | **0.17** |
| **Pitcairnia** | **elongata** | **PF** | **c3mes** | **59.18** | **86.33** | **58.53** | **263.33** | **0.70** | **0.51** |
| **Pitcairnia** | **elvirae** | **PF** | **c3mes** | **23.70** | **35.08** | **186.86** | **256.75** | **0.95** | **0.34** |
| **Pitcairnia** | **encholirioides** | **PF** | **c3mes** | **61.97** | **15.00** | **26.77** | **11.00** | **0.81** | **0.12** |

**
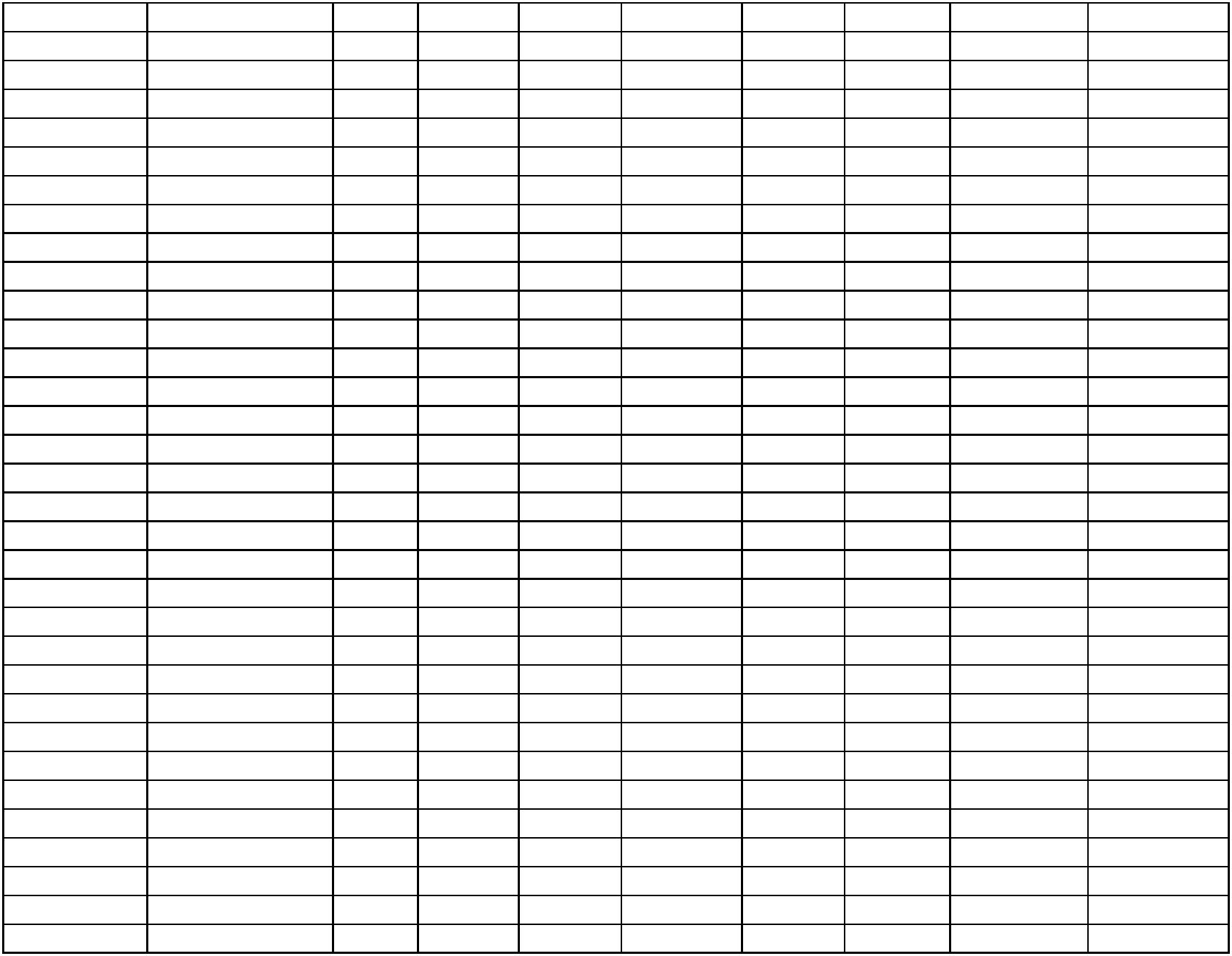
**

| **Pitcairnia** | **ensifolia** | **PF** | **c3mes** | **81.25** | **7.75** | **3.89** | **5.25** | **0.71** | **0.14** |
| --- | --- | --- | --- | --- | --- | --- | --- | --- | --- |
| **Pitcairnia** | **ferrell-ingramiae** | **PF** | **c3mes** | **29.37** | **59.67** | **117.43** | **138.92** | **0.97** | **0.19** |
| **Pitcairnia** | **filispina** | **PF** | **c3mes** | **67.08** | **17.25** | **37.08** | **33.25** | **0.85** | **0.10** |
| **Pitcairnia** | **flammea** | **PF** | **c3mes** | **53.21** | **70.17** | **42.84** | **104.10** | **0.80** | **0.43** |
| **Pitcairnia** | **flexuosa** | **PF** | **c3mes** | **94.41** | **52.00** | **6.08** | **28.25** | **0.59** | **0.39** |
| **Pitcairnia** | **fluvialis** | **PF** | **c3mes** | **40.86** | **4.75** | **97.17** | **11.50** | **0.95** | **0.05** |
| **Pitcairnia** | **fruticosa** | **PF** | **c3mes** | **33.94** | **4.00** | **91.58** | **18.33** | **0.97** | **0.01** |
| **Pitcairnia** | **fuertesii** | **PF** | **c3mes** | **44.03** | **30.75** | **48.01** | **62.00** | **0.72** | **0.34** |
| **Pitcairnia** | **funkiae** | **PF** | **c3mes** | **51.42** | **29.00** | **73.92** | **105.50** | **0.91** | **0.16** |
| **Pitcairnia** | **fusca** | **PF** | **c3mes** | **55.47** | **63.17** | **34.02** | **138.17** | **0.75** | **0.47** |
| **Pitcairnia** | **geyskesii** | **PF** | **c3mes** | **59.20** | **31.50** | **40.45** | **34.92** | **0.79** | **0.22** |
| **Pitcairnia** | **glaziovii** | **PF** | **c3mes** | **61.53** | **20.00** | **31.99** | **39.75** | **0.82** | **0.22** |
| **Pitcairnia** | **goudae** | **PF** | **c3mes** | **22.04** | **14.92** | **191.56** | **194.25** | **0.94** | **0.21** |
| **Pitcairnia** | **guzmanioides** | **PF** | **c3mes** | **68.24** | **88.75** | **50.77** | **153.75** | **0.76** | **0.56** |
| **Pitcairnia** | **halophila** | **PF** | **c3mes** | **65.82** | **44.00** | **33.61** | **92.33** | **0.84** | **0.28** |
| **Pitcairnia** | **harlingii** | **PF** | **c3mes** | **18.36** | **8.75** | **239.12** | **243.00** | **1.00** | **0.00** |
| **Pitcairnia** | **harrylutheri** | **PF** | **c3mes** | **20.69** | **15.67** | **160.18** | **49.00** | **1.00** | **0.02** |
| **Pitcairnia** | **heterophylla** | **PF** | **c3mes** | **74.99** | **91.33** | **24.31** | **155.25** | **0.73** | **0.82** |
| **Pitcairnia** | **hintoniana** | **PF** | **c3mes** | **104.85** | **16.00** | **4.25** | **5.75** | **0.54** | **0.14** |
| **Pitcairnia** | **hirtzii** | **PF** | **c3mes** | **21.22** | **11.58** | **145.24** | **100.50** | **0.96** | **0.20** |
| **Pitcairnia** | **hooveri** | **PF** | **c3mes** | **34.56** | **26.25** | **50.40** | **143.50** | **0.86** | **0.23** |
| **Pitcairnia** | **imbricata** | **PF** | **c3mes** | **68.34** | **77.17** | **37.19** | **163.67** | **0.80** | **0.62** |
| **Pitcairnia** | **integrifolia** | **PF** | **c3mes** | **51.65** | **58.83** | **45.21** | **105.92** | **0.75** | **0.45** |
| **Pitcairnia** | **irwiniana** | **PF** | **c3mes** | **78.14** | **15.00** | **6.86** | **12.00** | **0.70** | **0.26** |
| **Pitcairnia** | **juncoides** | **PF** | **c3mes** | **52.24** | **59.50** | **76.40** | **154.25** | **0.89** | **0.34** |
| **Pitcairnia** | **kalbreyeri** | **PF** | **c3mes** | **40.12** | **50.50** | **100.14** | **295.00** | **0.95** | **0.22** |
| **Pitcairnia** | **karwinskyana** | **PF** | **c3mes** | **101.02** | **44.58** | **5.90** | **17.50** | **0.52** | **0.52** |
| **Pitcairnia** | **kniphofioides** | **PF** | **c3mes** | **35.17** | **3.25** | **65.75** | **19.75** | **0.84** | **0.10** |
| **Pitcairnia** | **kressii** | **PF** | **c3mes** | **37.01** | **23.50** | **110.83** | **78.75** | **0.97** | **0.06** |
| **Pitcairnia** | **lanuginosa** | **PF** | **c3mes** | **64.04** | **67.33** | **28.53** | **133.67** | **0.73** | **0.58** |
| **Pitcairnia** | **lehmannii** | **PF** | **c3mes** | **33.77** | **49.33** | **114.16** | **234.33** | **0.90** | **0.55** |
| **Pitcairnia** | **leprosa** | **PF** | **c3mes** | **108.33** | **9.00** | **2.47** | **4.25** | **0.52** | **0.17** |
| **Pitcairnia** | **lignosa** | **PF** | **c3mes** | **53.85** | **63.08** | **43.81** | **127.42** | **0.67** | **0.38** |

**
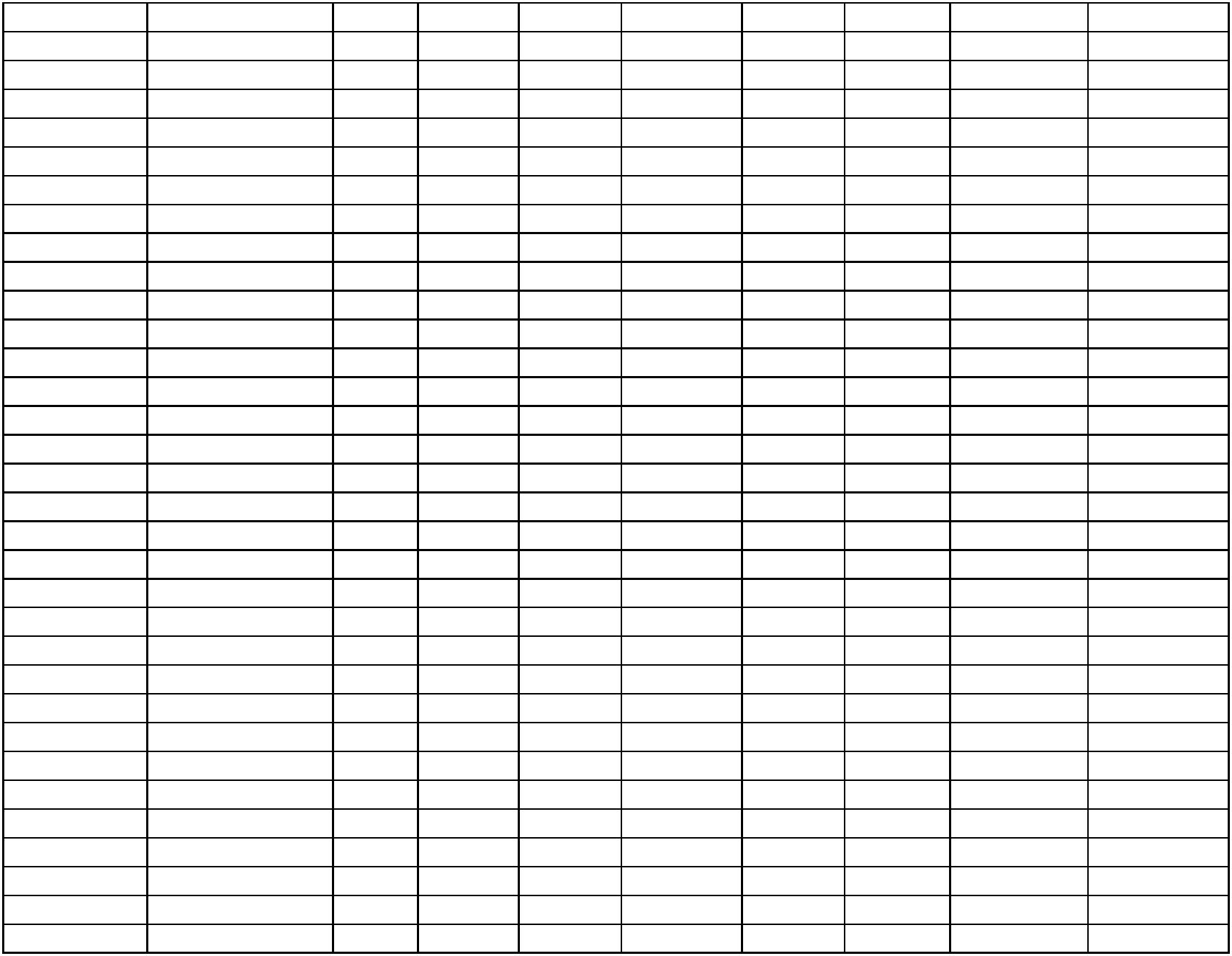
**

| **Pitcairnia** | **longipes** | **PF** | **c3mes** | **37.00** | **28.75** | **165.53** | **274.75** | **0.93** | **0.17** |
| --- | --- | --- | --- | --- | --- | --- | --- | --- | --- |
| **Pitcairnia** | **longissimiflora** | **PF** | **c3mes** | **55.92** | **2.75** | **21.92** | **24.25** | **0.57** | **0.34** |
| **Pitcairnia** | **luteyniorum** | **PF** | **c3mes** | **33.83** | **25.00** | **60.57** | **135.67** | **0.90** | **0.22** |
| **Pitcairnia** | **lymansmithiana** | **PF** | **c3mes** | **38.84** | **4.25** | **112.22** | **62.75** | **0.97** | **0.04** |
| **Pitcairnia** | **macarenensis** | **PF** | **c3mes** | **37.63** | **43.33** | **79.65** | **143.50** | **0.92** | **0.14** |
| **Pitcairnia** | **macranthera** | **PF** | **c3mes** | **42.62** | **73.33** | **152.25** | **499.33** | **0.90** | **0.38** |
| **Pitcairnia** | **maidifolia** | **PF** | **c3mes** | **50.32** | **80.83** | **62.63** | **254.92** | **0.87** | **0.68** |
| **Pitcairnia** | **marinii** | **PF** | **c3mes** | **20.94** | **1.50** | **160.39** | **0.67** | **1.00** | **0.01** |
| **Pitcairnia** | **megasepala** | **PF** | **c3mes** | **54.40** | **40.50** | **51.87** | **103.00** | **0.86** | **0.38** |
| **Pitcairnia** | **melanopoda** | **PF** | **c3mes** | **25.04** | **7.42** | **66.56** | **7.15** | **0.72** | **0.04** |
| **Pitcairnia** | **meridensis** | **PF** | **c3mes** | **50.58** | **44.25** | **47.15** | **69.58** | **0.83** | **0.32** |
| **Pitcairnia** | **micheliana** | **PF** | **c3mes** | **108.15** | **5.95** | **1.85** | **2.50** | **0.37** | **0.19** |
| **Pitcairnia** | **minicorallina** | **PF** | **c3mes** | **17.11** | **7.67** | **152.33** | **51.33** | **0.95** | **0.08** |
| **Pitcairnia** | **mituensis** | **PF** | **c3mes** | **22.75** | **4.50** | **200.08** | **52.25** | **1.00** | **0.00** |
| **Pitcairnia** | **modesta** | **PF** | **c3mes** | **109.08** | **5.75** | **2.58** | **1.75** | **0.54** | **0.02** |
| **Pitcairnia** | **mucida** | **PF** | **c3mes** | **28.92** | **13.75** | **143.39** | **138.50** | **0.97** | **0.08** |
| **Pitcairnia** | **multiflora** | **PF** | **c3mes** | **60.58** | **58.00** | **32.98** | **108.25** | **0.82** | **0.57** |
| **Pitcairnia** | **multiramosa** | **PF** | **c3mes** | **84.44** | **45.25** | **22.21** | **91.60** | **0.56** | **0.54** |
| **Pitcairnia** | **neillii** | **PF** | **c3mes** | **20.14** | **6.00** | **147.61** | **75.58** | **0.98** | **0.13** |
| **Pitcairnia** | **nigra** | **PF** | **c3mes** | **53.56** | **76.67** | **53.19** | **242.50** | **0.85** | **0.41** |
| **Pitcairnia** | **nobilis** | **PF** | **c3mes** | **22.36** | **2.08** | **81.17** | **28.17** | **0.63** | **0.20** |
| **Pitcairnia** | **nubigena** | **PF** | **c3mes** | **57.31** | **40.50** | **21.63** | **18.25** | **0.72** | **0.16** |
| **Pitcairnia** | **nuda** | **PF** | **c3mes** | **60.61** | **44.50** | **48.81** | **104.75** | **0.81** | **0.36** |
| **Pitcairnia** | **oaxacana** | **PF** | **c3mes** | **107.33** | **18.50** | **2.84** | **5.83** | **0.53** | **0.09** |
| **Pitcairnia** | **oblongifolia** | **PF** | **c3mes** | **79.92** | **8.25** | **7.36** | **1.25** | **0.58** | **0.07** |
| **Pitcairnia** | **occidentalis** | **PF** | **c3mes** | **40.72** | **28.75** | **96.64** | **128.75** | **0.95** | **0.16** |
| **Pitcairnia** | **palmeri** | **PF** | **c3mes** | **105.28** | **42.25** | **6.67** | **7.83** | **0.47** | **0.41** |
| **Pitcairnia** | **palmoides** | **PF** | **c3mes** | **40.88** | **103.75** | **114.85** | **243.00** | **0.90** | **0.51** |
| **Pitcairnia** | **paniculata** | **PF** | **c3mes** | **55.10** | **73.00** | **37.30** | **158.42** | **0.67** | **0.62** |
| **Pitcairnia** | **paraguayensis** | **PF** | **c3mes** | **33.67** | **1.00** | **44.25** | **3.75** | **0.74** | **0.03** |
| **Pitcairnia** | **patentiflora** | **PF** | **c3mes** | **39.32** | **55.50** | **101.80** | **206.92** | **0.93** | **0.19** |
| **Pitcairnia** | **pavonii** | **PF** | **c3mes** | **62.38** | **153.58** | **21.29** | **55.00** | **0.55** | **0.76** |
| **Pitcairnia** | **peruana** | **PF** | **c3mes** | **27.83** | **11.75** | **59.75** | **6.00** | **0.70** | **0.05** |

**
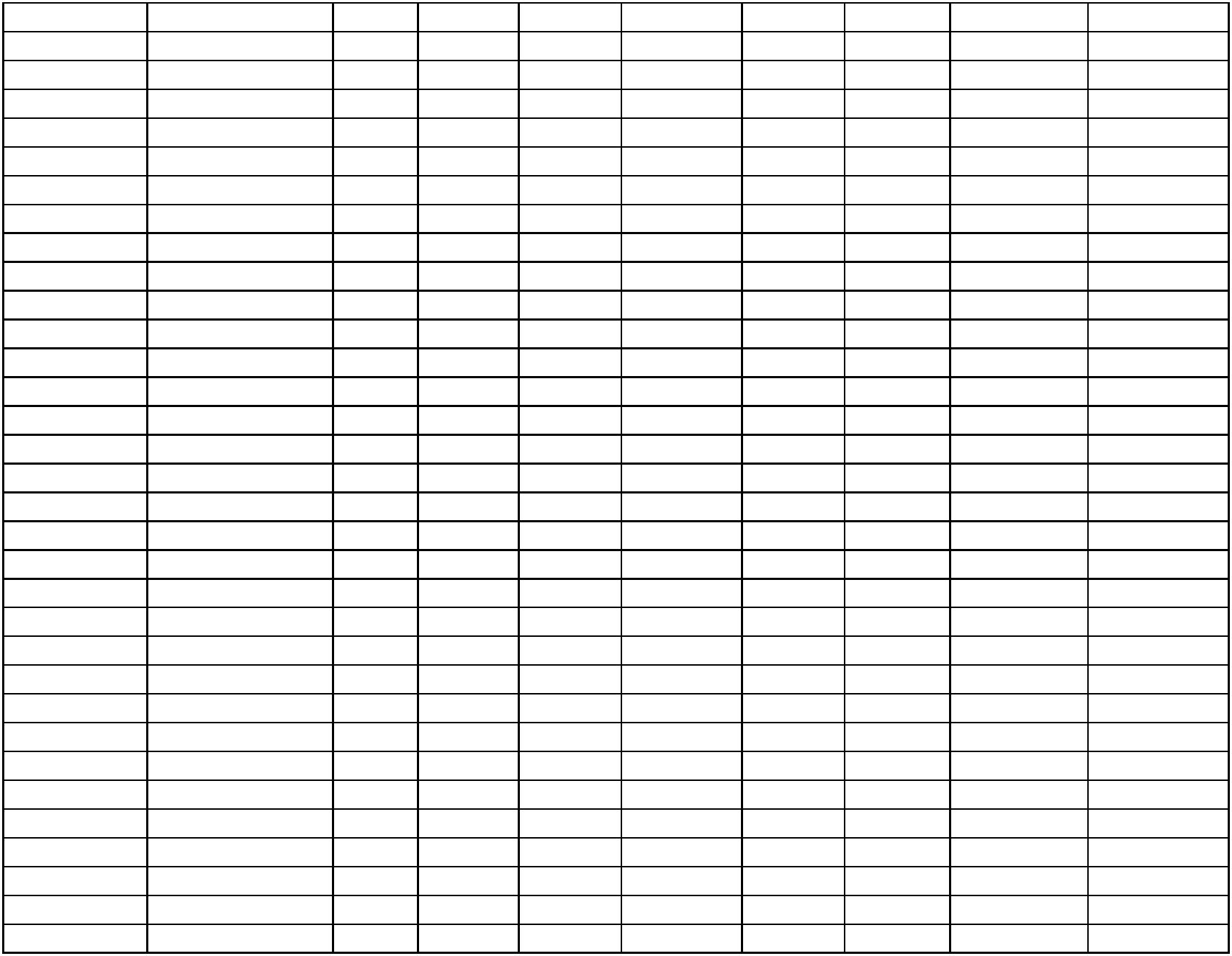
**

| **Pitcairnia** | **petraea** | **PF** | **c3mes** | **40.69** | **8.17** | **41.81** | **8.92** | **0.67** | **0.05** |
| --- | --- | --- | --- | --- | --- | --- | --- | --- | --- |
| **Pitcairnia** | **pomacochae** | **PF** | **c3mes** | **40.33** | **43.25** | **56.53** | **147.00** | **0.67** | **0.48** |
| **Pitcairnia** | **poortmanii** | **PF** | **c3mes** | **22.68** | **32.00** | **116.66** | **159.75** | **0.90** | **0.35** |
| **Pitcairnia** | **prolifera** | **PF** | **c3mes** | **107.53** | **11.17** | **3.39** | **1.50** | **0.48** | **0.06** |
| **Pitcairnia** | **pruinosa** | **PF** | **c3mes** | **66.85** | **47.00** | **38.40** | **129.58** | **0.79** | **0.32** |
| **Pitcairnia** | **puberula** | **PF** | **c3mes** | **79.73** | **51.50** | **16.08** | **46.17** | **0.67** | **0.38** |
| **Pitcairnia** | **pulverulenta** | **PF** | **c3mes** | **51.65** | **25.00** | **37.07** | **71.42** | **0.66** | **0.41** |
| **Pitcairnia** | **pungens** | **PF** | **c3mes** | **52.83** | **133.92** | **29.35** | **164.33** | **0.66** | **0.93** |
| **Pitcairnia** | **punicea** | **PF** | **c3mes** | **56.00** | **26.25** | **75.79** | **92.50** | **0.90** | **0.18** |
| **Pitcairnia** | **quesnelioides** | **PF** | **c3mes** | **62.78** | **1.33** | **45.43** | **10.80** | **0.86** | **0.02** |
| **Pitcairnia** | **recurvata** | **PF** | **c3mes** | **66.44** | **47.67** | **48.83** | **81.42** | **0.82** | **0.63** |
| **Pitcairnia** | **reflexiflora** | **PF** | **c3mes** | **47.49** | **72.00** | **61.18** | **151.33** | **0.73** | **0.51** |
| **Pitcairnia** | **ringens** | **PF** | **c3mes** | **79.56** | **35.00** | **28.44** | **46.42** | **0.66** | **0.54** |
| **Pitcairnia** | **riparia** | **PF** | **c3mes** | **29.28** | **85.50** | **114.55** | **255.83** | **0.85** | **0.54** |
| **Pitcairnia** | **roseana** | **PF** | **c3mes** | **106.13** | **8.25** | **2.18** | **2.50** | **0.47** | **0.16** |
| **Pitcairnia** | **rubiginosa** | **PF** | **c3mes** | **30.52** | **31.25** | **140.24** | **213.92** | **0.96** | **0.11** |
| **Pitcairnia** | **rundelliana** | **PF** | **c3mes** | **50.65** | **33.75** | **62.14** | **110.50** | **0.91** | **0.24** |
| **Pitcairnia** | **samuelssonii** | **PF** | **c3mes** | **58.94** | **6.67** | **21.44** | **4.67** | **0.70** | **0.12** |
| **Pitcairnia** | **sastrei** | **PF** | **c3mes** | **51.07** | **14.75** | **55.08** | **35.25** | **0.86** | **0.11** |
| **Pitcairnia** | **saxicola** | **PF** | **c3mes** | **69.20** | **24.50** | **25.77** | **25.75** | **0.80** | **0.14** |
| **Pitcairnia** | **scandens** | **PF** | **c3mes** | **29.29** | **36.25** | **68.50** | **114.75** | **0.75** | **0.41** |
| **Pitcairnia** | **sceptriformis** | **PF** | **c3mes** | **25.78** | **49.17** | **167.35** | **192.75** | **0.96** | **0.31** |
| **Pitcairnia** | **sceptrigera** | **PF** | **c3mes** | **74.85** | **98.75** | **45.73** | **189.25** | **0.77** | **0.70** |
| **Pitcairnia** | **schultzei** | **PF** | **c3mes** | **58.54** | **29.00** | **22.65** | **60.25** | **0.74** | **0.42** |
| **Pitcairnia** | **semaphora** | **PF** | **c3mes** | **39.22** | **18.75** | **95.38** | **53.67** | **0.98** | **0.03** |
| **Pitcairnia** | **simulans** | **PF** | **c3mes** | **38.72** | **61.67** | **77.92** | **151.50** | **0.89** | **0.66** |
| **Pitcairnia** | **sodiroi** | **PF** | **c3mes** | **52.87** | **60.25** | **37.93** | **157.25** | **0.79** | **0.37** |
| **Pitcairnia** | **spectabilis** | **PF** | **c3mes** | **28.46** | **36.75** | **108.18** | **274.50** | **0.94** | **0.22** |
| **Pitcairnia** | **sprucei** | **PF** | **c3mes** | **25.68** | **42.50** | **138.68** | **154.25** | **0.97** | **0.15** |
| **Pitcairnia** | **squarrosa** | **PF** | **c3mes** | **34.50** | **36.25** | **99.04** | **146.08** | **0.93** | **0.20** |
| **Pitcairnia** | **staminea** | **PF** | **c3mes** | **40.61** | **23.00** | **47.83** | **16.67** | **0.78** | **0.20** |
| **Pitcairnia** | **stenophylla** | **PF** | **c3mes** | **30.23** | **20.92** | **76.19** | **43.00** | **0.80** | **0.18** |
| **Pitcairnia** | **stevensonii** | **PF** | **c3mes** | **73.07** | **30.50** | **54.17** | **88.50** | **0.81** | **0.35** |

**
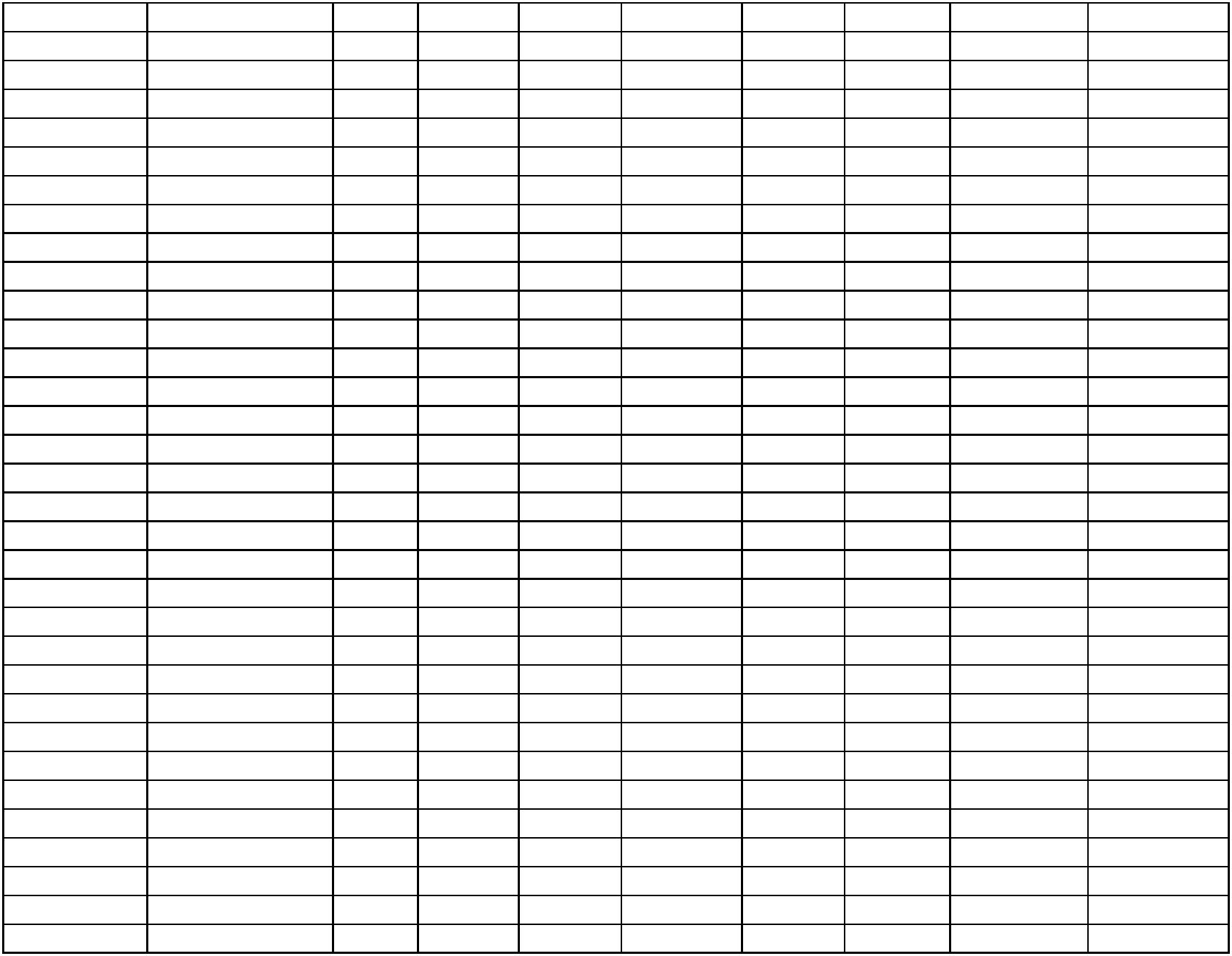
**

| **Pitcairnia** | **suaveolens** | **PF** | **c3mes** | **58.24** | **9.33** | **39.17** | **10.33** | **0.85** | **0.29** |
| --- | --- | --- | --- | --- | --- | --- | --- | --- | --- |
| **Pitcairnia** | **susannae** | **PF** | **c3mes** | **27.78** | **4.67** | **126.39** | **31.50** | **0.99** | **0.01** |
| **Pitcairnia** | **tarapotensis** | **PF** | **c3mes** | **37.86** | **34.17** | **51.91** | **63.00** | **0.71** | **0.23** |
| **Pitcairnia** | **tillandsioides** | **PF** | **c3mes** | **106.25** | **4.25** | **3.67** | **2.00** | **0.57** | **0.03** |
| **Pitcairnia** | **tillii** | **PF** | **c3mes** | **24.83** | **3.33** | **156.06** | **36.00** | **1.00** | **0.01** |
| **Pitcairnia** | **torresiana** | **PF** | **c3mes** | **71.13** | **17.50** | **9.63** | **18.50** | **0.67** | **0.16** |
| **Pitcairnia** | **trianae** | **PF** | **c3mes** | **35.31** | **64.00** | **73.44** | **181.92** | **0.80** | **0.52** |
| **Pitcairnia** | **truncata** | **PF** | **c3mes** | **44.29** | **68.00** | **50.16** | **72.50** | **0.71** | **0.24** |
| **Pitcairnia** | **turbinella** | **PF** | **c3mes** | **36.43** | **30.50** | **103.85** | **194.50** | **0.92** | **0.41** |
| **Pitcairnia** | **uaupensis** | **PF** | **c3mes** | **29.87** | **46.33** | **138.32** | **194.50** | **0.97** | **0.14** |
| **Pitcairnia** | **ulei** | **PF** | **c3mes** | **78.51** | **22.42** | **5.23** | **7.75** | **0.71** | **0.31** |
| **Pitcairnia** | **undulata** | **PF** | **c3mes** | **53.08** | **17.75** | **89.56** | **55.00** | **0.71** | **0.31** |
| **Pitcairnia** | **unilateralis** | **PF** | **c3mes** | **111.50** | **21.50** | **3.42** | **10.25** | **0.46** | **0.15** |
| **Pitcairnia** | **valerioi** | **PF** | **c3mes** | **51.77** | **55.25** | **65.44** | **174.75** | **0.91** | **0.33** |
| **Pitcairnia** | **vallisoletana** | **PF** | **c3mes** | **98.42** | **3.25** | **7.25** | **1.50** | **0.51** | **0.13** |
| **Pitcairnia** | **wendlandii** | **PF** | **c3mes** | **47.15** | **73.25** | **82.85** | **187.33** | **0.93** | **0.41** |
| **Puya** | **aequatorialis** | **Puy** | **c3terr** | **47.40** | **75.50** | **24.19** | **62.75** | **0.87** | **0.37** |
| **Puya** | **alpestris** | **Puy** | **c3terr** | **96.50** | **50.00** | **8.02** | **27.75** | **0.57** | **0.18** |
| **Puya** | **angelensis** | **Puy** | **c3terr** | **32.67** | **8.50** | **35.36** | **9.83** | **0.90** | **0.15** |
| **Puya** | **angulonis** | **Puy** | **c3terr** | **53.72** | **59.75** | **22.72** | **35.50** | **0.68** | **0.49** |
| **Puya** | **angusta** | **Puy** | **c3camterr** | **65.07** | **27.00** | **14.15** | **47.33** | **1.00** | **0.00** |
| **Puya** | **argentea** | **Puy** | **c3terr** | **54.32** | **59.50** | **71.10** | **151.58** | **0.70** | **0.31** |
| **Puya** | **aristeguietae** | **Puy** | **c3terr** | **58.89** | **2.08** | **19.31** | **13.25** | **0.53** | **0.52** |
| **Puya** | **atra** | **Puy** | **c3terr** | **95.42** | **59.00** | **4.38** | **13.50** | **0.70** | **0.45** |
| **Puya** | **berteroana** | **Puy** | **c3terr** | **72.60** | **8.58** | **10.61** | **4.42** | **0.59** | **0.07** |
| **Puya** | **berteroniana** | **Puy** | **camterr** | **96.41** | **31.00** | **7.86** | **17.00** | **0.18** | **0.28** |
| **Puya** | **bicolor** | **Puy** | **c3terr** | **45.08** | **13.00** | **43.54** | **49.42** | **0.49** | **0.09** |
| **Puya** | **boliviensis** | **Puy** | **c3camterr** | **83.40** | **97.50** | **16.24** | **114.00** | **0.61** | **0.53** |
| **Puya** | **brackeana** | **Puy** | **c3terr** | **19.79** | **2.08** | **56.63** | **19.50** | **0.67** | **0.24** |
| **Puya** | **brittoniana** | **Puy** | **c3terr** | **71.63** | **15.67** | **10.95** | **9.00** | **0.63** | **0.17** |
| **Puya** | **cajasensis** | **Puy** | **c3terr** | **38.24** | **34.75** | **34.79** | **43.50** | **0.96** | **0.00** |
| **Puya** | **cardenasii** | **Puy** | **c3camterr** | **77.67** | **46.50** | **35.95** | **95.10** | **0.89** | **0.13** |
| **Puya** | **castellanosii** | **Puy** | **camterr** | **117.17** | **3.50** | **0.10** | **0.10** | **0.41** | **0.05** |

**
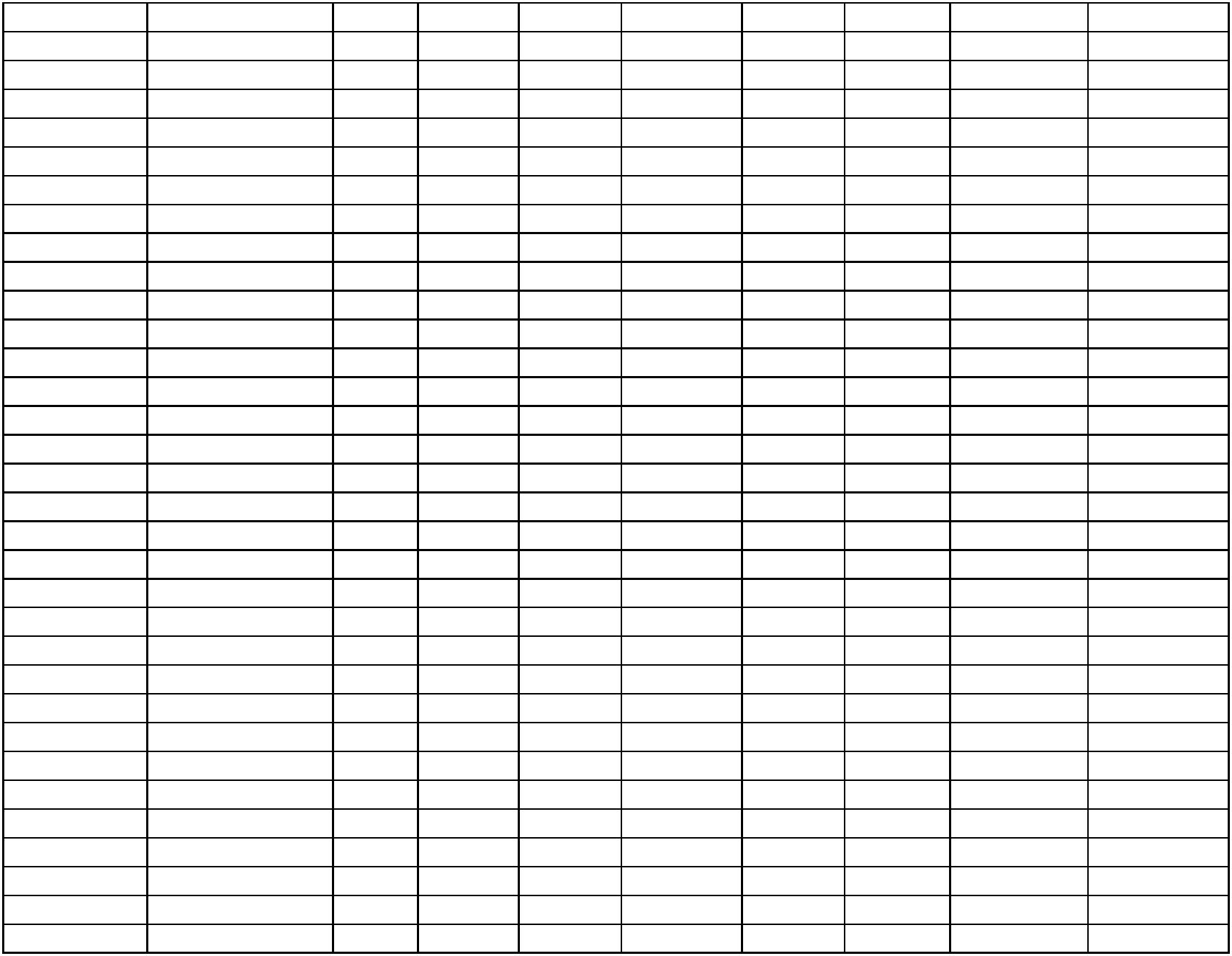
**

| **Puya** | **chilensis** | **Puy** | **camterr** | **96.41** | **31.00** | **7.86** | **17.00** | **0.71** | **0.49** |
| --- | --- | --- | --- | --- | --- | --- | --- | --- | --- |
| **Puya** | **claudiae** | **Puy** | **c3terr** | **75.83** | **0.50** | **9.92** | **2.50** | **0.45** | **0.21** |
| **Puya** | **clava-herculis** | **Puy** | **c3terr** | **23.70** | **36.67** | **59.50** | **55.58** | **0.56** | **0.52** |
| **Puya** | **coerulea** | **Puy** | **c3camterr** | **94.98** | **35.50** | **5.41** | **14.65** | **0.17** | **0.11** |
| **Puya** | **cryptantha** | **Puy** | **c3terr** | **38.51** | **27.75** | **52.32** | **104.92** | **0.74** | **0.10** |
| **Puya** | **ctenorhyncha** | **Puy** | **camterr** | **78.96** | **12.50** | **7.06** | **3.25** | **0.45** | **0.18** |
| **Puya** | **cuevae** | **Puy** | **c3terr** | **42.90** | **23.17** | **43.42** | **21.92** | **0.37** | **0.04** |
| **Puya** | **cylindrica** | **Puy** | **camterr** | **77.17** | **92.00** | **27.25** | **54.75** | **0.90** | **0.07** |
| **Puya** | **dasylirioides** | **Puy** | **c3terr** | **57.91** | **18.00** | **49.80** | **44.50** | **0.63** | **0.31** |
| **Puya** | **densiflora*** | **Puy** | **camterr** | **75.25** | **3.25** | **7.92** | **5.25** | **0.58** | **0.18** |
| **Puya** | **dyckioides** | **Puy** | **c3camterr** | **100.80** | **32.10** | **2.16** | **7.20** | **0.35** | **0.57** |
| **Puya** | **entre-riosensis** | **Puy** | **c3terr** | **91.00** | **1.75** | **2.33** | **1.25** | **0.42** | **0.25** |
| **Puya** | **eryngioides** | **Puy** | **c3terr** | **38.88** | **56.42** | **49.24** | **62.75** | **0.90** | **0.18** |
| **Puya** | **exigua** | **Puy** | **c3terr** | **28.85** | **22.67** | **66.93** | **138.33** | **0.82** | **0.37** |
| **Puya** | **ferreyrae** | **Puy** | **camterr** | **61.53** | **35.08** | **40.65** | **90.25** | **0.44** | **0.20** |
| **Puya** | **ferruginea** | **Puy** | **c3terr** | **62.97** | **103.25** | **28.33** | **174.33** | **0.75** | **0.41** |
| **Puya** | **floccosa** | **Puy** | **c3camterr** | **55.59** | **46.83** | **42.64** | **106.50** | **0.55** | **0.23** |
| **Puya** | **fulgens** | **Puy** | **c3terr** | **25.83** | **29.00** | **98.27** | **128.42** | **0.89** | **0.34** |
| **Puya** | **glandulosa** | **Puy** | **c3camterr** | **67.25** | **50.25** | **21.42** | **53.00** | **0.81** | **0.03** |
| **Puya** | **glaucovirens** | **Puy** | **c3terr** | **34.25** | **37.75** | **85.25** | **144.75** | **0.19** | **0.97** |
| **Puya** | **glomerifera** | **Puy** | **c3terr** | **39.18** | **64.33** | **34.20** | **60.00** | **0.48** | **0.08** |
| **Puya** | **goudotiana** | **Puy** | **c3terr** | **73.41** | **34.60** | **20.20** | **92.60** | **0.48** | **0.53** |
| **Puya** | **harmsii** | **Puy** | **camterr** | **98.25** | **21.25** | **2.39** | **6.50** | **0.17** | **0.08** |
| **Puya** | **herrerae** | **Puy** | **c3camterr** | **64.01** | **36.00** | **17.77** | **51.75** | **0.19** | **0.01** |
| **Puya** | **herzogii** | **Puy** | **c3terr** | **67.35** | **38.00** | **26.49** | **90.35** | **0.69** | **0.07** |
| **Puya** | **hirtzii** | **Puy** | **c3terr** | **41.36** | **4.42** | **25.06** | **22.67** | **0.54** | **0.63** |
| **Puya** | **hofstenii** | **Puy** | **c3terr** | **70.42** | **60.00** | **37.20** | **97.60** | **0.67** | **0.44** |
| **Puya** | **humilis** | **Puy** | **c3camterr** | **81.41** | **52.00** | **29.50** | **109.75** | **0.33** | **0.38** |
| **Puya** | **hutchisonii** | **Puy** | **c3terr** | **37.07** | **20.50** | **52.56** | **34.00** | **0.45** | **0.20** |
| **Puya** | **joergensenii** | **Puy** | **c3terr** | **47.98** | **4.75** | **26.10** | **4.42** | **0.61** | **0.43** |
| **Puya** | **killipii** | **Puy** | **c3terr** | **45.78** | **6.33** | **24.15** | **12.75** | **0.40** | **0.01** |
| **Puya** | **kuntzeana** | **Puy** | **c3terr** | **64.48** | **33.50** | **30.04** | **87.00** | **0.64** | **0.27** |
| **Puya** | **lanata** | **Puy** | **camterr** | **50.13** | **19.67** | **27.85** | **35.75** | **0.66** | **0.47** |

**
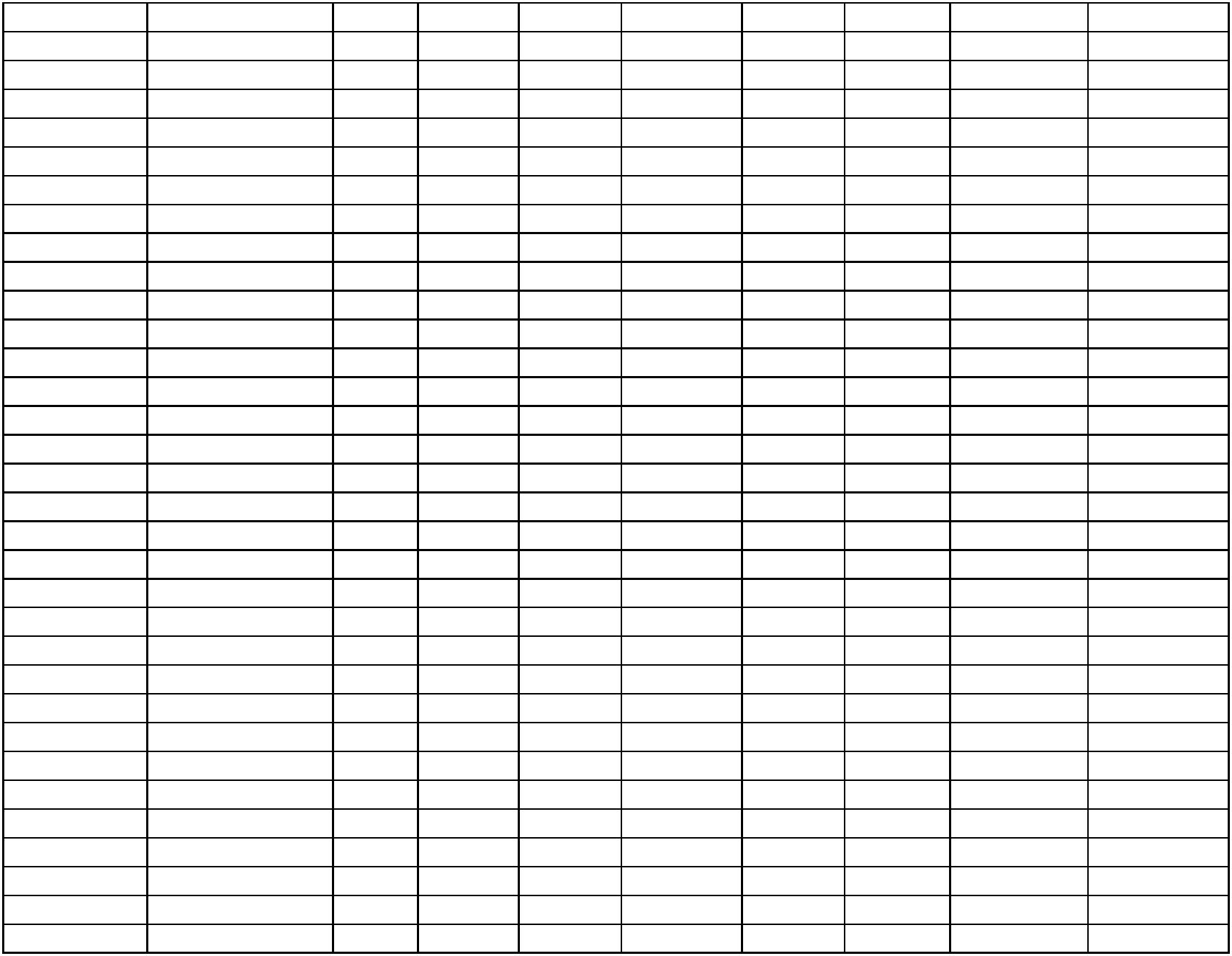
**

| **Puya** | **lasiopoda** | **Puy** | **c3terr** | **63.47** | **19.33** | **26.36** | **30.83** | **0.85** | **0.41** |
| --- | --- | --- | --- | --- | --- | --- | --- | --- | --- |
| **Puya** | **leptostachya** | **Puy** | **c3terr** | **68.56** | **28.25** | **16.10** | **19.83** | **0.53** | **0.08** |
| **Puya** | **lilloi** | **Puy** | **c3terr** | **93.29** | **54.25** | **4.97** | **21.75** | **0.61** | **0.10** |
| **Puya** | **lineata*** | **Puy** | **c3terr** | **42.55** | **27.00** | **39.27** | **25.25** | **0.23** | **0.32** |
| **Puya** | **longispina** | **Puy** | **c3terr** | **24.75** | **6.67** | **54.83** | **18.67** | **0.70** | **0.07** |
| **Puya** | **maculata** | **Puy** | **c3terr** | **35.96** | **42.25** | **56.17** | **150.33** | **0.70** | **0.45** |
| **Puya** | **micrantha** | **Puy** | **c3terr** | **89.83** | **53.00** | **20.79** | **114.00** | **0.92** | **0.20** |
| **Puya** | **mirabilis** | **Puy** | **c3terr** | **94.35** | **63.20** | **9.29** | **97.60** | **0.75** | **0.03** |
| **Puya** | **mollis** | **Puy** | **c3terr** | **73.41** | **34.60** | **20.20** | **92.60** | **0.81** | **0.34** |
| **Puya** | **nana** | **Puy** | **c3terr** | **75.24** | **10.50** | **10.62** | **11.50** | **0.95** | **0.07** |
| **Puya** | **navarroana** | **Puy** | **c3terr** | **19.68** | **9.00** | **71.75** | **104.25** | **0.57** | **0.61** |
| **Puya** | **nitida** | **Puy** | **c3terr** | **37.93** | **28.50** | **41.62** | **57.25** | **0.91** | **0.49** |
| **Puya** | **novarae** | **Puy** | **c3terr** | **108.38** | **3.25** | **0.10** | **0.10** | **0.83** | **0.48** |
| **Puya** | **nutans** | **Puy** | **c3terr** | **35.86** | **28.92** | **31.88** | **33.50** | **0.64** | **0.24** |
| **Puya** | **obconica** | **Puy** | **c3terr** | **40.31** | **28.33** | **61.06** | **135.67** | **0.69** | **0.18** |
| **Puya** | **ochroleuca** | **Puy** | **c3terr** | **29.92** | **1.00** | **98.58** | **12.00** | **0.28** | **0.22** |
| **Puya** | **olivacea** | **Puy** | **c3camterr** | **73.44** | **35.25** | **23.57** | **91.10** | **0.66** | **0.26** |
| **Puya** | **parviflora** | **Puy** | **c3terr** | **42.19** | **4.50** | **42.25** | **8.00** | **0.61** | **0.36** |
| **Puya** | **pattersoniae** | **Puy** | **c3terr** | **31.83** | **25.50** | **34.18** | **23.75** | **0.67** | **0.37** |
| **Puya** | **pearcei** | **Puy** | **c3terr** | **64.63** | **17.00** | **20.31** | **19.67** | **0.75** | **0.36** |
| **Puya** | **pichinchae** | **Puy** | **c3terr** | **58.78** | **43.75** | **20.17** | **3.08** | **0.95** | **0.07** |
| **Puya** | **pygmaea** | **Puy** | **c3terr** | **35.84** | **34.33** | **41.72** | **156.00** | **0.76** | **0.19** |
| **Puya** | **raimondii** | **Puy** | **c3terr** | **86.41** | **64.75** | **7.19** | **54.33** | **0.60** | **0.13** |
| **Puya** | **reducta** | **Puy** | **c3terr** | **59.53** | **53.50** | **39.03** | **122.08** | **0.48** | **0.38** |
| **Puya** | **retrorsa** | **Puy** | **c3terr** | **31.02** | **9.00** | **33.57** | **20.50** | **0.56** | **0.20** |
| **Puya** | **robin-fosteri** | **Puy** | **c3terr** | **53.84** | **25.50** | **38.72** | **81.50** | **0.53** | **0.34** |
| **Puya** | **roldanii** | **Puy** | **c3terr** | **36.79** | **0.50** | **69.56** | **34.50** | **0.10** | **0.03** |
| **Puya** | **roseana** | **Puy** | **c3terr** | **51.53** | **6.42** | **22.61** | **2.50** | **0.67** | **0.24** |
| **Puya** | **rusbyi** | **Puy** | **c3terr** | **55.36** | **24.75** | **37.79** | **81.35** | **0.73** | **0.24** |
| **Puya** | **sanctae-crucis** | **Puy** | **c3terr** | **64.77** | **25.00** | **12.56** | **6.75** | **0.88** | **0.10** |
| **Puya** | **sanctae-martae** | **Puy** | **c3terr** | **51.61** | **33.00** | **39.92** | **8.50** | **0.59** | **0.04** |
| **Puya** | **santosii** | **Puy** | **c3terr** | **44.25** | **23.00** | **34.38** | **25.25** | **0.54** | **0.99** |
| **Puya** | **smithii** | **Puy** | **c3camterr** | **97.79** | **14.10** | **2.51** | **4.70** | **0.61** | **0.39** |

**
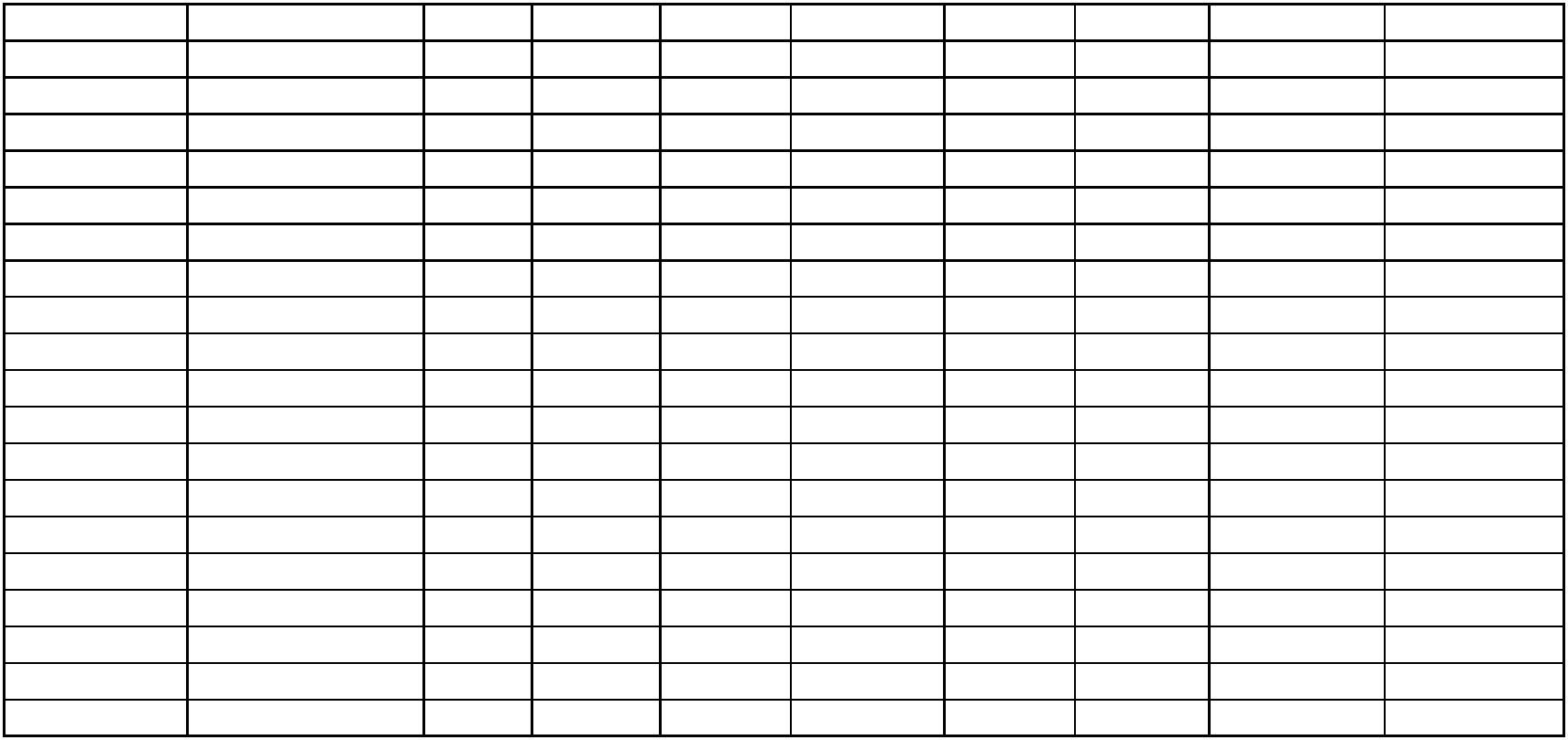
**

| **Puya** | **sodiroana** | **Puy** | **c3terr** | **49.75** | **0.10** | **19.50** | **0.10** | **0.62** | **0.52** |
| --- | --- | --- | --- | --- | --- | --- | --- | --- | --- |
| **Puya** | **spathacea** | **Puy** | **c3terr** | **74.52** | **55.50** | **8.21** | **23.00** | **0.50** | **0.06** |
| **Puya** | **stenothyrsa** | **Puy** | **camterr** | **71.70** | **46.80** | **17.61** | **37.30** | **0.68** | **0.54** |
| **Puya** | **thomasiana** | **Puy** | **c3terr** | **59.61** | **55.58** | **25.14** | **42.17** | **0.37** | **0.05** |
| **Puya** | **tillii** | **Puy** | **c3terr** | **45.25** | **47.00** | **56.83** | **91.00** | **0.85** | **0.35** |
| **Puya** | **trianae** | **Puy** | **c3terr** | **36.66** | **24.50** | **48.32** | **93.00** | **0.60** | **0.60** |
| **Puya** | **tuberosa** | **Puy** | **c3terr** | **74.60** | **31.75** | **9.88** | **18.25** | **0.69** | **0.39** |
| **Puya** | **tunarensis** | **Puy** | **camterr** | **89.04** | **23.42** | **6.55** | **10.50** | **0.86** | **0.16** |
| **Puya** | **venusta** | **Puy** | **camterr** | **104.37** | **28.50** | **1.70** | **17.00** | **0.39** | **0.76** |
| **Puya** | **vestita** | **Puy** | **c3terr** | **50.64** | **5.08** | **18.53** | **3.92** | **0.72** | **0.15** |
| **Puya** | **volcanensis** | **Puy** | **c3terr** | **108.12** | **9.08** | **0.50** | **1.25** | **0.96** | **0.00** |
| **Puya** | **weberbaueri** | **Puy** | **c3terr** | **56.53** | **39.33** | **37.19** | **85.75** | **0.34** | **0.38** |
| **Puya** | **weberiana** | **Puy** | **camterr** | **104.01** | **40.00** | **1.67** | **7.75** | **0.27** | **0.54** |
| **Puya** | **weddelliana** | **Puy** | **c3terr** | **66.67** | **28.50** | **37.37** | **95.85** | **0.46** | **0.65** |
| **Puya** | **wrightii** | **Puy** | **camterr** | **43.67** | **15.00** | **33.25** | **33.50** | **0.70** | **0.43** |
| **Puya** | **yakespala** | **Puy** | **c3terr** | **85.10** | **66.00** | **1.08** | **3.25** | **0.70** | **0.17** |
| **Sequencia** | **serrata** | **Nav** | **c3mes** | **31.87** | **15.00** | **106.67** | **74.33** | **0.97** | **0.04** |
| **Steyerbromelia** | **deflexa** | **Nav** | **c3mes** | **44.81** | **3.33** | **96.00** | **21.67** | **0.96** | **0.04** |
| **Steyerbromelia** | **discolor** | **Nav** | **c3mes** | **43.90** | **3.75** | **100.40** | **12.75** | **0.97** | **0.05** |
| **Steyerbromelia** | **ramosa** | **Nav** | **c3mes** | **41.23** | **23.00** | **110.00** | **93.50** | **0.97** | **0.05** |
|  |  |  |  |  |  |  |  |  |  |

**Supporting Information Table S2**. Correlations (showing linear regression coefficients, *m*) between sample size and calculated ranges for bioclimatic variables across all sampled terrestrial bromeliads (*n* = 564).

| **Bioclimatic variable range** | **Linear correlation with *n*** | | |
| --- | --- | --- | --- |
|  | ***m*** | ***r*^2^** | ***P*** |
| **AI (mm mm^-1^)** | 0.72 | 29.31 | < 0.001 |
| **AET/PET (mm mm^-1^)** | 0.61 | 20.79 | < 0.001 |
| **MAP (mm)** | 0.75 | 28.68 | < 0.001 |
| **P_dry_­ (mm)** | 0.85 | 26.54 | < 0.001 |
| **P_seas_ (%)** | 0.76 | 34.37 | < 0.001 |

Across all species there were statistically significant positive correlations between the number of presence points and the range of values observed for each bioclimatic variable, implying that species with few presence points tended to show narrower estimated habitat ranges. This could either represent an artefact of under-recording, or could be consistent with species rarity being associated with narrow habitat ranges.

**Supporting Information Table S3.** Extent of taxon sampling for distributional and bioclimatic analyses across terrestrial bromeliad genera and subfamilies.

| **Subfamily** | **Genus** | **Taxa sampled/Total taxa** | **Percentage coverage (%)** |
| --- | --- | --- | --- |
| Brocchinioideae | *Brocchinia* | 8/21 | 38.1 |
| Pitcairnioideae | *Deuterocohnia* | 10/18 | 55.6 |
|  | *Dyckia* | 51/150 | 34.7 |
|  | *Encholirium* | 18/26 | 69.2 |
|  | *Fosterella* | 25/31 | 80.6 |
|  | *Pitcairnia* | 183/395 | 46.3 |
| Lindmanioideae | *Connellia* | 3/6 | 50.0 |
|  | *Lindmania* | 14/38 | 36.8 |
| Hechtioideae | *Hechtia* | 26/67 | 38.8 |
| Navioideae | *Brewcaria* | 3/6 | 50.0 |
|  | *Cottendorfia* | 1/1 | 100.0 |
|  | *Navia* | 20/91 | 22.0 |
|  | *Sequencia* | 1/1 | 100.0 |
|  | *Steyerbromelia* | 3/6 | 50.0 |
| Puyoideae | *Puya* | 99/227 | 43.6 |
| Bromelioideae | *Ananas* | 5/6 | 83.3 |
|  | *Bromelia* | 23/61 | 37.7 |
|  | *Cryptanthus* | 16/67 | 23.9 |
|  | *Deinacanthon* | 1/1 | 100.0 |
|  | *Disteganthus* | 3/3 | 100.0 |
|  | *Fascicularia* | 1/1 | 100.0 |
|  | *Fernseea* | ½ | 50.0 |
|  | *Greigia* | 18/35 | 51.4 |
|  | *Neoglaziovia* | 1/3 | 33.3 |
|  | *Ochagavia* | 2/4 | 50.0 |
|  | *Orthophytum* | 28/68 | 41.2 |
| **Terrestrial Bromeliaceae** |  | **564/1335** | 42.0 |

Estimates of hydrological bioclimatic habitat position and range were generated for a total of 564 species using distributional data retrieved from GBIF. The number of species represented in each terrestrial bromeliad genus, and the proportion of species in each of these genera represented, is displayed above. There was no discernible taxonomic bias in terms of percentage genus coverage at the subfamilial level (AOV: *F* = 0.365, *p* = 0.892).

**Supporting Information Table S4**. Pairwise linear correlations between genus-mean positions for bioclimatic indices based on data for 564 terrestrial bromeliad species.

|  | **AI (mm mm^-1^)** | **MAP (mm)** | **P_dry_ (mm)** | **P_seas_ (%)** |
| --- | --- | --- | --- | --- |
| **AET/PET (mm mm^-1^)** | +, *r*^2^ = 0.77  *p* < 0.001 | +, *r*^2^ = 0.80  *p* < 0.001 | +, *r*^2^ = 0.72  *p* < 0.001 | -, *r*^2^ = 0.69  *p* < 0.001 |
| **AI (mm mm^-1^)** |  | +, *r*^2^ = 0.83  *p* < 0.001 | +, *r*^2^ = 0.75  *p* < 0.001 | -, *r*^2^ = 0.49  *p* < 0.001 |
| **MAP (mm)** |  |  | +, *r*^2^ = 0.89  *p* < 0.001 | -, *r*^2^ = 0.61  *p* < 0.001 |
| **P_dry_ (mm)** |  |  |  | -, *r*^2^ = 0.72  *p* < 0.001 |

Pairwise linear regression was performed between mean univariate habitat positions for all genera to assess the degree of collinearity. All pairs showed strong, statistically significant correlations, summarised above. Of all the variables, P_seas_ was the most weakly coordinated with other variables, as was borne out in PCA-based estimation of multivariate hydrological habitat position and range. This suggests that variation in overall moisture requirement could be partly decoupled from variation in tolerance of environmental seasonality.
